# Identification of Potent Inhibitors Targeting EGFR and HER3 for Effective Treatment of Chemoresistance in Non-Small Cell Lung Cancer

**DOI:** 10.3390/molecules28124850

**Published:** 2023-06-19

**Authors:** Ayed A. Dera, Sumera Zaib, Areeba, Nadia Hussain, Nehal Rana, Hira Javed, Imtiaz Khan

**Affiliations:** 1 Department of Clinical Laboratory Sciences, College of Applied Medical Sciences, King Khalid University, Abha 62529, Saudi Arabia; 2 Department of Basic and Applied Chemistry, Faculty of Science and Technology, University of Central Punjab, Lahore 54590, Pakistan; 3 Department of Pharmaceutical Sciences, College of Pharmacy, Al Ain University, Al Ain P.O. Box 64141, United Arab Emirates; 4 AAU Health and Biomedical Research Center, Al Ain University, Abu Dhabi P.O. Box 144534, United Arab Emirates; 5 Department of Chemistry and Manchester Institute of Biotechnology, The University of Manchester, 131 Princess Street, Manchester M1 7DN, UK

**Keywords:** chemoresistance, epidermal growth factor receptor, HER3, lung cancer, pharmacophore design

## Abstract

Non-small cell lung cancer (NSCLC) is the most common form of lung cancer. Despite the existence of various therapeutic options, NSCLC is still a major health concern due to its aggressive nature and high mutation rate. Consequently, HER3 has been selected as a target protein along with EGFR because of its limited tyrosine kinase activity and ability to activate PI3/AKT pathway responsible for therapy failure. We herein used a BioSolveIT suite to identify potent inhibitors of EGFR and HER3. The schematic process involves screening of databases for constructing compound library comprising of 903 synthetic compounds (602 for EGFR and 301 for HER3) followed by pharmacophore modeling. The best docked poses of compounds with the druggable binding site of respective proteins were selected according to pharmacophore designed by SeeSAR version 12.1.0. Subsequently, preclinical analysis was performed via an online server SwissADME and potent inhibitors were selected. Compound **4k** and **4m** were the most potent inhibitors of EGFR while **7x** effectively inhibited the binding site of HER3. The binding energies of **4k, 4m,** and **7x** were −7.7, −6.3 and −5.7 kcal/mol, respectively. Collectively, **4k**, **4m** and **7x** showed favorable interactions with the most druggable binding sites of their respective proteins. Finally, in silico pre-clinical testing by SwissADME validated the non-toxic nature of compounds **4k**, **4m** and **7x** providing a promising treatment option for chemoresistant NSCLC.

## 1. Introduction

Lung cancer is one of the leading causes of death worldwide [1]. It has become the frequently diagnosed cancer with a high mortality rate than the other prevalent cancers (breast, colon, and pancreatic) [2]. Small cell lung carcinoma (SCLC) and non-small cell lung carcinoma (NSCLC) are the two types of lung cancer. NSCLC accounts for 85% of all lung cancer cases, while SCLC accounts for 15% [2,3]. NSCLC is the most common malignant transformation in the world. This type of lung cancer is further divided into adenocarcinoma, squamous-cell carcinoma, and large-cell carcinoma [2]. NSCLC is a heterogeneous class of tumor that is related to poor diagnosis. Even though there are many different therapeutic approaches available, it frequently occurs owing to excessive proliferation and poor prognosis [4]. Although air pollution and radon exposure also play a part in the development of this disease but smoking continues to be the predominant risk factor. The majority of patients acquire an advanced-stage diagnosis which gives them a very dismal prognosis due to inadequate screening services and the late onset of symptoms [2,4]. A shift towards an earlier diagnosis of NSCLC may result from advancements in diagnostic techniques (such as screening with low-dose computed tomography scans) and increased public knowledge of the abnormalities [5,6,7,8,9]. It can be diagnosed using a number of methods such as X-ray, CT scan, and PET imaging, as well as histological analysis of tumor specimens. To choose the best treatment plan, the cancer must be accurately staged. The management strategies include surgery, chemotherapy, radiotherapy, adjuvant therapy, immunotherapy, and targeted treatments (such as anti-angiogenic inhibitors) [4].

Adenocarcinoma and squamous cell carcinoma is considered the major subtypes of NSCLC, and it accounts for almost 87% of all cases. It has been challenging to treat the disease for over three years, and the survival rate for lung cancer patients is only 18% [10]. Although multiple approaches like surgery, chemotherapy, immunotherapy, and other therapies are recommended for NSCLC, it is quite challenging to treat NSCLC [11]. Platinum is recommended to patients in the early stages of chemotherapy, but it is not used as the standard first-line regimen [12]. In addition, pemetrexed is extensively used to treat NSCLC and is considered an antifolate. It acts as an antagonist against folates, where folates donate one carbon unit in the biosynthesis of thymine, purine, and DNA. The disturbance in DNA causes an interruption in DNA synthesis, which causes the failure in the growth of tumor cells [13]. Pemetrexed has three steps that cause an inhibitory effect in which three uptake receptors perform their function effectively, such as proton-coupled folate receptor, folate receptor-α (FR-α), and reduced folate receptor (RFC) [10]. These receptors are responsible for modifying the pharmacologically active form of the agent through the action of folylpolyglutamate synthetase (FPGS) in the cytosol and mitochondria. It causes the agent to remain in the cell and obtain great affinity than the parent form [10]. It has also shown efficacy as the first-line therapy with platinum and a single agent for second-line treatment. Despite the high efficacy, it has been expected that NSCLC will develop chemoresistance that will affect its clinical efficacy after long-term use [10,14].

There are many different types of lung cancer, from very slow-growing and surgically treatable SCLCs to extremely aggressive and substantially metastatic NSCLCs. Identifying these variations in lung cancer is greatly aided by the discovery of driver oncogene mutations of tumors. These inherited mutations cause constitutive signaling and promote oncogenic transformation [4]. NSCLC is due to the mutation in human epidermal growth factor receptor 3 (HER3) and epidermal growth factor receptor (EGFR). EGFR, a transmembrane receptor, is a member of the HER family of receptor tyrosine kinases [15]. The EGFR primarily exists in a monomeric form that is auto-inhibited, but when a ligand binds to it, it adopts a conformation that is ready to form either homodimers or heterodimers with HER2 or other receptors [16]. This ligand-activated EGFR then effectively stimulate multiple intracellular signaling pathways, resulting in enhanced robustness [17]. Mutant forms of EGFR that possess oncogenic properties imitate the ligand-induced activation of the wild-type counterpart. However, despite the enzymatic activity and transforming ability exhibited by the mutated EGFRs in tumors, their tyrosine phosphorylation status is notably lower when compared to the ligand-activated wild-type receptors [18]. Evidently, this seemingly modest yet enduring activity produces intracellular signals that deviate from the conventional biochemical mechanisms leading to tumorigenesis [19]. On the other hand, HER3 is the major cause of chemoresistance due to its role in PI3K/AKT signaling pathway. In addition, HER3 preferably binds with the EGFR as a heterodimeric partner [20]. Heregulin is the sole ligand known to bind HER3; other ligands for EGFR include EGF, TGF, and EPG. The binding of heregulin induces the heterodimerization of HER3 and HER2, resulting in the formation of the most potent ErbB receptor complex involved in ontogenesis. Co-expression of HER3 and HER2, but not HER3 and EGFR, acts synergistically to achieve this effect. Additionally, the inhibition of HER2-HER3 dimerization, which is thought to be a key step in the promotion of carcinogenesis, can diminish the proliferation and migration of transformed cells [21]. The high mutation rate in NSCLC, specifically in Asians, in the amino acid sequence of EGFR, is one of the causes of treatment failure. In the active form, the cell-surface tyrosine kinase receptor EGFR can open up pathways involved in cell growth and proliferation. Through persistent stimulation, EGFR mutations in malignancies cause unchecked cell division [2,22]. Exons 18–21, which encode a part of the EGFR kinase domain, are the sites of frequent EGFR mutations that offer vulnerability to EGFR tyrosine kinase inhibitors. Exon 19 deletions and the L858R point mutation on exon 21 make up about 90% of these alterations [2,23,24].

**Figure 1:**
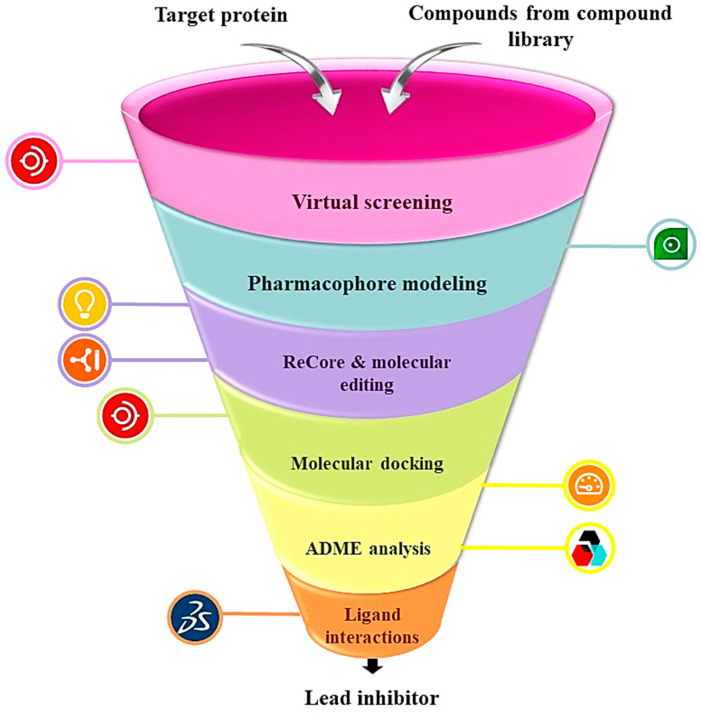
The schematic representation of methodology employed to obtain lead inhibitor by computational drug designing using BioSolveIT suit. The symbols with each step are indicating softwares used in this research.

Chemoresistance is brought on by the rapid incidence of mutations, which also reduces the effectiveness of chemotherapy. Platinum compounds-mediated formation of DNA adducts creates alternations in DNA structure. It causes the activation of the DNA repair system, which protects the cells from apoptosis. The upregulation in the DNA repair pathway creates resistance to platinum-based chemotherapeutic agents. It has been found that NSCLC showed less response to cisplatin due to an increase in DNA repair capacity. The overexpression of genes linked to the nucleotide excision repair system (NER) causes cisplatin resistance [25]. Furthermore, the platinum-mediated DNA adduct formation triggers the DNA damage response, which causes cell arrest and apoptosis. The cisplatin regulation induces the upregulation of ATM and phosphorylation in the downstream effectors, such as CHK2 and p53 [26]. The overexpression of anti-apoptotic proteins bcl-2 and bcl-xl induces cell-cycle arrest at the G2/M phase through the deregulation of cell-cycle linked proteins cdc2 and cdc25C. It has been found to cause resistance to platinum drugs [26]. As a result, the recovery rate of patients with NSCLC gets reduced. It is mandatory to target the novel druggable proteins that are not prone to genetic alterations and thus can help subdue chemoresistance. Therefore, HER3 and EGFR are simultaneously targeted in this research to treat NSCLC and overcome chemoresistance using BioSolveIT suite as shown in Figure 1.

## 2. Results and Discussion

### 2.1. Virtual Screening 

**Figure 2:**
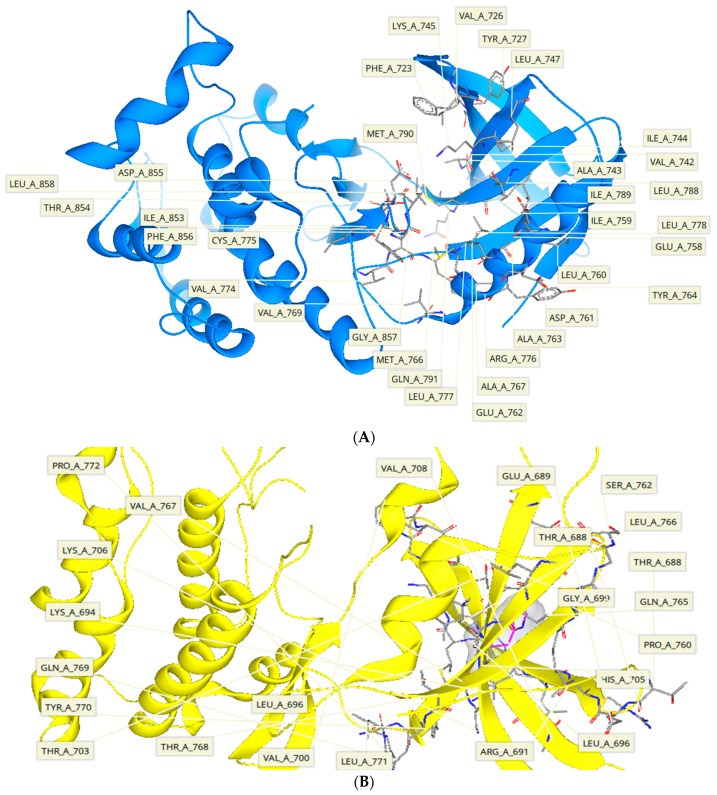
(**A**) The visualization of binding site residues of EGFR (33 amino acid residues) which interact with the inhibitors through various intermolecular interactions. These amino acid residues are the part of active site backbone and include Phe723, Val726, Tyr727, Val742, Ala743, Ile744, Lys745, Leu747, Glu758, Ile759, Leu760, Asp761, Glu762, Ala763, Tyr764, Met766, Ala767, Val769, Val774, Cys775, Arg776, Leu777, Leu778, Leu788, Ile789, Met790, Gln791, Ile853, Thr854, Asp855, Phe856, Gly857, and Leu858. (**B**) The visualization of binding site residues of HER3 (23 amino acid residues). All the residues interact with inhibitors by different intermolecular interactions. These amino acid residues are component of active site backbone and include Pro772, Val767, Lys706, Lys694, Gln769, Tyr770, Thr703, Thr768, Leu696, Val700, Leu771, Arg691, Leu696, Ser762, Glu689, Thr688, Gly699, His705, Leu696, Ser762, Leu766, Thr688, Gln765, and Pro760.

After generating the analog libraries and selection of target proteins, the compounds were virtually screened using FlexX functionality of SeeSAR. Before molecular docking analysis, the proteins were individually uploaded in the protein mode of SeeSAR and binding sites were automatically defined. For EGFR, the binding site consisted of 33 residues, and for HER3, 23 residues make up the active site as shown in Figure 2. Afterwards, analog libraries for both the target proteins were uploaded in the docking mode of SeeSAR and reference docking was performed to generate 10 poses for each compound in the library. A total of 5730 and 3972 poses were generated for EGFR and HER3, respectively. 

### 2.2. Pharmacophore Modeling 

**Figure 3:**
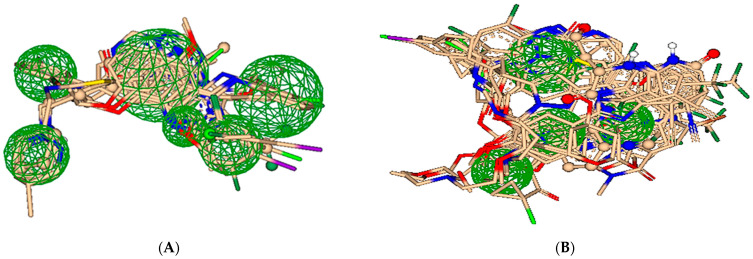
Ligand-based pharmacophore design for screening the best-docked poses of inhibitors of target proteins. (**A**) Pharmacophore of EGFR is formed by 14 ligands that contain aromatic rings, nitrogen and oxygen atoms interacting with the amino acid residues of EGFR active site. (**B**) Pharmacophore of HER3 is composed of 23 ligands that perfectly align and interacts with the active site residues via similar groups. These include aromatic rings, any oxygen and any nitrogen.

All the docked poses, which were obtained after blind docking for both proteins, were screened to obtain the best suitable poses for pharmacophore modeling. The screening criteria for the best suitable poses was based on estimated affinities, torsion angles and clashes. Consequently, 14 well-aligned poses were chosen to generate the ligand-based pharmacophore for EGFR while 23 best-posed compounds were selected for the construction of ligand-based pharmacophore for HER3 as depicted in Figure 3.

### 2.3. ReCore and Molecular Editing

**Figure 4:**
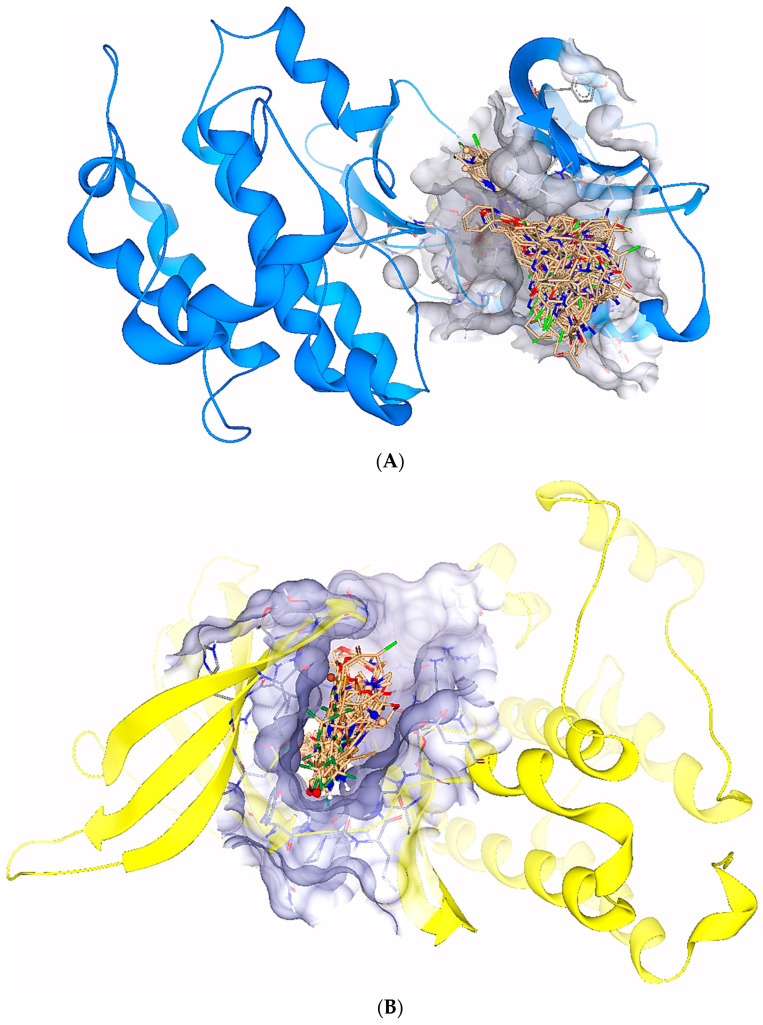
(**A**) The representation of 98 hits docked in the binding site of the EGFR. (**B**) The representation of 53 hits docked inside the binding site of HER3.

One of the most critical steps in drug discovery process is the identification of hits which are the molecules having a high probability of interaction with the biological target [27,28]. In current research, the hits were screened by ligand-based pharmacophore and some specific parameters such as estimated affinities, torsions, clashes, and optibrium properties. Therefore, 98 and 33 hits were selected that optimally inhibit EGFR and HER3, respectively, as shown in Figure 4. 

The optimization of hits is also critical for discovering a lead inhibitor. Therefore, some of the best compounds were optimized by using the ReCore functionality and molecular editor mode of SeeSAR. 117 novel compounds for EGFR and 123 novel compounds for HER3 were generated by editing the structures of best-selected hits. These were docked by standard docking through FlexX functionality of SeeSAR version 12.1.0 to generate 1170 and 1570 poses, respectively. These poses were screened to obtain 73 (EGFR) and 66 (HER3) compounds for further analysis. Additionally, some of the screened compounds were subsequently analyzed using the inspirator mode of SeeSAR in order to identify new scaffolds. A particular fragment of each compound was chosen and substituted with suitable fragments produced from the fragment library utilizing ReCore. Resultantly, 110 new compounds were obtained and docked against EGFR that generated 1068 poses. Similarly, 120 new compounds were docked against HER3 that generated 1098 poses. These poses were screened on the basis of estimated affinities, torsions, and clashes resulting in the selection of 49 (Table S2) and 43 compounds (Table S4), respectively. 

### 2.4. Pre-Clinical Testing

SwissADME, an online software for pharmacokinetic analysis, was used for the selection of safe and druggable inhibitors for both target proteins. For a druggable inhibitor, certain criteria should be fulfilled; TPSA (topological polar surface area) should be greater than 20 Å^2^ and less than 130 Å^2^ [29], rotatable bonds less than 9, and molecular weight should range from 150 g/mol to 500 g/mol [30]. In order to prevent skin permeation, the log Kp value should be negative. The negative value of log Kp is directly related to a decrease in skin permeability [30]. The synthetic accessibility value, which ranges from 1 to 10, should be lower to ensure that the compound is druggable. This value predicts the ease with which a compound can be synthesized in vitro, with higher values indicating greater difficulty [30,31].

In case of EGFR, the pharmacokinetic analysis of 122 novel compounds (73 compounds from molecular editing and 49 compounds from inspirator) was carried out to obtain a lead inhibitor, which follows all the druggable criteria. According to these criteria, **4k** and **4m** were found to follow all the druggable criteria. 

In HER3, the pharmacokinetic analysis of 109 novel compounds (66 from molecular editing and 43 compounds from inspirator) was used to obtain the lead inhibitor that follow all the druggable criteria. In this case, **7x** was identified as the lead molecule.

Compound **4k** namely 1-((7-((2*R*,4*S*)-4-hydroxytetrahydrofuran-2-yl)-6-methyl-7*H*-pyrrolo[2,3-*d*]pyrimidin-4-yl)methyl)azepan-1-ium has a molecular weight of 331.43 g/mol with 24 heavy atoms, 9 aromatic heavy atoms, 3 rotatable bonds, 4 hydrogen bond acceptors and 2 hydrogen bond donors. In addition, the TPSA, molar refractivity, consensus log P, and log Kp values are 64.61 Å^2^, 97.78 m^3^mol^−1^, 0.88, and −7.24 cm/s, respectively. This compound is non-toxic as it does not inhibit any cytochrome P450 (CYP) and has high gastrointestinal absorption. Moreover, **4k** follows all the druggable rules, exhibits no Brenk or PAINS alerts (Table 1).

Compound **4m** namely (1*S*,3*R*)-1-(cyclopentylmethyl)-3-((4-methoxy-1*H*-pyrazol-1-yl)methyl)azetidin-1-ium follows all the druggable rules and has a molecular weight of 250.36 g/mol. In addition, it is safe with no toxicity, and zero PAINS and Brenk alerts. Moreover, **4m** contains 18 heavy atoms, 5 aromatic heavy atoms, 5 rotatable bonds, 2 hydrogen bond acceptors and 1 hydrogen bond donors. The TPSA, molar refractivity, consensus log P, and log Kp values are 31.49 Å^2^, 76.79 m^3^mol^−1^, 1.02, and −6.39 cm/s, respectively. The solubility and gastrointestinal absorption are high with no permeation to blood-brain barrier as shown in Table 1.

Compound **7x** namely (S)-2-((5-bromo-1H-pyrazol-3-yl)amino)-N-ethylpropanamide follows all the druggable rules showing molecular weight of 261.12 g/mol, 14 heavy atoms, 5 aro-matic heavy atoms, 5 rotatable bonds, 2 number of H-bond acceptors and 3 donors. In addition, **7x** exhibits zero toxicity, PAINS and Brenk alerts while showing good solubility. It also follows Lipinski’s rule and shows lead-likeness properties (Table 1).

The binding energies of the best-selected inhibitors of EGFR and HER3 are represented in Table 2. 

### 2.5. Protein-Ligand Interactions

Discovery Studio plays a vital role in providing a visual representation of the interactions that occur between a ligand and the corresponding target protein at the molecular level. 

**Figure 5:**
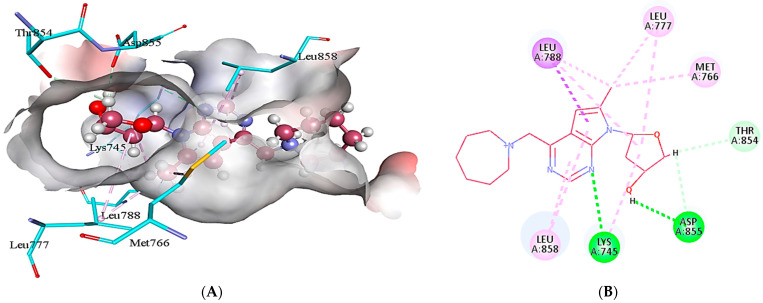
The illustration of intermolecular interactions between **4k** (dark pink color) and binding site residues of EGFR (light blue color), in both three-dimensional (**A**) and two-dimensional (**B**) formats. The different types of interactions are shown through dotted lines of various colors. The conventional hydrogen bonds are depicted with green dotted lines, while alkyl and π-alkyl interactions are shown with pink dotted lines. The light blue lines represent carbon hydrogen bonds, whereas, the purple dotted line depicts π-sigma interaction.

In case of EGFR, **4k** interacts with the binding site residues by forming conventional hydrogen bonds, carbon hydrogen bonds, alkyl, π-alkyl and π-sigma bond. A conventional hydrogen bond is formed by the interaction of amino group of Lys745 and the N5 of **4k** with a bond distance of 2.87 Å. The other conventional hydrogen bond is developed between the oxygen of Asp855 and H49 of **4k** (1.88 Å). The H50 of the **4k** interacts with the Thr854 and Asp855 via carbon hydrogen bond interactions. In addition, alkyl bond interactions are formed by Lys745, Met766, Leu777, and Leu788 with C12 and tetrahydrofuran ring of **4k**. Leu858 develops π-alkyl interactions with the 7*H*-pyrrolo[2,3-*d*]pyrimidine ring of **4k**, whereas, Leu788 forms a π-sigma bond with the pyrrole ring of **4k** as depicted in Figure 5.

**Figure 6:**
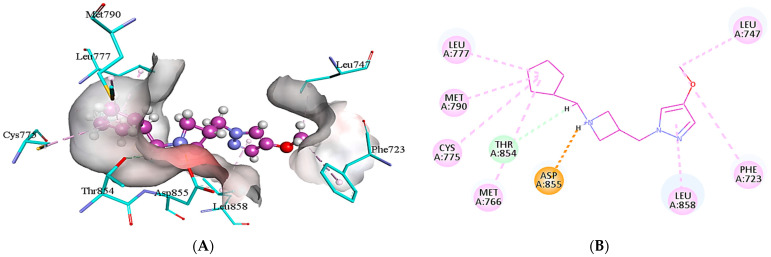
The illustration of intermolecular interactions between **4m** and EGFR binding site residues in both three-dimensional (**A**) and two-dimensional (**B**) form. Compound **4m** is depicted as a purple color, while the binding site residues are represented by light blue color. The different types of interactions are portrayed using dotted lines of various colors. The carbon hydrogen bond is depicted with light blue dotted lines, whereas, pink dotted lines are used to show alkyl and π-alkyl interactions. The salt bridge is elucidated by orange dotted lines.

Similarly, **4m** interacts with the active pocket residues of EGFR by developing salt bridge, carbon hydrogen bond, alkyl and π-alkyl interactions. Asp855 forms a salt bridge with the H26 of **4m** having a bond distance of 2.00 Å. The carbon hydrogen bond is formed by the interaction of H49 of **4m** with Thr854 of target protein (2.88 Å). In addition, the cyclopentyl group of **4m** develops alkyl interactions with the Met766, Cys775, Leu777, and Met790, which are present at a distance of 4.93, 5.28, 4.48 and 4.89 Å from the ligand, respectively. Moreover, two π-alkyl interactions are formed by C18 of **4m** with Phe723 (5.14 Å) and Leu747 (4.28 Å) of EGFR. Leu858 of the target protein also develops a π-alkyl interaction with the heterocyclic ring of **4m** as represented in Figure 6.

**Figure 7:**
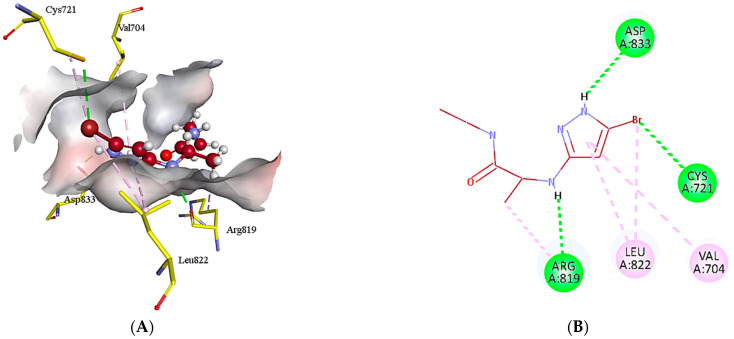
3D (**A**) and 2D (**B**) binding mode interactions of **7x** and HER3 binding site residues. The intermolecular interactions between the ligand and amino acid residues can be clearly visualized. Light green color shows the binding site residues, while red color represents compound **7x**. The different interactions have been shown in varying colors using dotted lines. The green color shows the hydrogen conventional bond while purple color illustrates π-alkyl bond.

In case of HER3, **7x** established various interactions with the amino acid residues such as conventional hydrogen bond, π-alkyl and alkyl (Figure 7). The conventional hydrogen bonding has been observed between the NH group of heterocyclic compound and Asp833(1.90 Å) and between Br and Crys721 (3.64 Å, 4.12 Å). In addition, the conventional hydrogen bonding has been found between NH group and Arg819 (4.57 Å, 1.91 Å). On the other hand, π-alkyl bonding has been identified between the heterocyclic ring and Val704 (5.31 Å) and Leu822 (3.64 Å).

The methyl group, hydroxyl group, pyrimidine and furan rings present in the **4k** are the major contributors to the interaction with the EGFR active site. The methyl group donates its electrons and stabilizes the ligand; likewisely, furan is quite stable and contribute to the overall stability of the ligand. The pyrazole, cyclopentane, and methoxy ring are the core groups in **4m**, which interact with the active site residues of EGFR. The presence of pyrazole is an indication of the biological activity of **4m** because a number of compounds having anti-cancer activities possess pyrazole ring. Both these inhibitors have comparable docking score of −7.7 kcal/mol (**4k**) and −6.3 kcal/mol (**4m**) with the SD−06 (−8.0 kcal/mol) and Amgen 16 (−7.7 kcal/mol) that are also EGFR inhibitors [32]. Moreover, **4k** and **4m** do not show any intermolecular interaction with the amino acids at 797 (serine), 844 (valine), 948 (arginine) in the active site responsible for ineffectiveness of EGFR inhibitors because these sites are more prone to mutations [33]. In addition, methyl group, pyrazole ring and bromine are crucial in the interaction of **7x** with the HER3 active pocket. The pyrazole ring is capable of forming conventional hydrogen bond with the amino acid residue. The presence of electronegative atom (bromine) substitution at the pyrazole ring also facilitated another hydrogen bond formation. Methyl group is highly stable and is involved in the hydrophobic interactions.

## 3. Materials and Methods

### 3.1. Selection of Target Protein

To perform the docking analysis, the proteins were selected from the Research Collaboratory for Structural Bioinformatics (RCSB) protein data bank (PDB) (https://www.rcsb.org/ accessed on 14 January 2022), a database established by Walter Hamilton for protein selection with three-dimensional structures and other information required for experimental studies. RCSB PDB is a data center in US that maintains stores and validates the experimentally determined 3D structures of proteins [34]. The proteins selected in this study were HER3 (4RIY) and EGFR (5ZWJ) and their selection was based on high enrichment value and resolution. The selected 3D structure of EGFR (PDB id: 5ZWJ) consisted of a chain A of 355 amino acid residues with a resolution of 2.90 Å [33], whereas, the 3D structure of HER3 (PDB id: 4RIY) contains two chains namely A and C which are cumulatively made up of 326 amino acid residues. The resolution of HER3 was 2.98 Å [35].

### 3.2. Construction of Compound Library

Several studies from literature and databases helped us in the identification of inhibitors of HER3 and EGFR proteins. The reported inhibitors of the targeted proteins were determined from the published literatures and their analogs were obtained from the chemical structures databases namely ZINC (https://zinc.docking.org/ accessed on 27 January 2022) [36], PubChem (https://pubchem.ncbi.nlm.nih.gov/ accessed on 18 February 2022) [37] and NPC browser (https://tripod.nih.gov/?p=182 accessed on 11 March 2022) [38]. Zinc online database was constructed by the Irwin and Shoichet Laboratories in the Department of Pharmaceutical Chemistry at the University of California, San Francisco (UCSF) [39]; whereas, PubChem is based in National Institute of Health (NIH) since 2004 [37]. In addition, using the BioSolveIT tool, InfiniSee version 4.3.0 (https://www.biosolveit.de/infiniSee accessed on 21 February 2022), 2-dimensional (2D) similarity search of the reported inhibitors was performed. BioSolveIT is a custom scientific software development company based in Augustin, Germany. One of its softwares, InfiniSee contains the in-built chemical space libraries that determine the compound libraries for scaffold hops and evaluates the molecules on the basis of chemistry and pharmacophore-based designing [40]. In this case, one specific inhibitor was uploaded in the software and different databases, eMolecules, Freedom space, REAL space, GalaXi, CHEMriya, Virtual space and CoLibri were marked to obtain the maximum analogs of the inhibitor [41].

To obtain the 2D structures of similar compounds for EGFR, gefitinib was used as a query in infiniSee, which is the selected reported inhibitor for EGFR. Similarly, the similar structures for HER3 were obtained by providing the 2D structure of lapatinib as an input to infiniSee. All the settings were kept are default. As a result, the analog library of EGFR was built containing 602 compounds (Table S1) while HER3 library consists of 301 compounds (Table S3).

### 3.3. Virtual Screening

In order to virtually screen the compound library, blind molecular docking was performed by using SeeSAR version 12.1.0 (www.biosolveit.de/SeeSAR accessed on 9 August 2022), a BioSolveIT tool that has several modes, such as protein mode, analyzer mode, binding site mode, molecular editor mode, inspirator mode, and docking mode [42]. All these modes were used to perform docking of a library of compounds and determine the ligand-protein binding based on affinities, torsion angles and other parameters. The PDB ID of each targeted protein was uploaded in the protein mode and its co-crystalline structure and chain were analyzed for the docking studies. The particular structure was shifted to the binding site mode and binding site was identified. After binding site selection, the ligands were uploaded in the docking mode and standard docking was performed. The results showed multiple poses of each ligand which were subsequently screened based on their affinities and torsion angles. In addition to torsion angles, clashes and other optibrium properties such as log P, log S, molecular weight, and blood brain barrier were also investigated for each ligand for further screening [43].

### 3.4. Pharmacophore Modeling

Ligand based pharmacophore modeling was done in the docking mode of SeeSAR version 12.1.0 after blind docking [44,45]. It represents the features for ligand interactions with biological target [46]. This mode was used for docking of ligand where standard docking was performed and different poses were generated. The poses showed the rotational conformation of various ligands that interacted with the active site of protein. The ligand conformations further exhibited maximum interactions between protein and ligand poses. The poses with maximum interactions were filtered for pharmacophore modelling.

### 3.5. ReCore and Molecular Editing

The inspirator mode of SeeSAR version 12.1.0 contains the built-in tool ‘ReCore’ that utilizes the fragment based lead discovery for fragment linking and core replacement. ReCore defines the bonds and interactions through matching them with new fragment. It has the other option of hits that uses the databases like PDB, CSD and ZINC for providing fragments [47]. ReCore was used to replace the unfavorable fragments with favorable fragments based on the parameter of torsions, optibrium properties and torsion angles. On the other hand, molecule editing is the other mode of SeeSAR version 12.1.0 and it was used for removing the torsions, interclashes of molecules.

### 3.6. Molecular Docking

FlexX functionality of SeeSAR version 12.1.0 was used for the standard molecular docking of novel compounds generated via ReCore and molecular editor mode of SeeSAR. By using the incremental construction algorithm, SeeSAR generates 10 best aligned poses for each of the novel compound. The best pose was selected on the basis of estimated affinity, torsion angles and optibrium properties [48,49].

### 3.7. ADME Analysis

The ADME analysis of ligands were conducted through SwissADME and analyzer mode of SeeSAR version 12.1.0. Initially, the selected molecules were transported to analyzer mode to determine their ADME properties in complex with protein target [50]. In other process, the online tool SwissADME, formed by Molecular Modeling Group of Swiss Institute of Bioinformatics, Switzerland, was used to apprehend safety and efficiency of ligands as the drug candidates. The SMILES was exported to SwissADME and results were interpreted in tabular and graphical form [30]. This further predicted the protein binding, CYP450 inhibition, blood-brain barrier permeability, solubility, lipophilicity, lead-likeness and drug-likeness [51].

### 3.8. Ligand Interactions

After performing the pharmacokinetics, ligand interactions of potent inhibitors were evaluated by BIOVIA discovery studio 2021 molecular visualization software made by Dassault system, a France based software company [52]. It helps in showing the 2D and 3D structures of complexes. The intermolecular interactions, such as π-alkyl bonds, hydrogen bonds, and unfavorable interactions were indicated by dotted lines upon visualization in 2D and 3D formats [53,54].

## 4. Conclusions

Non-small cell lung cancer (NSCLC) is a lethal disease and a major health issue worldwide due to its aggressive nature and high mutation rate, particularly in the epidermal growth factor receptor (EGFR). The identification of EGFR and HER3 as potential therapeutic targets for NSCLC has opened up new avenues for its treatment. This study employed a comprehensive computational approach to screen compound libraries for potent inhibitors of EGFR and HER3. The results identified **4k**, **4m**, and **7x** as the most effective inhibitors of EGFR and HER3. These inhibitors demonstrated favorable interactions with the druggable binding sites of their respective proteins and exhibited non-toxic properties in preclinical testing. In addition, these inhibitors do not cross the blood-brain barrier and do not permeate the skin. Therefore, the identified inhibitors present a promising treatment option for NSCLC with EGFR mutations, paving the way for further development and potential clinical applications.

## Figures and Tables

**Table 1:** Pharmacokinetic analysis of potent inhibitors (**4k** and **4m**) of EGFR and (**7x**) of HER3.

Attributes	4k	4m	7x
Structure	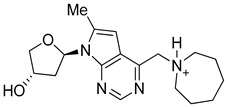	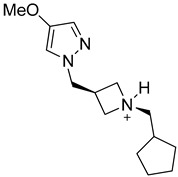	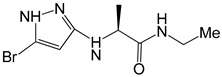
Formula	C_18_H_27_N_4_O_2_^+^	C_14_H_24_N_3_O^+^	C_8_H_13_BrN_4_O
Molecular weight (g/mol)	331.43	250.36	261.12
Number of heavy atoms	24	18	14
Number of aromatic heavy atoms	9	5	5
Fraction C(sp^3^)	0.67	0.79	0.50
Number of rotatable bonds	3	5	5
Number of H-bond acceptors	4	2	2
Number of H-bond donors	2	1	3
Molar refractivity (m^3^mol^−1^)	97.78	76.79	57.82
TPSA (Å^2^)	64.61	31.49	69.81
Consensus Log *P*_o/w_	0.88	1.02	1.18
Class	Soluble	Soluble	Soluble
GI absorption	High	High	High
Blood-brain barrier	No	No	No
P-gp substrate	Yes	Yes	No
CYP1A2 inhibitor	No	No	No
CYP2C19 inhibitor	No	No	No
CYP2C9 inhibitor	No	No	No
CYP2D6 inhibitor	No	No	No
CYP3A4 inhibitor	No	No	No
Log Kp (cm/s)	−7.24	−6.39	−6.71
Lipinski	Yes; 0 violation	Yes; 0 violation	Yes; 0 violation
Ghose	Yes	Yes	Yes
Veber	Yes	Yes	Yes
Egan	Yes	Yes	Yes
Muegge	Yes	Yes	Yes
Bioavailability score	0.55	0.55	0.55
PAINS	0 alert	0 alert	0 alert
Brenk	0 alert	0 alert	0 alert
Leadlikeness	Yes	Yes	Yes
Synthetic accessibility	3.88	3.39	2.77

**Table 2:** Binding energies of best inhibitors having optimum pharmacokinetic properties.

Best Selected Inhibitors	Binding Energies ((kcal/mol)
**4k**	−7.7
**4m**	−6.3
**7x**	−5.7

## Data Availability

The data presented in this study are available in supplementary material.

## Supplementary Materials

The following supporting information can be downloaded at: https://www.mdpi.com/article/10.3390/molecules28124850/s1, Table S1: EFGR reported inhibitors; Table S2: EGFR inhibiting compounds generated from molecular editor and inspirator mode of SeeSAR via adding and replacing atoms and group of atoms. Table S3: HER3 reported inhibitors; Table S4: HER3 inhibiting compounds generated from molecular editor and inspirator mode of SeeSAR via adding and replacing atoms and group of atoms.
