# Supplementary material for: Identification of Potent Inhibitors Targeting EGFR and HER3 for Effective Treatment of Chemoresistance in Non-Small Cell Lung Cancer
Source: Molecules. 2023 Jun 19;28(12):4850. doi: 10.3390/molecules28124850 (PMC10304665; doi:10.3390/molecules28124850)
Supplement: Supplementary file 1 [file molecules-28-04850-s001.zip › molecules-2420447-supplementary.pdf]

Identification of Potent Inhibitors Targeting EGFR and HER3 for Effective Treatment of Chemoresistance in Non-Small Cell Lung Cancer

Ayed A. Dera [1], Sumera Zaib [2,*], Areeba [2], Nadia Hussain [3,4], Nehal Rana [2], Hira Javed [2] and Imtiaz Khan [5,*]

[1]Department of Clinical Laboratory Sciences, College of Applied Medical Sciences, King Khalid University, Abha, Saudi Arabia

[2]Department of Basic and Applied Chemistry, Faculty of Science and Technology, University of Central Punjab, Lahore 54590, Pakistan

[3]Department of Pharmaceutical Sciences, College of Pharmacy, Al Ain University, Al Ain P.O. Box 64141, United Arab Emirates

[4]AAU Health and Biomedical Research Center, Al Ain University, Abu Dhabi P.O. Box 144534, United Arab Emirates

[5]Department of Chemistry and Manchester Institute of Biotechnology, The University of Manchester, 131 Princess Street, Manchester M1 7DN, United Kingdom

*Correspondence: sumera.zaib@ucp.edu.pk (S.Z.); kimtiaz@hotmail.co.uk (I.K.)

Table S1: EFGR reported inhibitors.

No.	Analog Structures	No.	Analog Structures
1	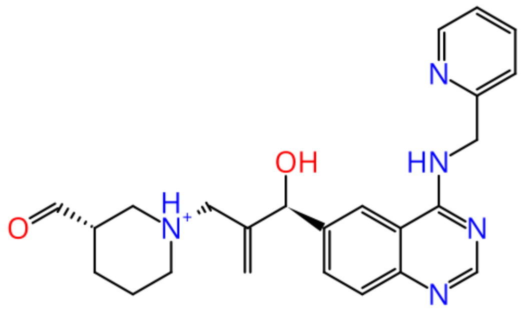	2	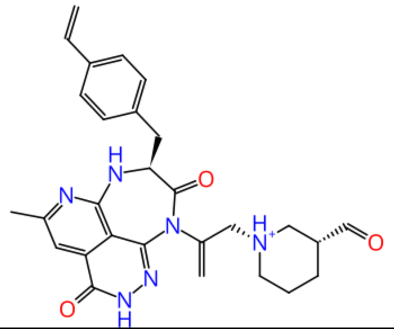
3	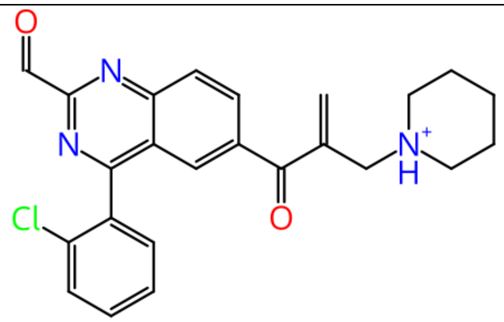	4	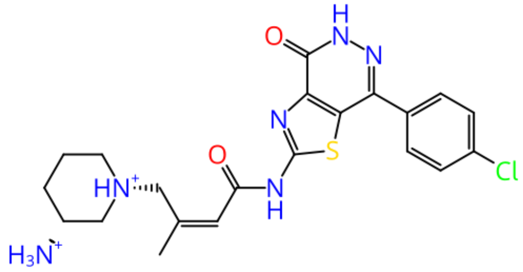
5	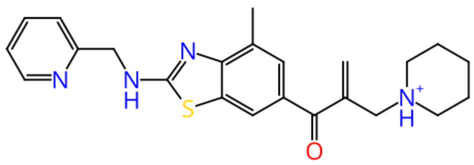	6	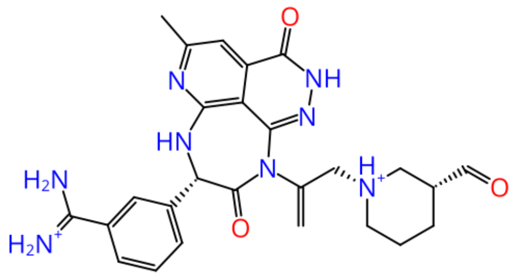
7	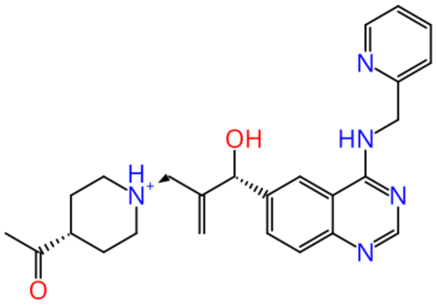	8	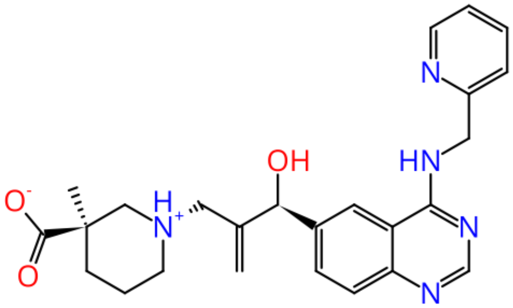
9	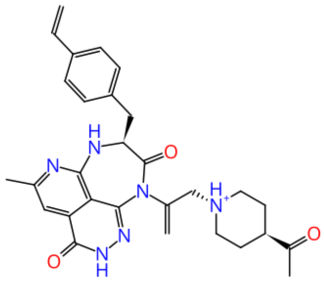	10	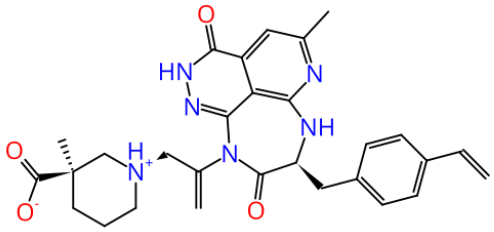

11	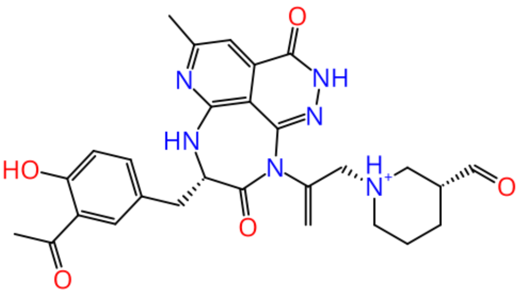	12	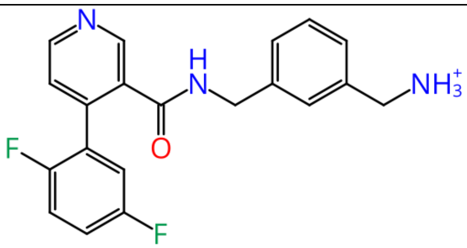
13	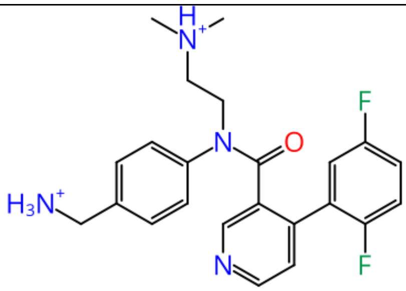	14	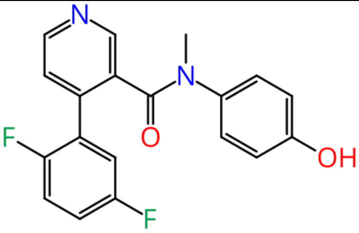
15	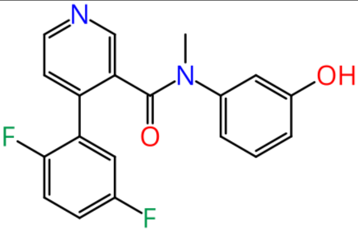	16	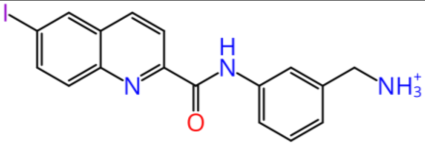
17	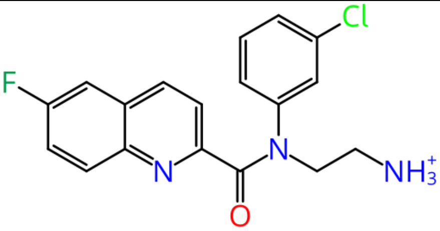	18	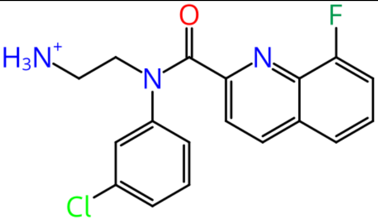
19	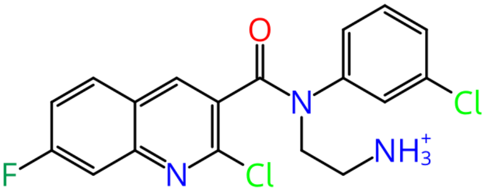	20	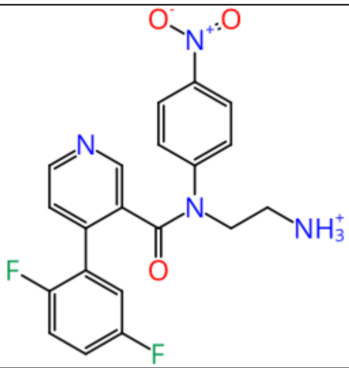
21	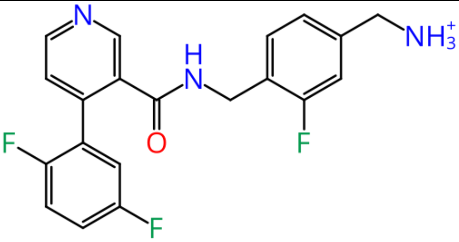	22	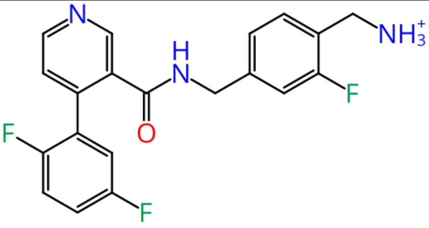

23	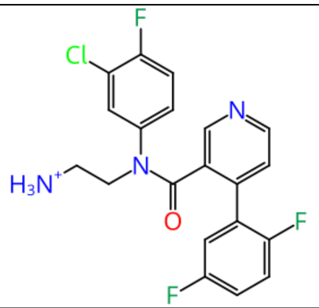	24	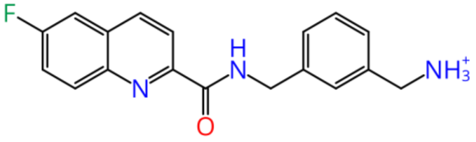
25	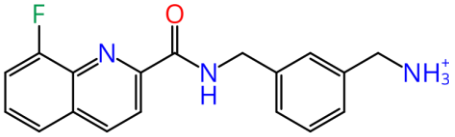	26	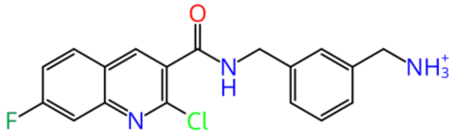
27	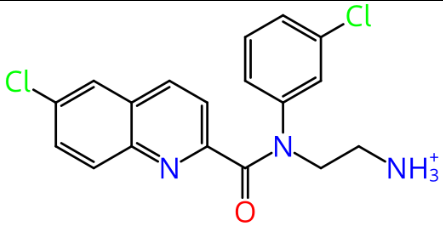	28	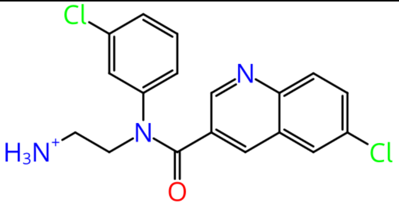
29	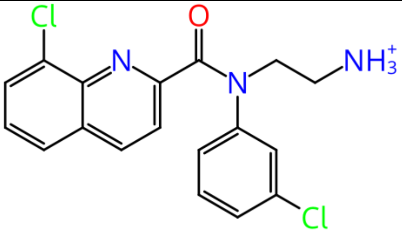	30	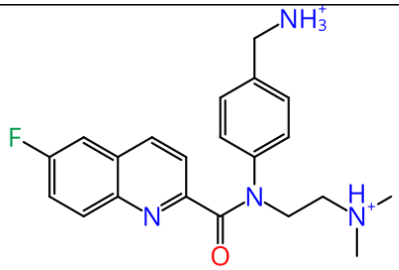
31	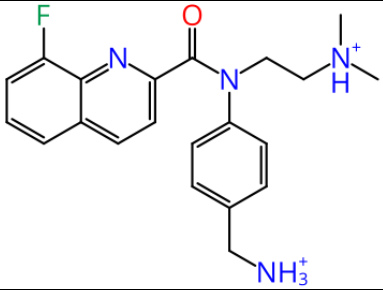	32	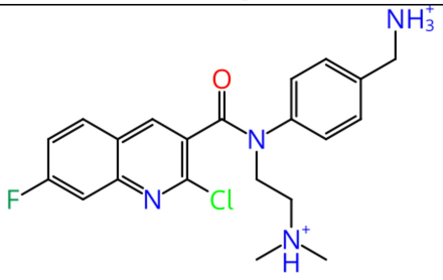
33	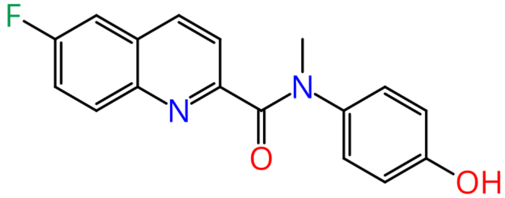	34	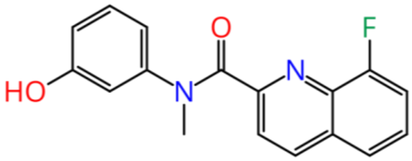
35	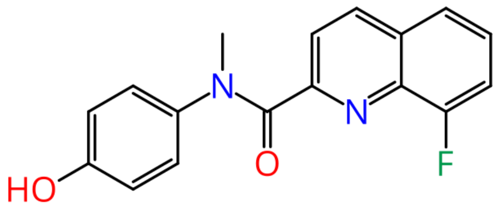	36	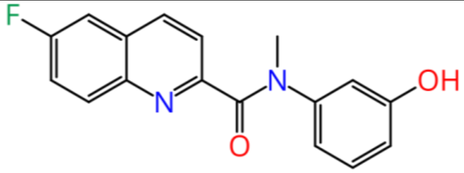
37	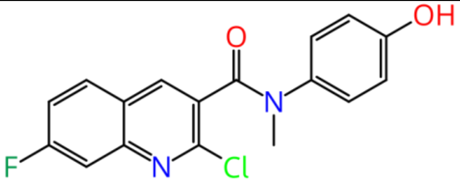	38	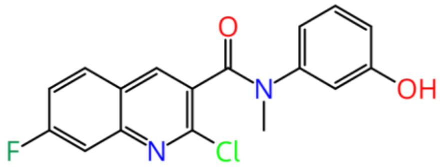

39	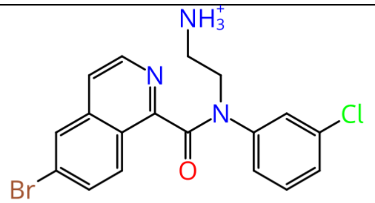	40	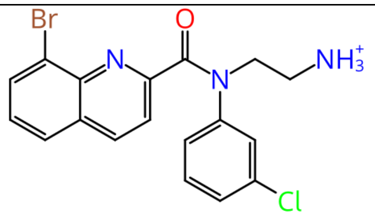
41	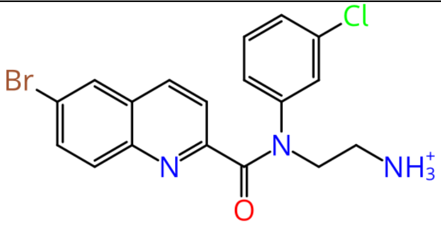	42	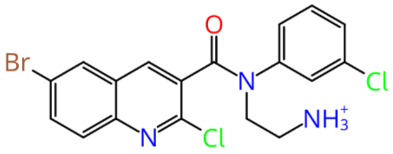
43	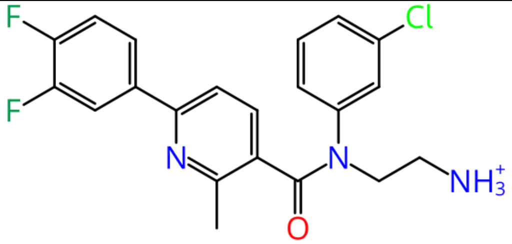	44	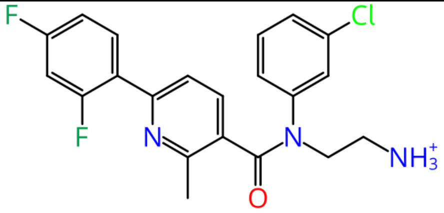
45	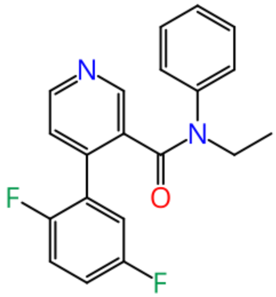	46	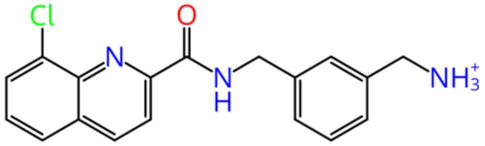
47	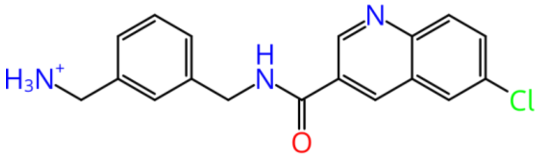	48	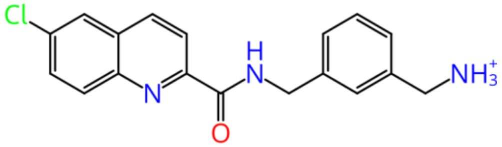
49	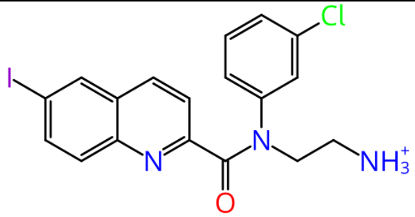	50	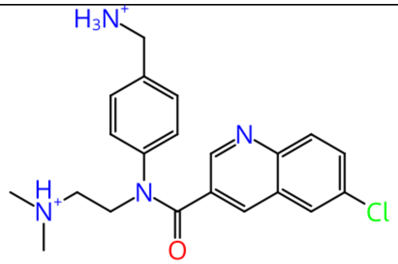
51	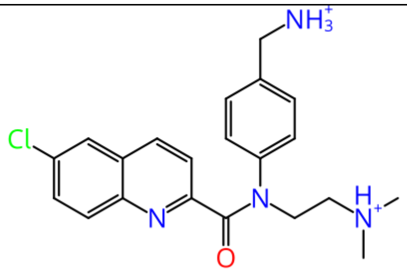	52	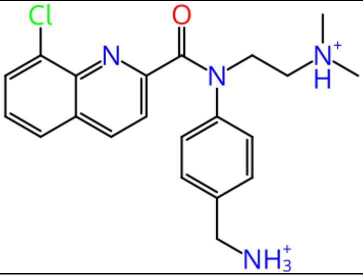
53	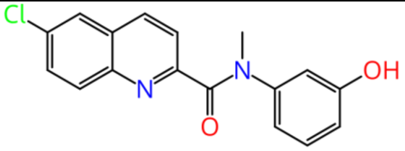	54	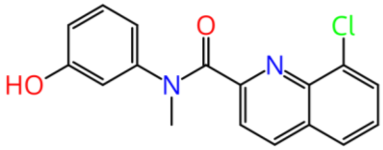

55	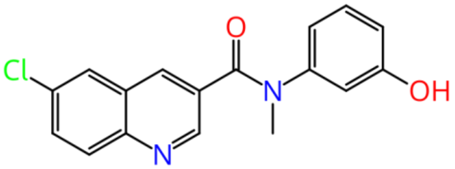	56	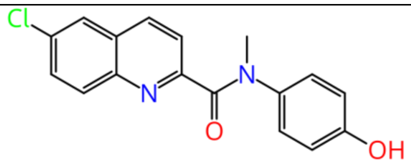
57	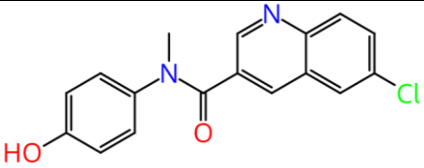	58	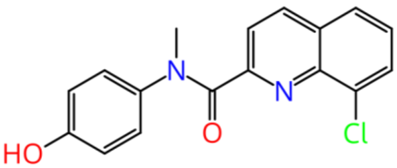
59	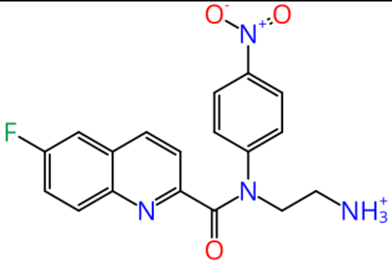	60	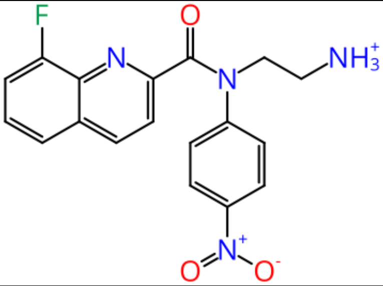
61	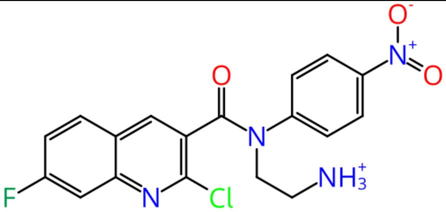	62	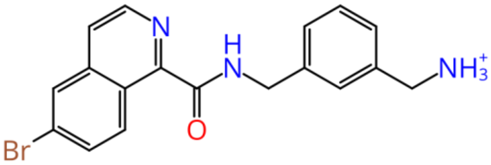
63	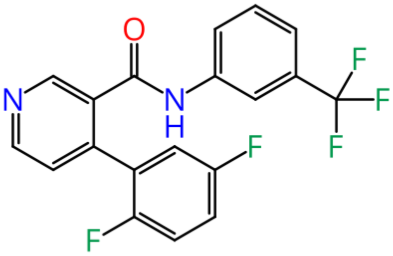	64	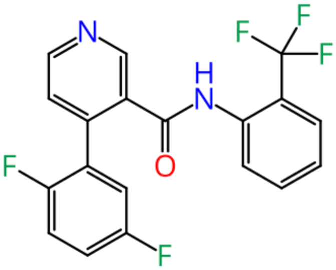
65	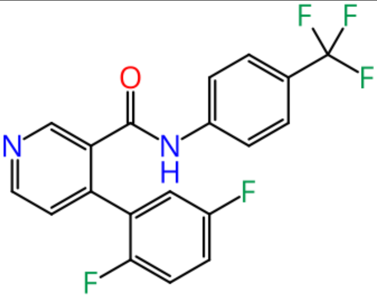	66	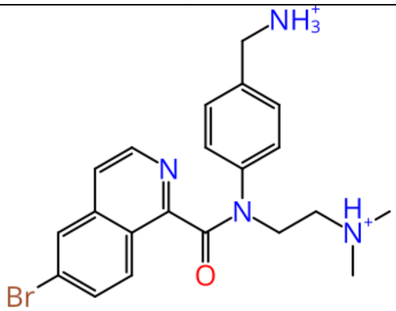
67	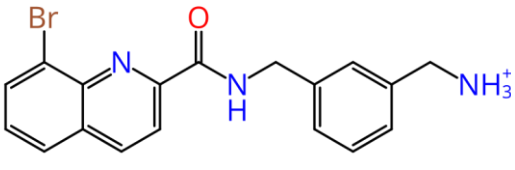	68	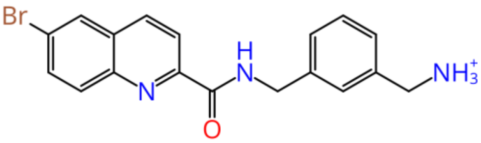

69	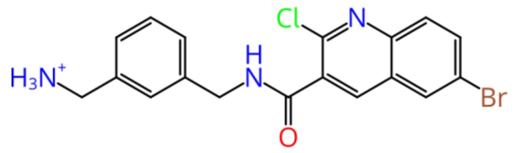	70	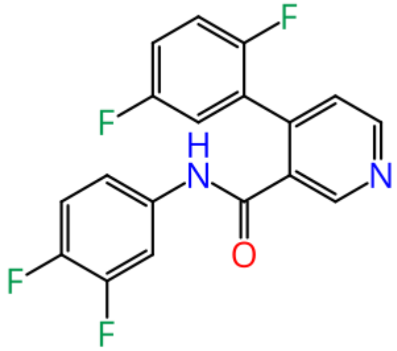
71	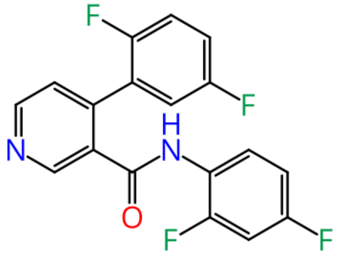	72	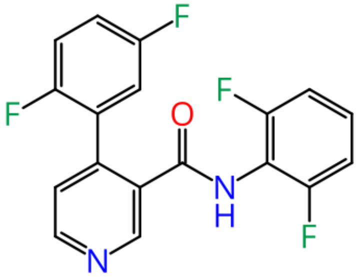
73	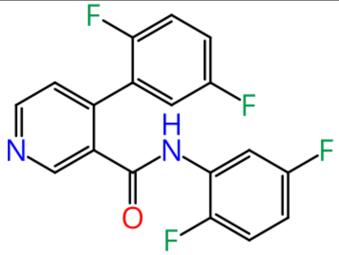	74	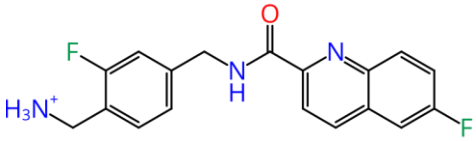
75	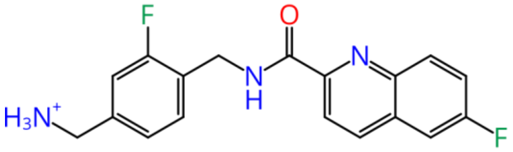	76	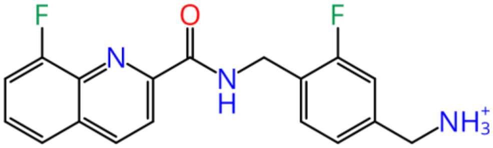
77	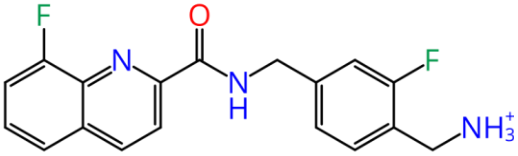	78	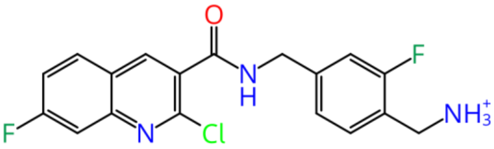
79	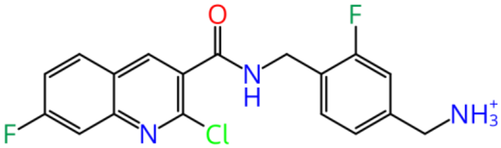	80	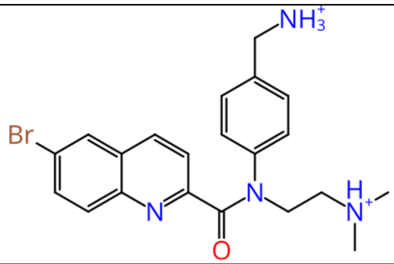
81	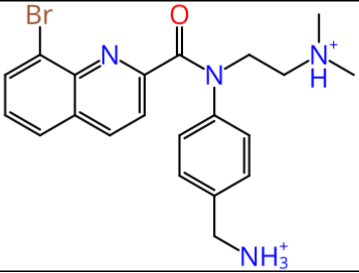	82	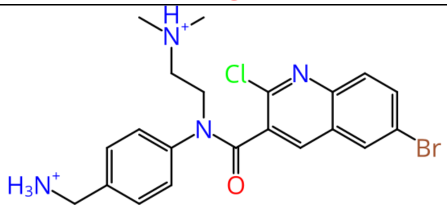

83	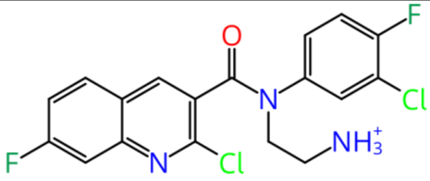	84	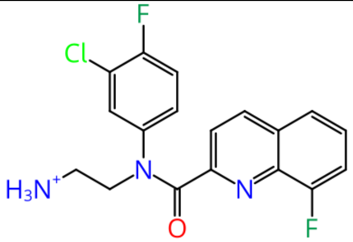
85	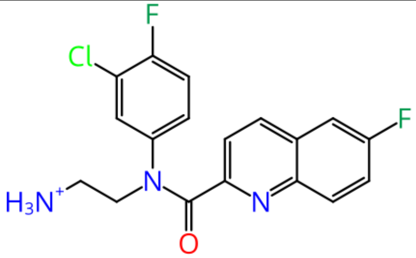	86	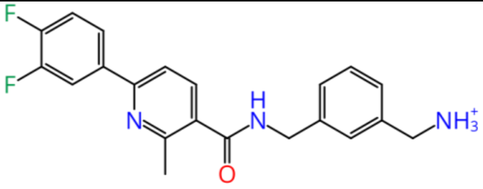
87	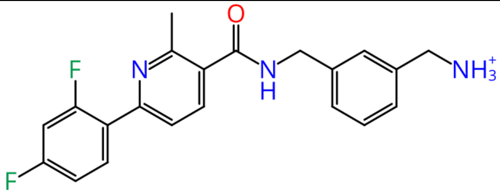	88	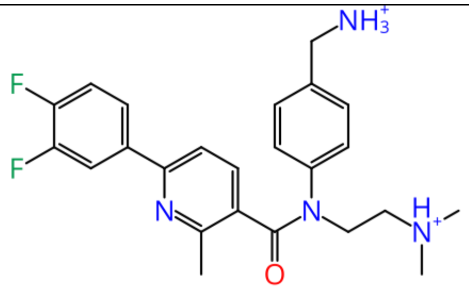
89	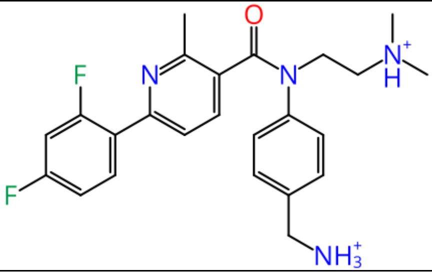	90	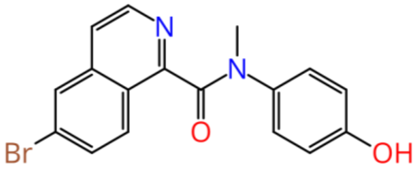
91	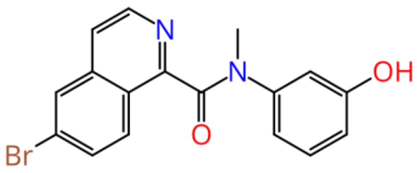	92	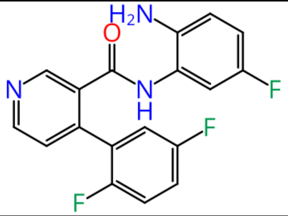
93	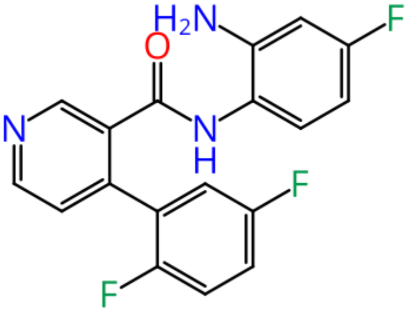	94	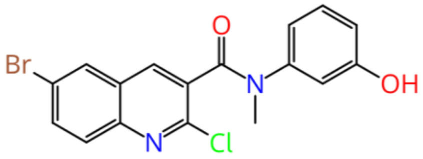
95	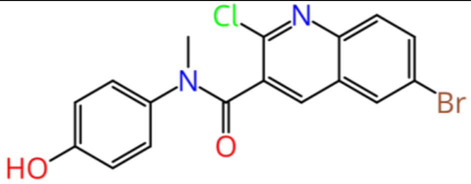	96	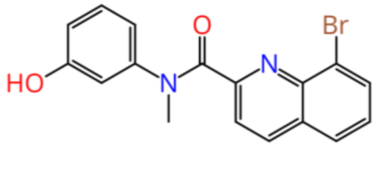

97	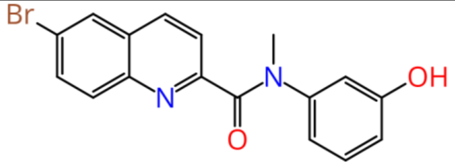	98	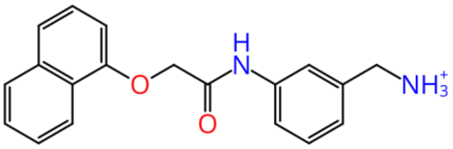
99	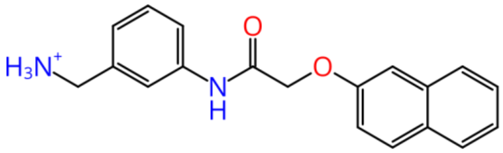	100	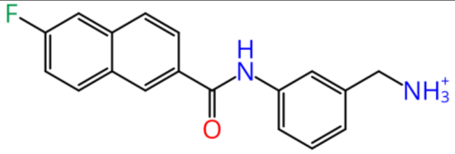
101	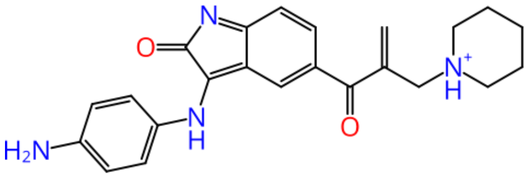	102	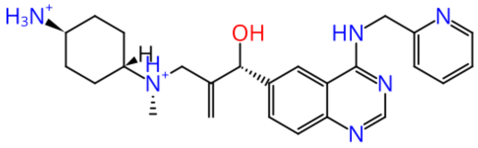
103	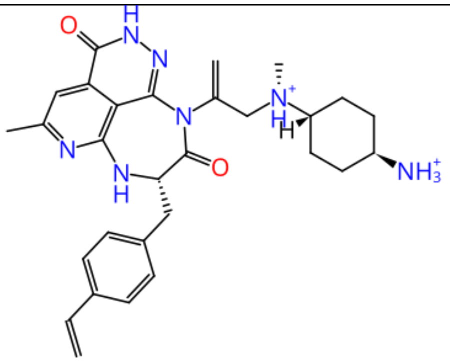	104	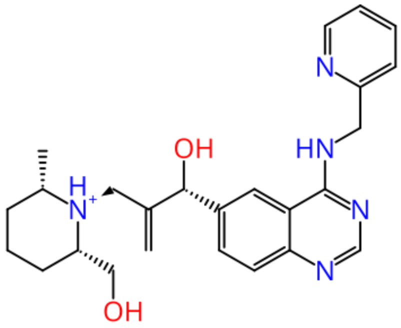
105	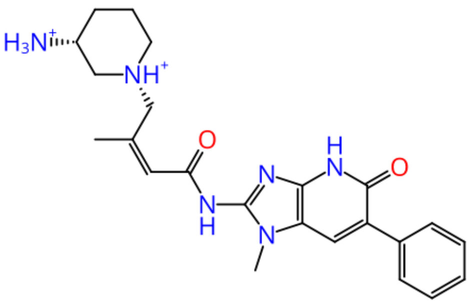	106	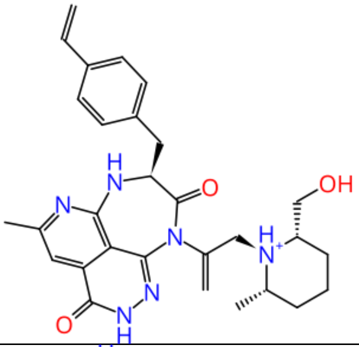
107	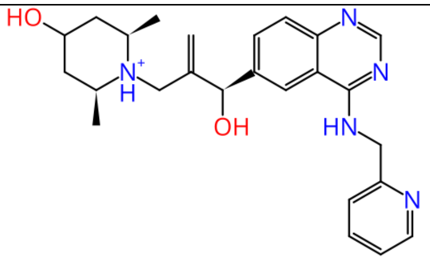	108	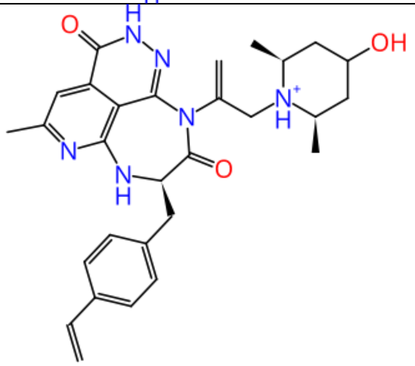

109	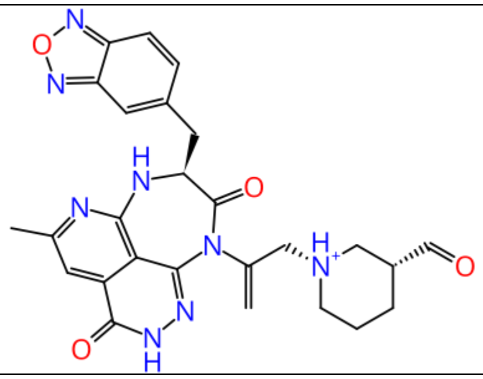	110	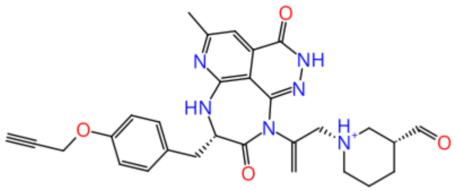
111	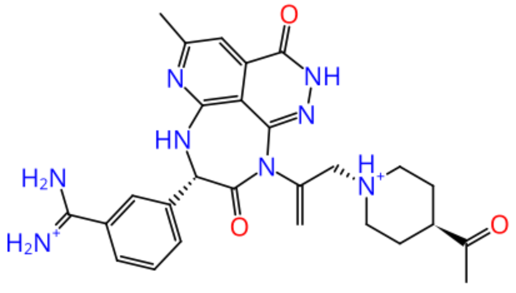	112	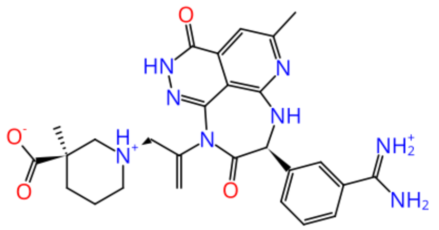
113	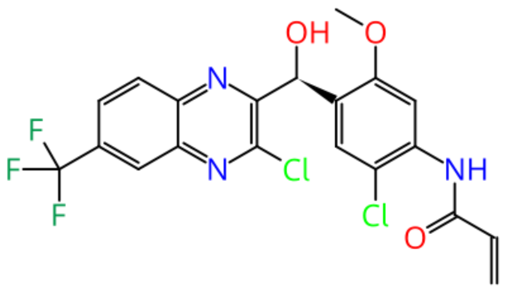	114	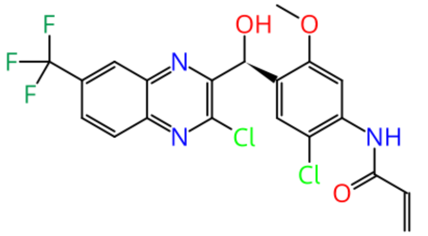
115	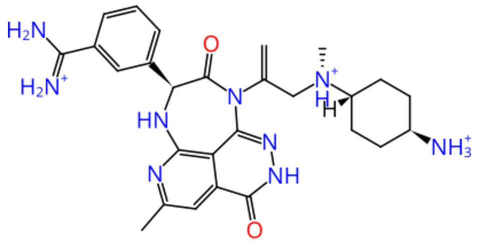	116	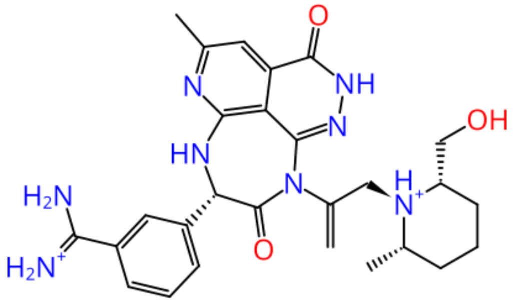
117	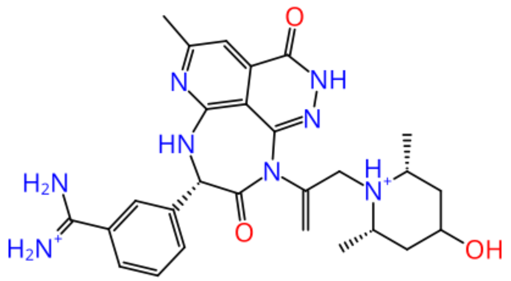	118	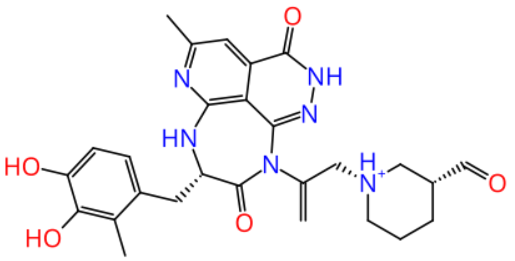

119	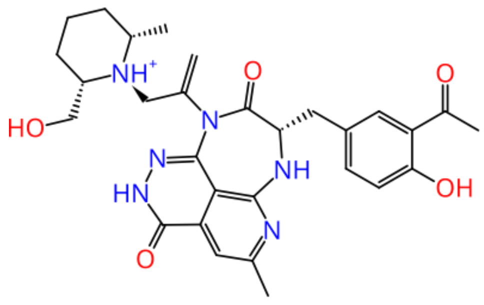	120	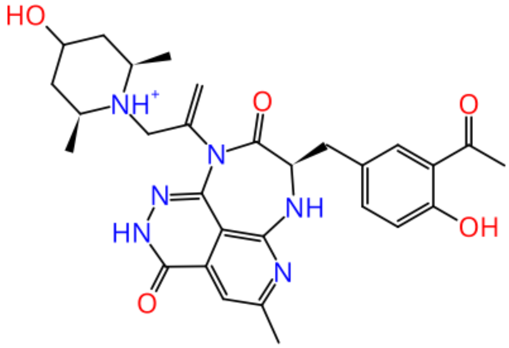
121	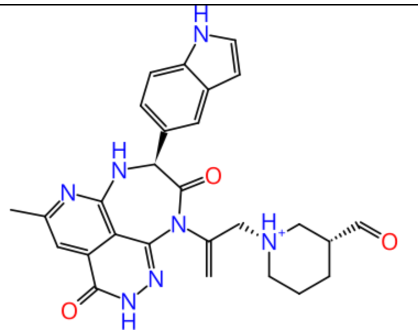	122	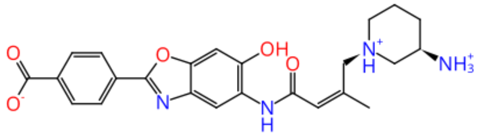
123	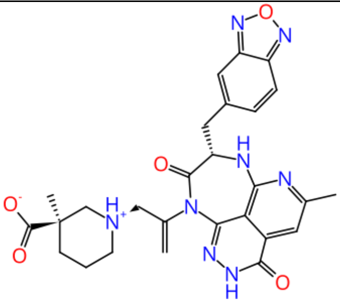	124	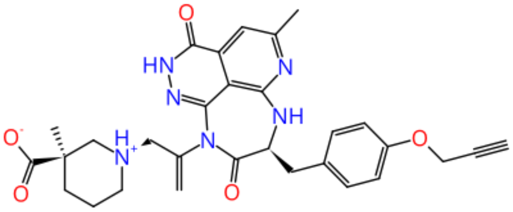
125	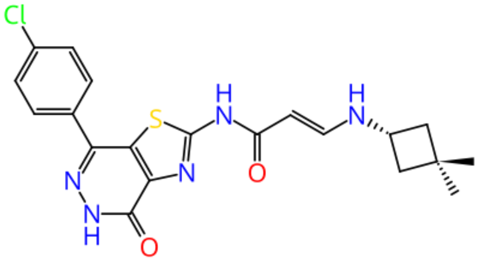	126	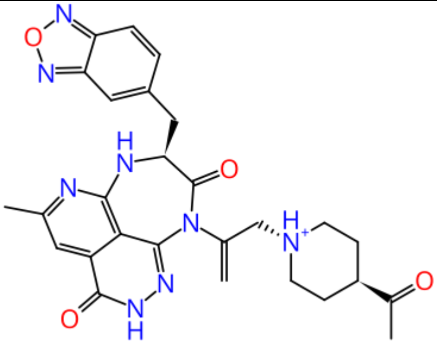
127	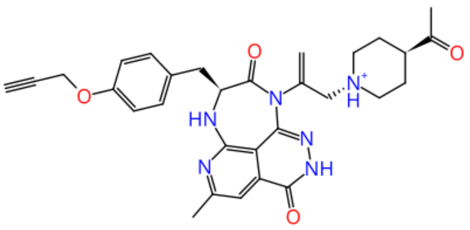	128	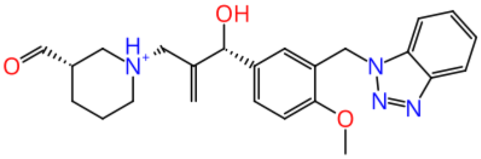

129	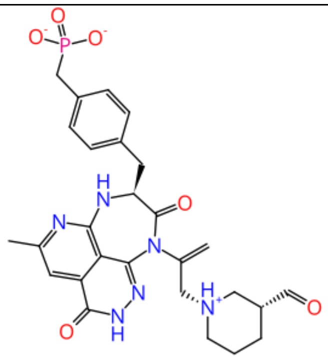	130	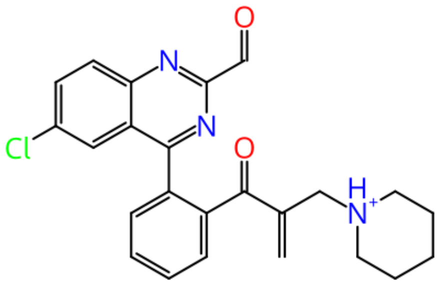
131	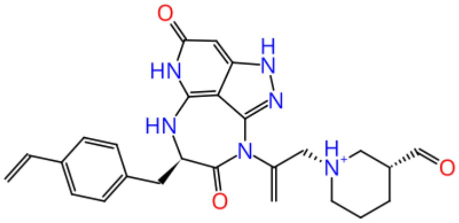	132	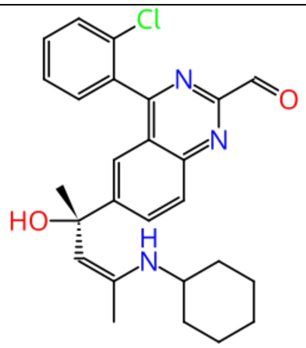
133	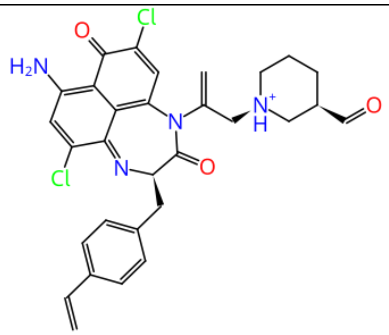	134	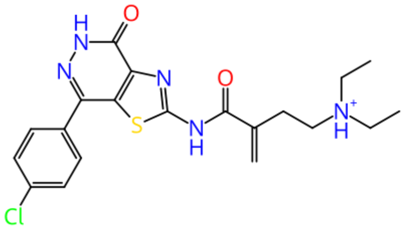
135	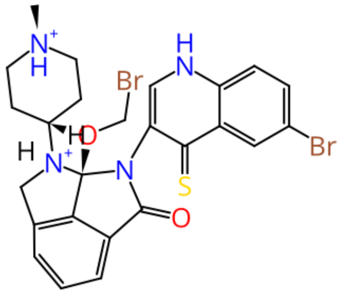	136	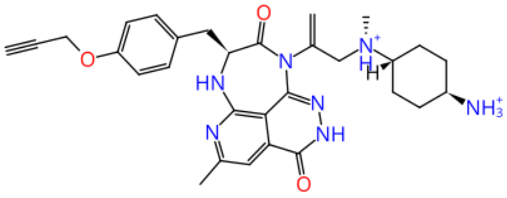
137	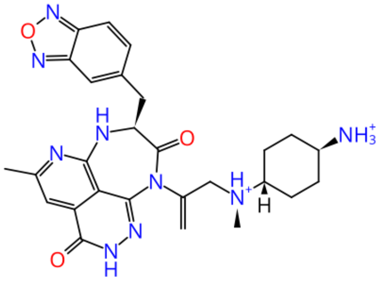	138	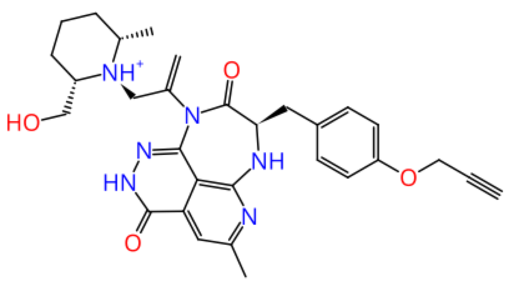

139	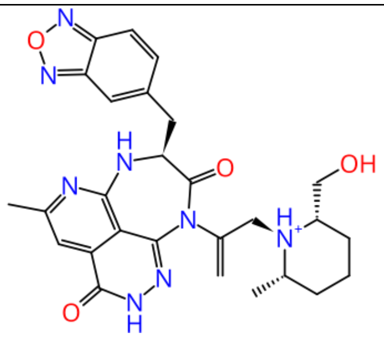	140	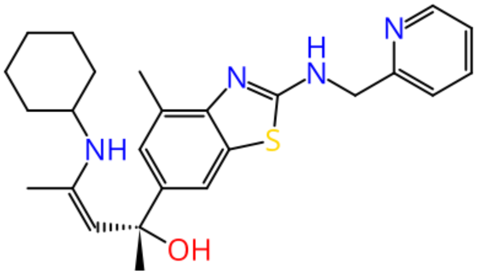
141	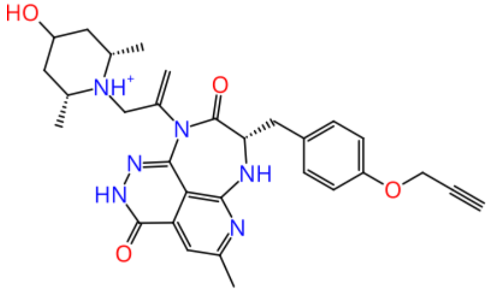	142	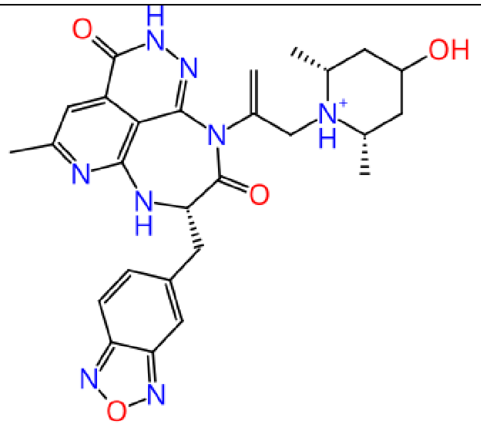
143	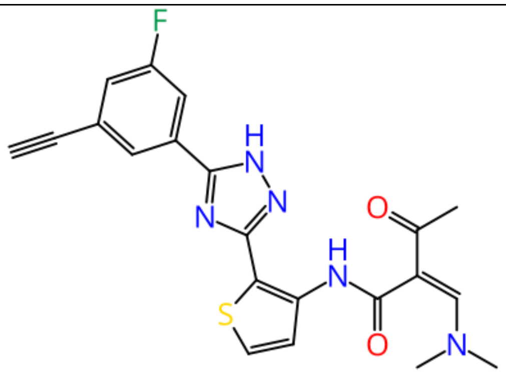	144	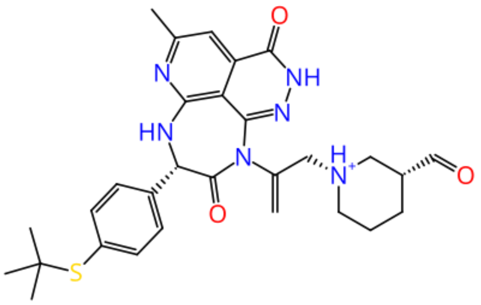
145	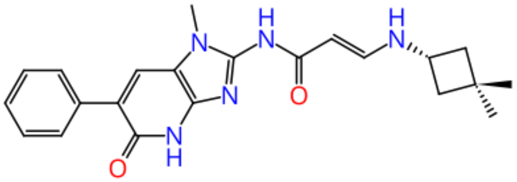	146	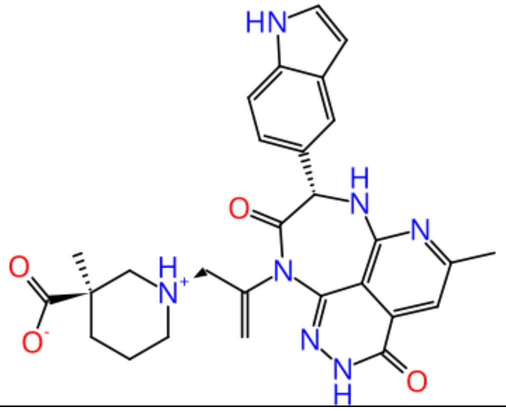

147	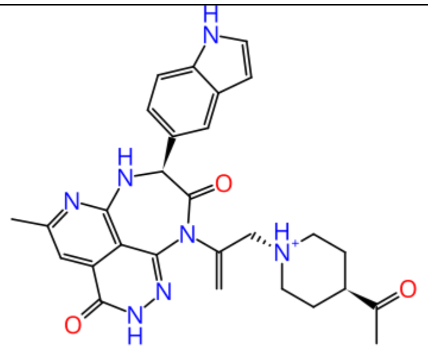	148	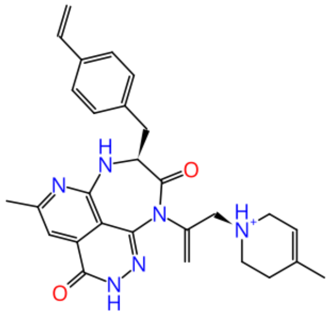
149	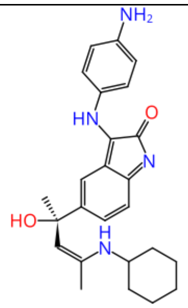	150	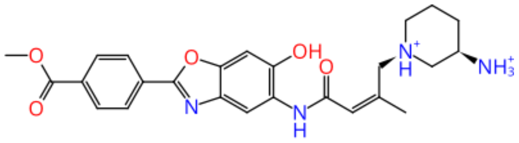
151	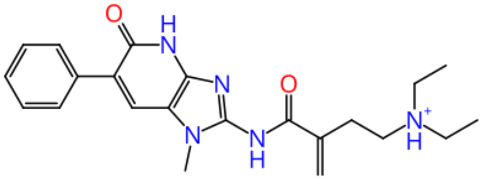	152	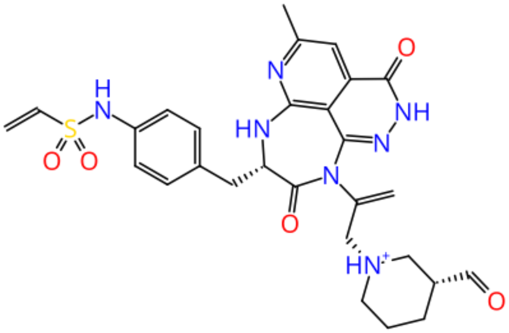
153	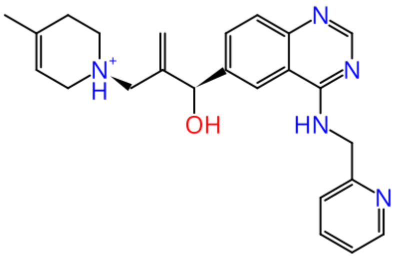	154	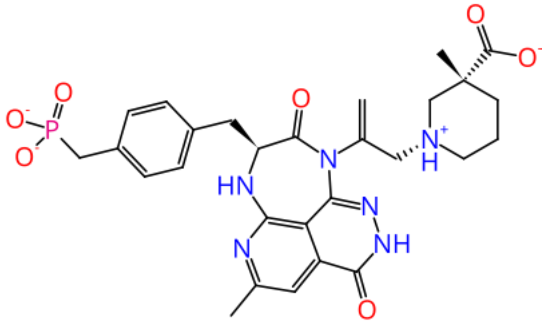
155	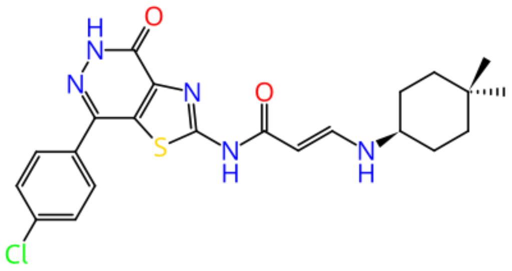	156	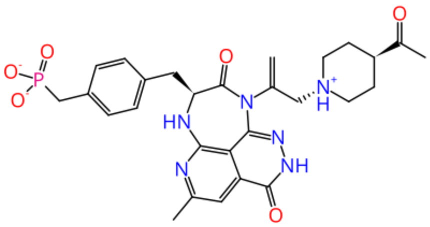

157	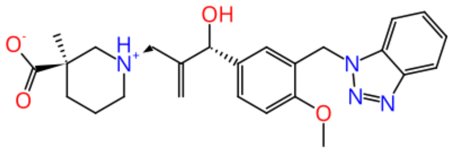	158	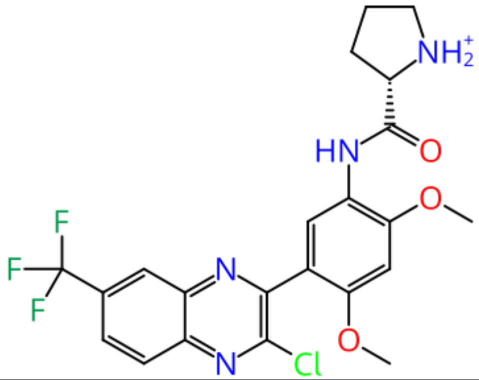
159	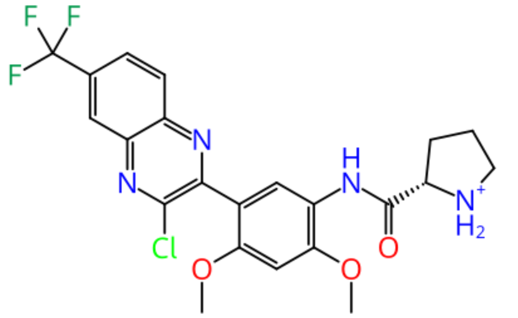	160	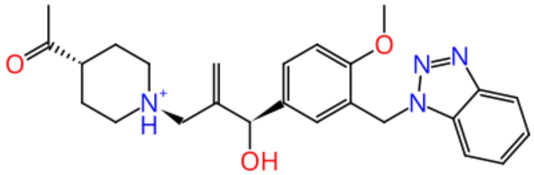
161	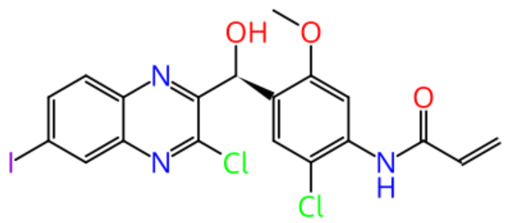	162	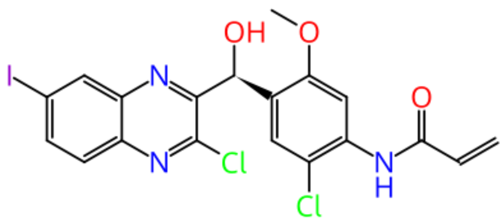
163	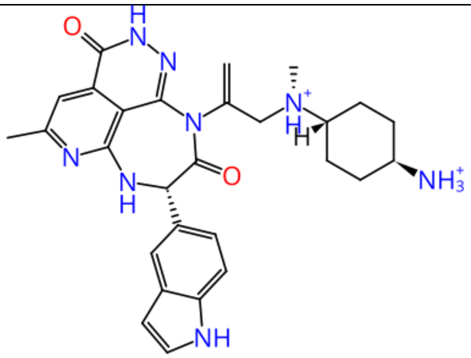	164	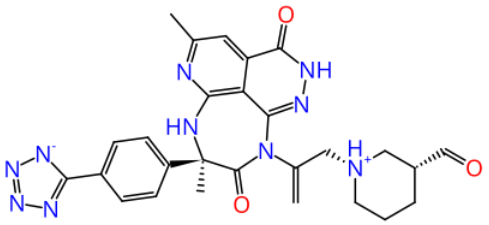

165	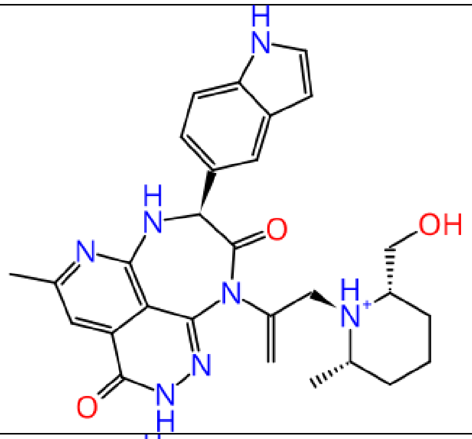	166	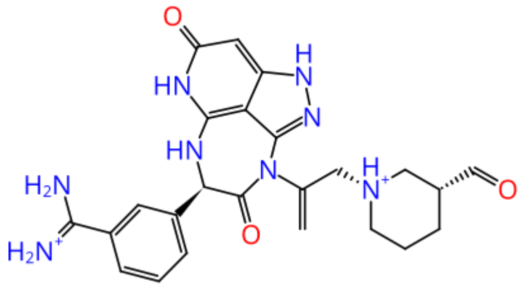
167	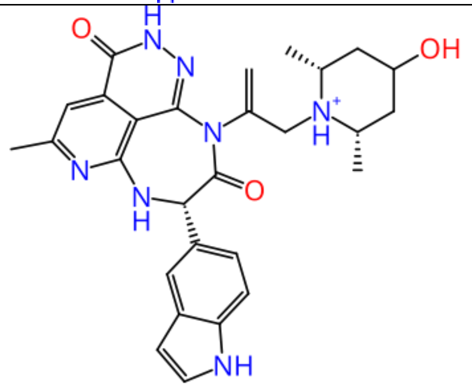	168	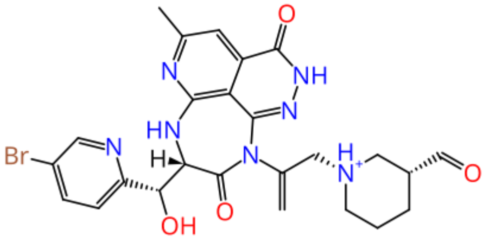
169	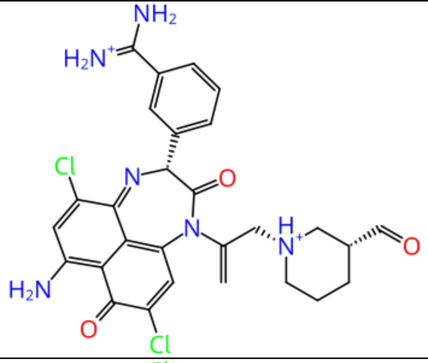	170	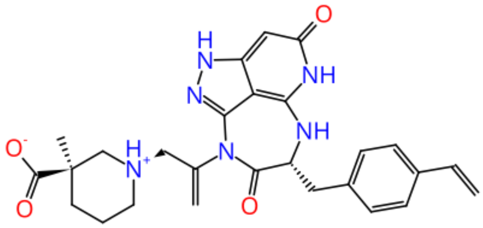
171	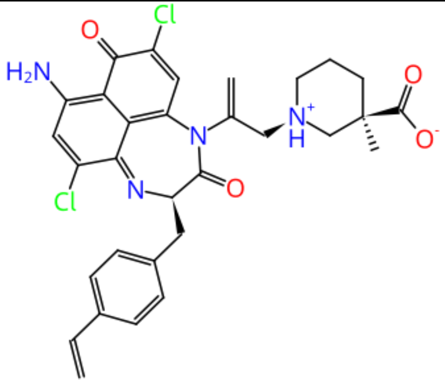	172	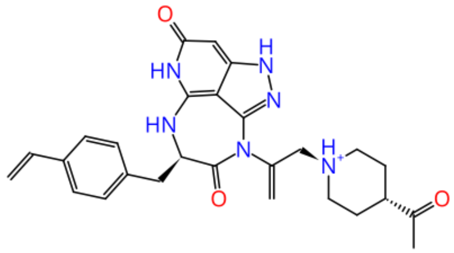

173	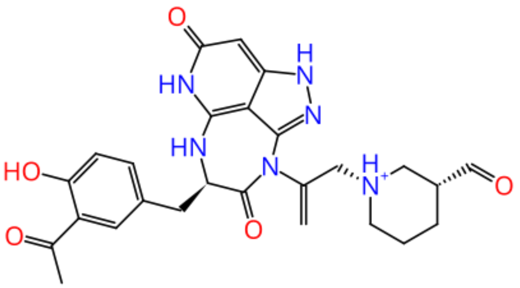	174	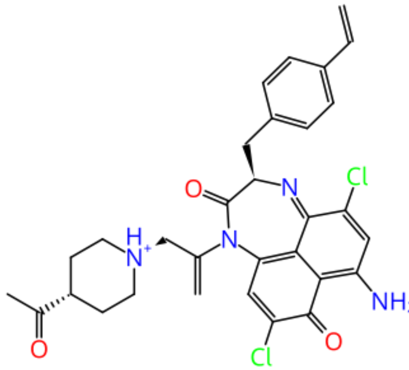
175	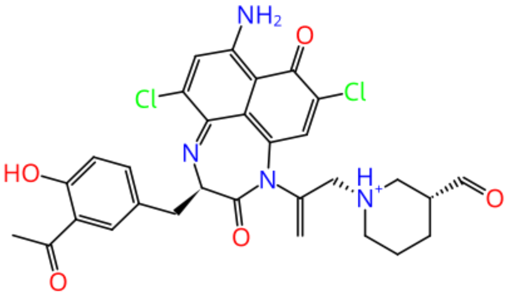	176	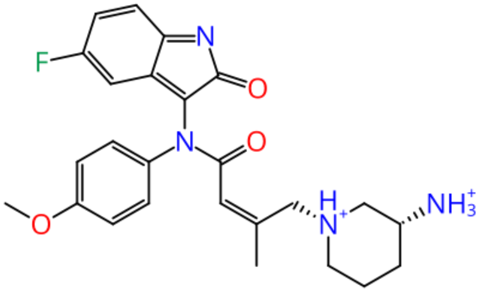
177	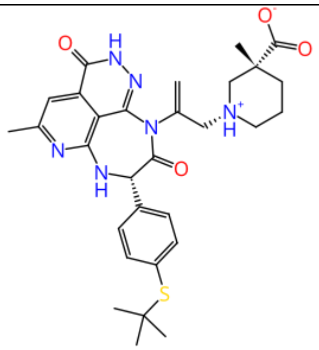	178	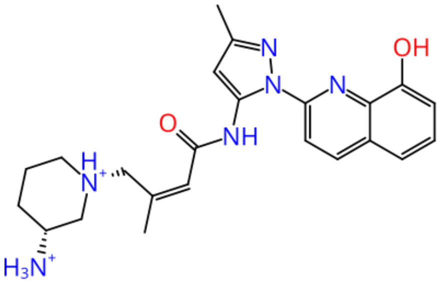
179	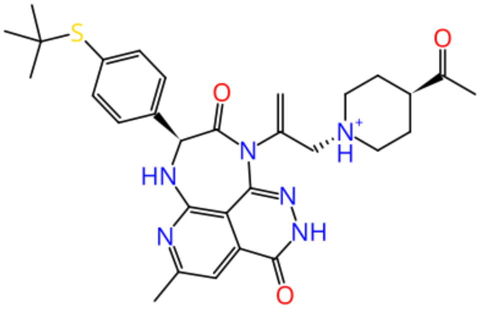	180	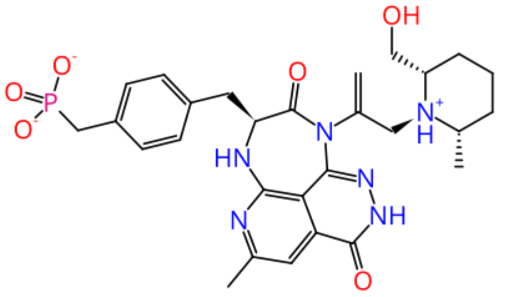
181	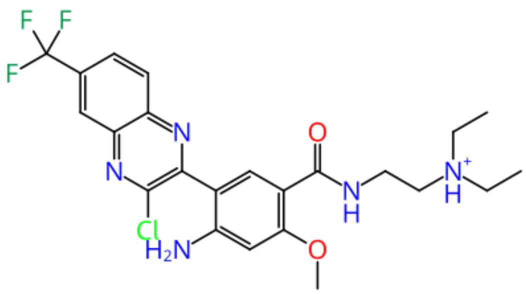	182	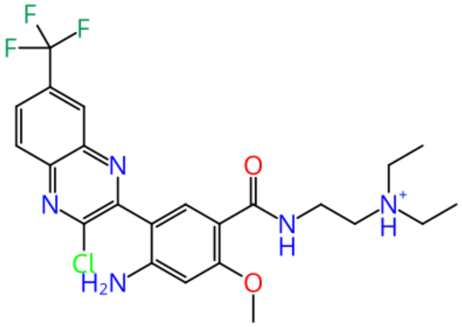

183	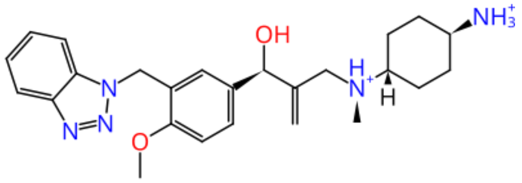	184	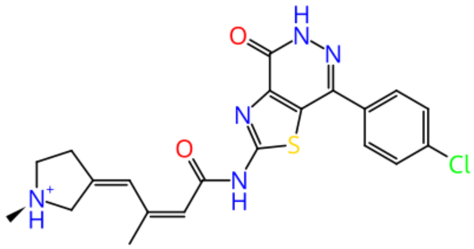
185	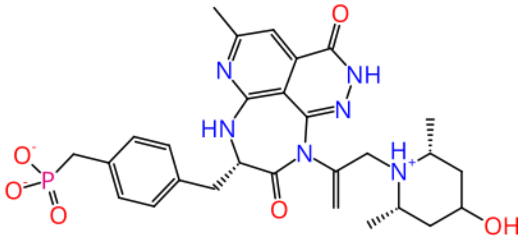	186	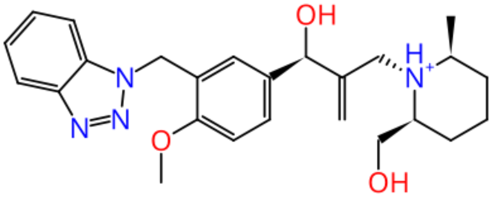
187	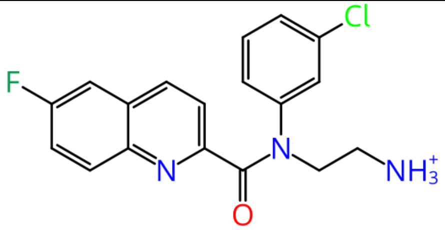	188	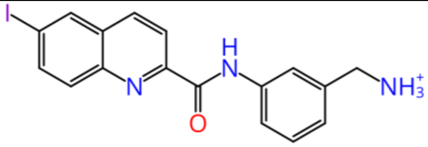
189	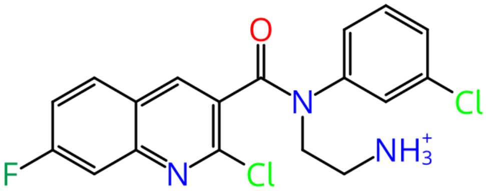	190	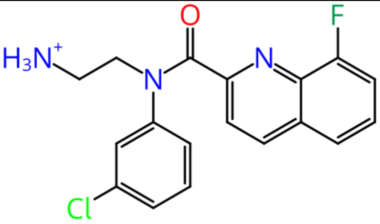
191	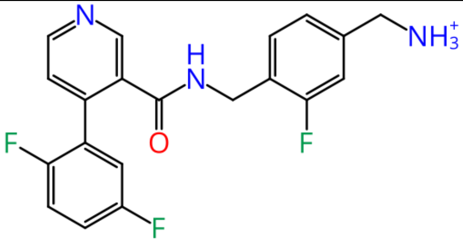	192	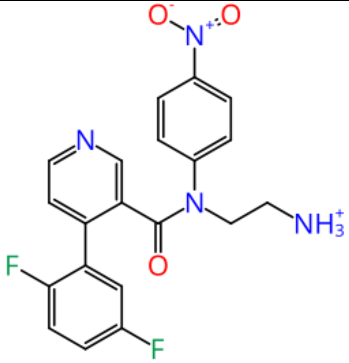
193	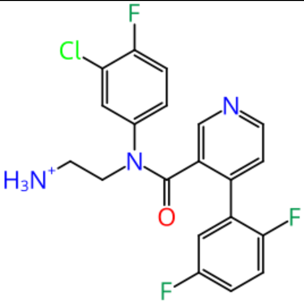	194	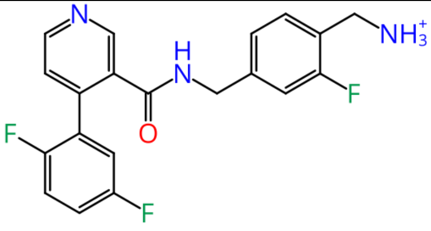

195	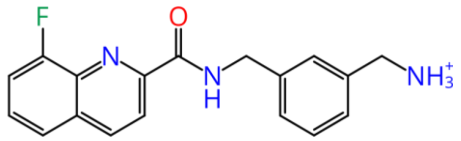	196	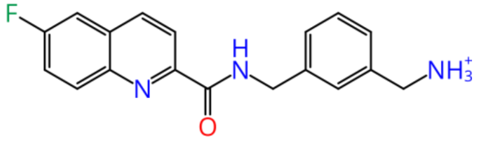
197	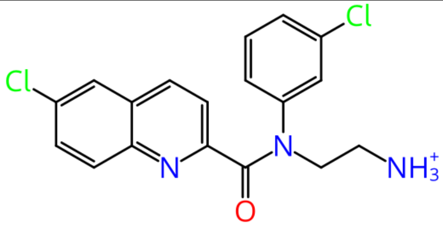	198	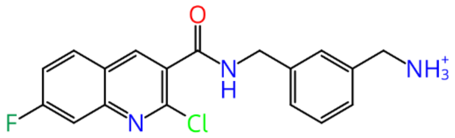
199	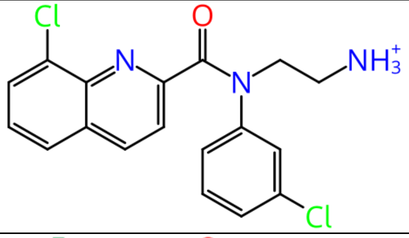	200	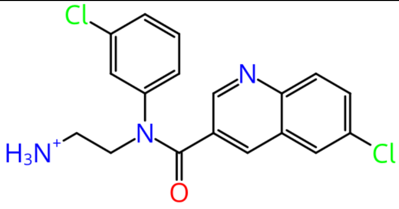
201	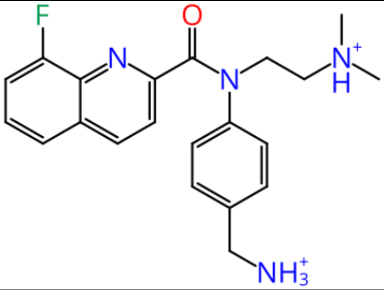	202	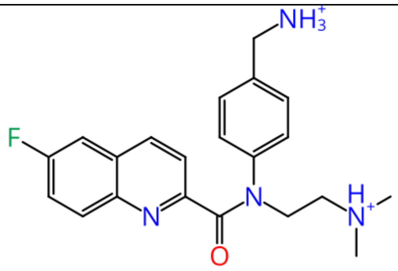
203	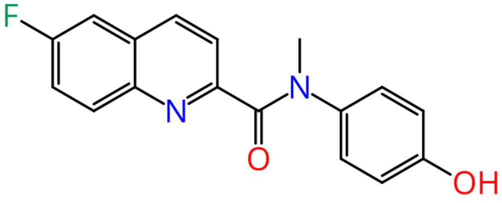	204	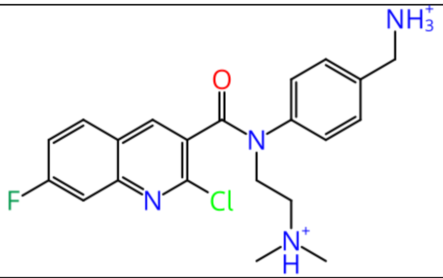
205	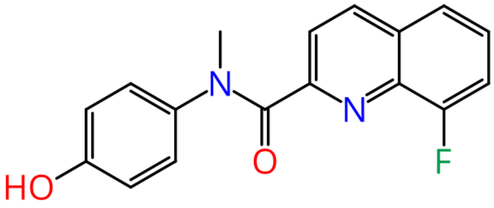	206	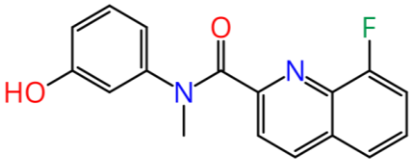
207	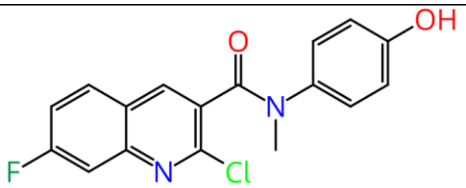	208	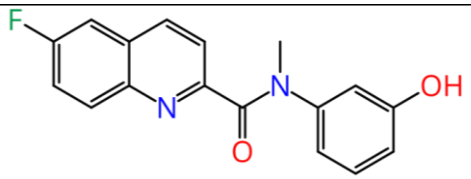
209	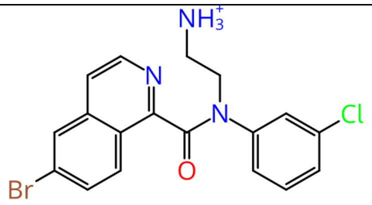	210	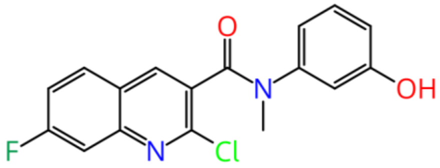

211	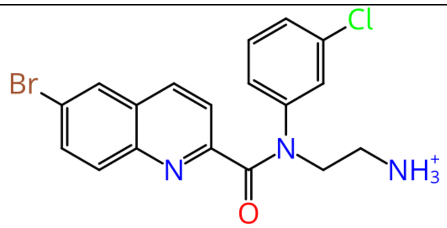	212	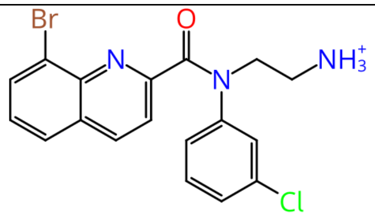
213	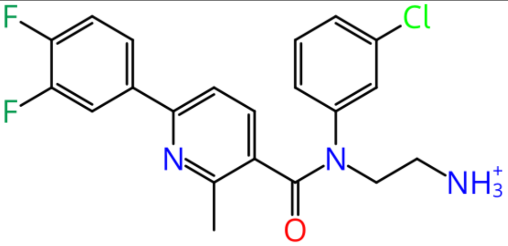	214	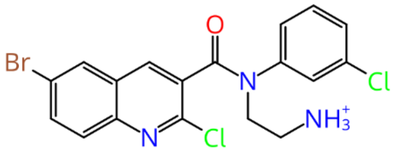
215	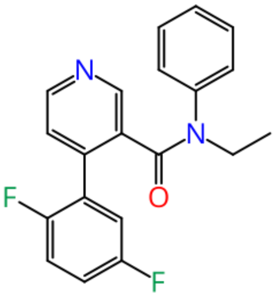	216	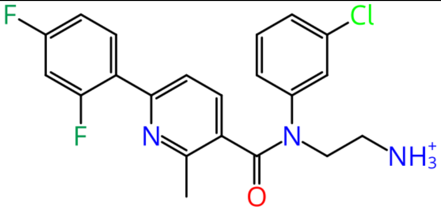
217	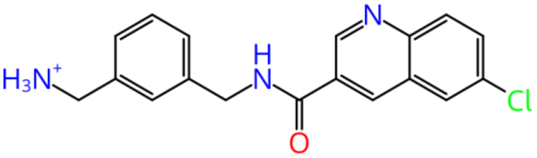	218	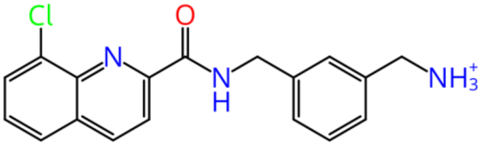
219	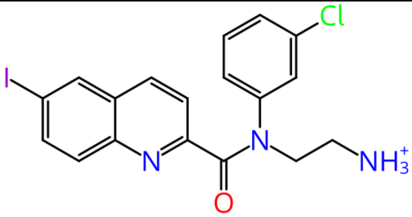	220	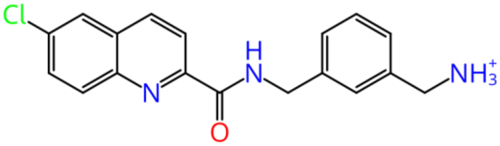
221	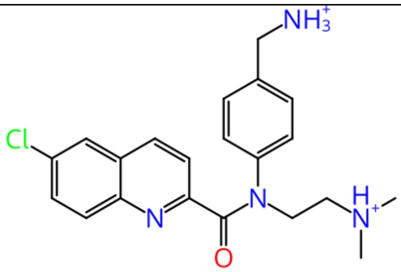	222	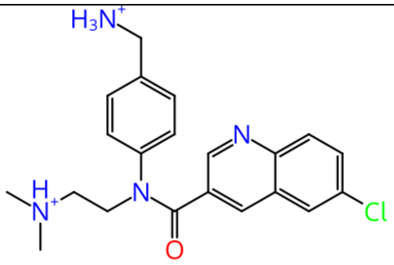
223	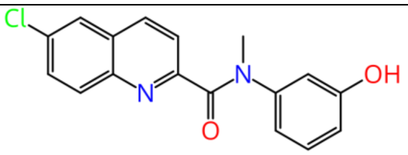	224	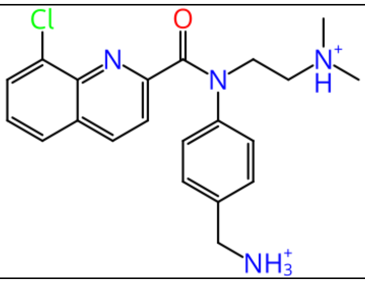
225	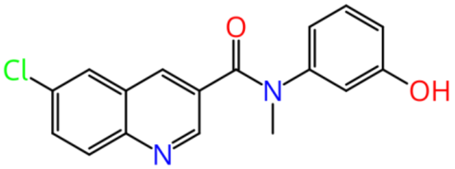	226	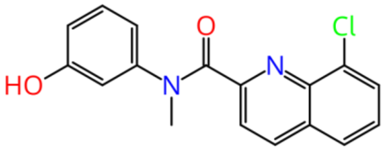

227	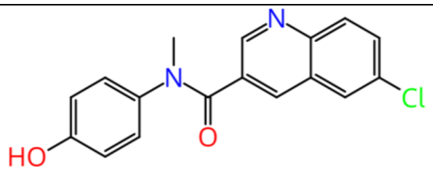	228	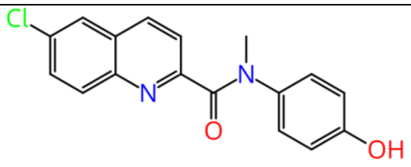
229	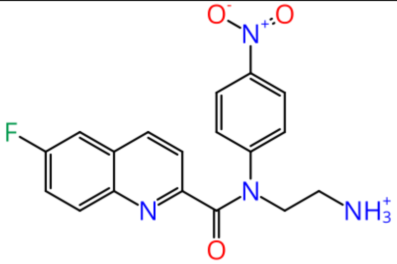	230	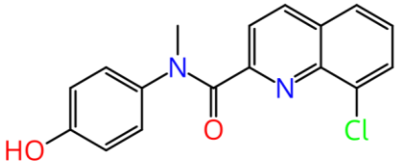
231	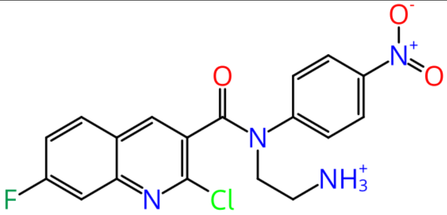	232	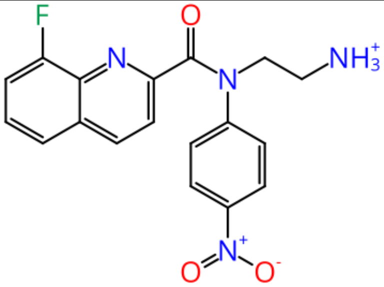
233	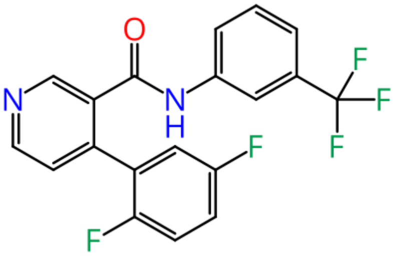	234	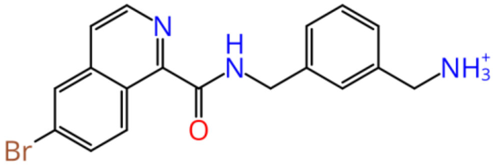
235	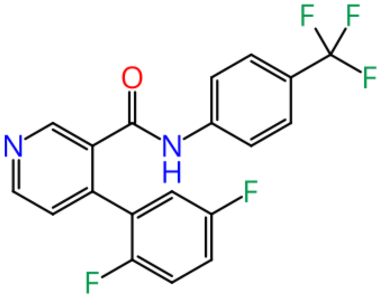	236	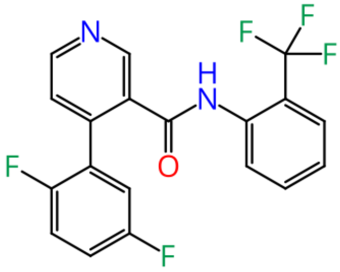
237	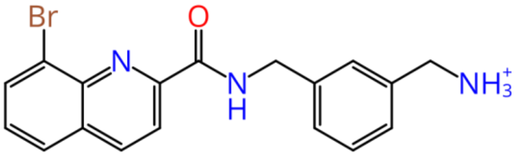	238	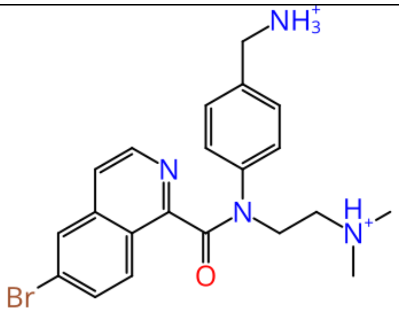
239	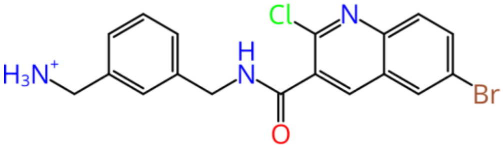	240	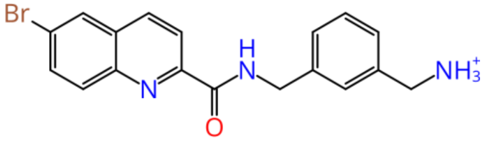

241	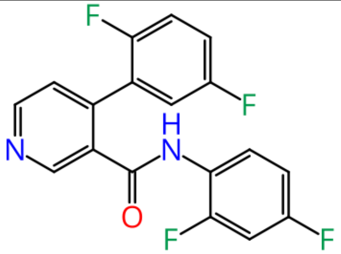	242	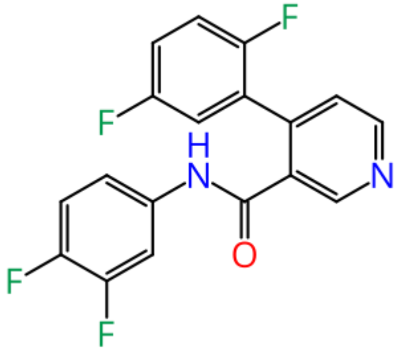
243	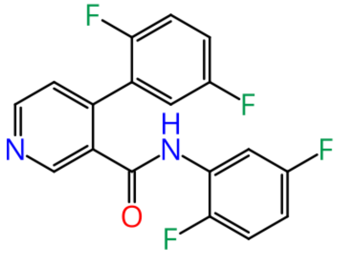	244	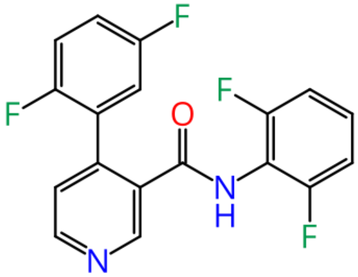
245	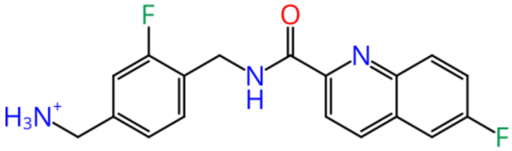	246	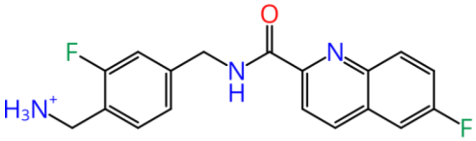
247	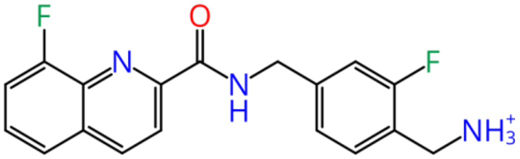	248	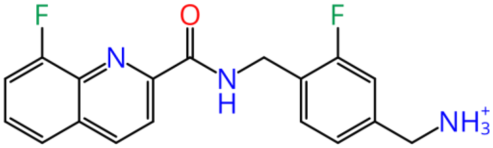
249	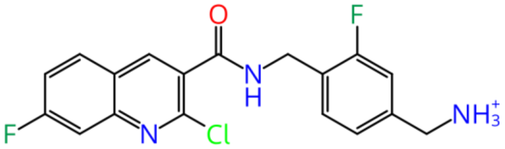	250	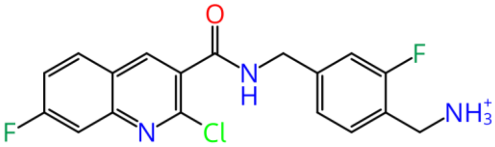
251	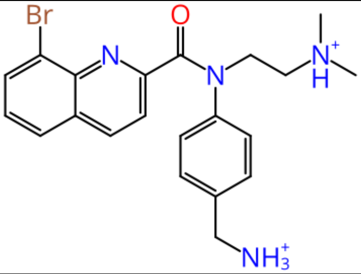	252	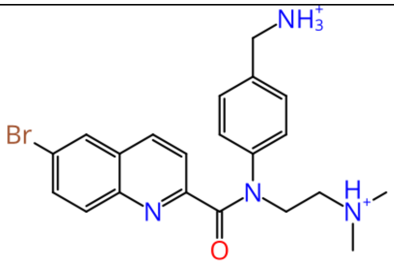
253	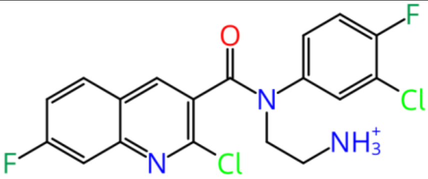	254	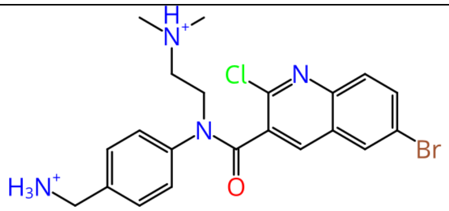
255	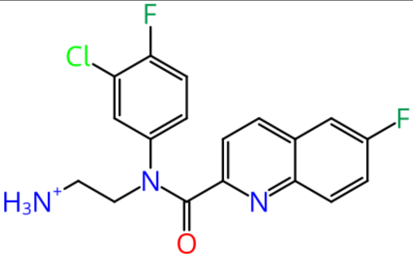	256	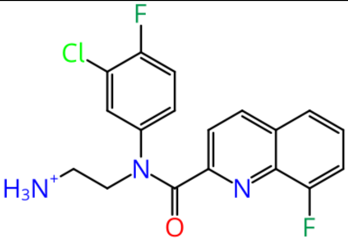

257	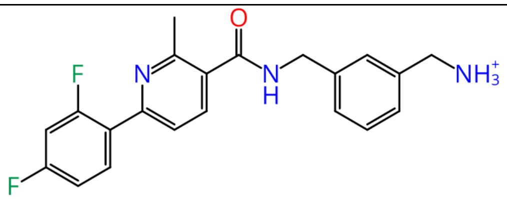	258	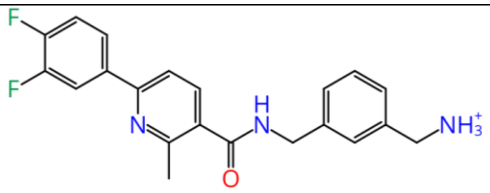
259	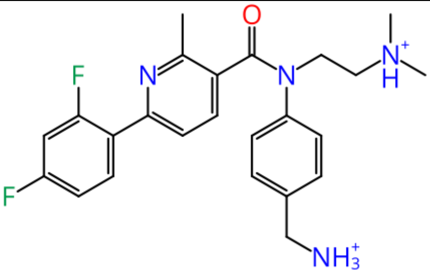	260	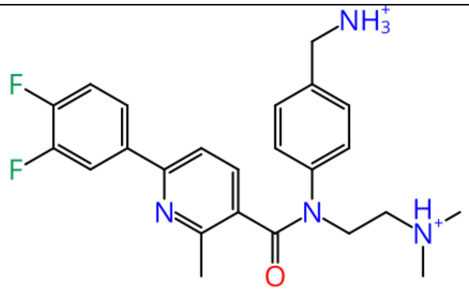
261	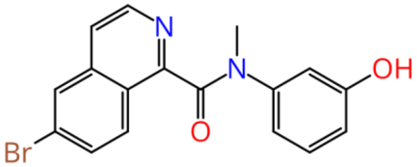	262	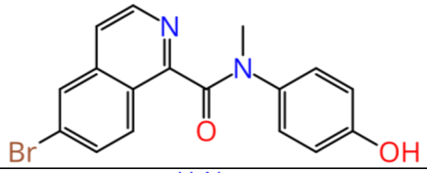
263	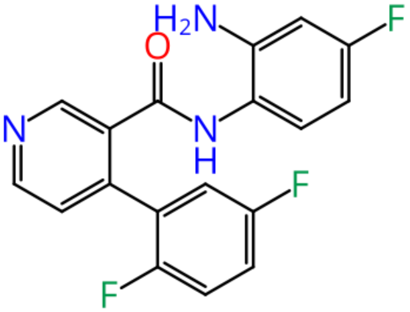	264	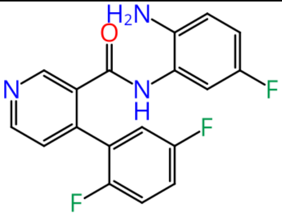
265	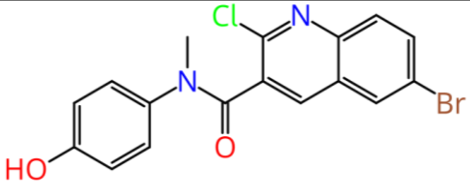	266	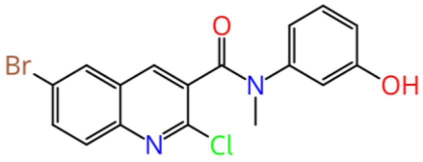
267	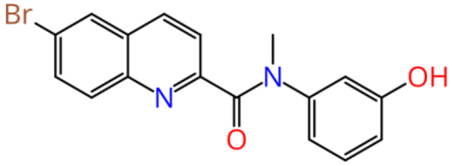	268	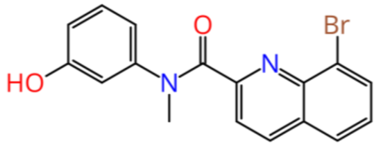
269	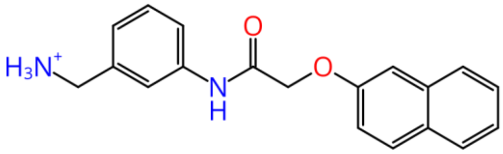	270	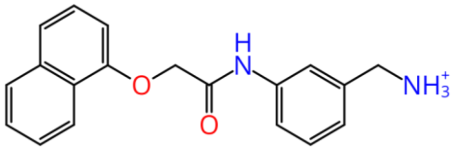
271	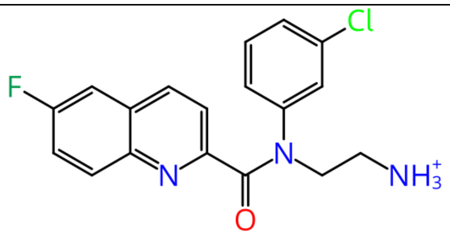	272	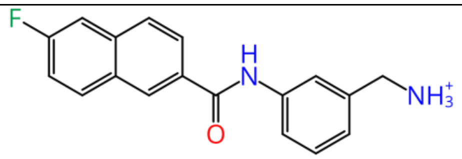

273	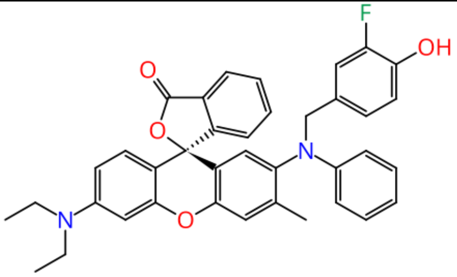	274	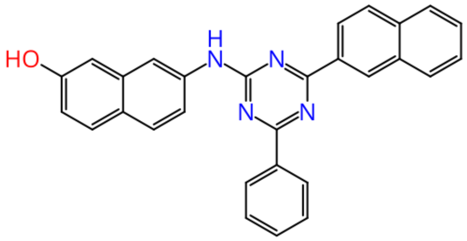
275	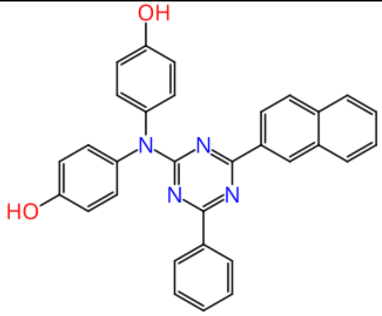	276	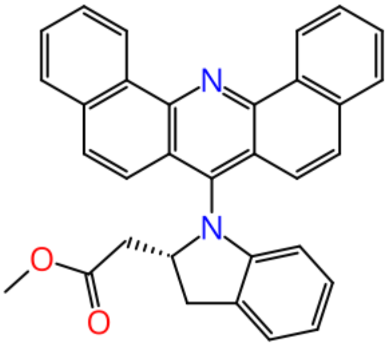
277	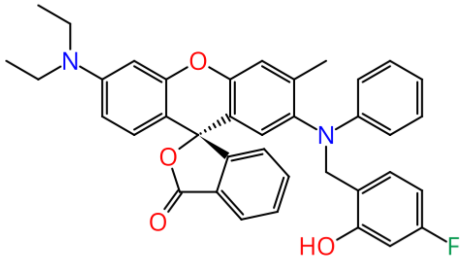	278	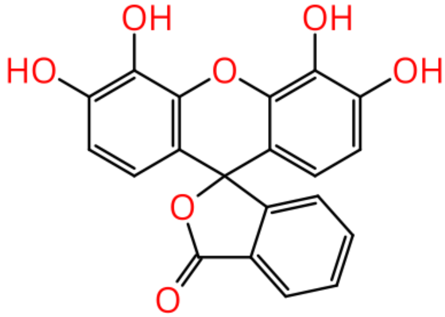
279	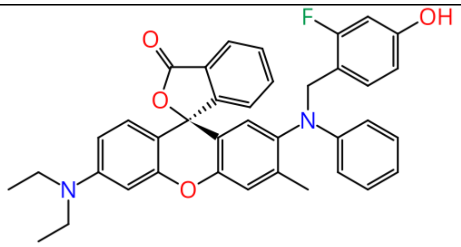	280	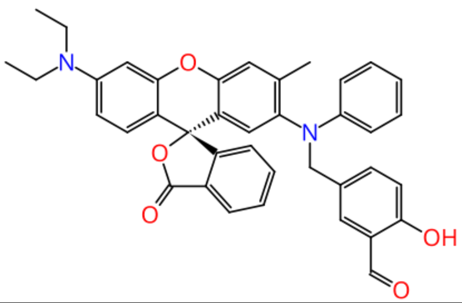
281	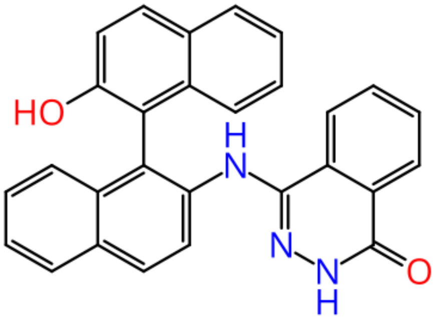	282	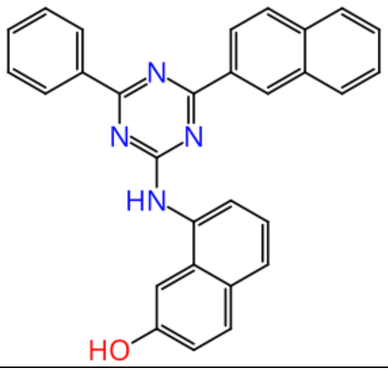

283	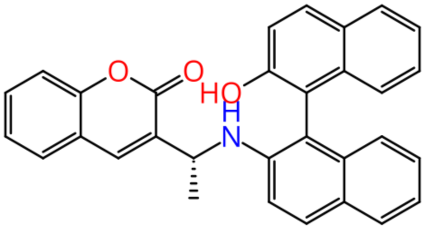	284	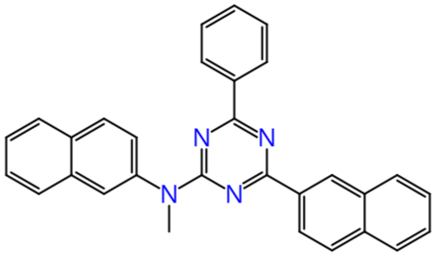
285	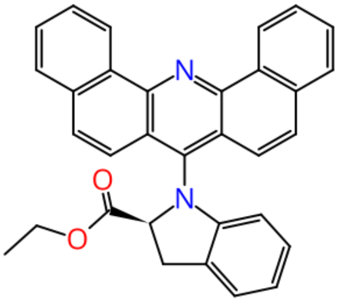	286	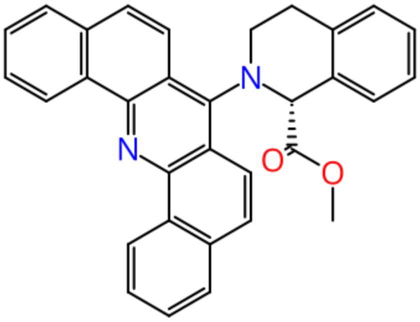
287	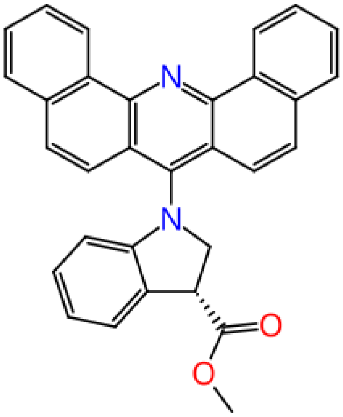	288	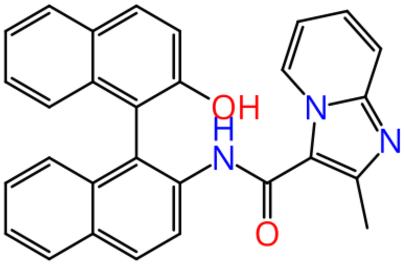
289	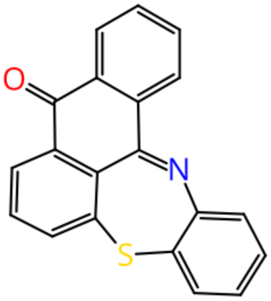	290	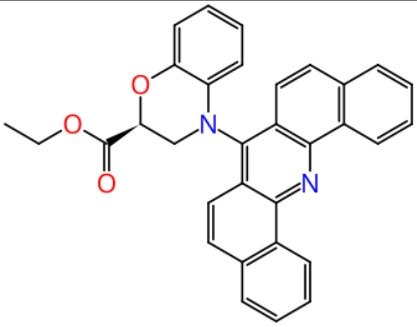
291	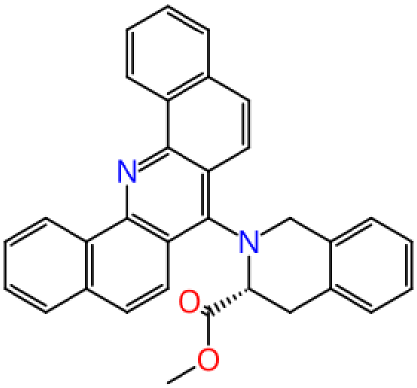	292	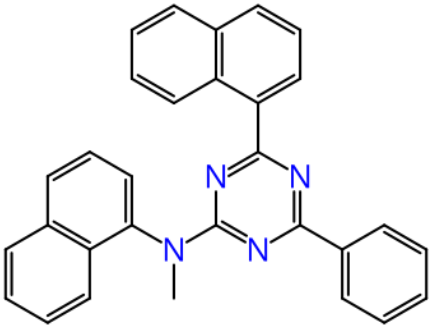

293	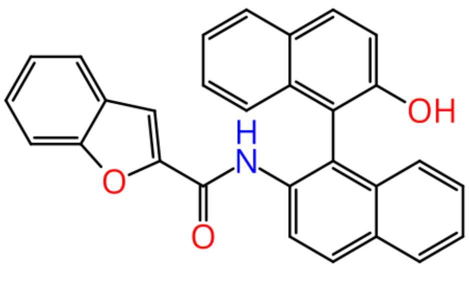	294	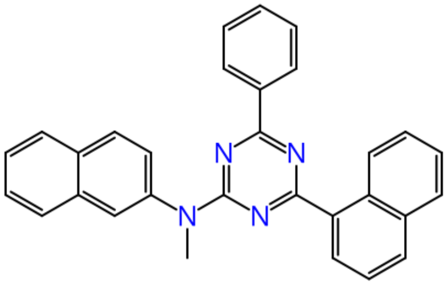
295	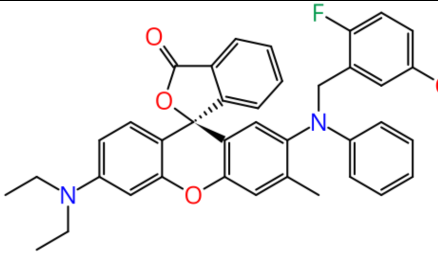	296	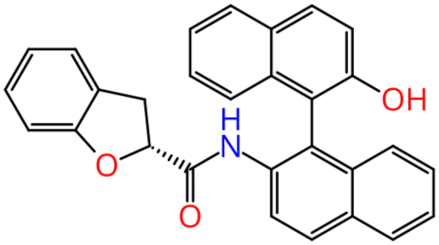
297	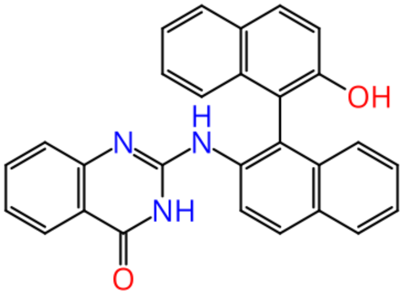	298	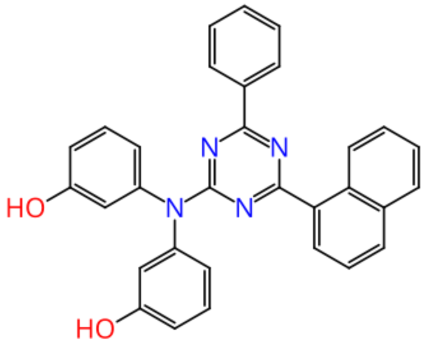
299	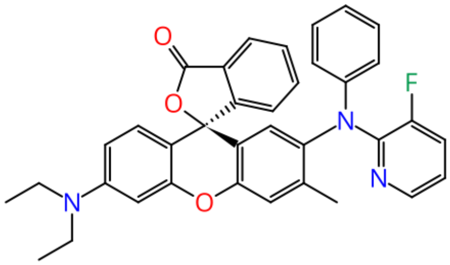	300	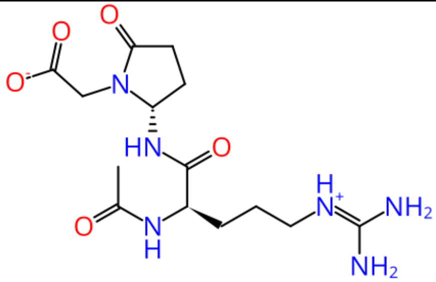
301	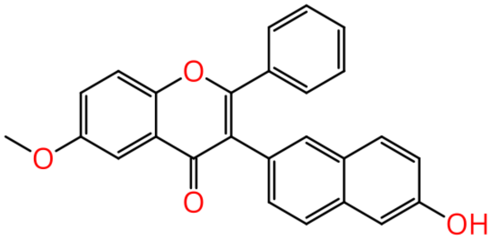	302	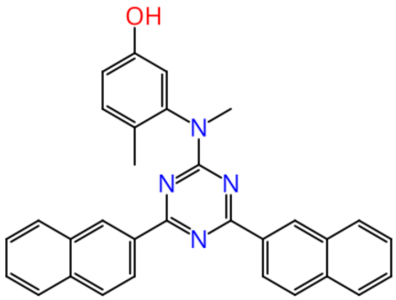
303	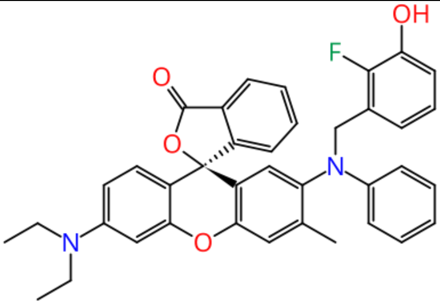	304	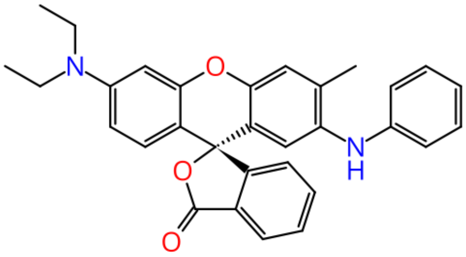

305	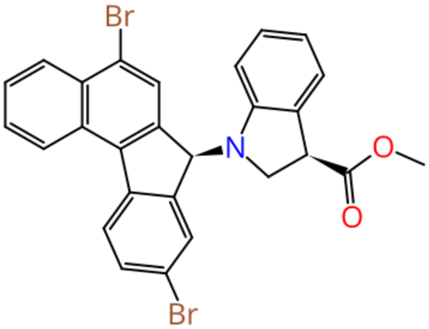	306	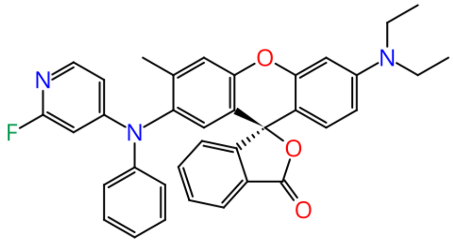
307	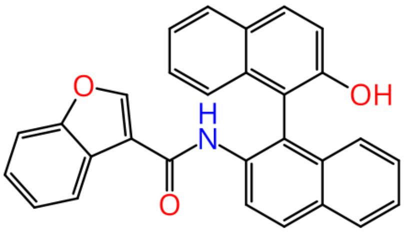	308	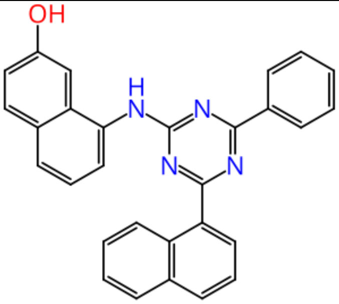
309	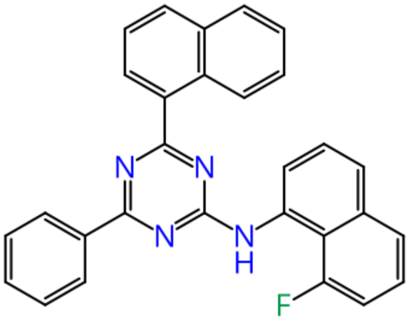	310	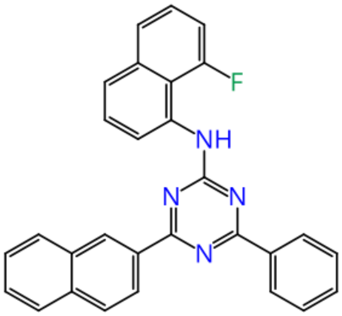
311	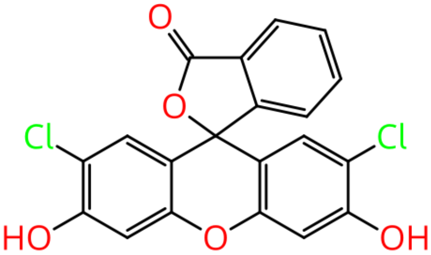	312	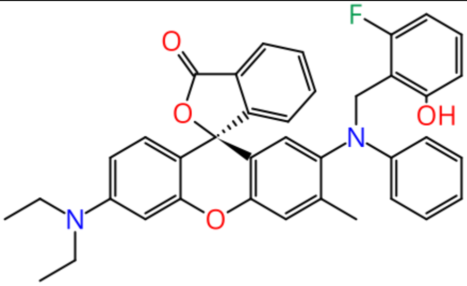
313	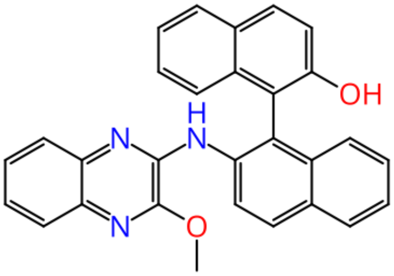	314	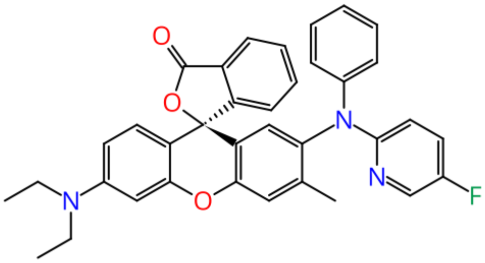
315	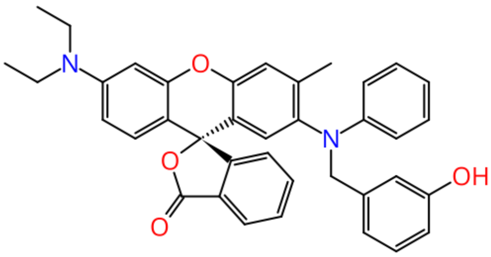	316	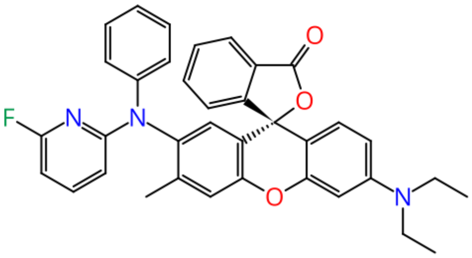

317	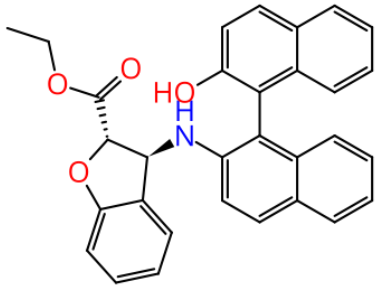	318	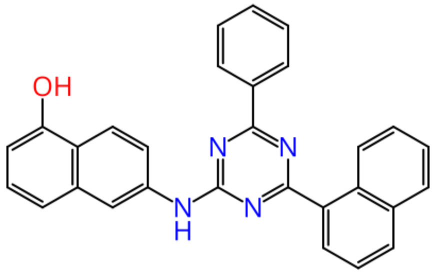
319	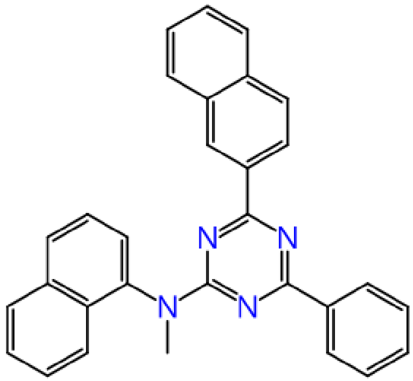	320	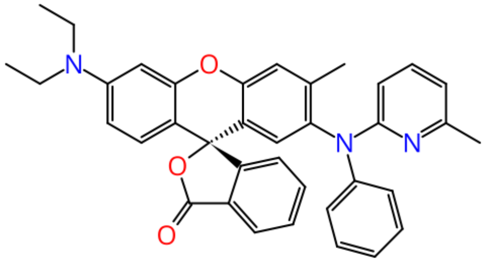
321	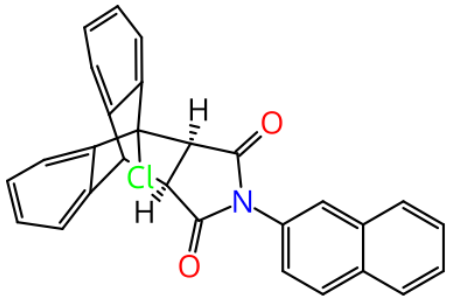	322	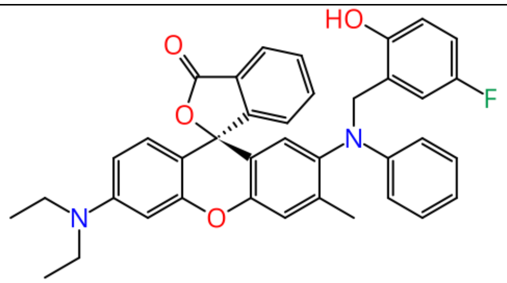
323	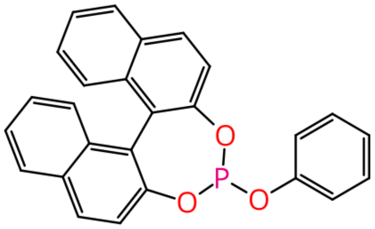	324	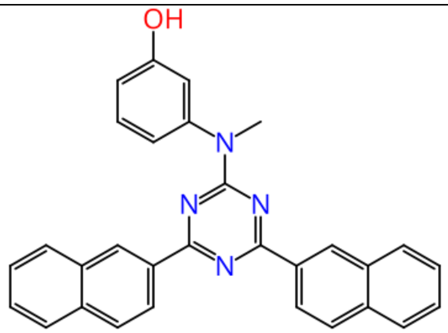
325	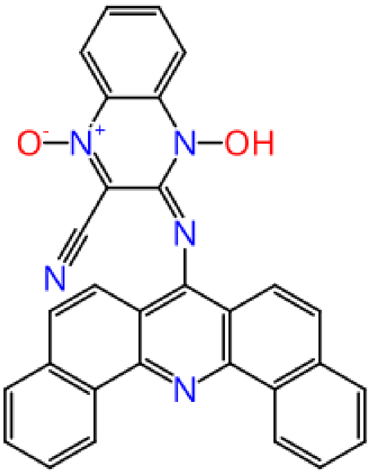	326	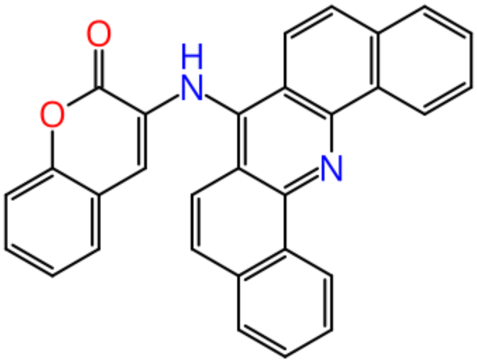

327	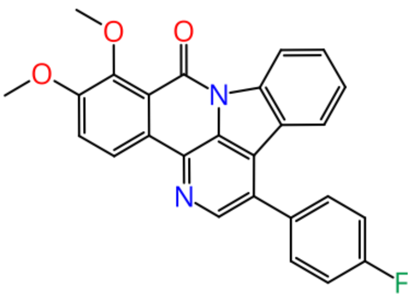	328	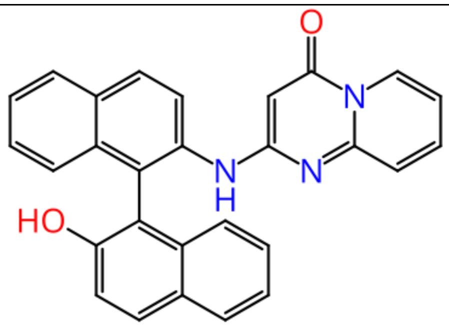
329	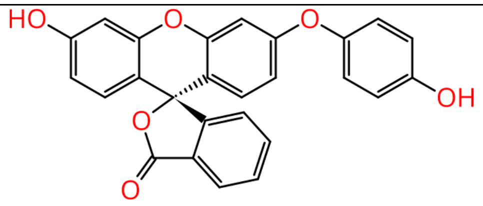	330	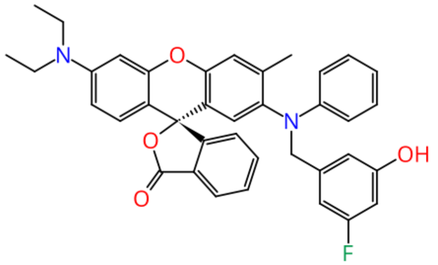
331	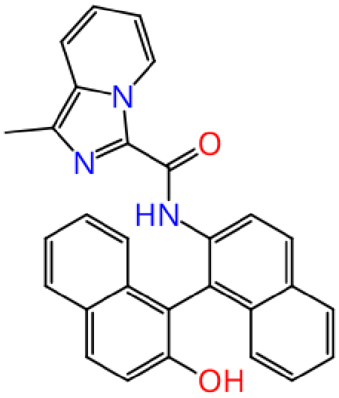	332	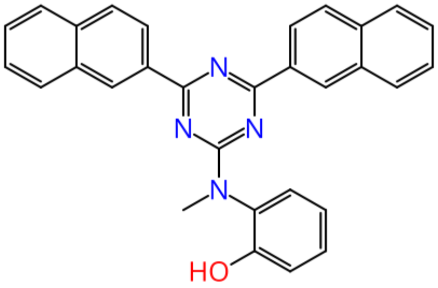
333	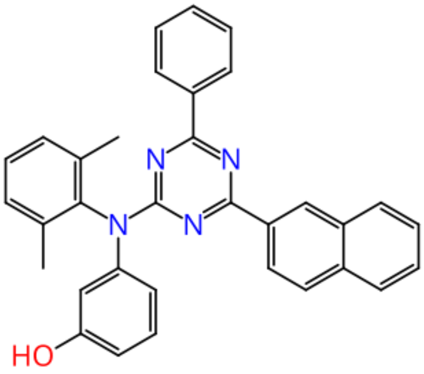	334	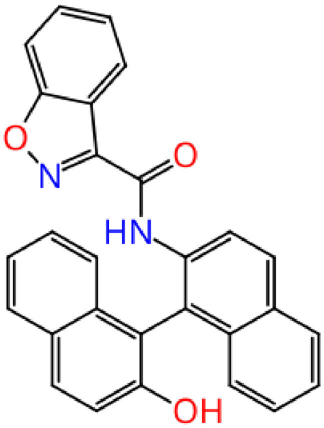
335	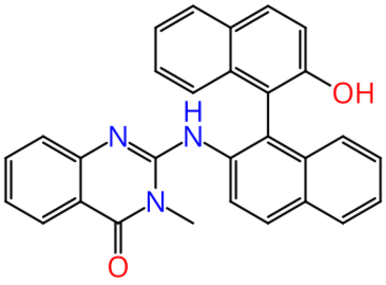	336	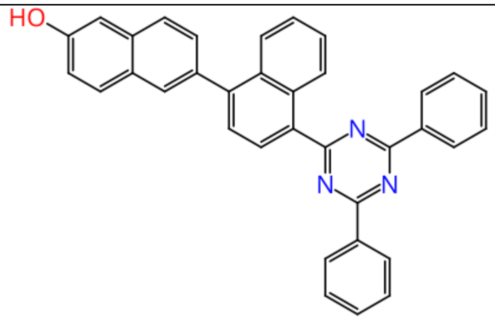

337	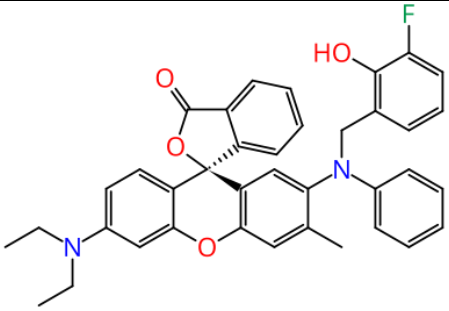	338	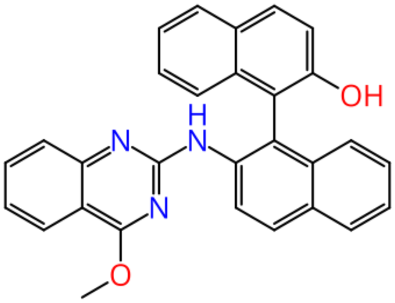
339	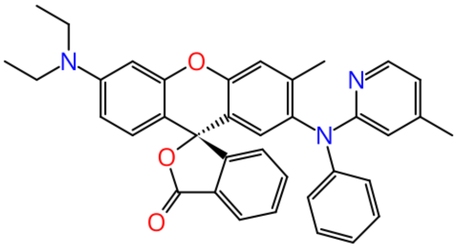	340	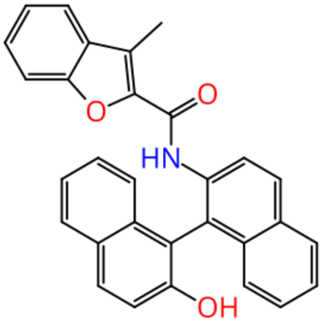
341	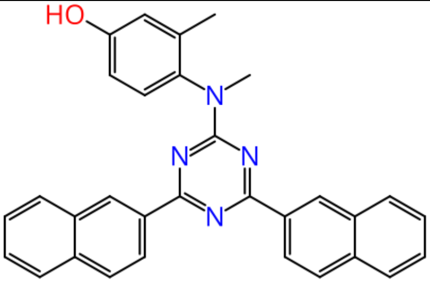	342	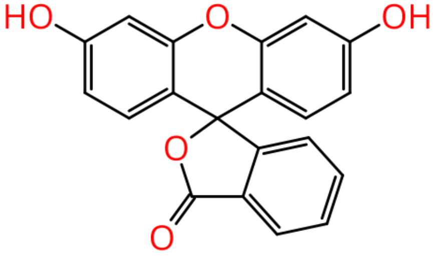
343	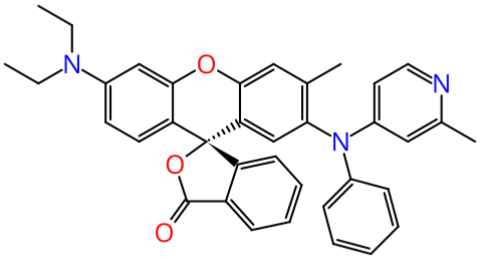	344	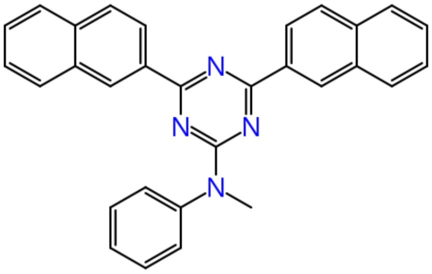
345	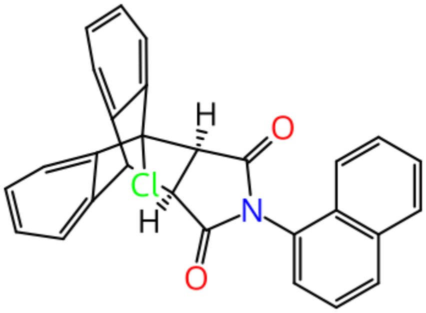	346	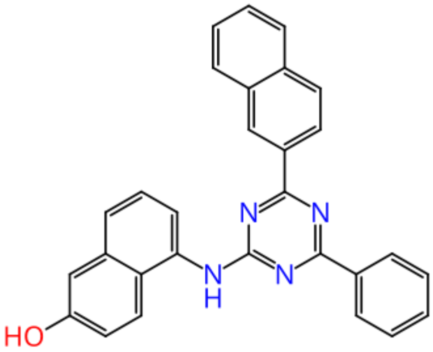

347	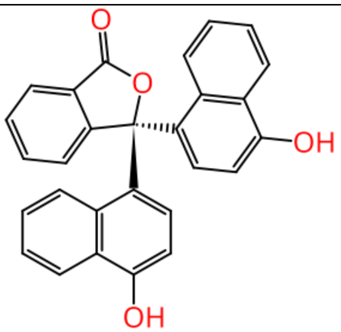	348	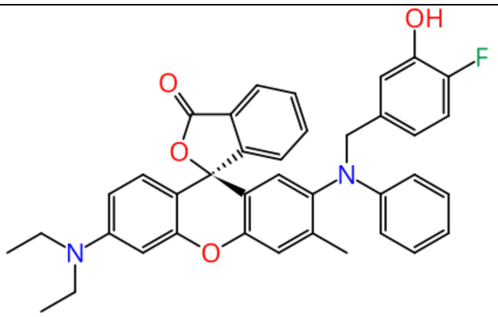
349	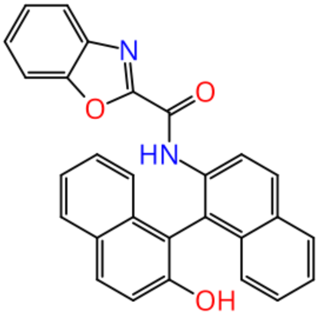	350	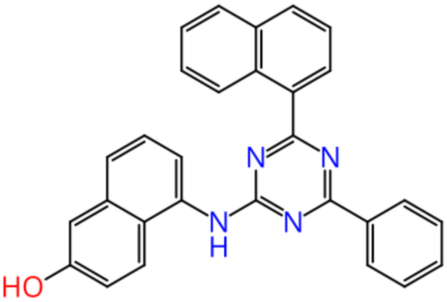
351	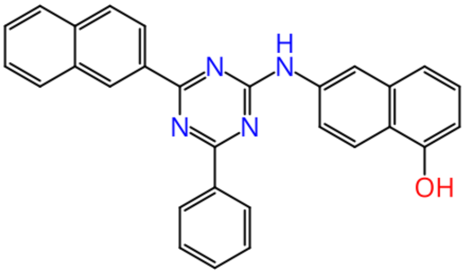	352	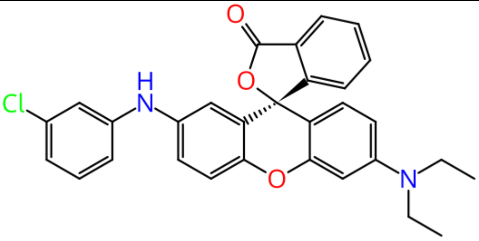
353	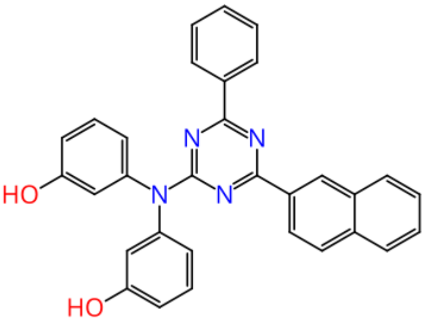	354	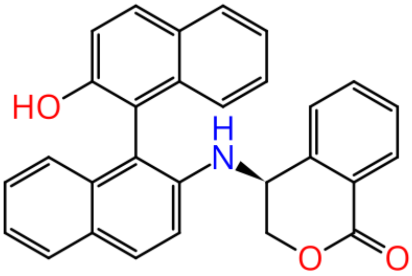
355	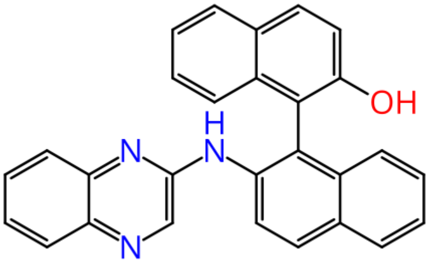	356	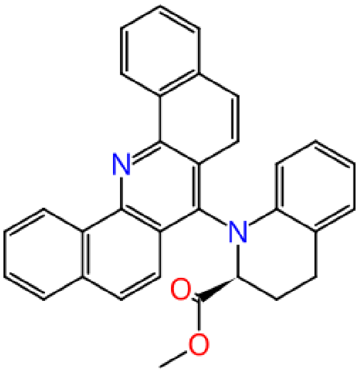

357	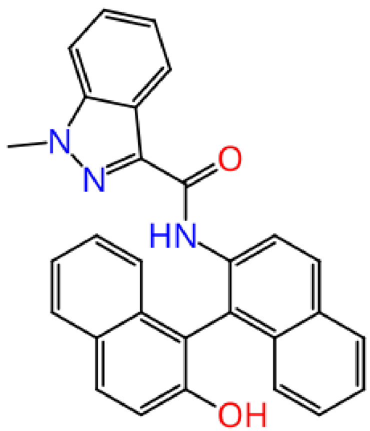	358	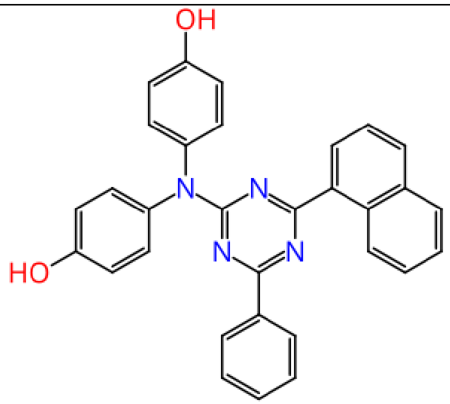
359	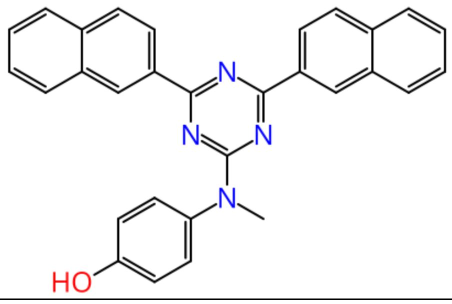	360	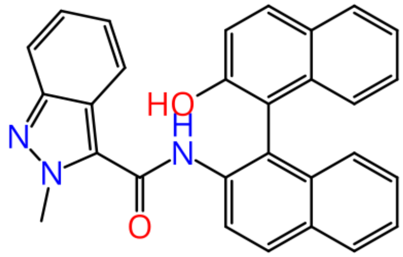
361	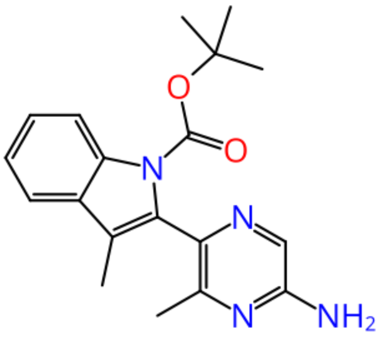	362	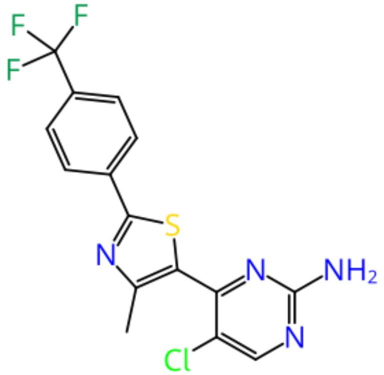
363	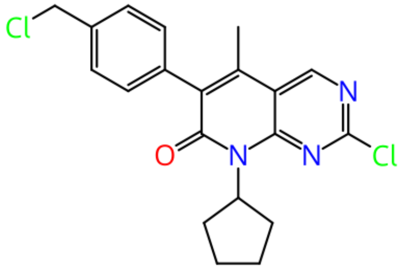	364	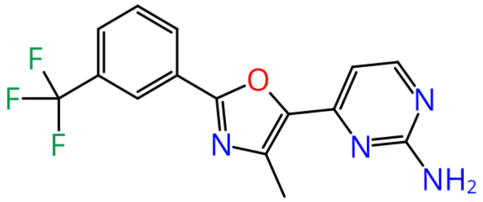
365	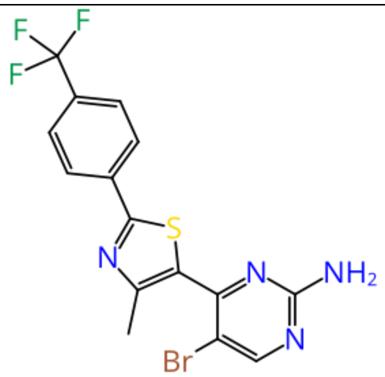	366	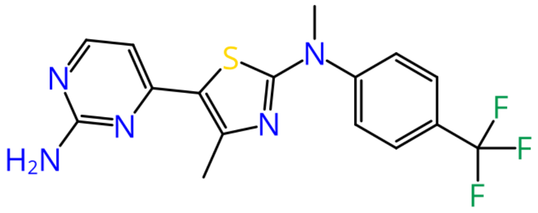

367	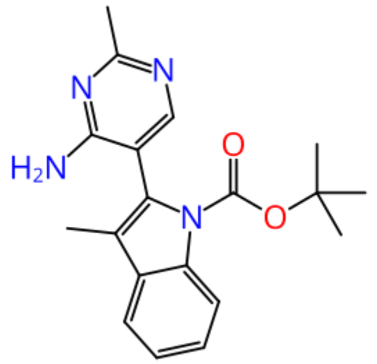	368	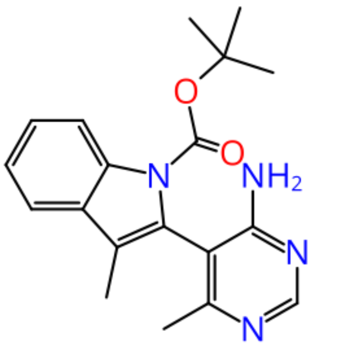
369	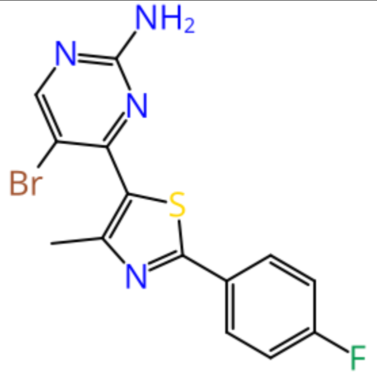	370	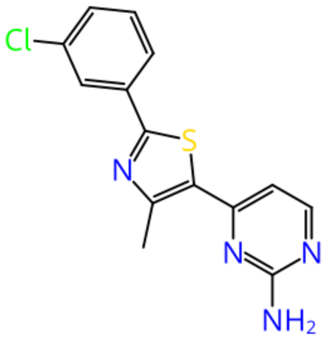
371	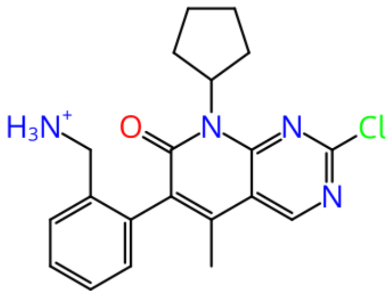	372	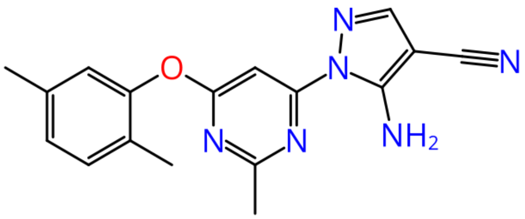
373	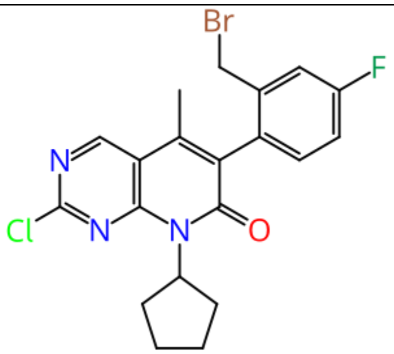	374	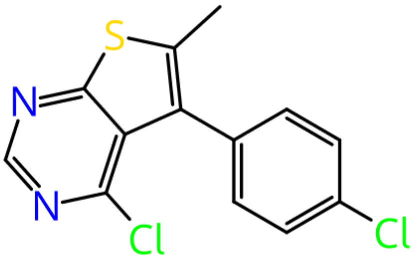
375	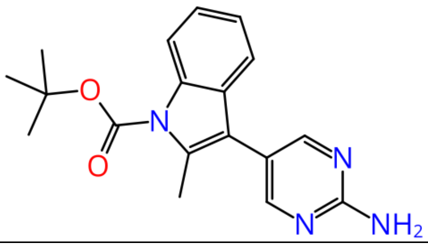	376	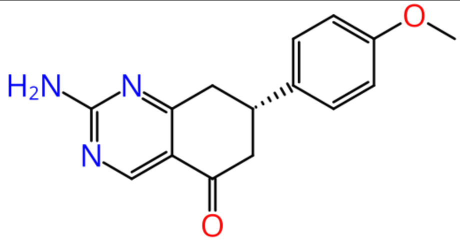

377	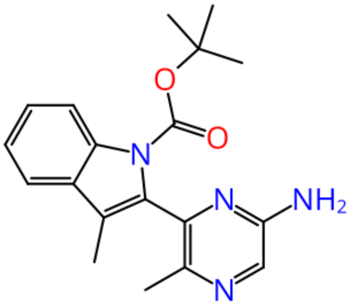	378	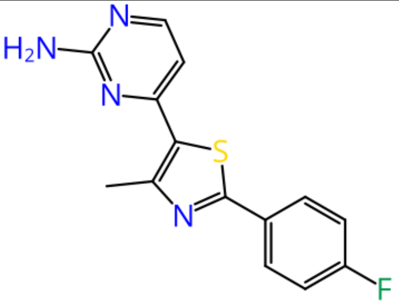
379	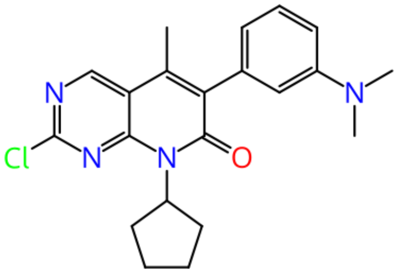	380	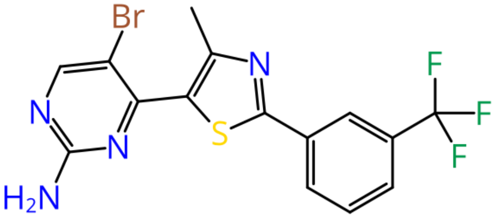
381	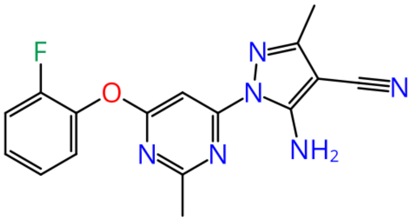	382	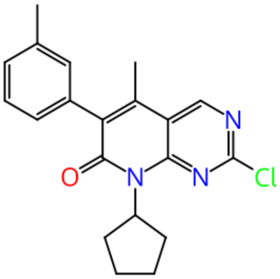
383	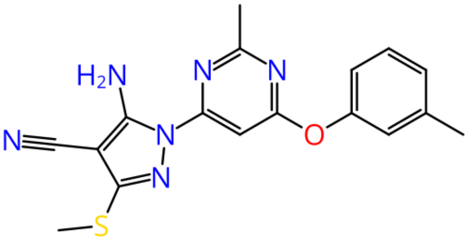	384	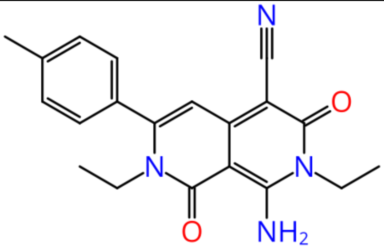
385	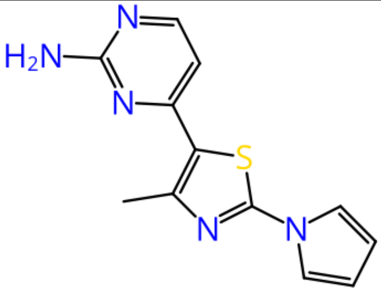	386	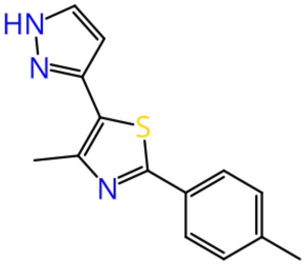
387	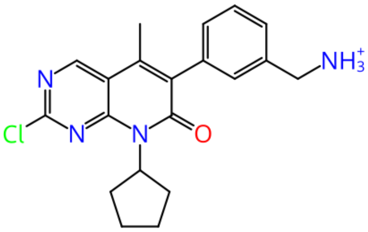	388	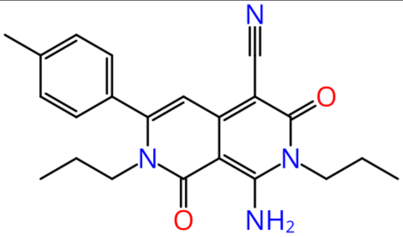

389	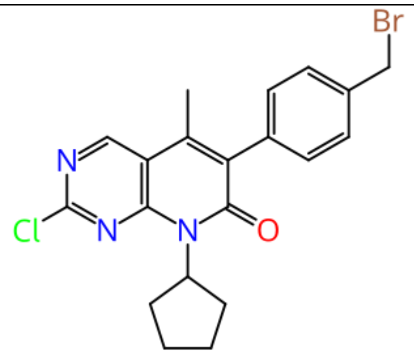	390	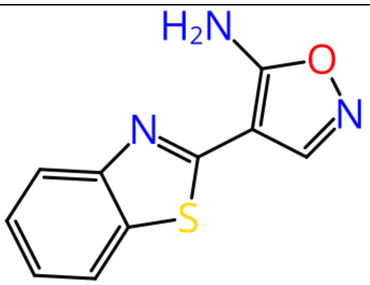
391	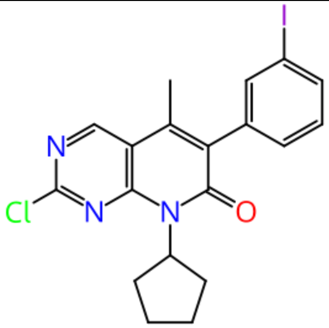	392	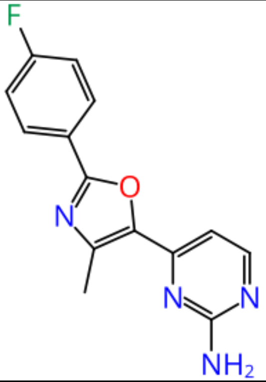
393	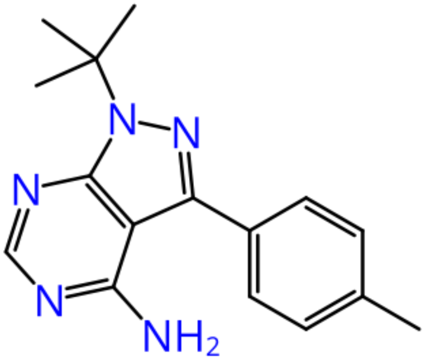	394	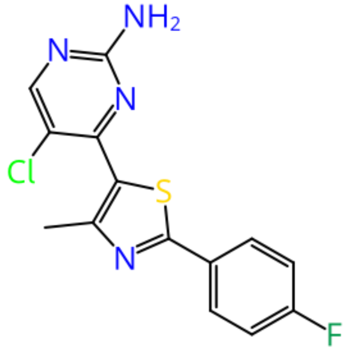
395	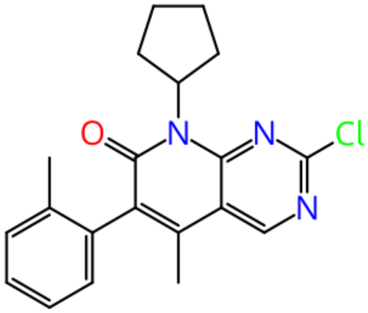	396	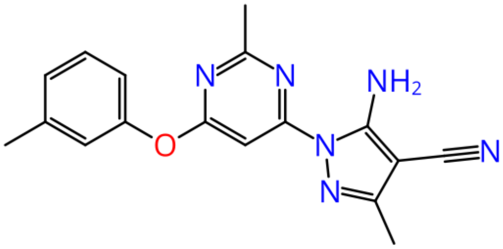
397	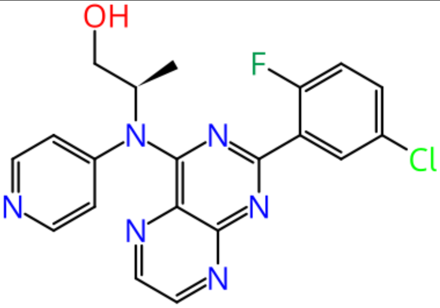	398	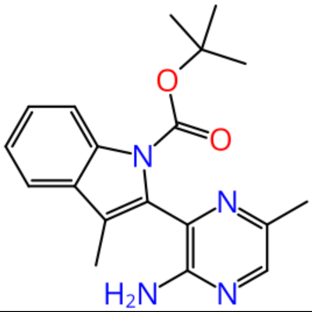

399	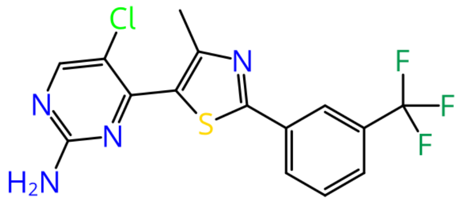	400	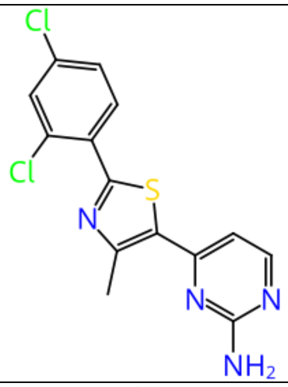
401	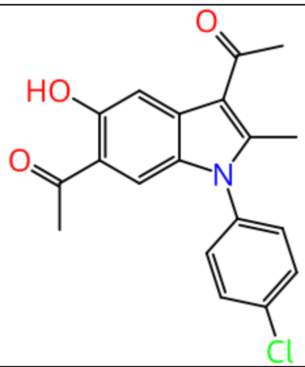	402	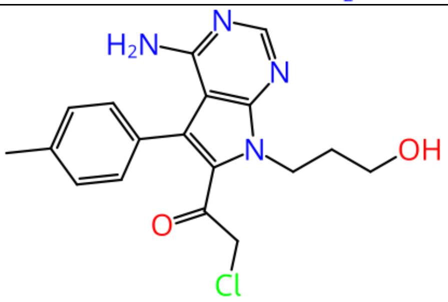
403	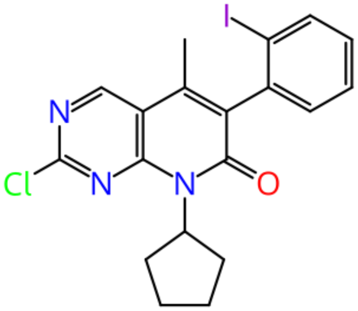	404	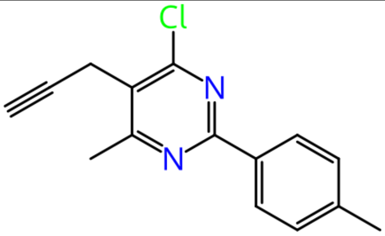
405	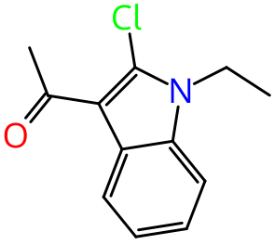	406	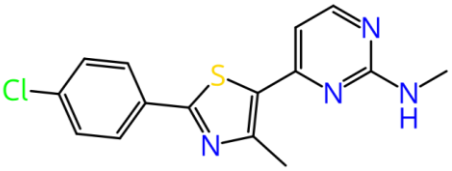
407	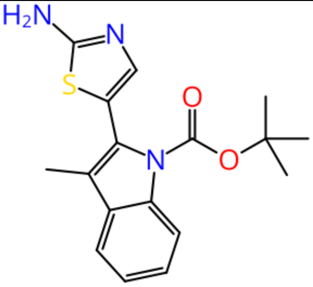	408	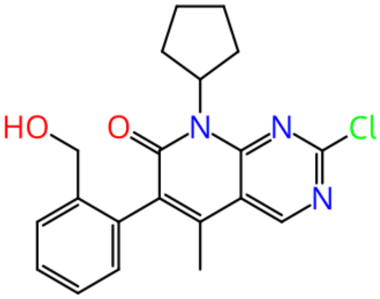

409	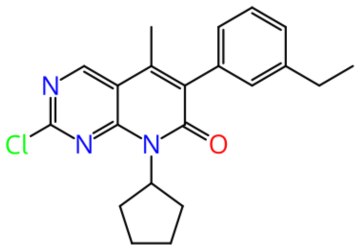	410	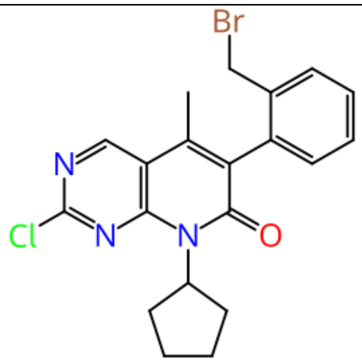
411	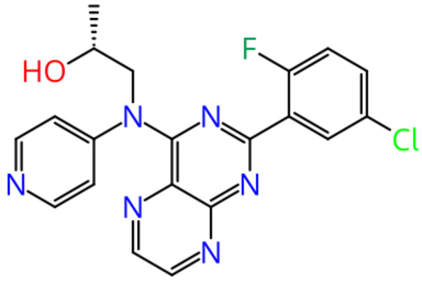	412	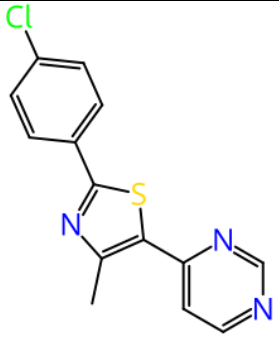
413	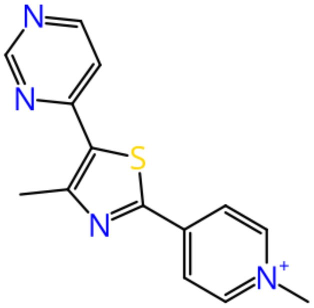	414	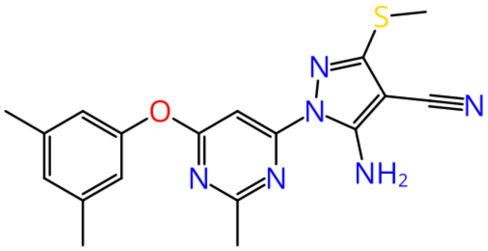
415	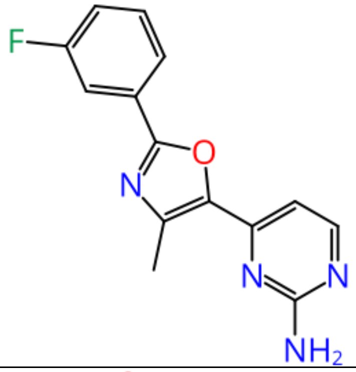	416	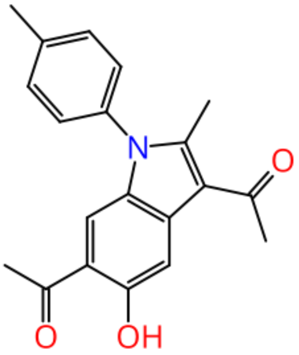
417	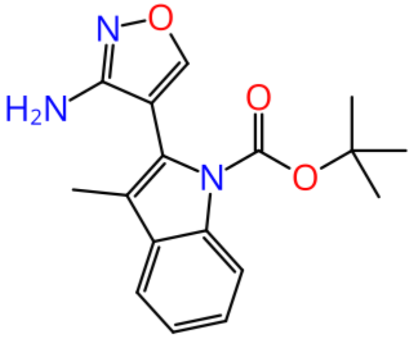	418	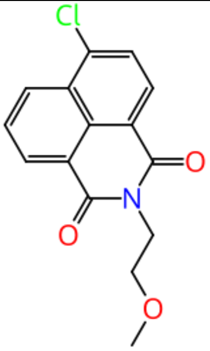

419	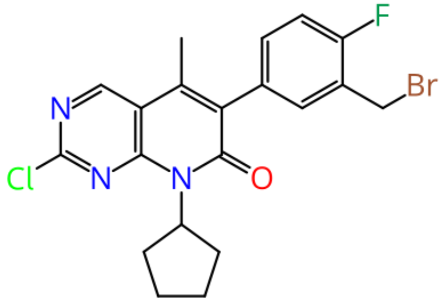	420	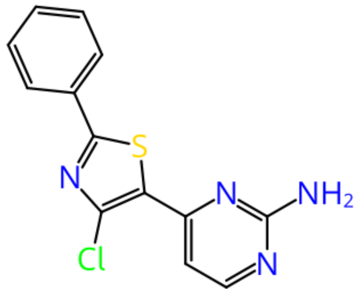
421	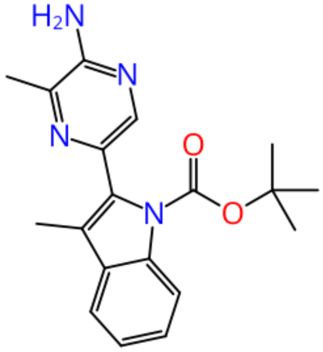	422	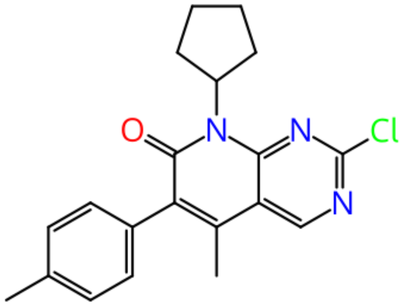
423	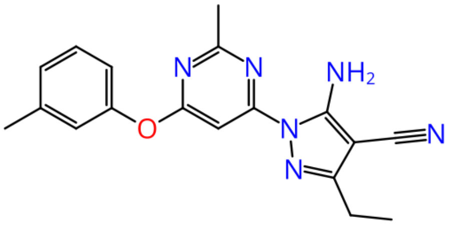	424	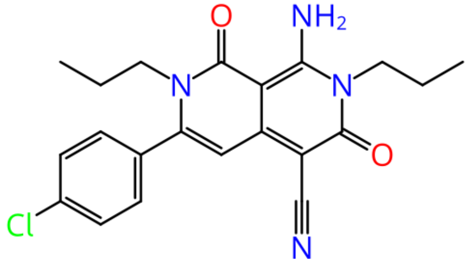
425	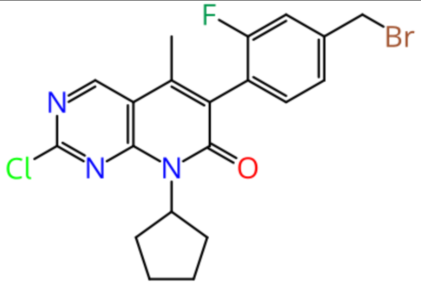	426	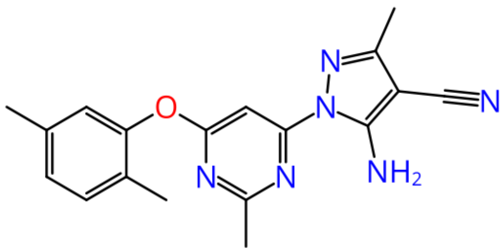
427	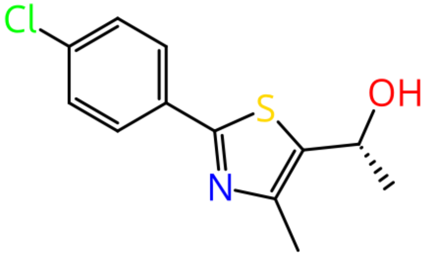	428	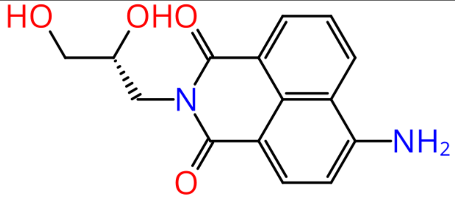
429	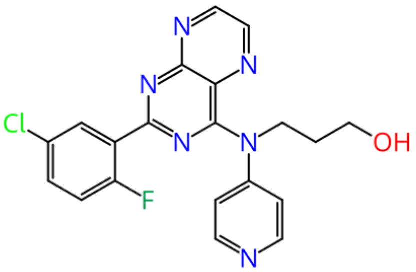	430	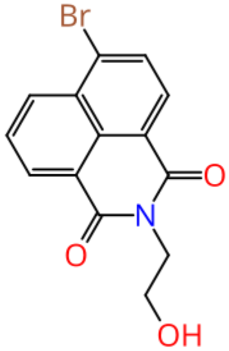

431	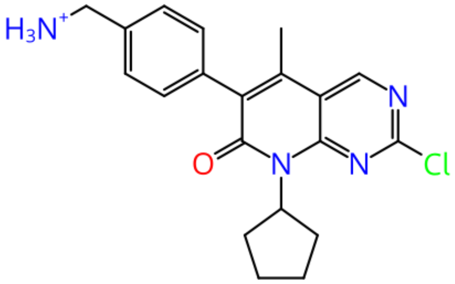	432	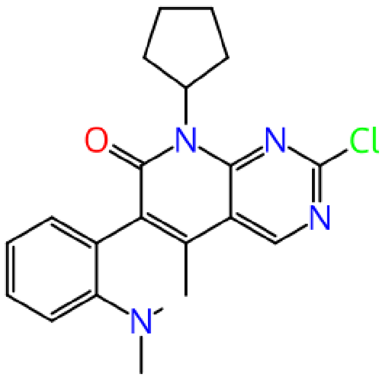
433	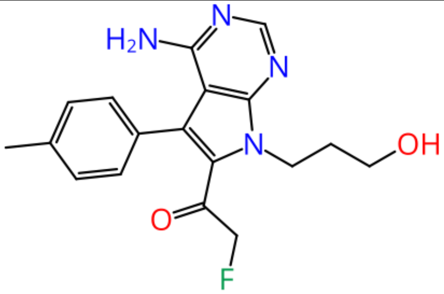	434	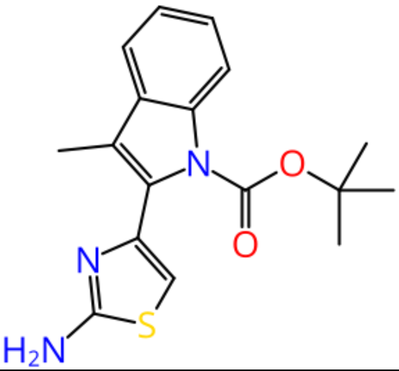
435	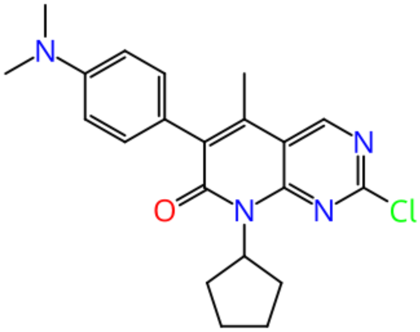	436	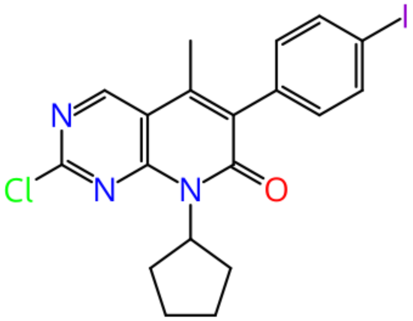
437	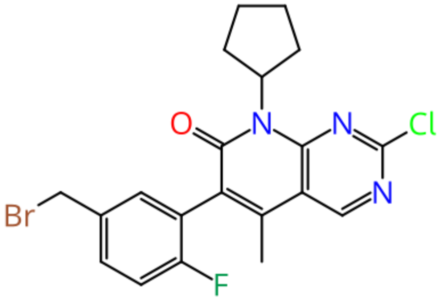	438	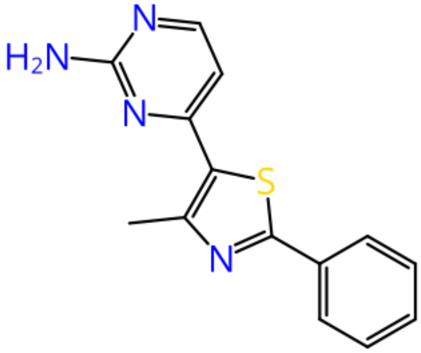
439	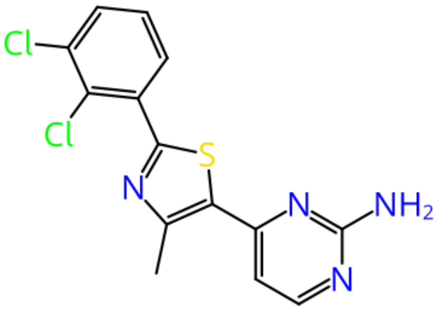	440	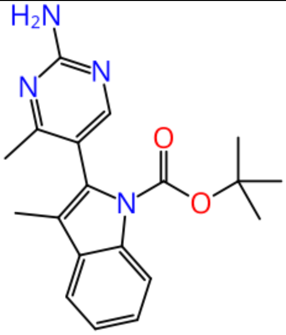

441	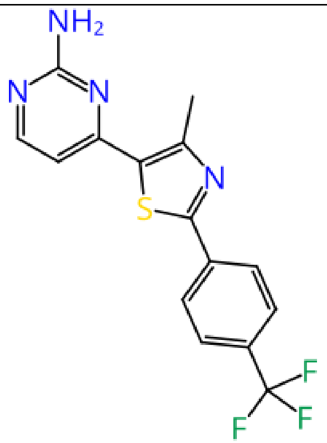	442	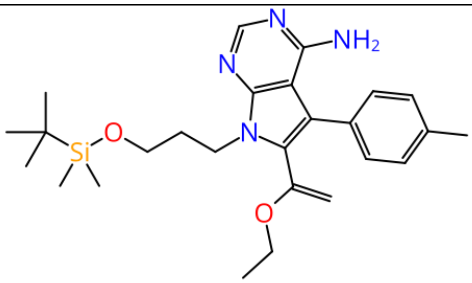
443	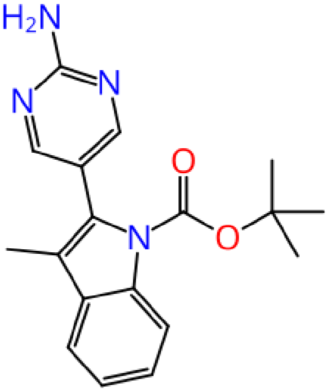	444	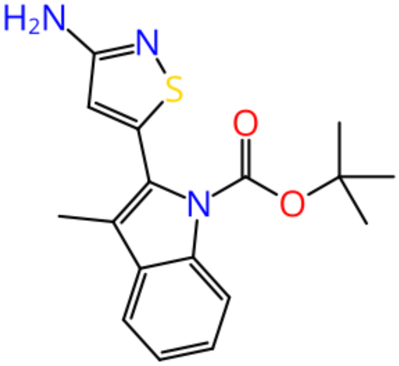
445	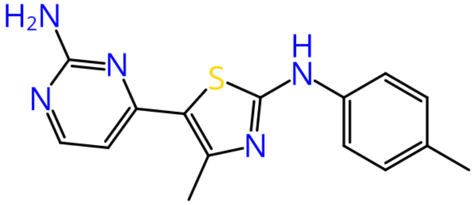	446	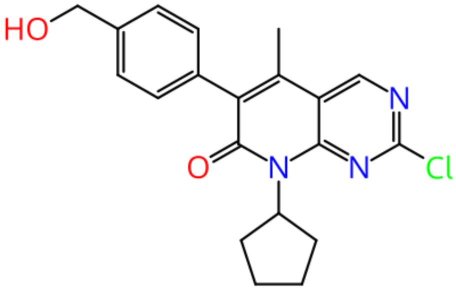
447	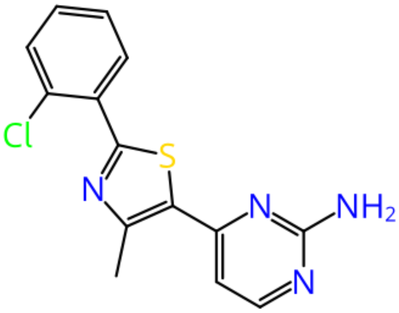	448	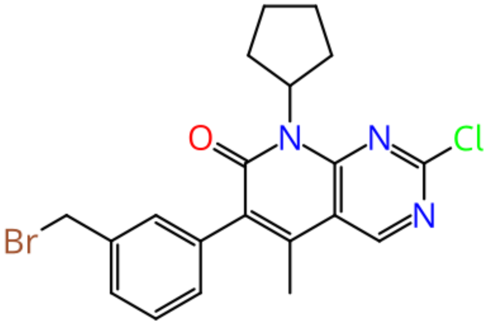
449	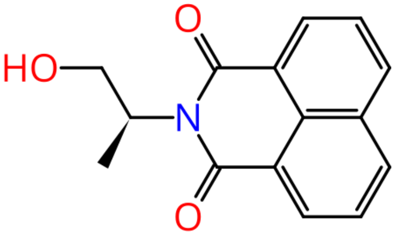	450	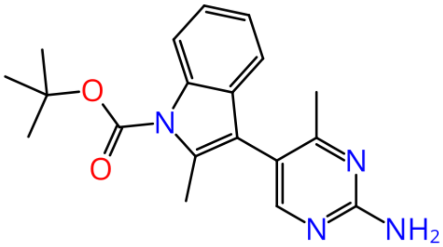

451	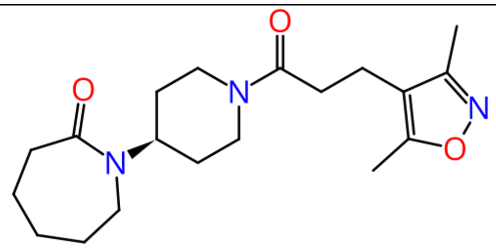	452	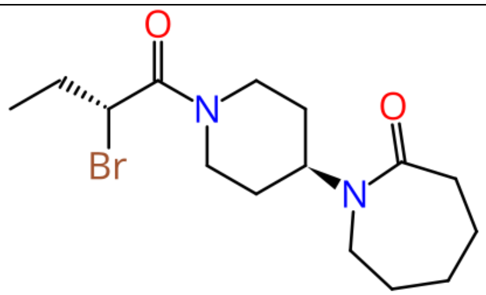
453	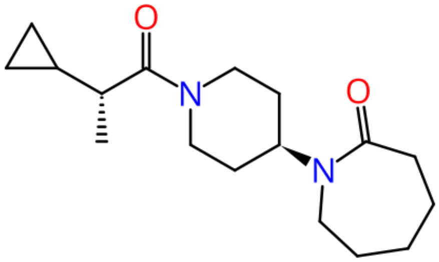	454	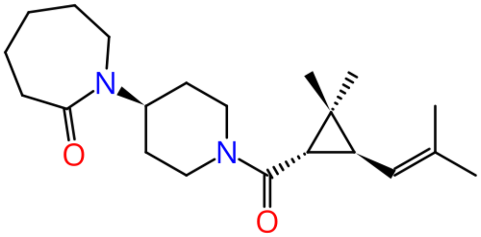
455	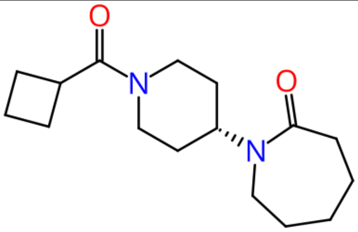	456	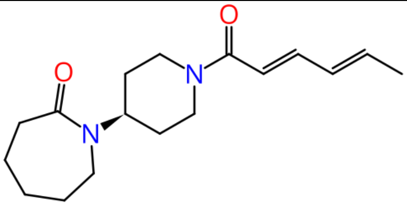
457	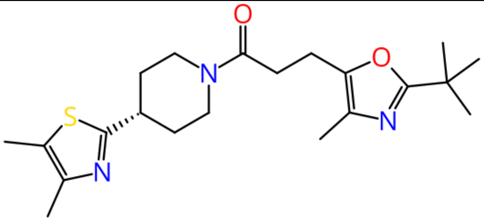	458	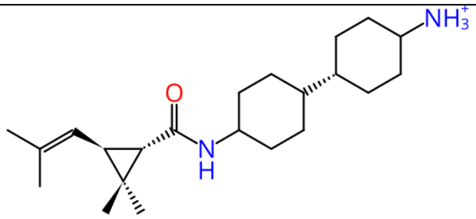
459	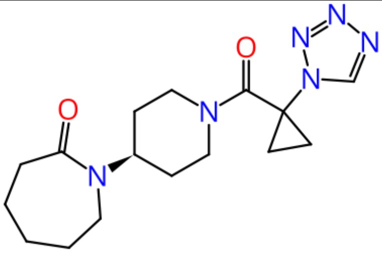	460	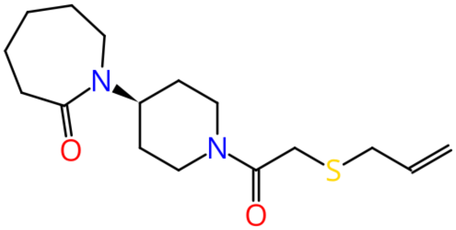
461	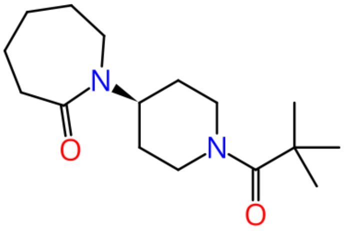	462	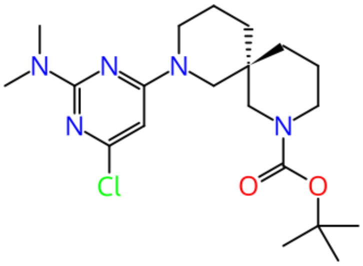
463	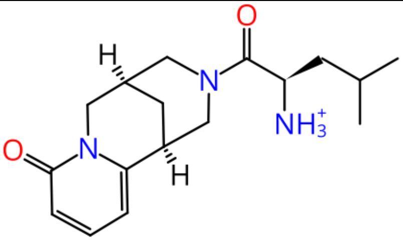	464	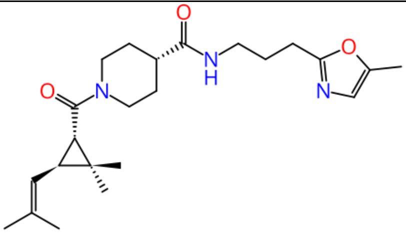

465	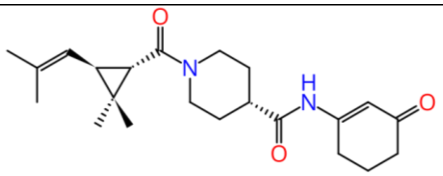	466	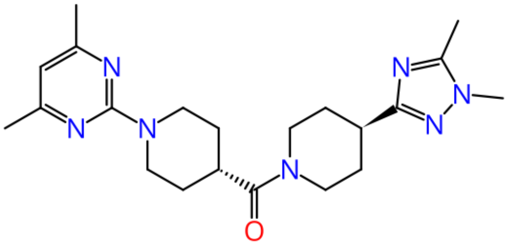
467	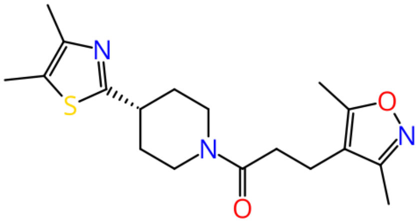	468	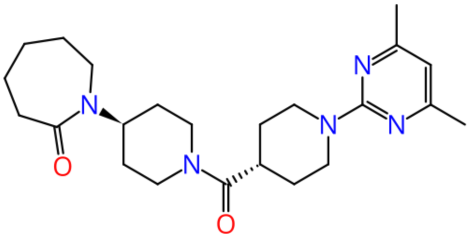
469	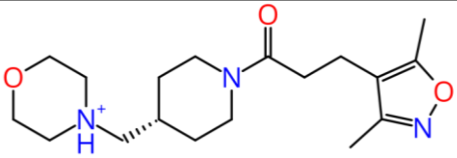	470	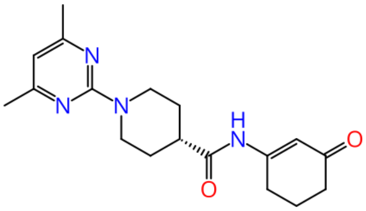
471	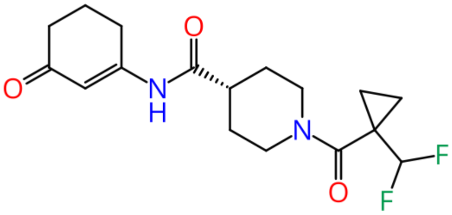	472	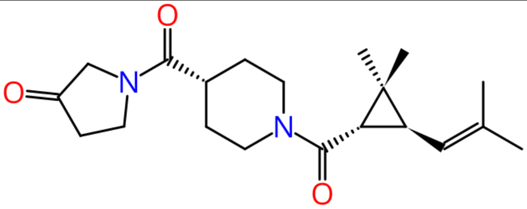
473	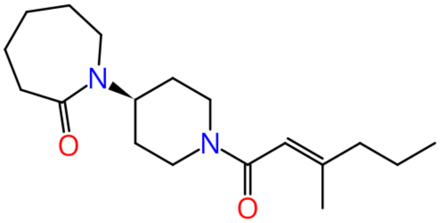	474	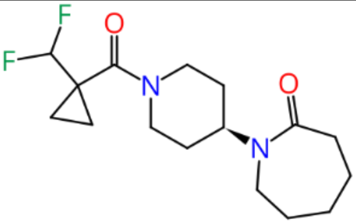
475	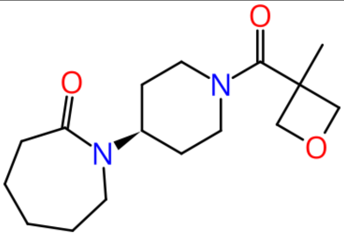	476	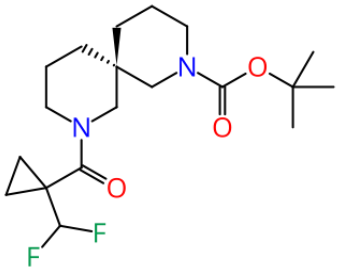
477	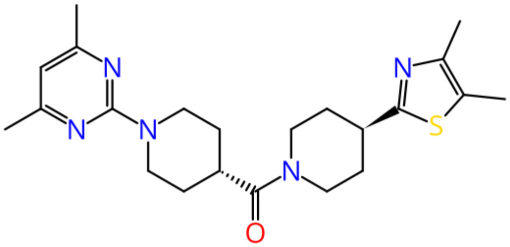	478	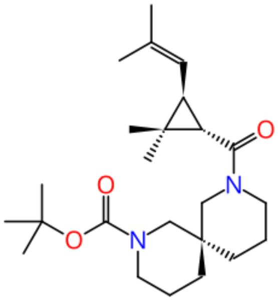

479	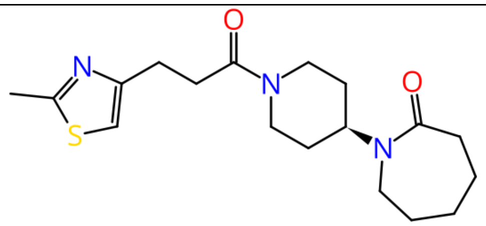	480	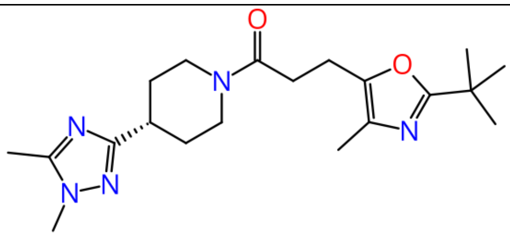
481	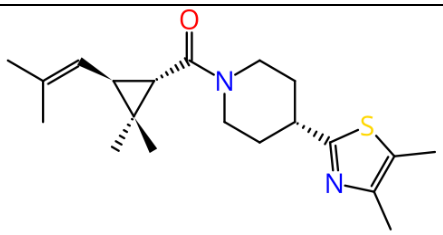	482	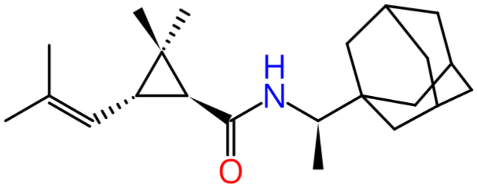
483	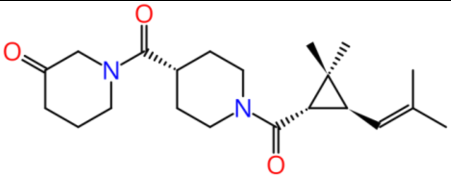	484	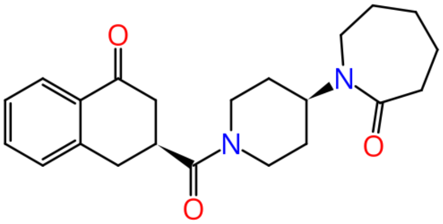
485	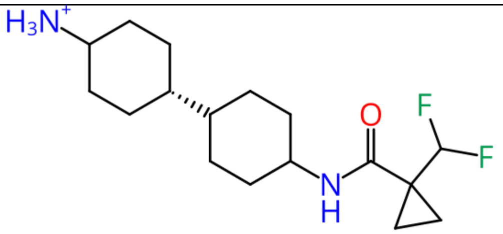	486	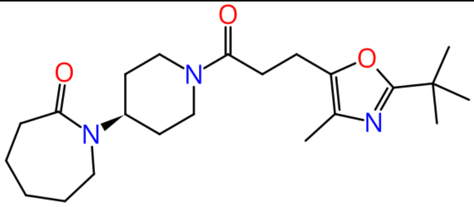
487	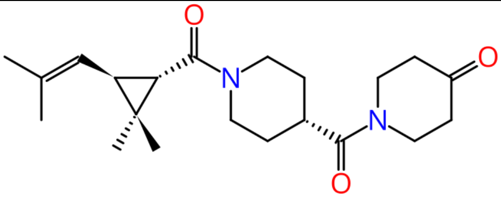	488	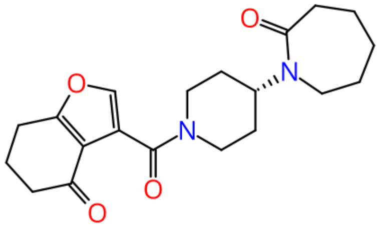
489	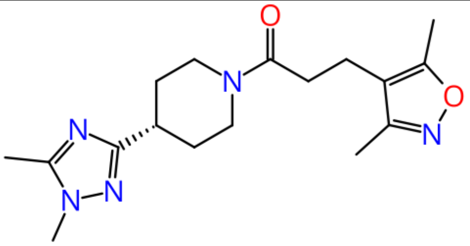	490	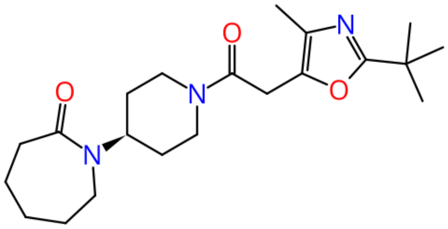
491	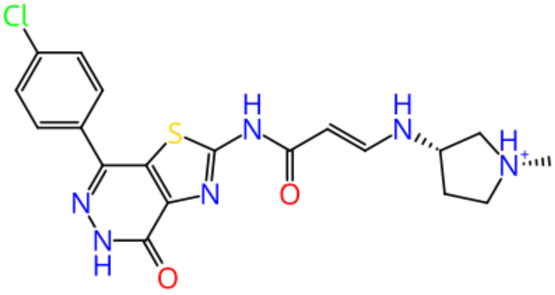	492	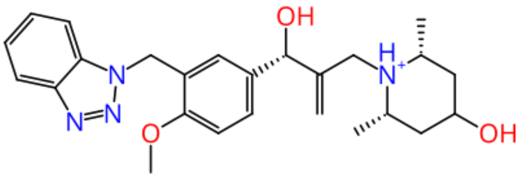

493	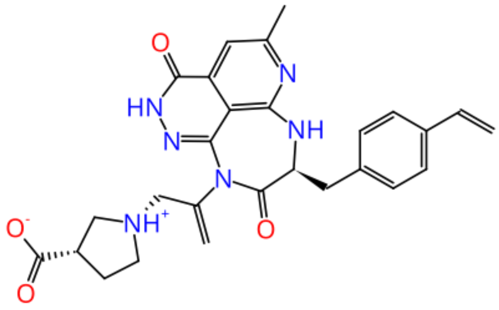	494	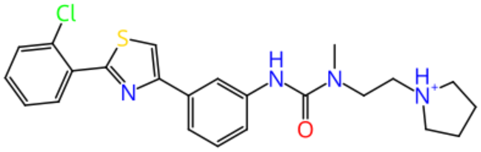
495	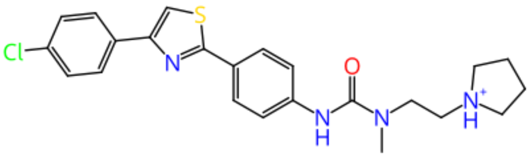	496	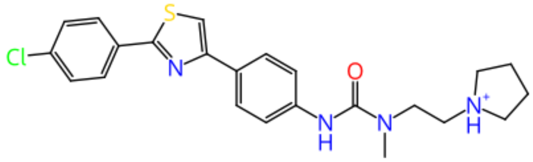
497	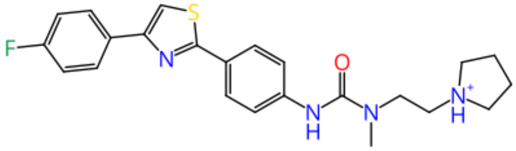	498	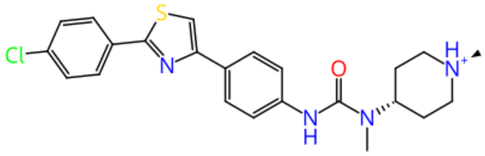
499	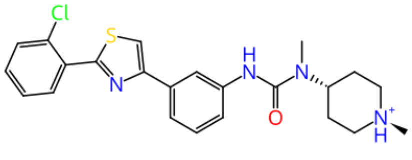	500	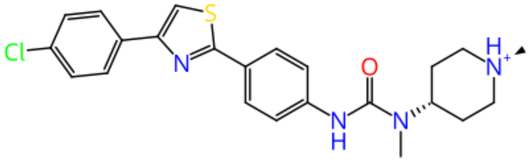
501	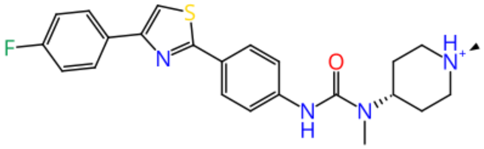	502	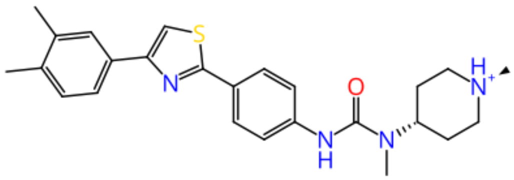

503	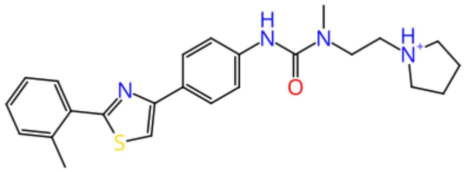	504	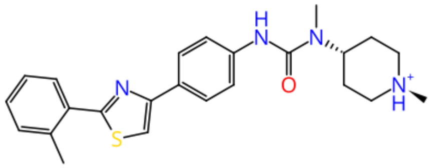
505	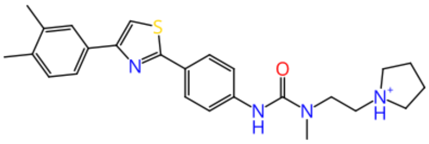	506	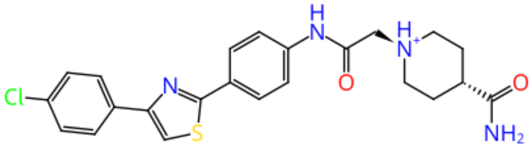
507	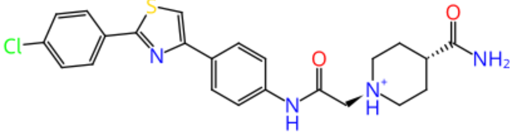	508	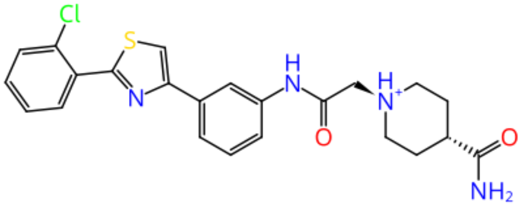
509	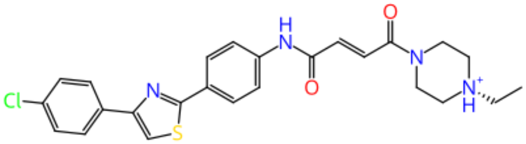	510	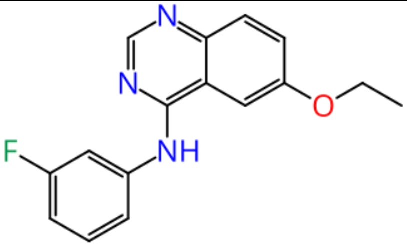
511	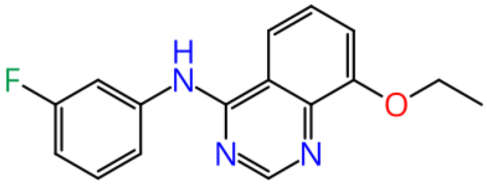	512	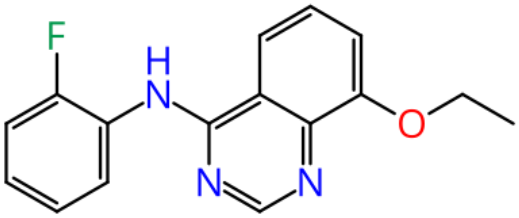
513	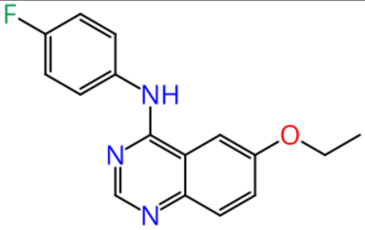	514	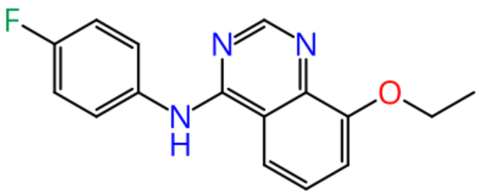

515	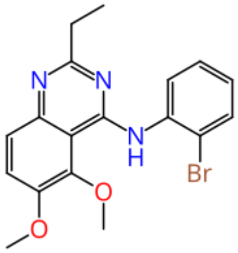	516	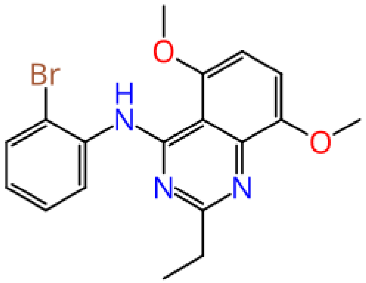
517	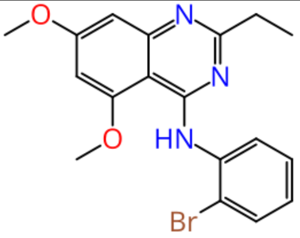	518	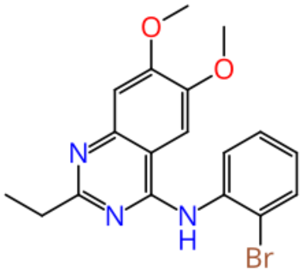
519	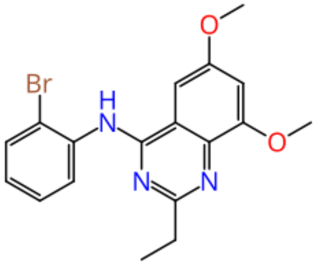	520	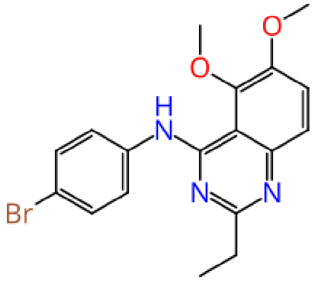
521	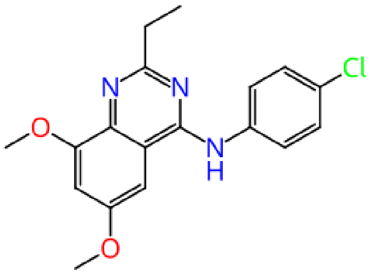	522	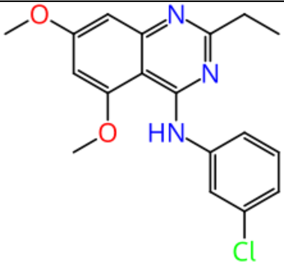
523	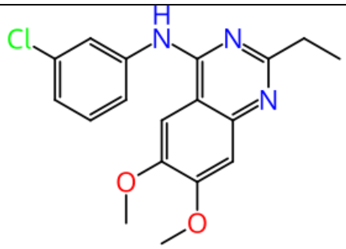	524	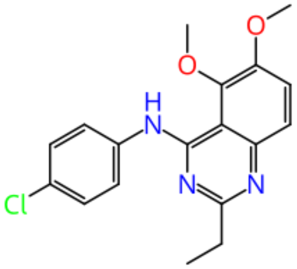
525	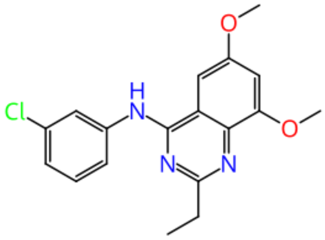	526	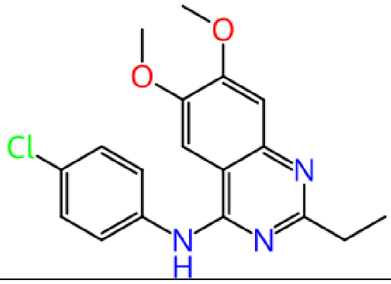

527	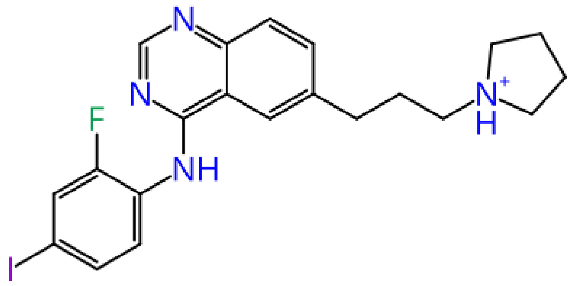	528	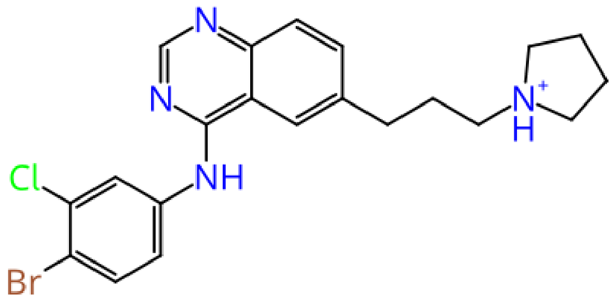
529	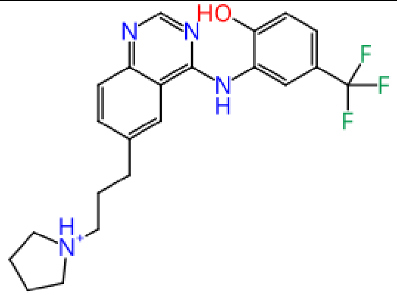	530	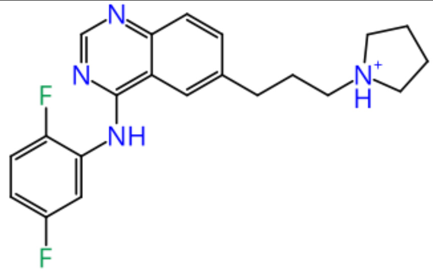
531	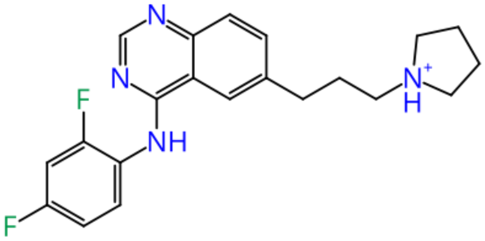	532	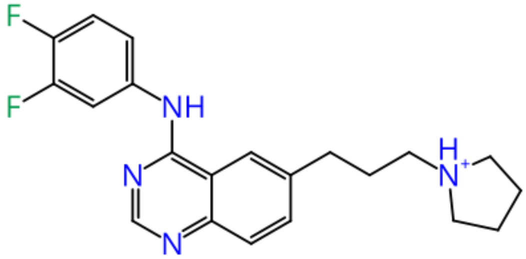
533	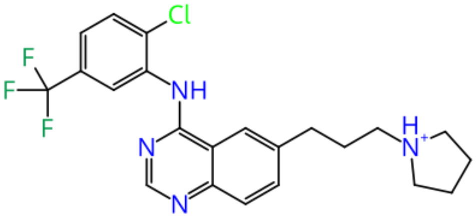	534	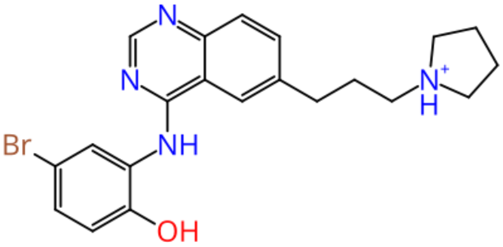
535	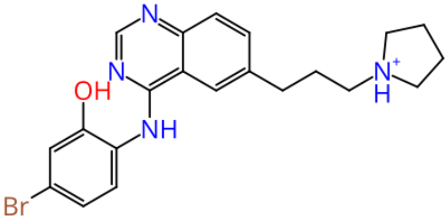	536	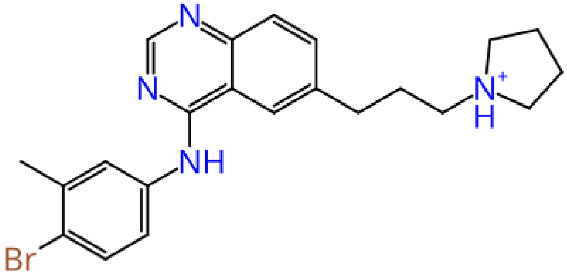
537	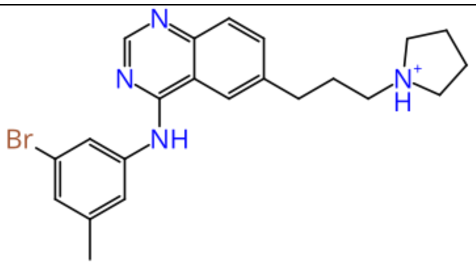	538	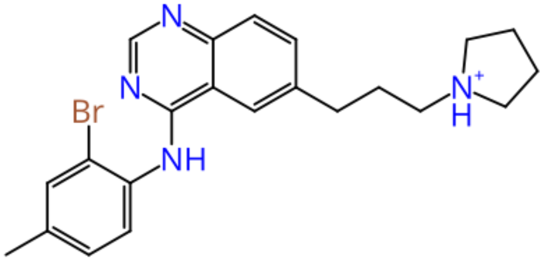

539	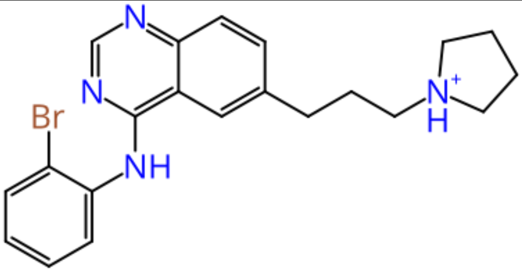	540	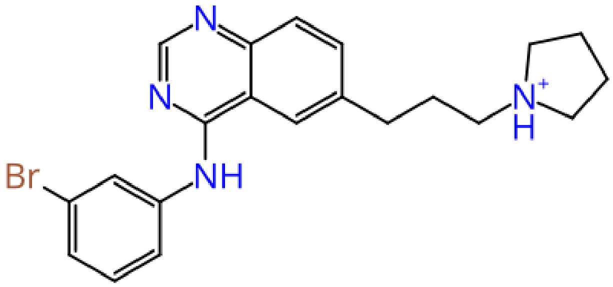
541	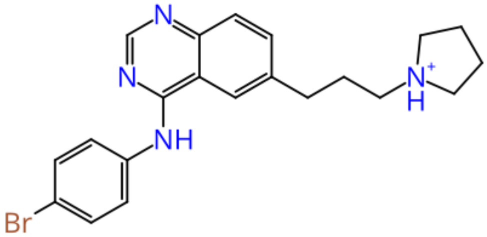	542	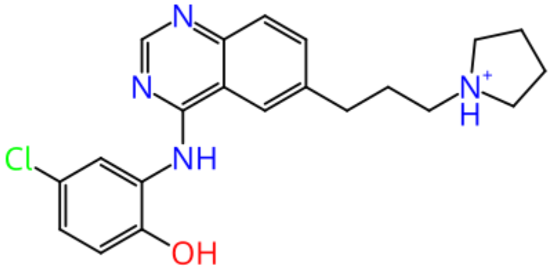
543	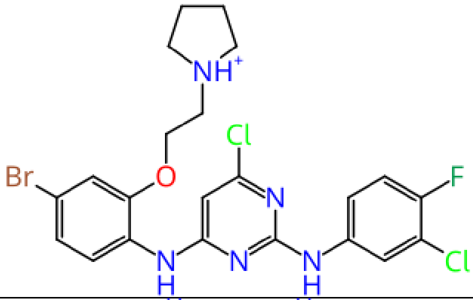	544	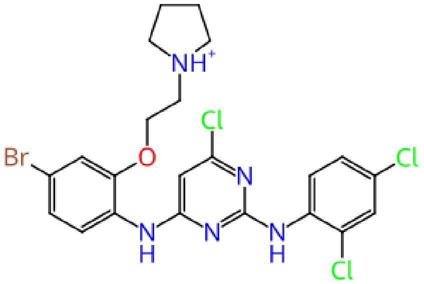
545	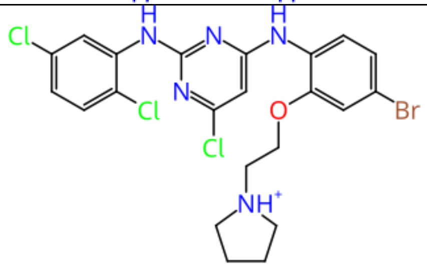	546	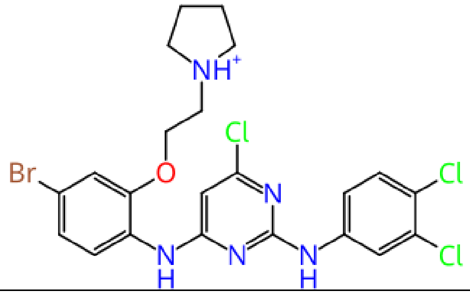
547	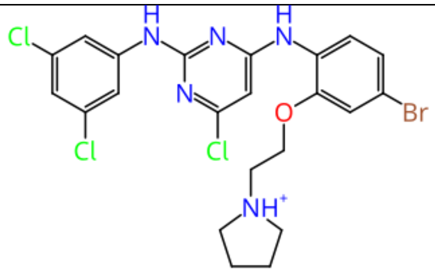	548	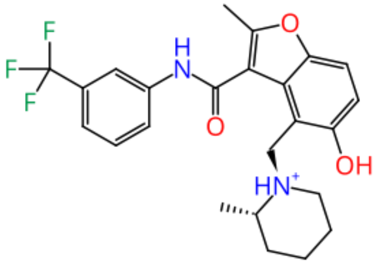
549	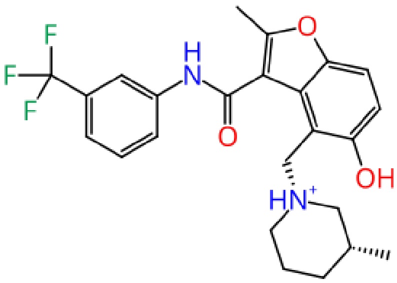	550	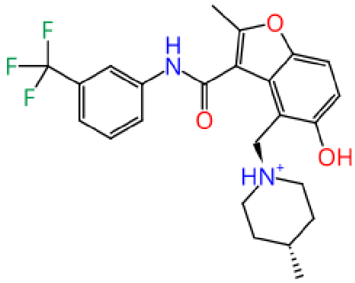

551	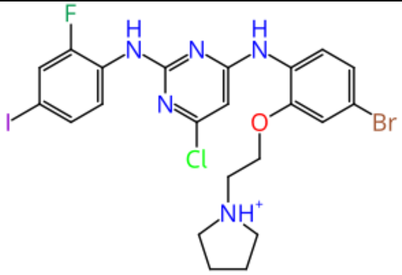	552	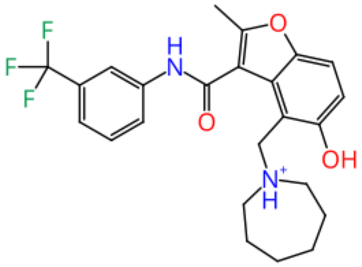
553	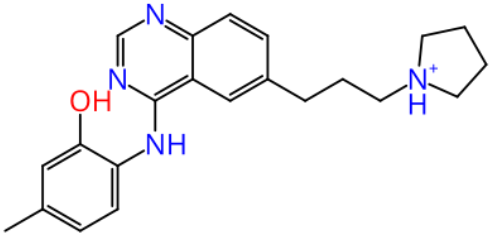	554	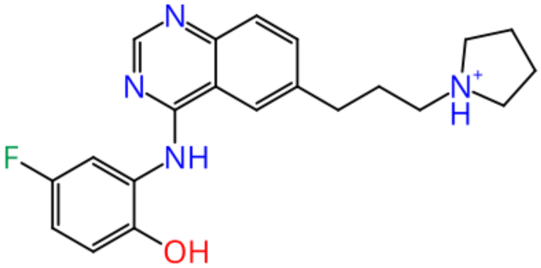
555	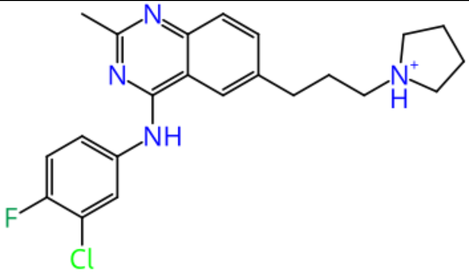	556	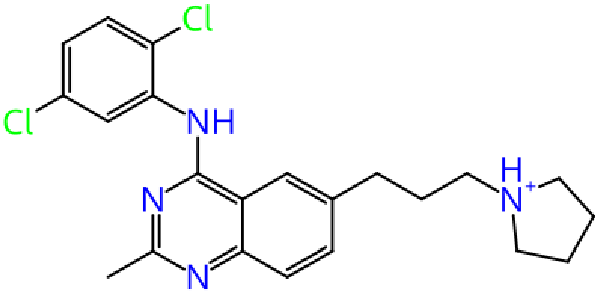
557	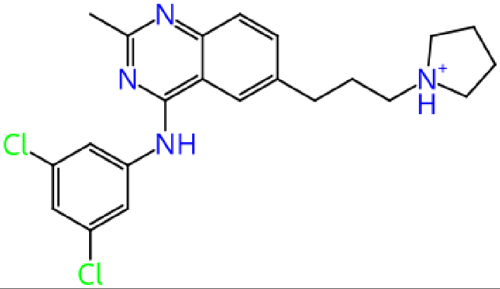	558	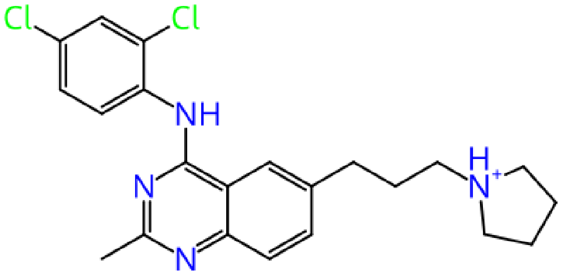
559	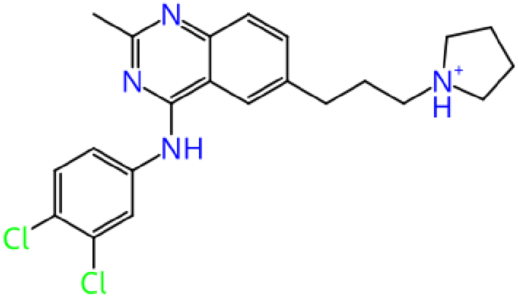	560	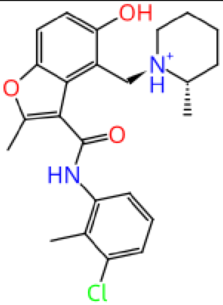
561	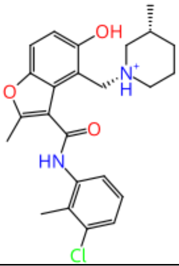	562	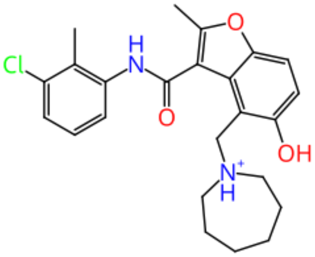

563	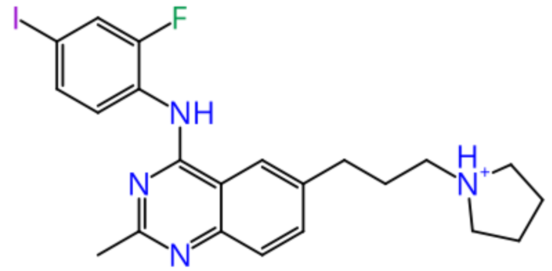	564	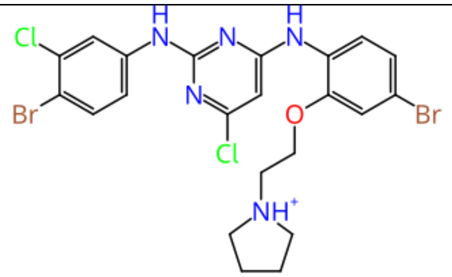
565	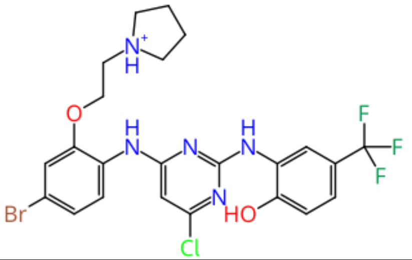	566	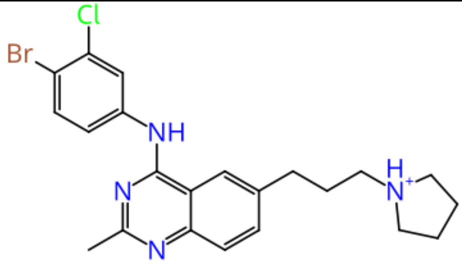
567	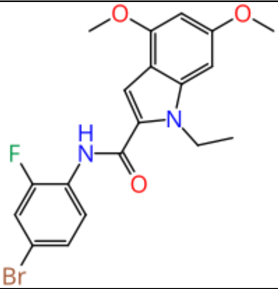	568	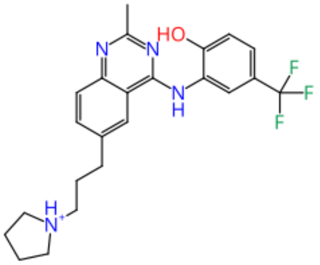
569	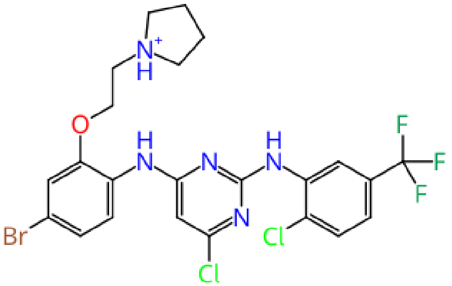	570	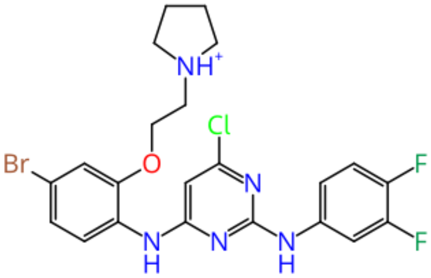
571	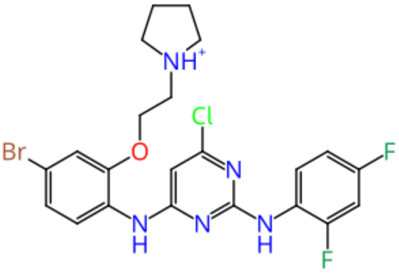	572	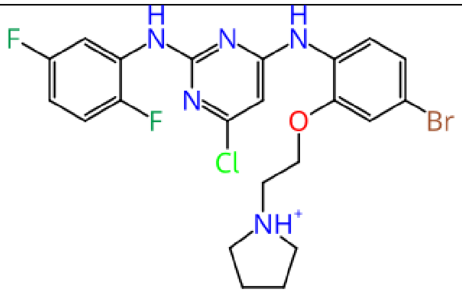
573	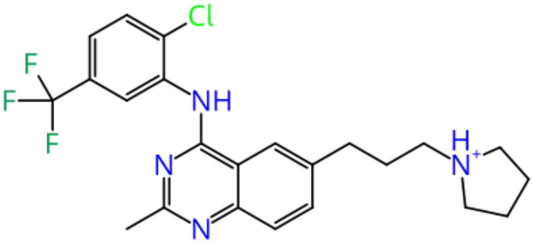	574	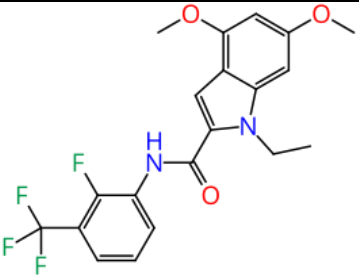

575	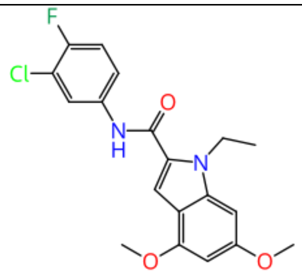	576	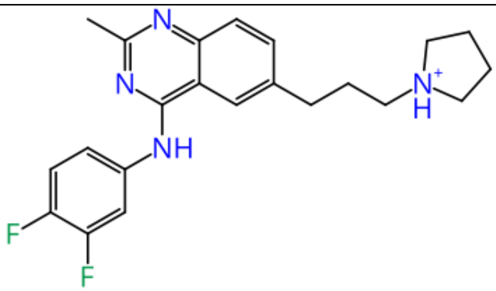
577	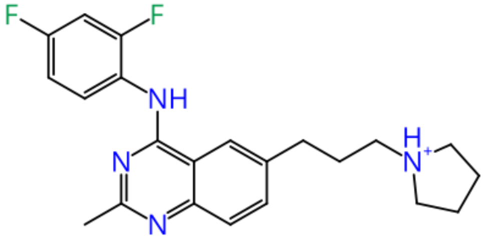	578	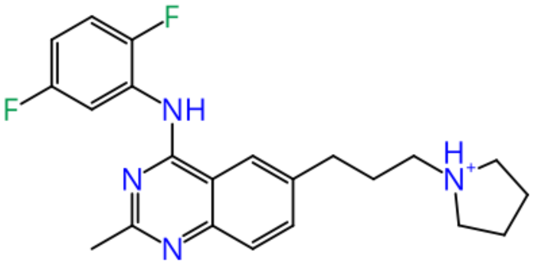
579	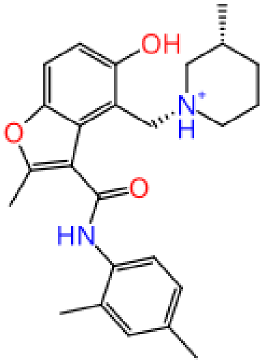	580	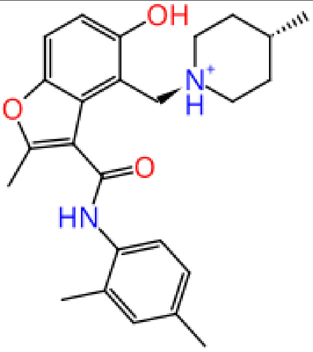
581	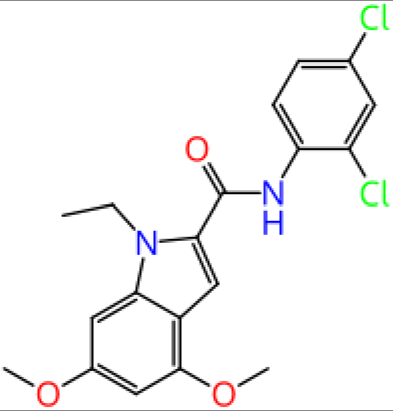	582	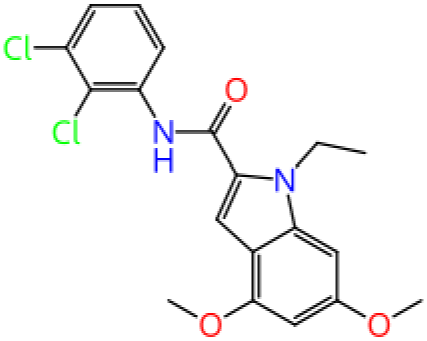
583	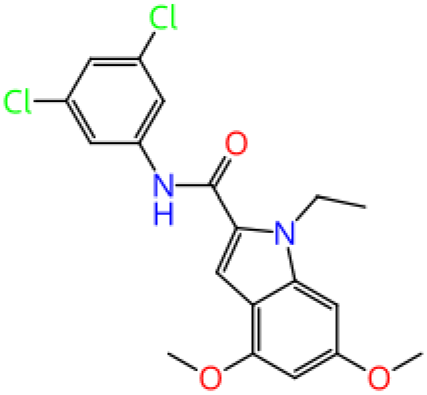	584	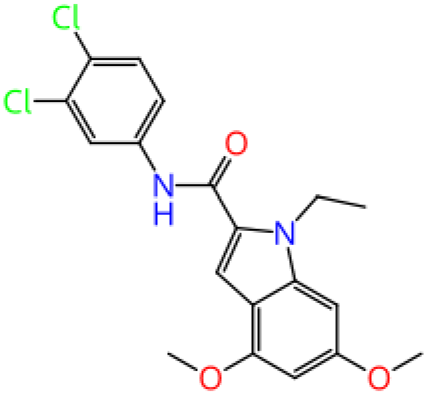

585	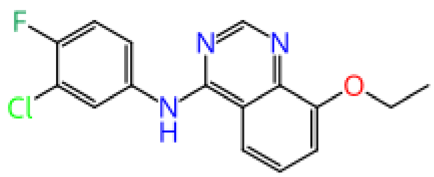	586	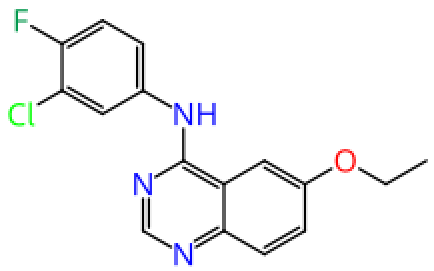
587	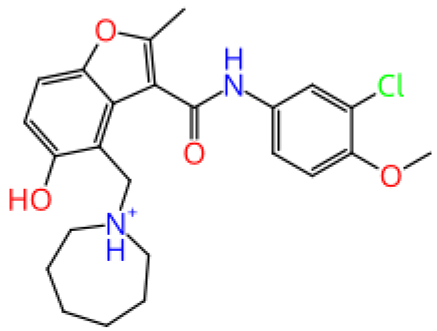	588	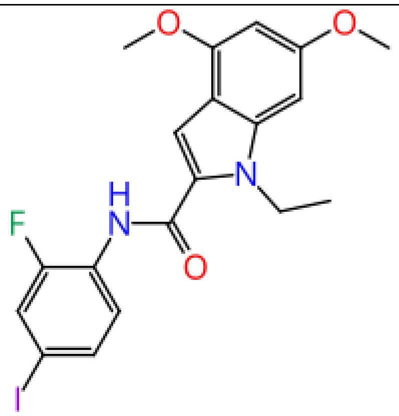
589	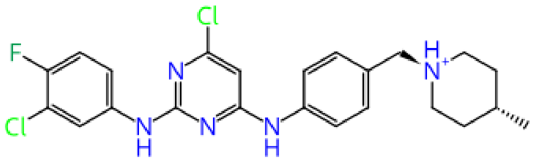	590	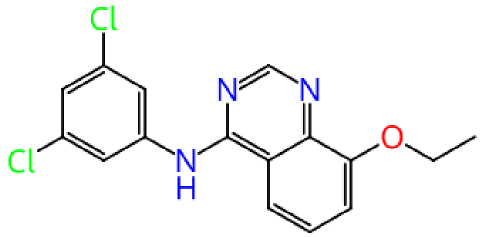
591	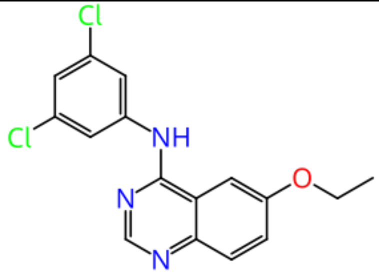	592	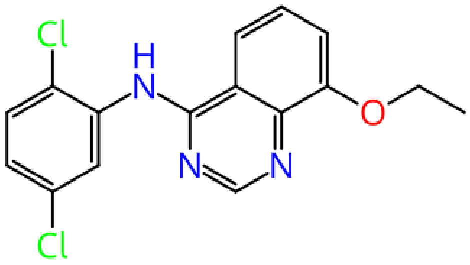
593	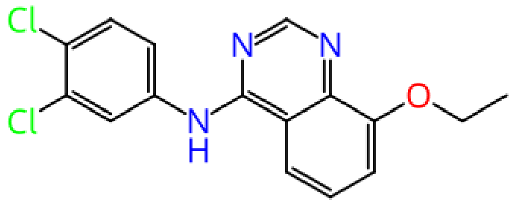	594	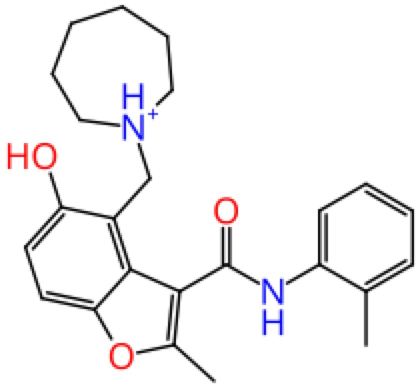

595	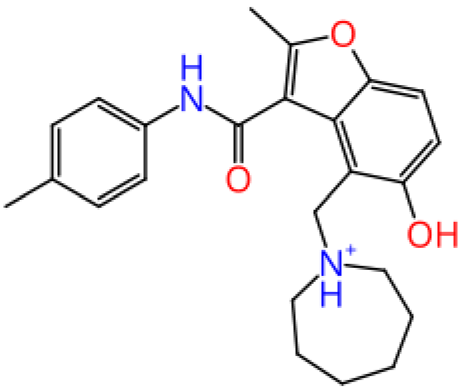	596	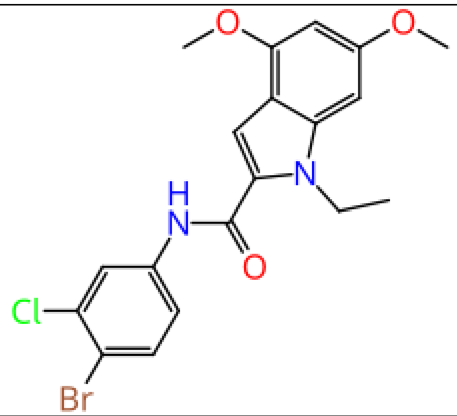
597	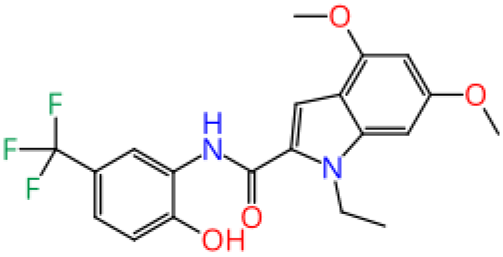	598	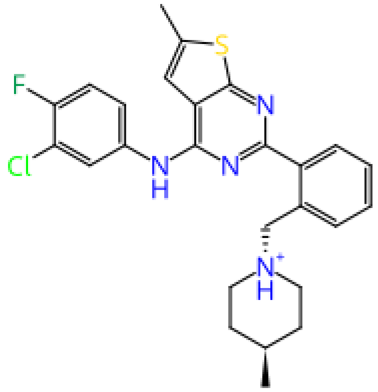
599	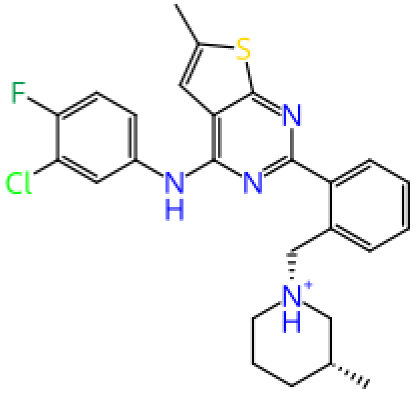	600	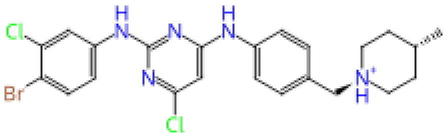
601	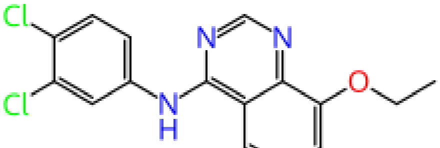	602	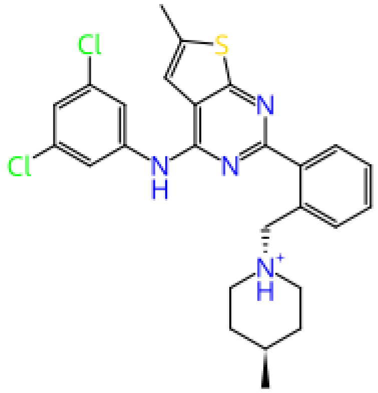

Table S2: EGFR inhibiting compounds generated from molecular editor and inspirator mode of SeeSAR via adding and replacing atoms and group of atoms. (The orange highlighted portion of compounds are representing fragments generated by ReCore).

No	Structure of compounds	No	Structure of compounds
2a	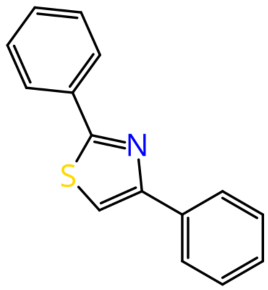	2b	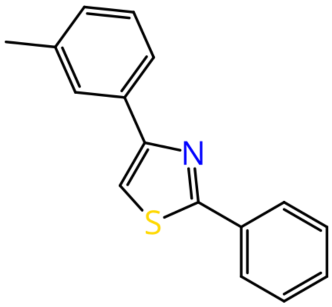
2c	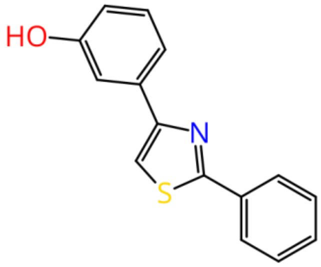	2d	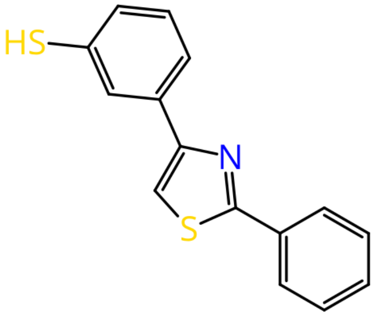
2e	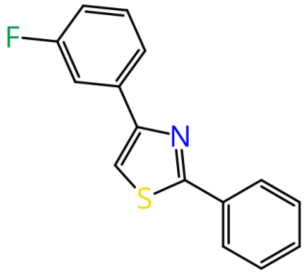	2f	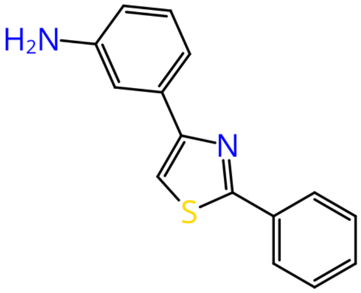
2g	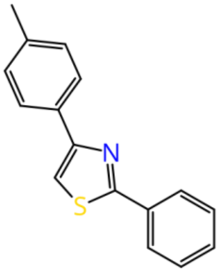	2h	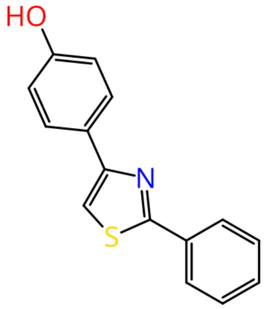

2i	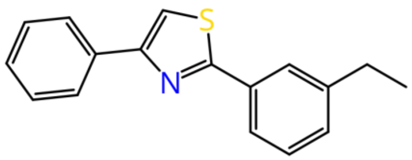	2j	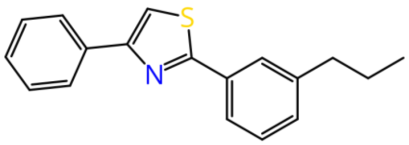
2k	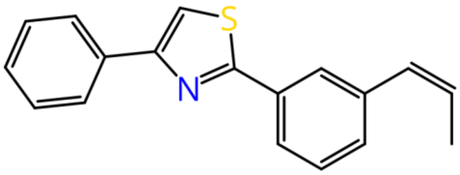	2l	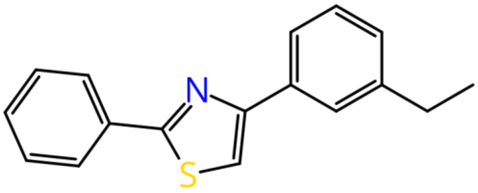
2m	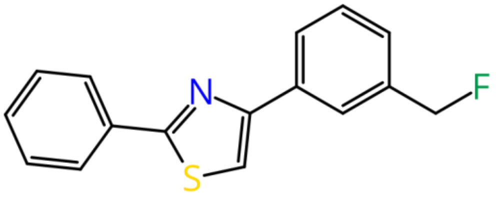	2n	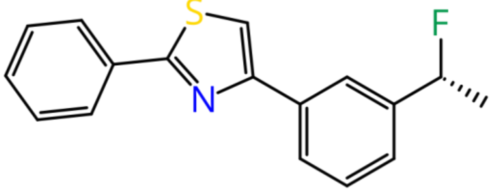
2o	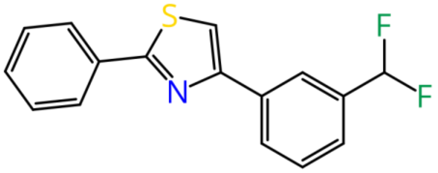	2p	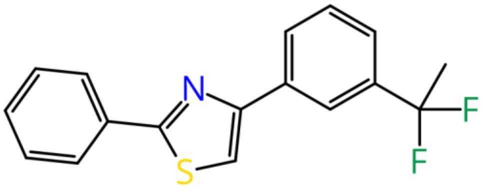
2q	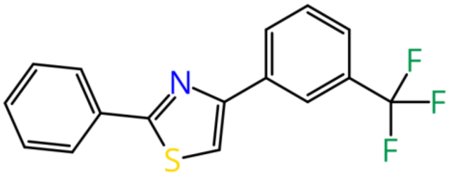	2r	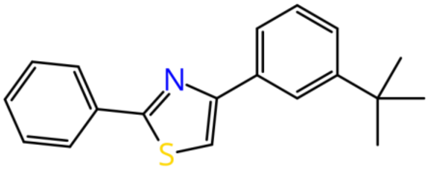
2s	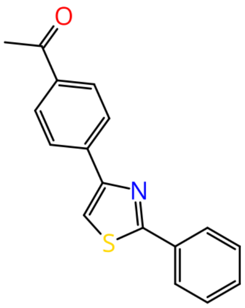	2t	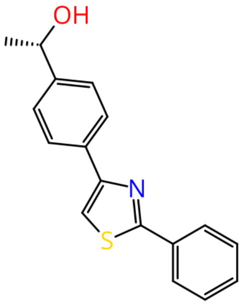

2u	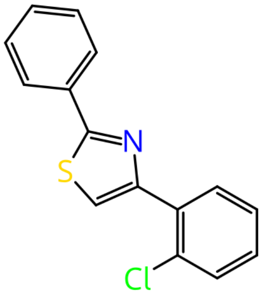	2v	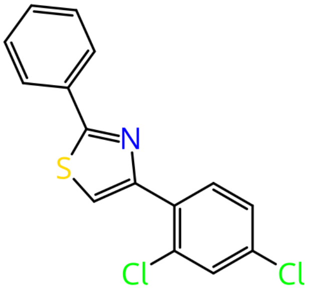
2w	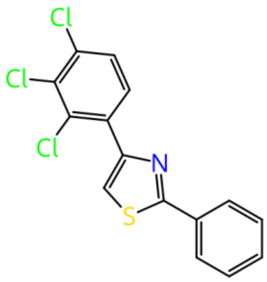	2x	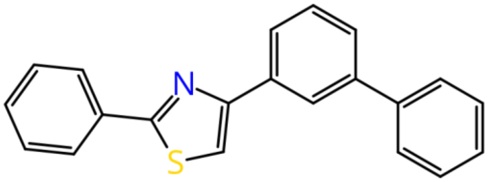
2y	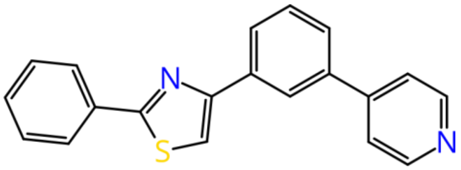	2z	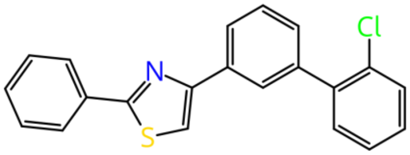
3a	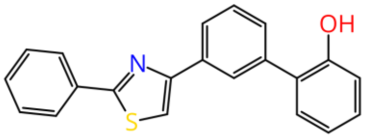	3b	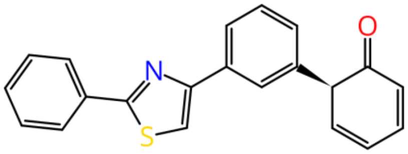
3c	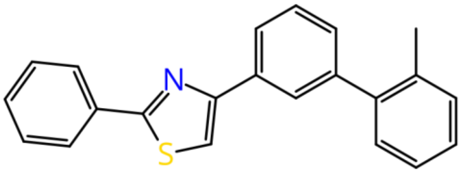	3d	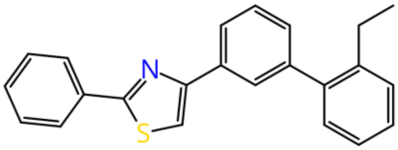
3e	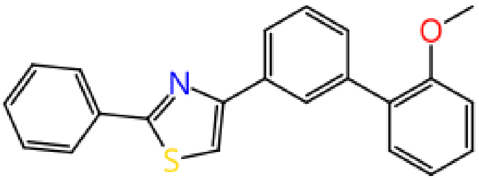	3f	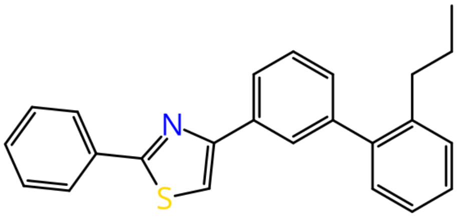

3g	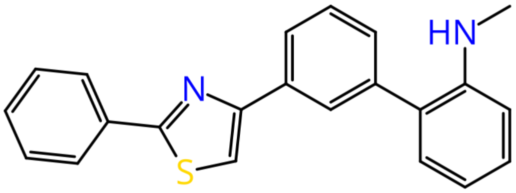	3h	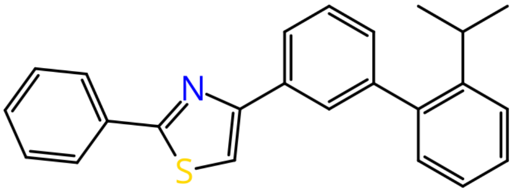
3i	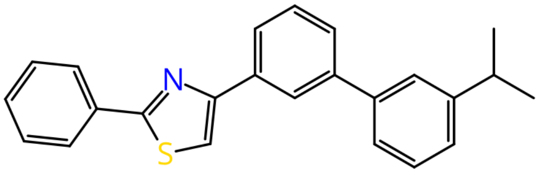	3j	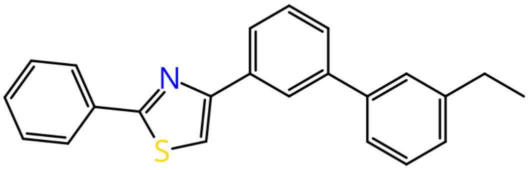
3k	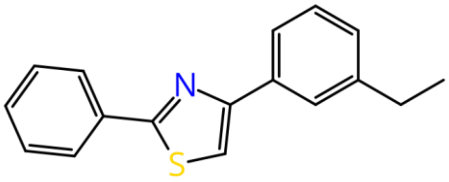	3l	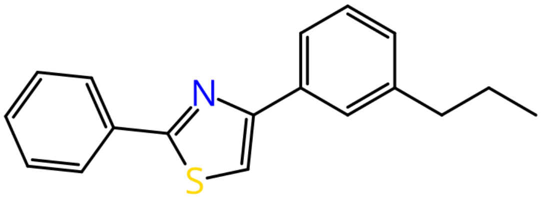
3m	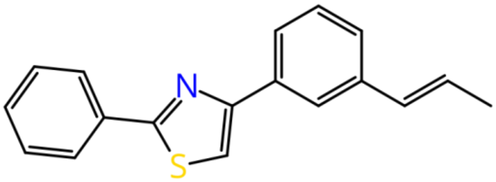	3n	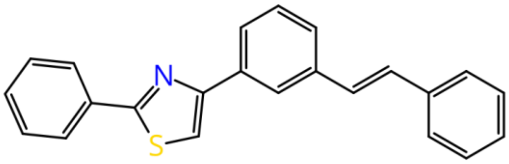
3o	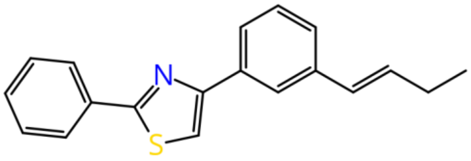	3p	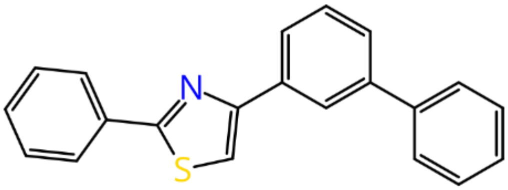
3q	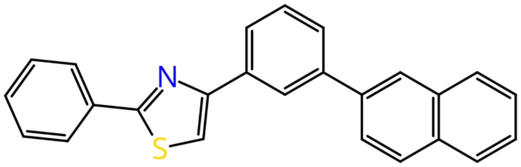	3r	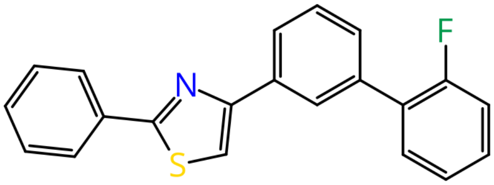
3s	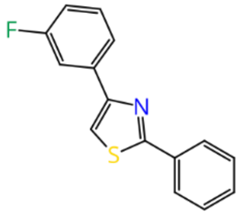	3t	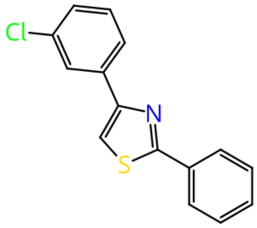
3u	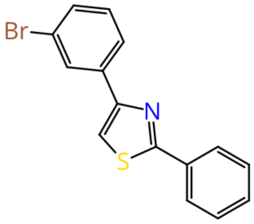	3v	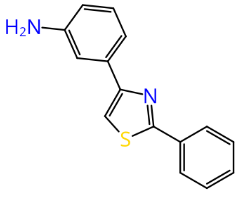

3w	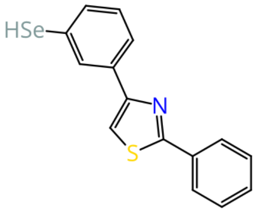	3x	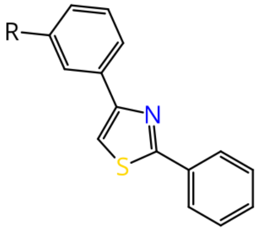
3y	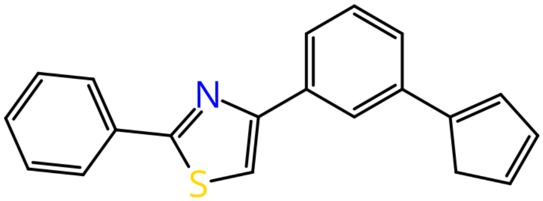	3z	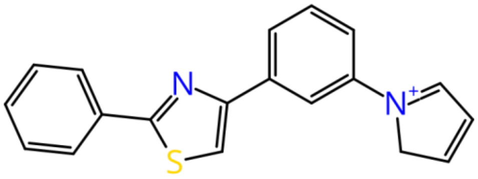
4a	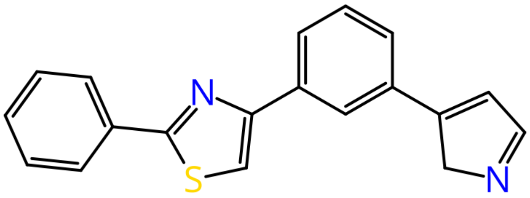	4b	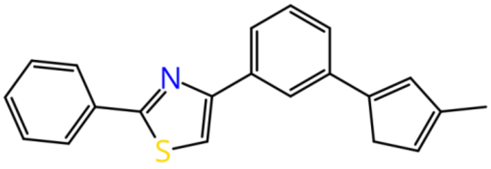
4c	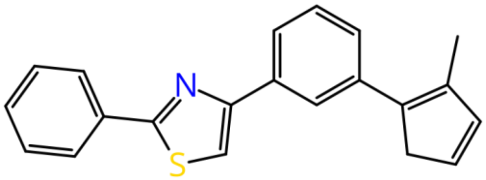	4d	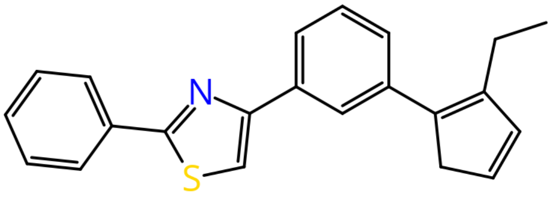
4e	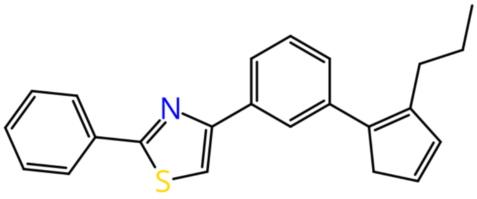	4f	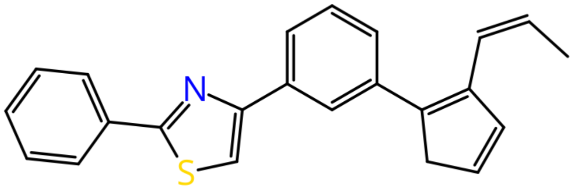
4g	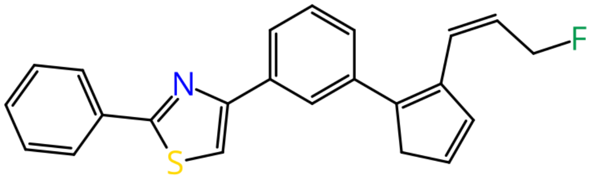	4h	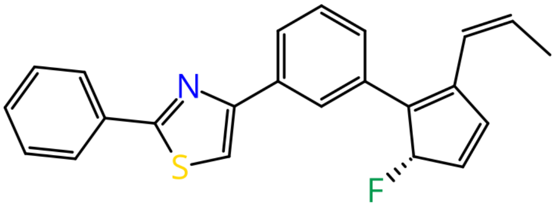
4i	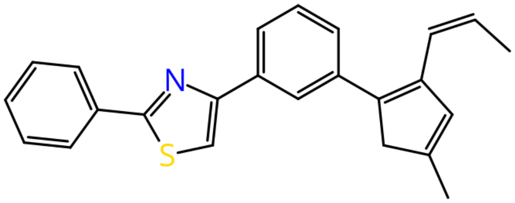	4j	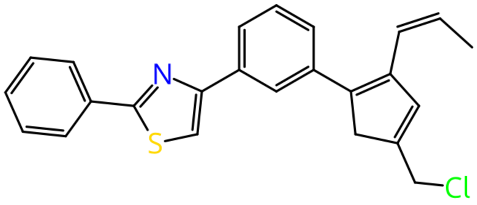

4k	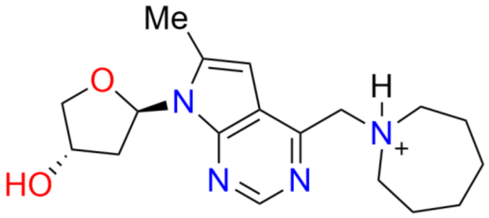	4l	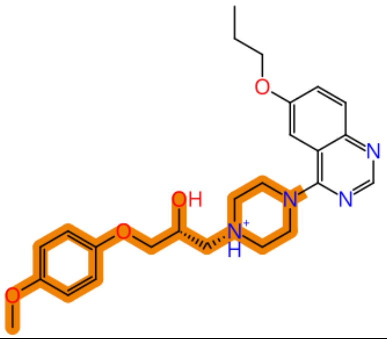
4m	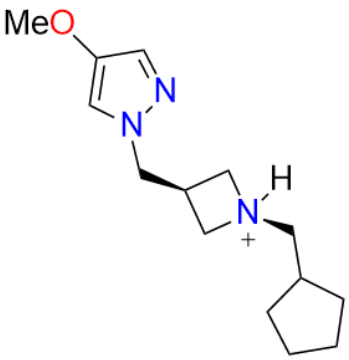	4n	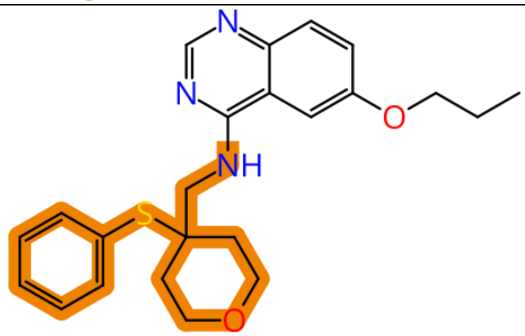
4o	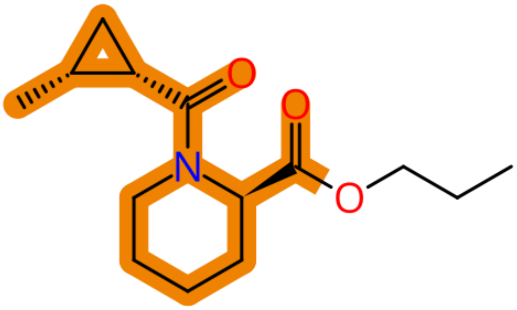	4p	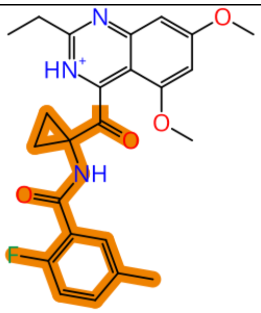
4q	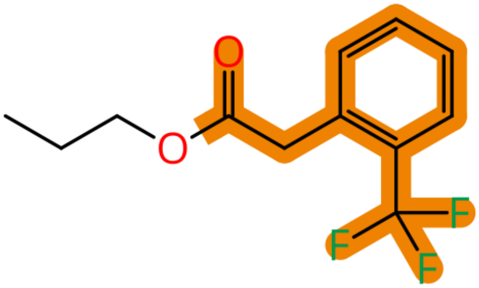	4r	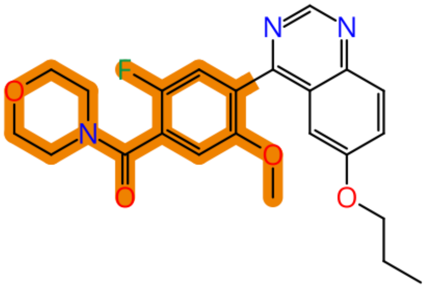
4s	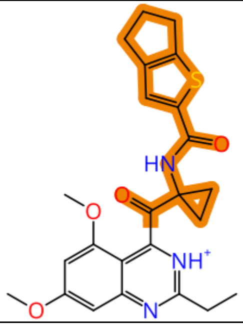	4t	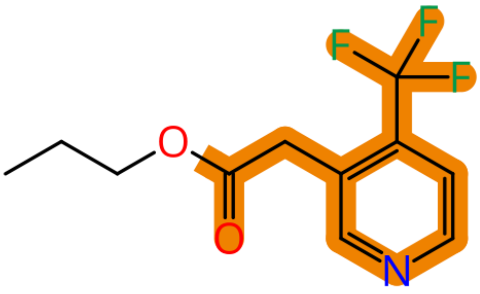

4u	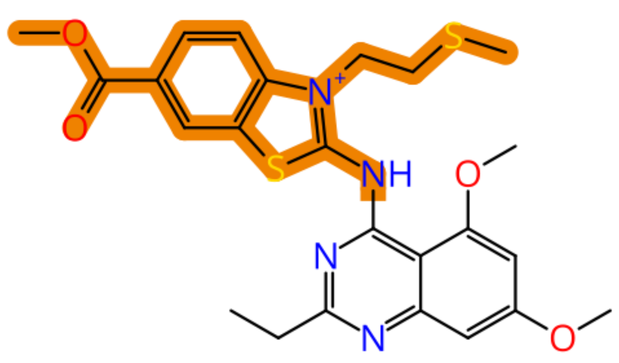	4v	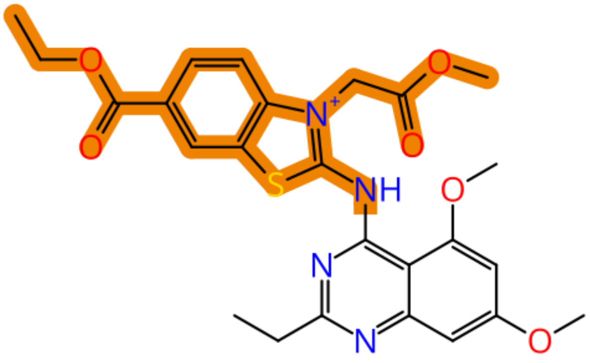
4w	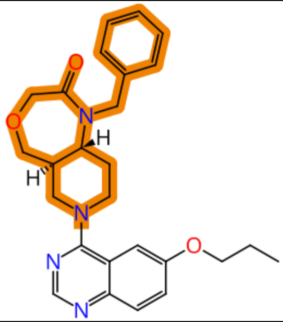	4x	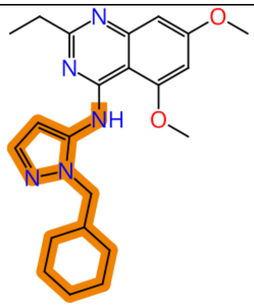
4y	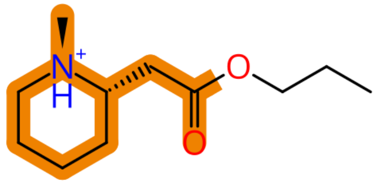	4z	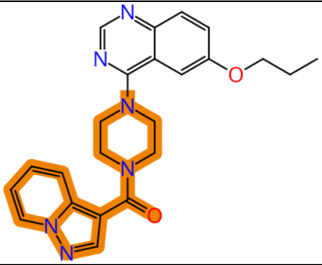
5a	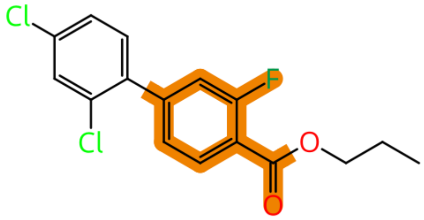	5b	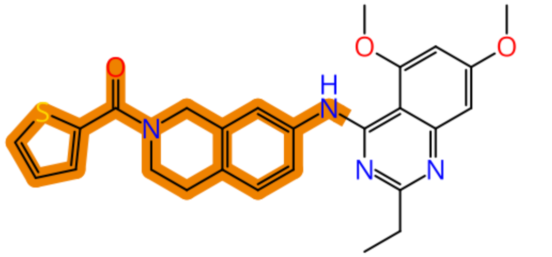
5c	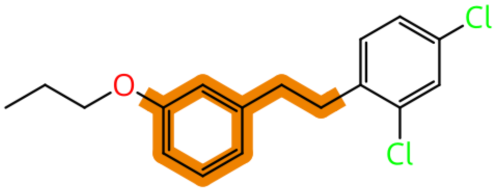	5d	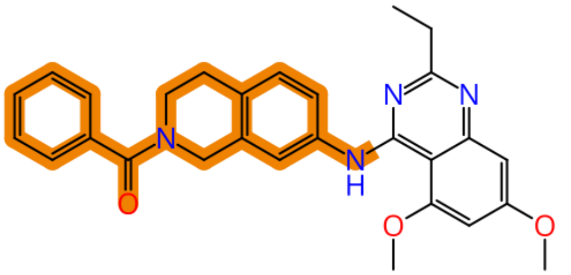

5e	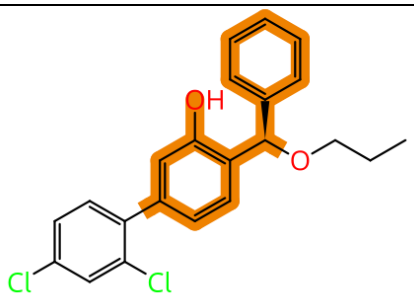	5f	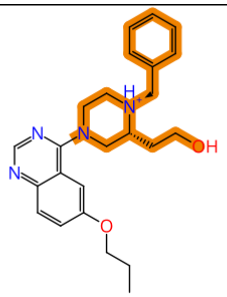
5g	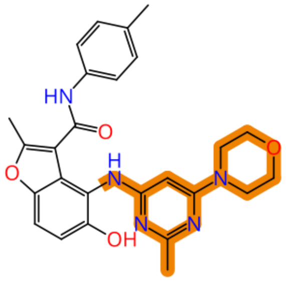	5h	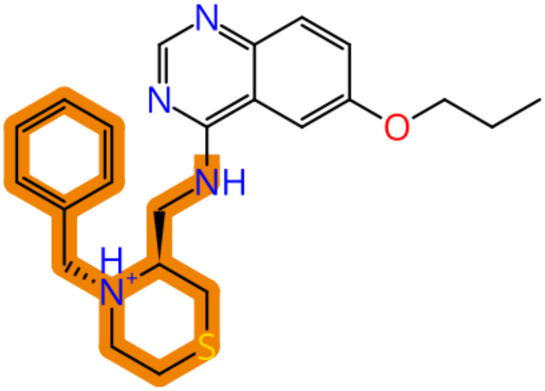
5i	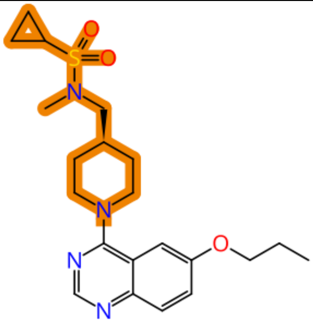	5j	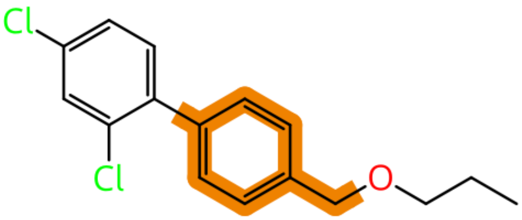
5k	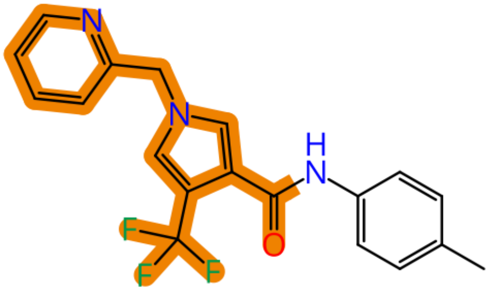	5l	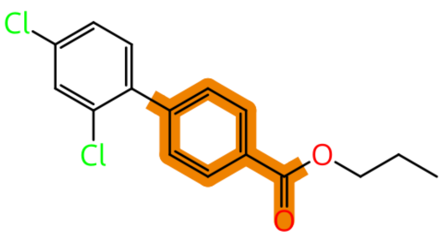
5m	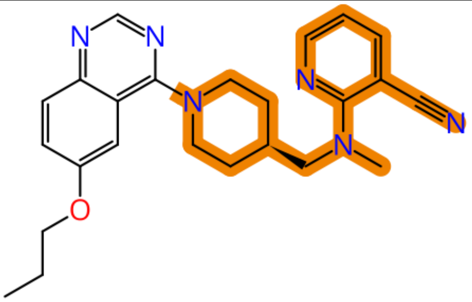	5n	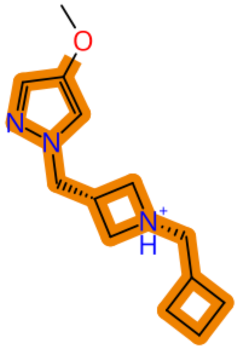

5o	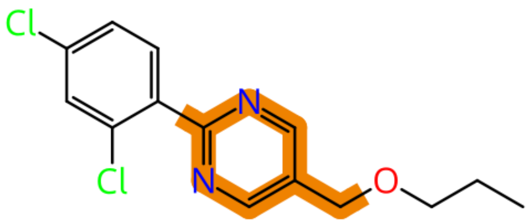	5p	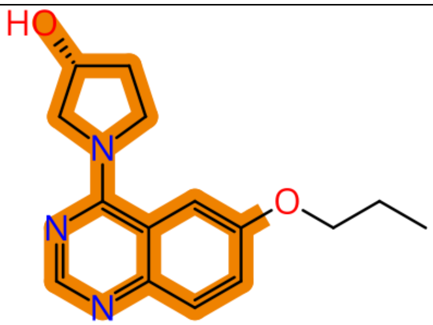
5q	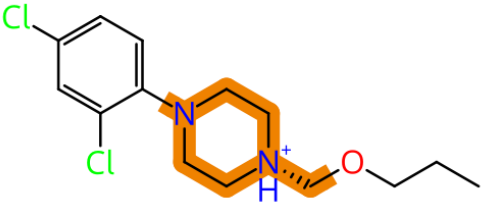	5r	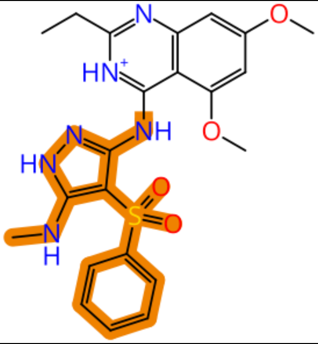
5s	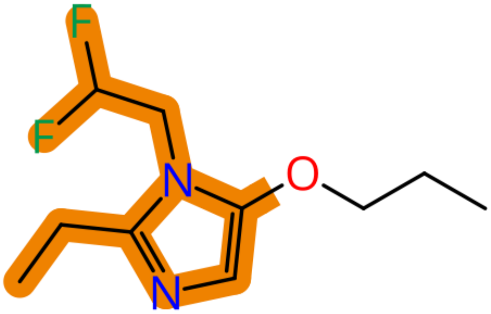	5t	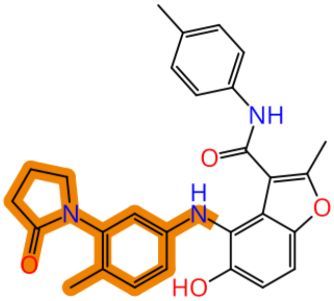
5u	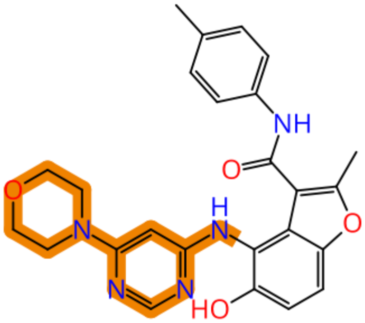	5v	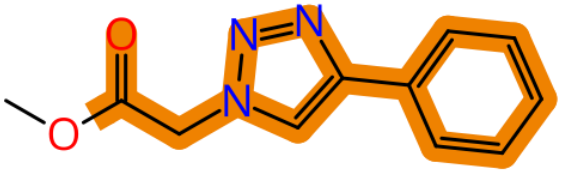
5w	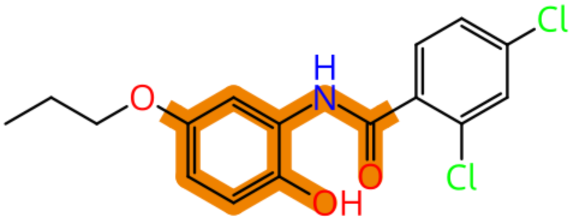	5x	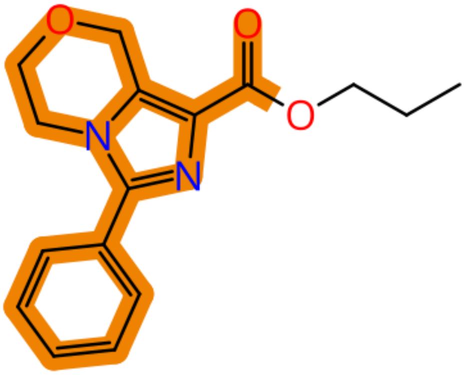

5y	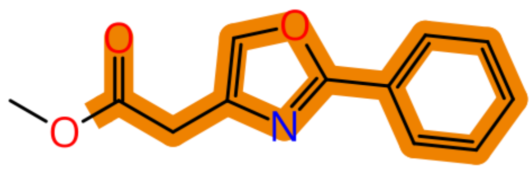	5z	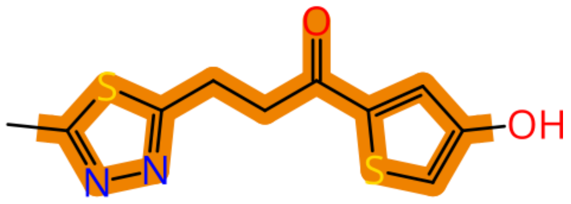
6a	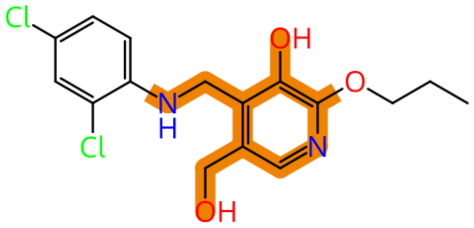	6b	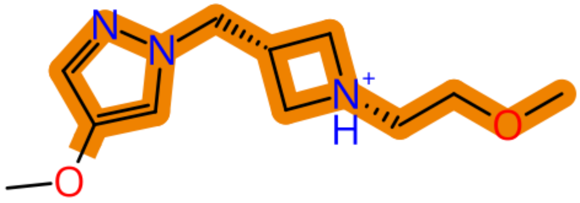
6c	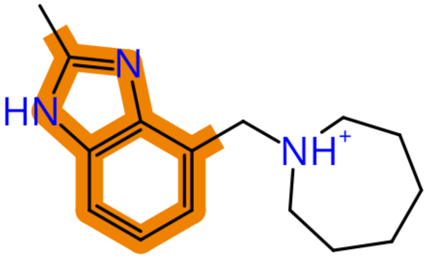	6d	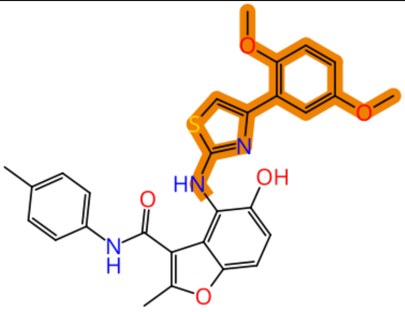
6e	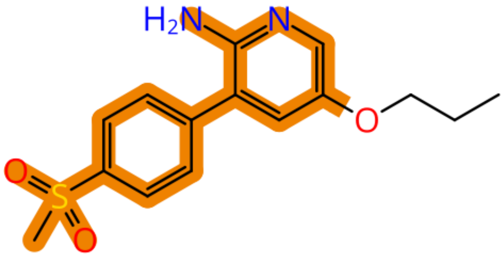	6f	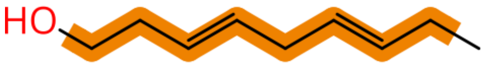
6g	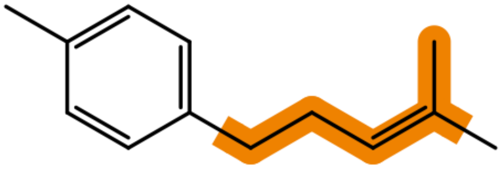	6h	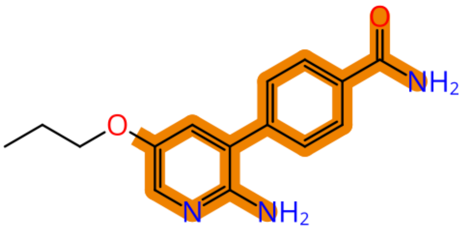

Table S3. HER3 reported inhibitor analogs.

No	Analog Structures	No	Analog Structures
1	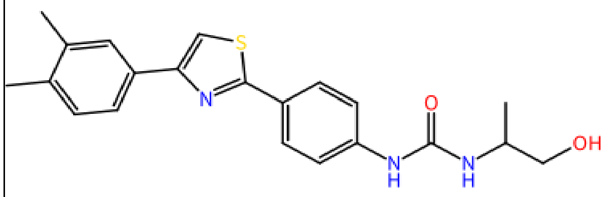	2	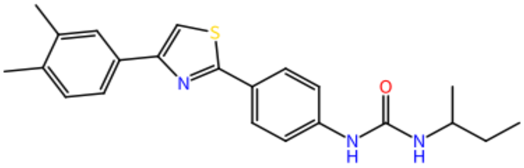
3	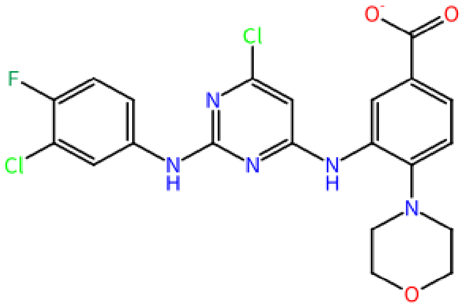	4	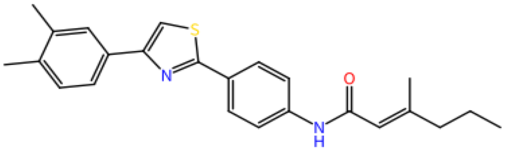
5	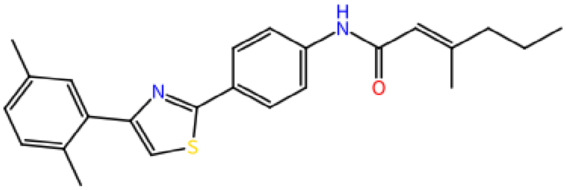	6	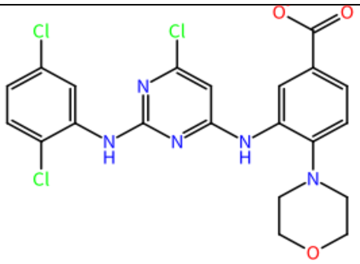
7	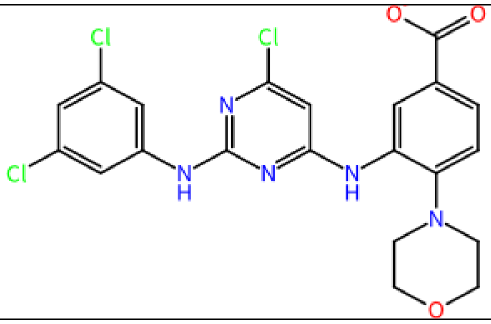	8	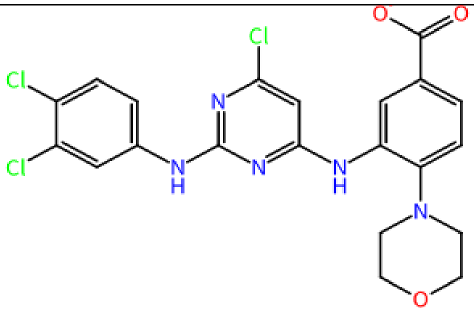
9	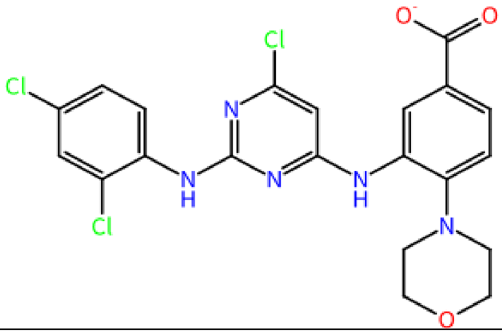	10	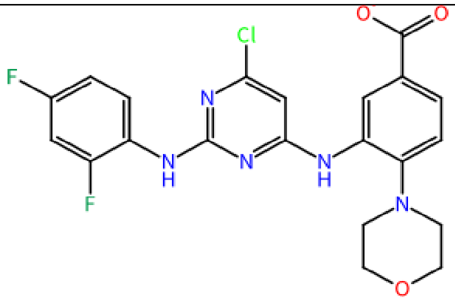

11	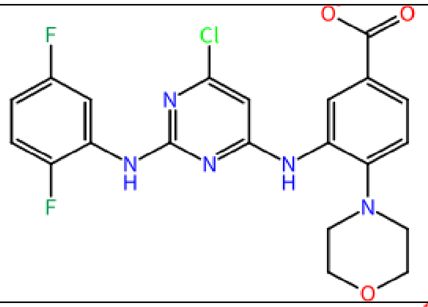	12	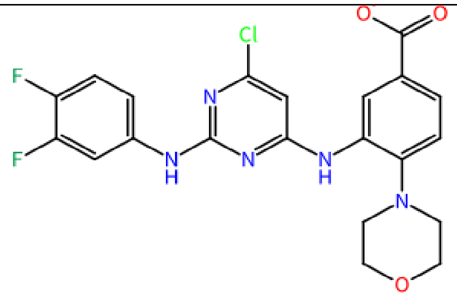
13	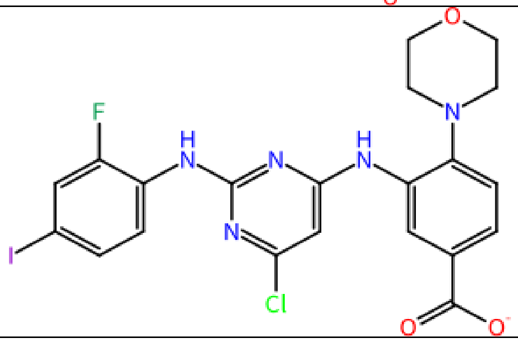	14	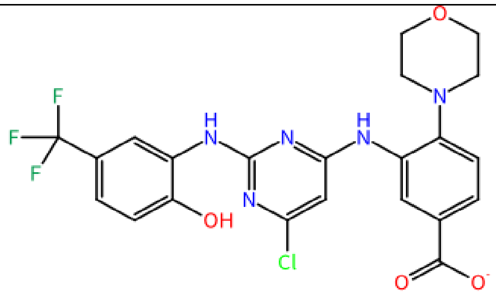
15	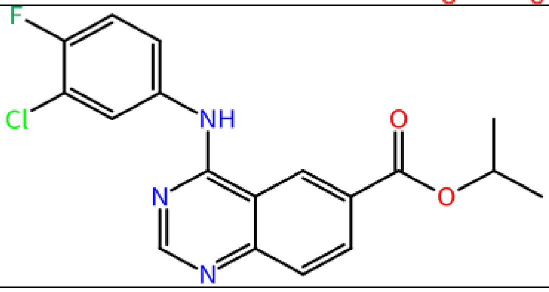	16	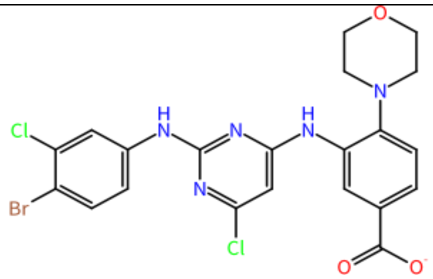
17	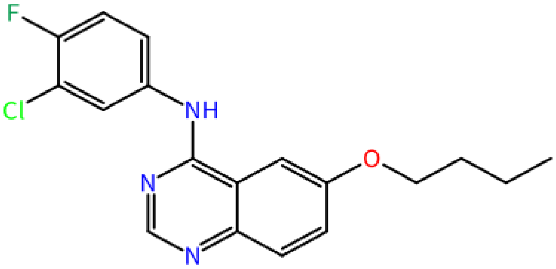	18	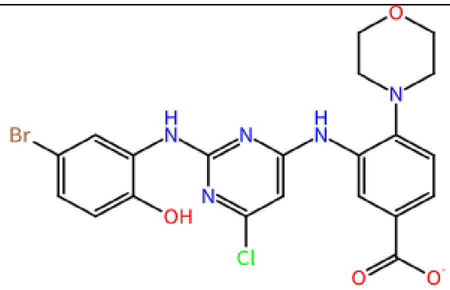
19	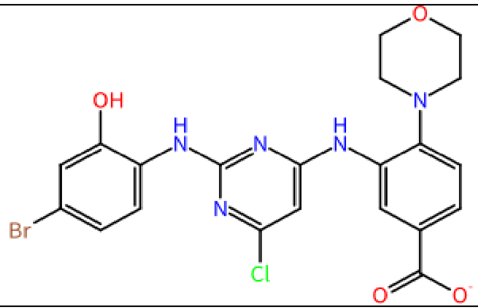	20	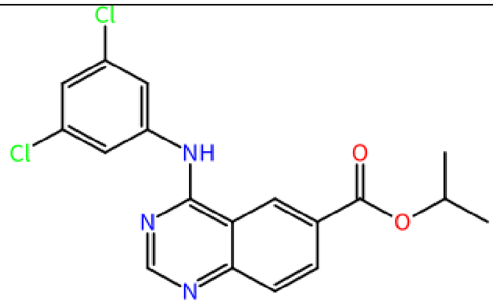
21	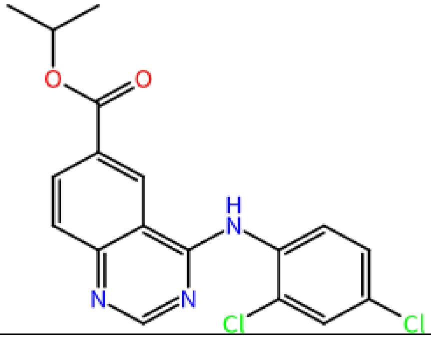	22	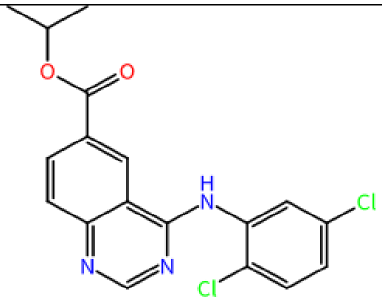

23	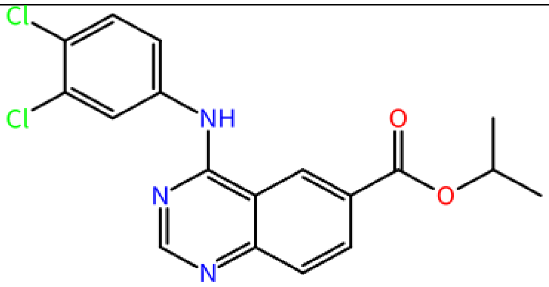	24	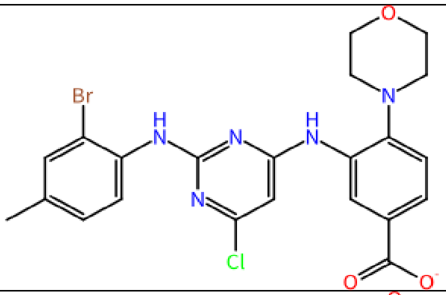
25	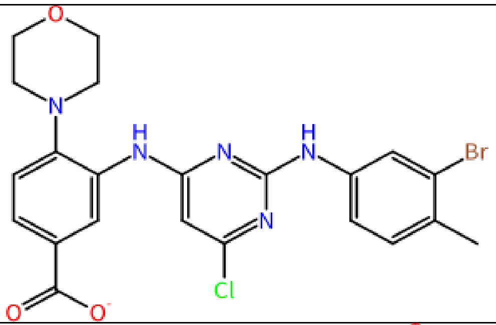	26	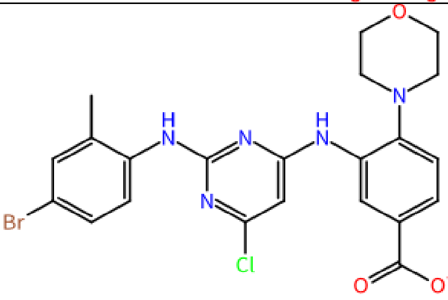
27	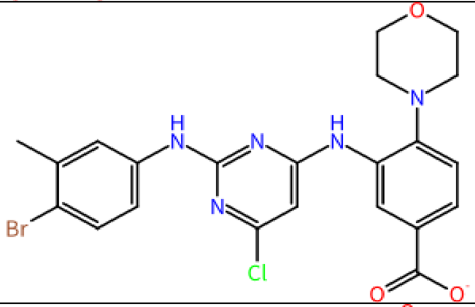	28	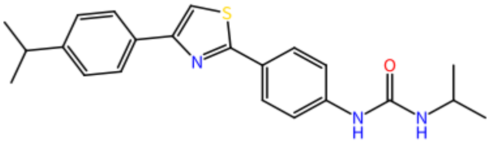
29	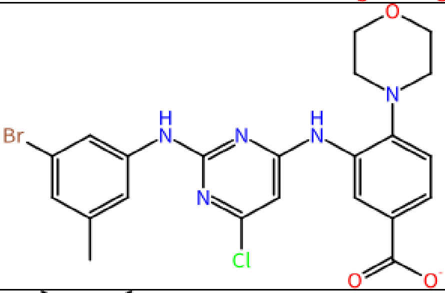	30	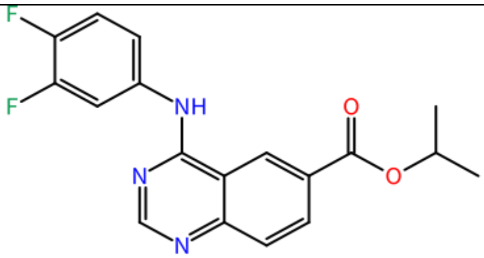
31	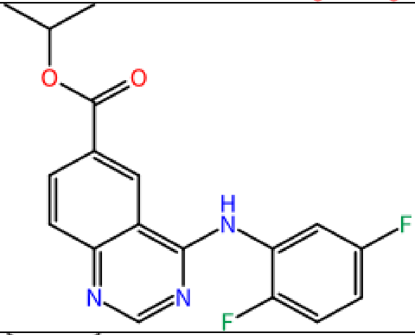	32	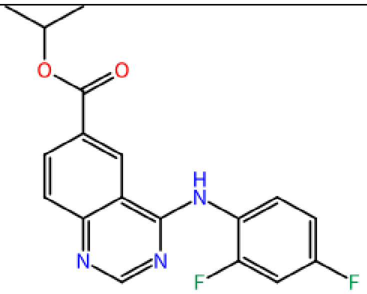
33	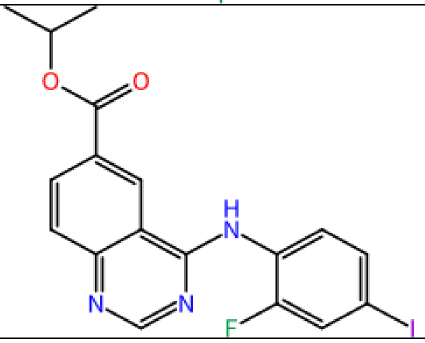	34	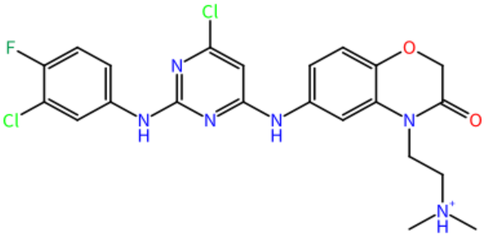

35	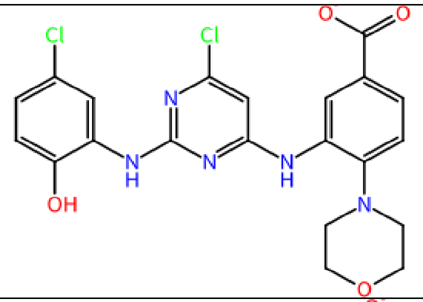	36	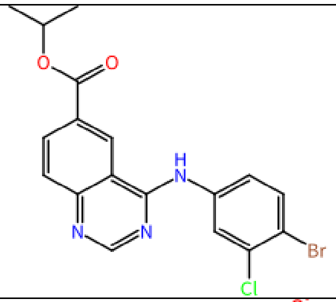
37	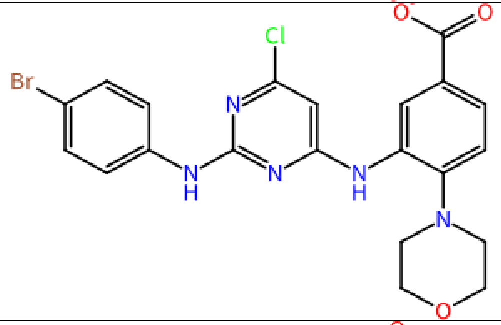	38	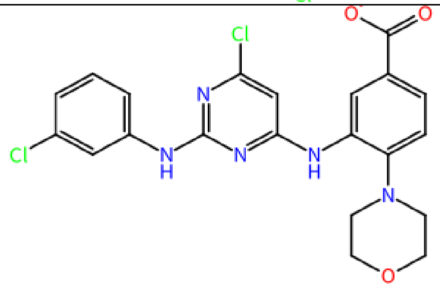
39	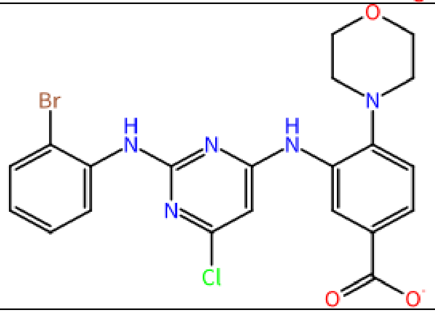	40	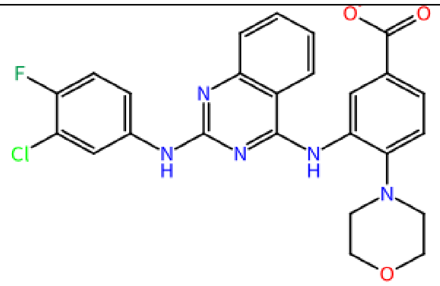
41	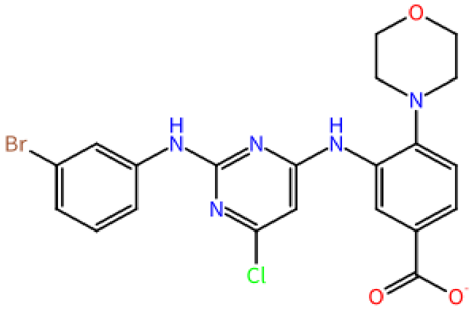	42	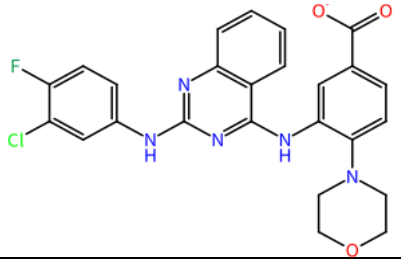
43	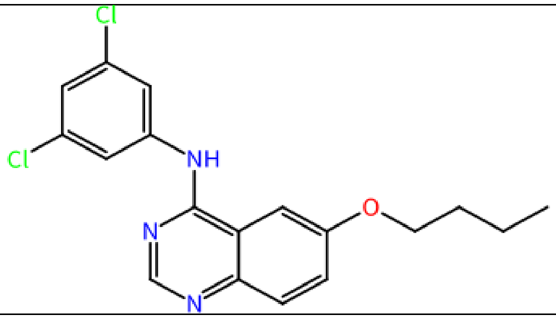	44	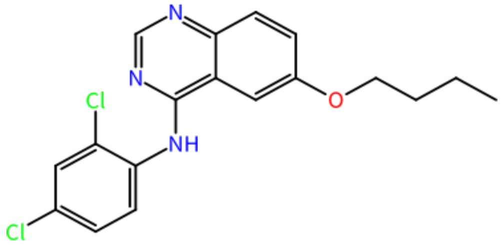

45	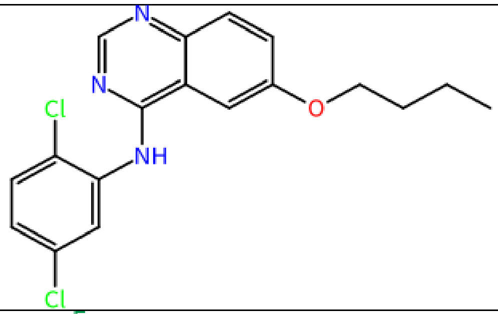	46	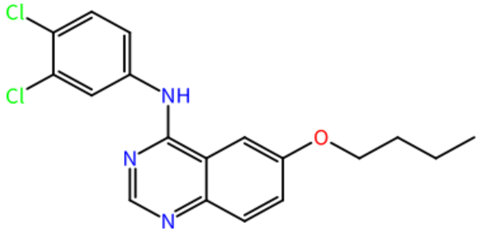
47	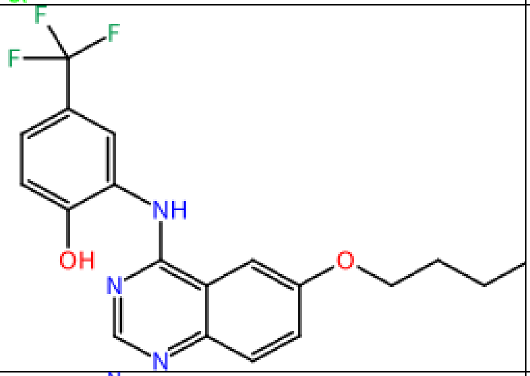	48	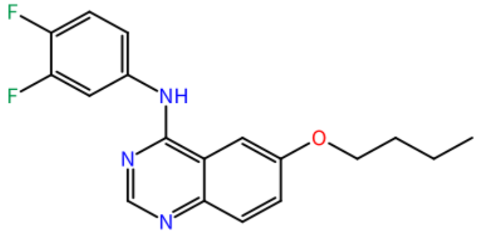
49	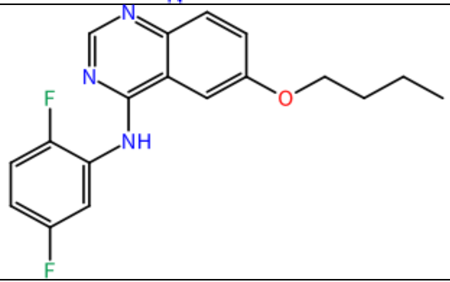	50	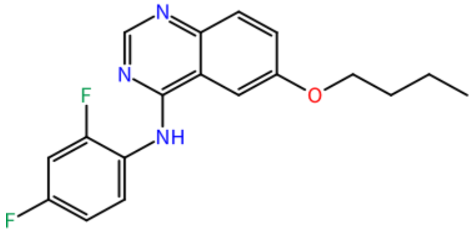
51	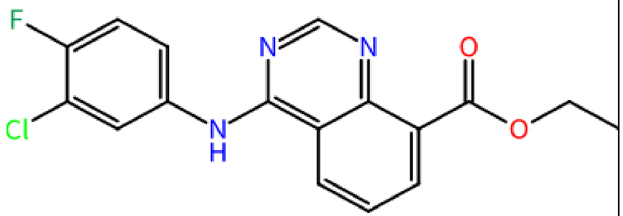	52	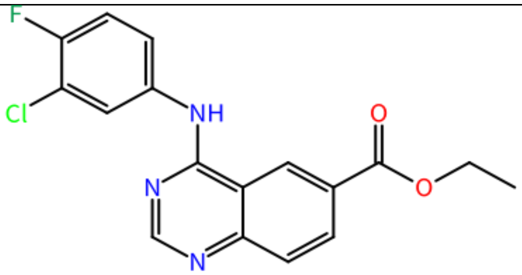
53	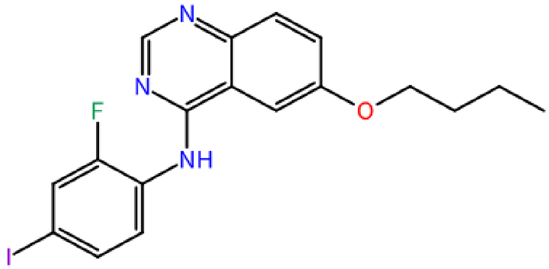	54	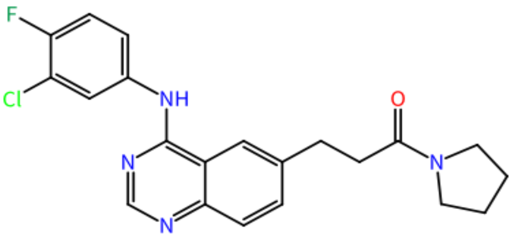
55	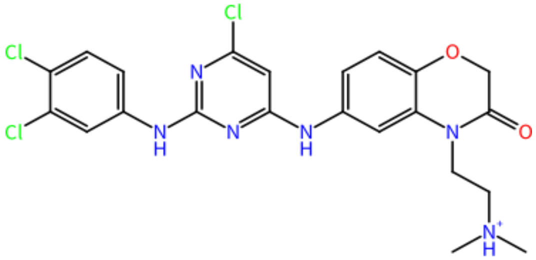	56	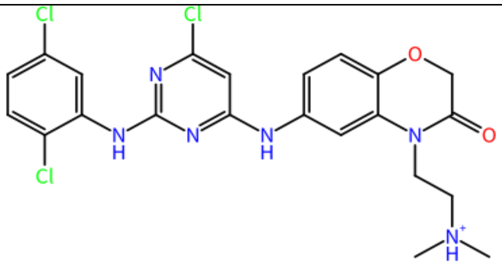

57	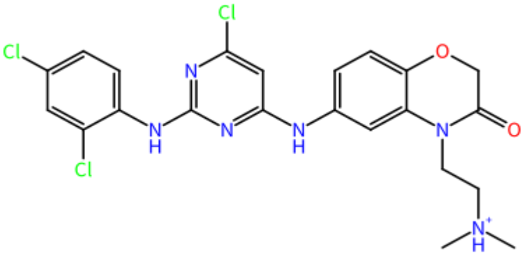	58	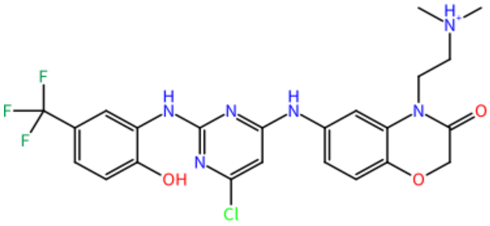
59	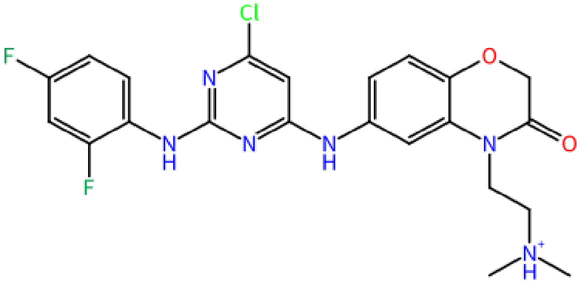	60	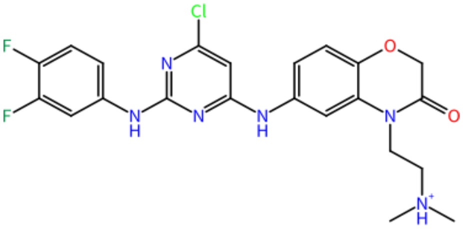
61	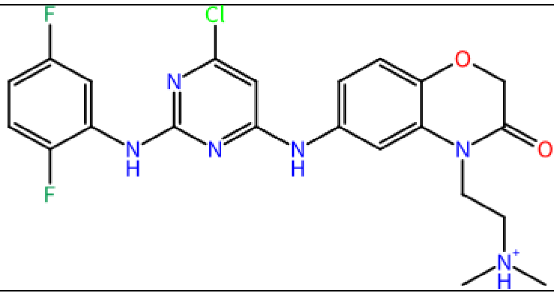	62	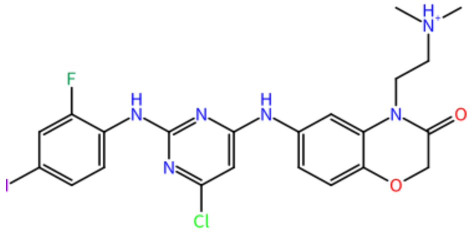
63	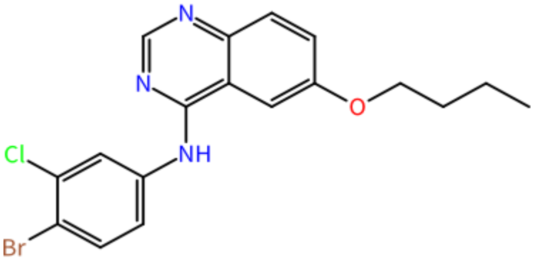	64	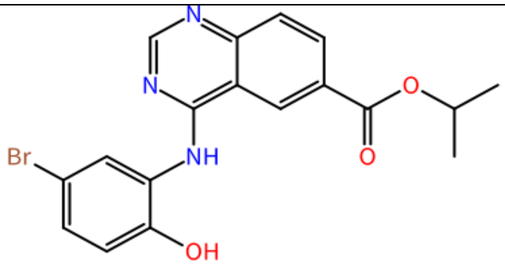
65	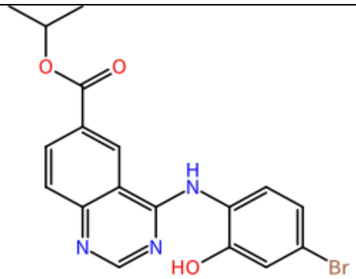	66	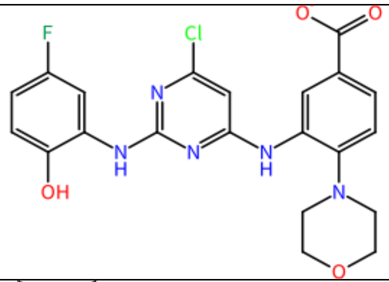
67	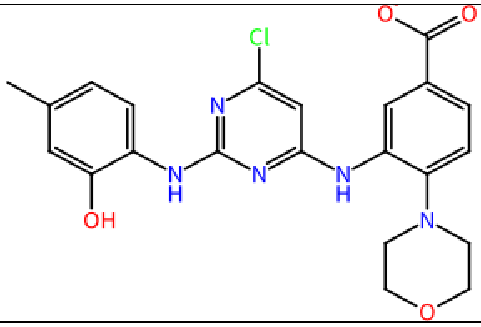	68	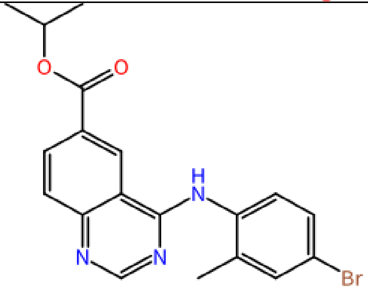

69	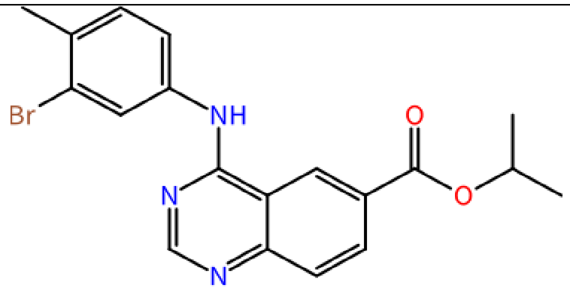	70	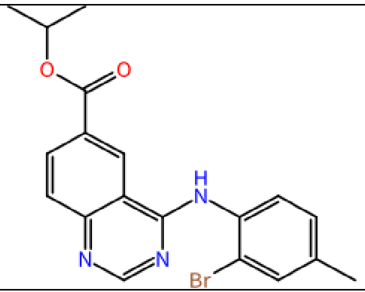
71	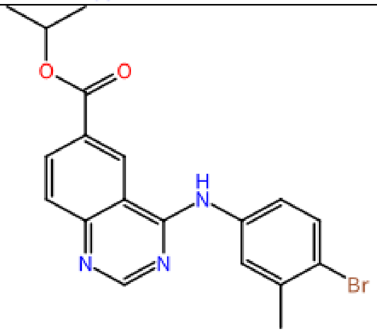	72	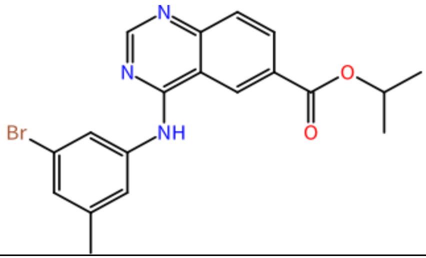
73	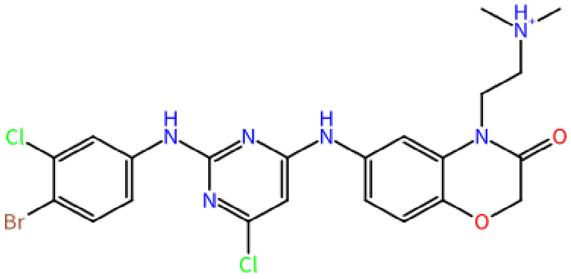	74	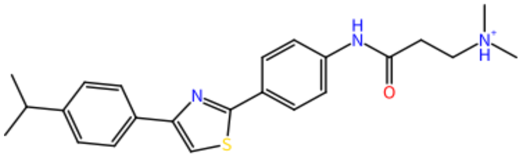
75	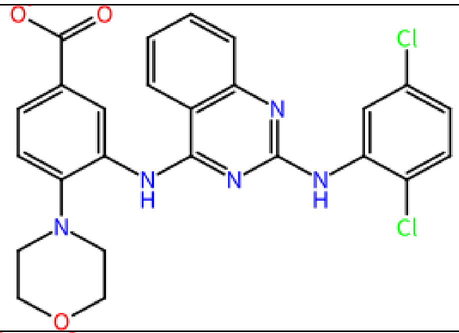	76	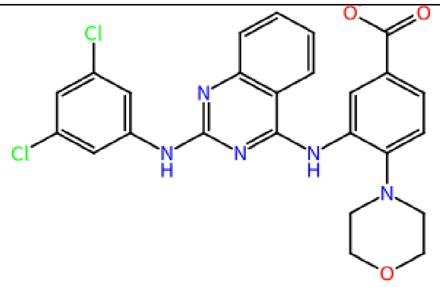
77	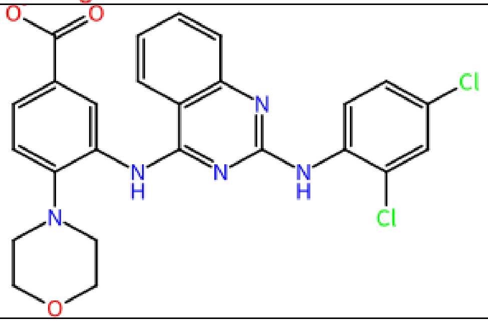	78	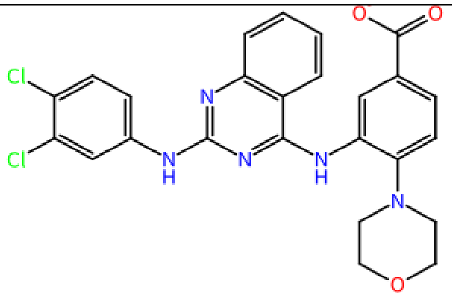
79	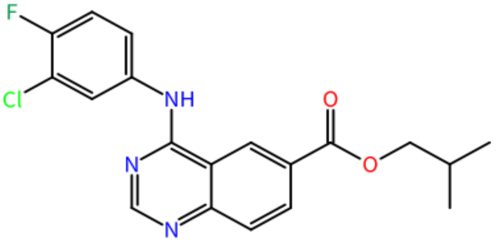	80	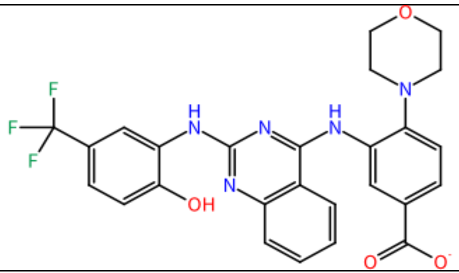

81	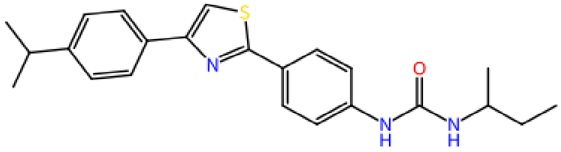	82	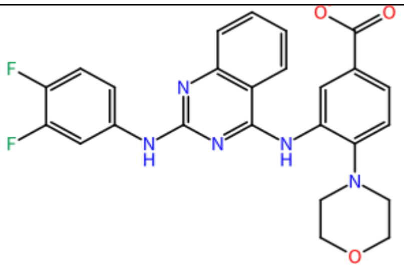
83	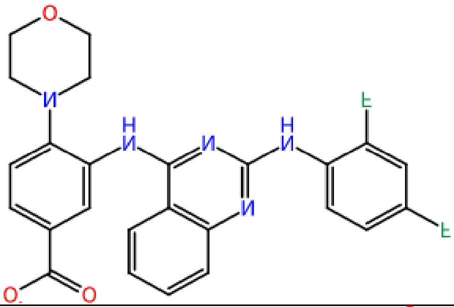	84	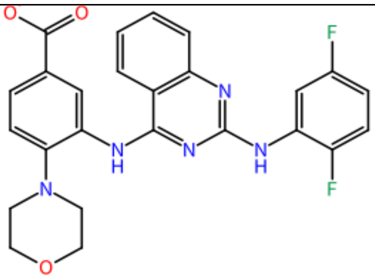
85	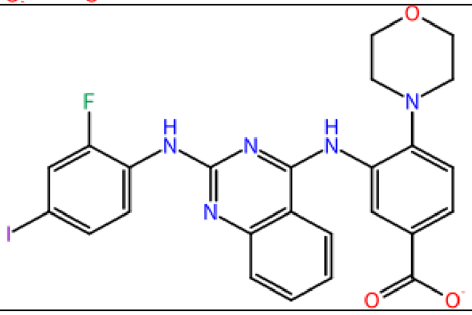	86	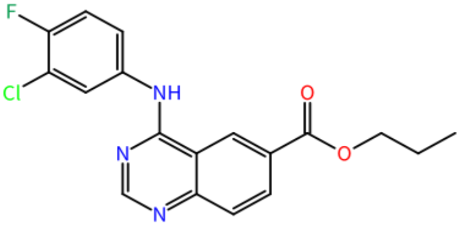
87	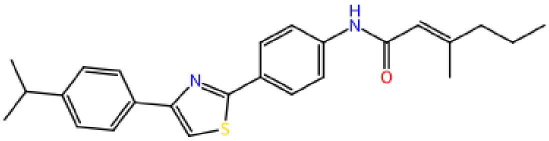	88	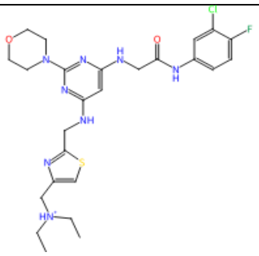
89	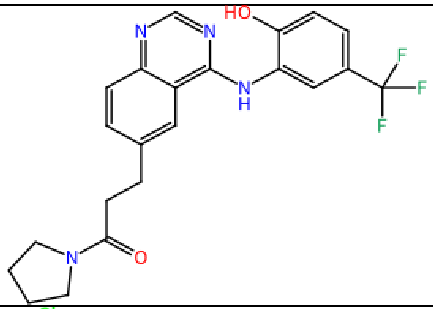	90	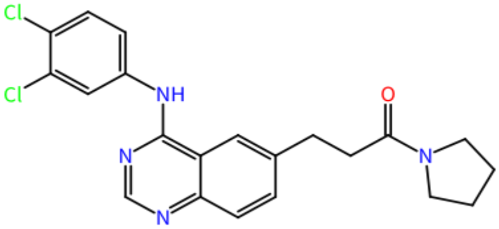
91	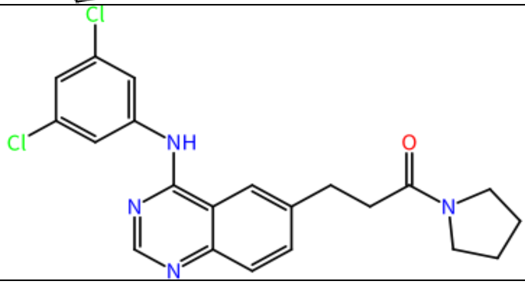	92	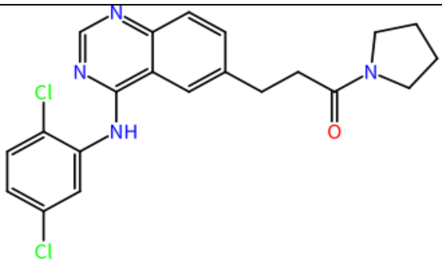

93	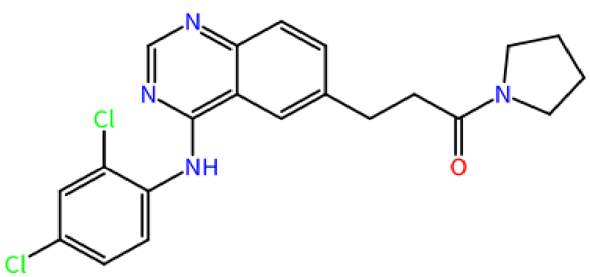	94	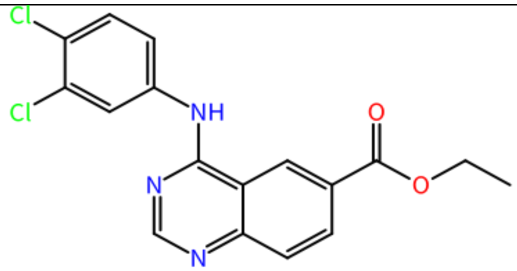
95	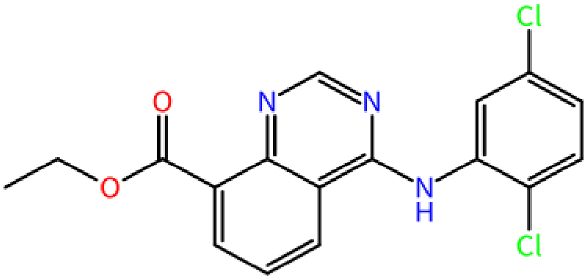	96	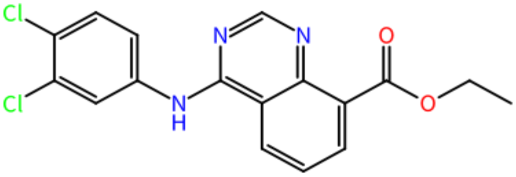
97	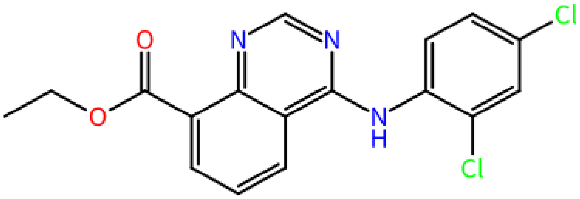	98	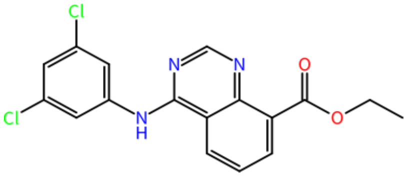
99	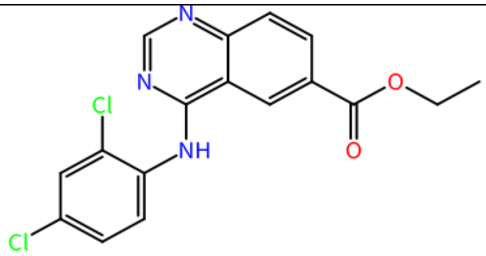	100	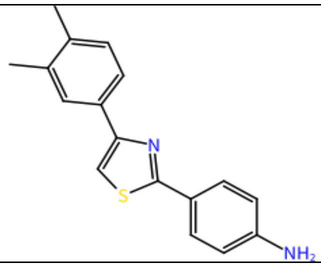
101	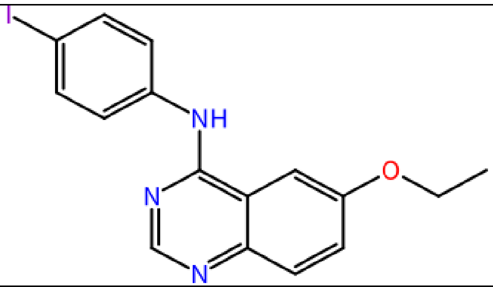	102	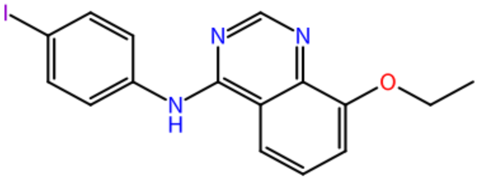
103	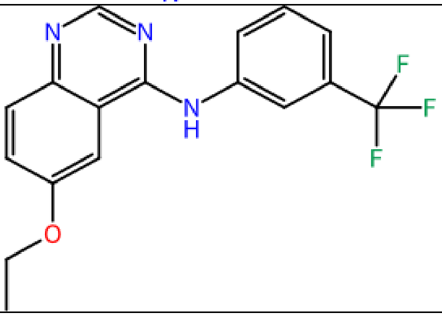	104	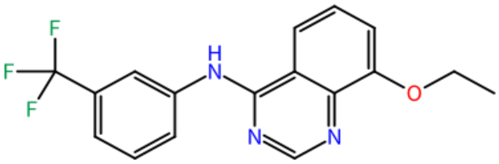

105	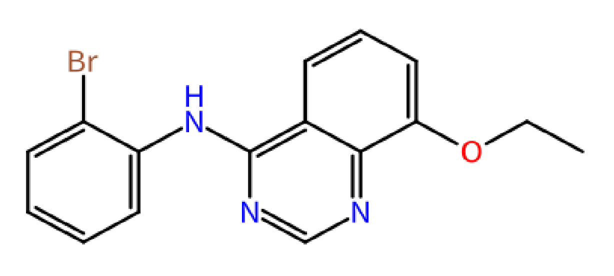	106	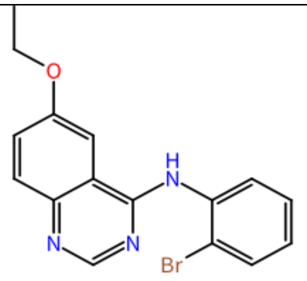
107	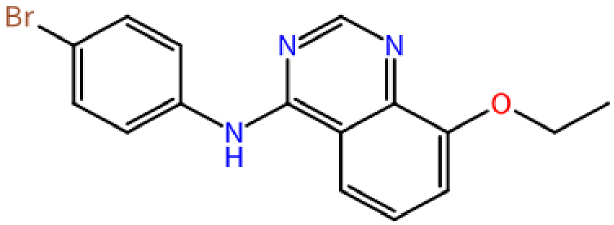	108	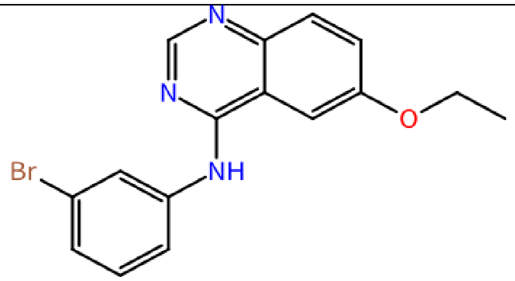
109	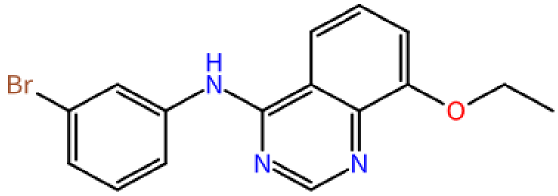	110	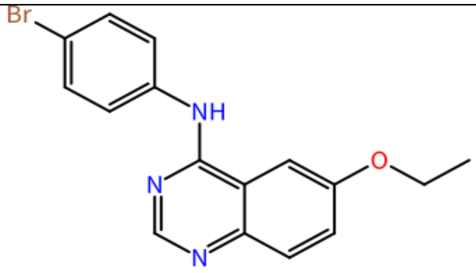
111	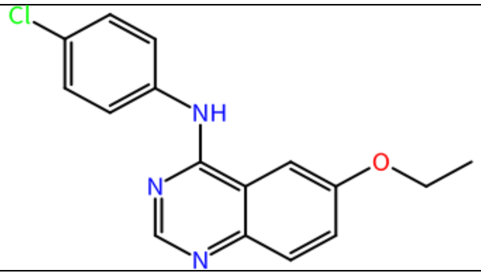	112	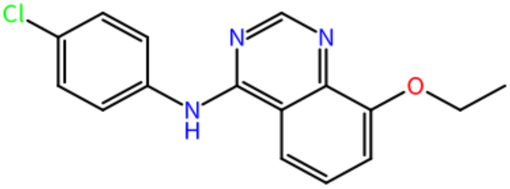
113	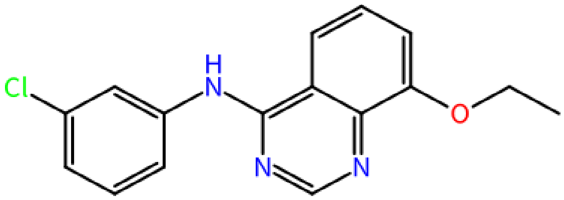	114	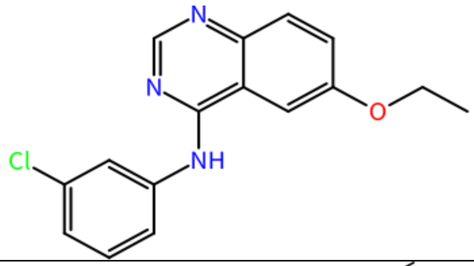
115	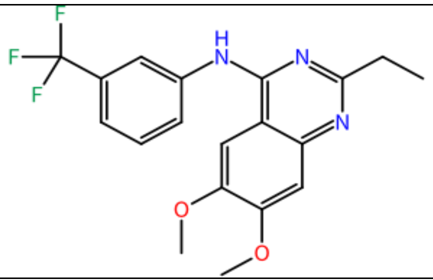	116	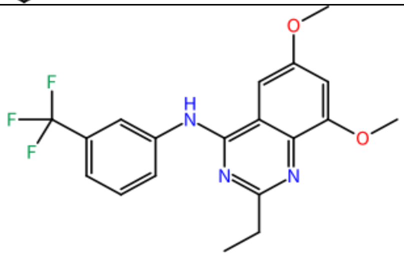

117	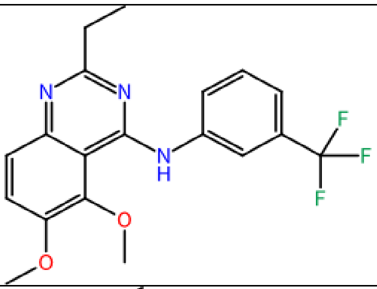	118	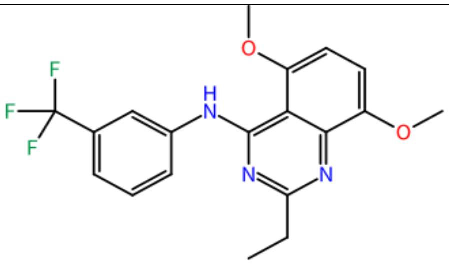
119	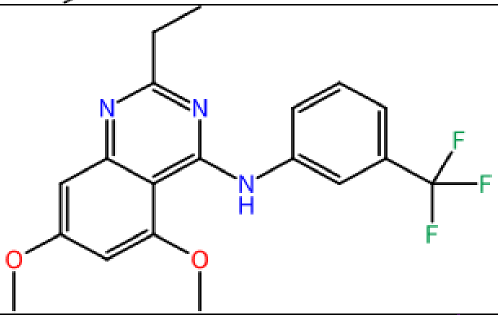	120	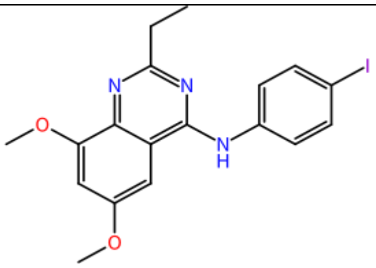
121	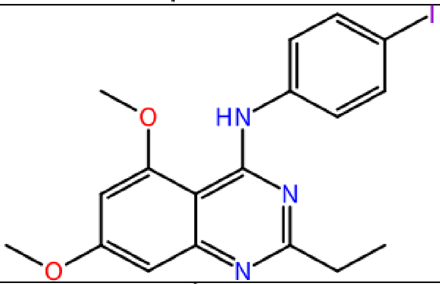	122	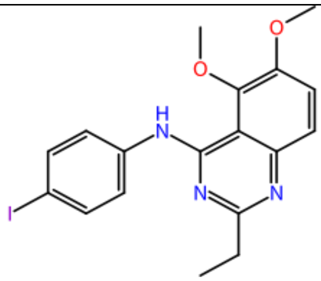
123	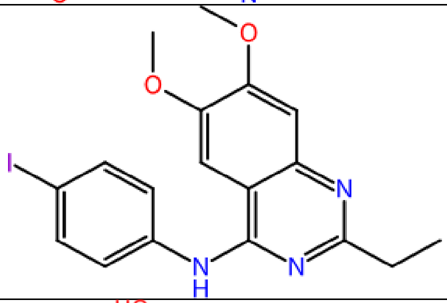	124	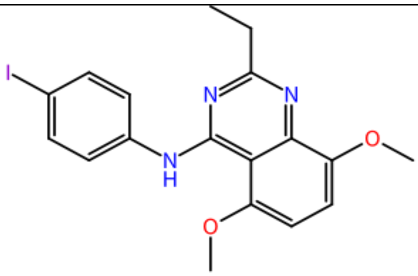
125	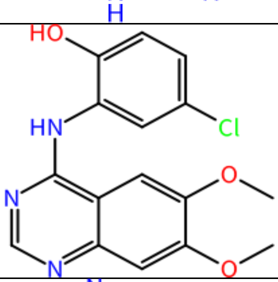	126	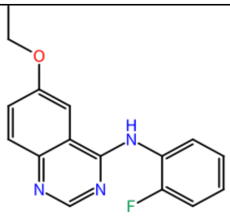
127	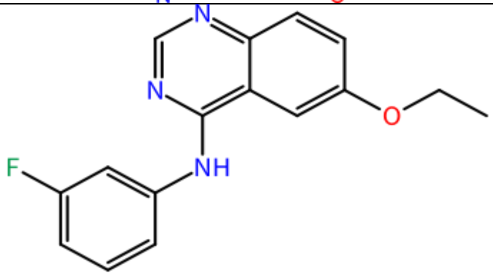	128	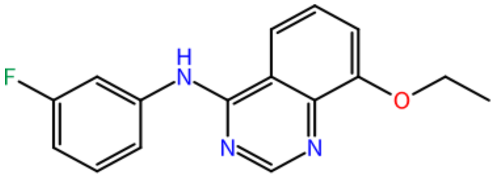

129	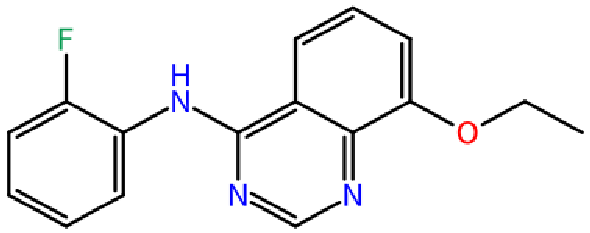	130	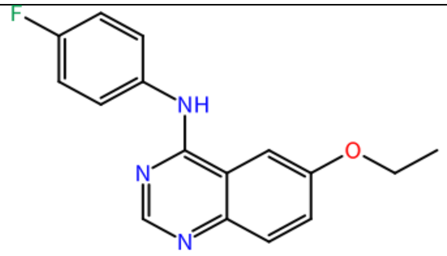
131	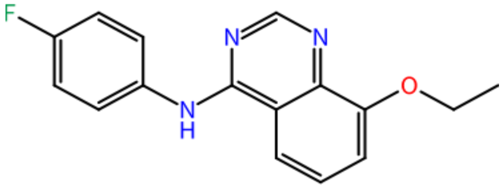	132	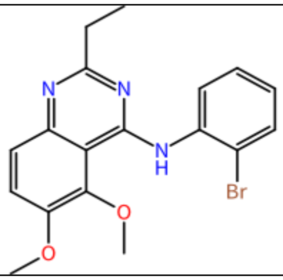
133	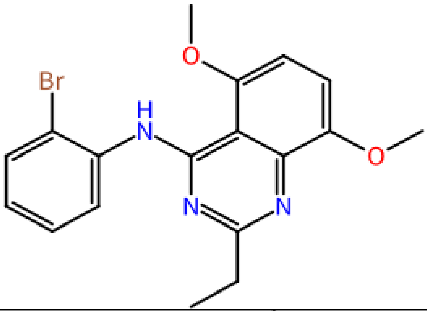	134	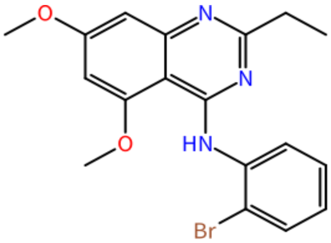
135	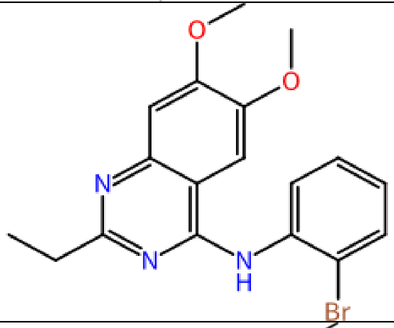	136	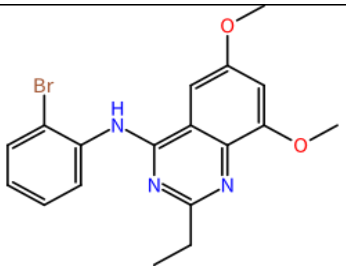
137	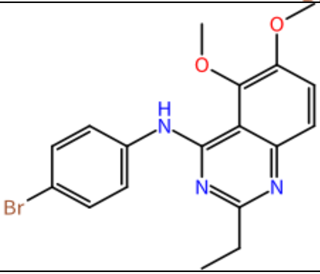	138	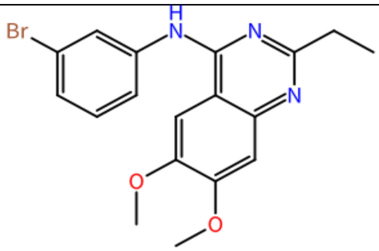
139	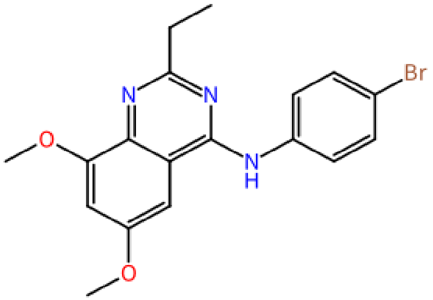	140	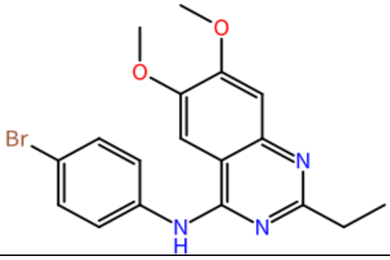

141	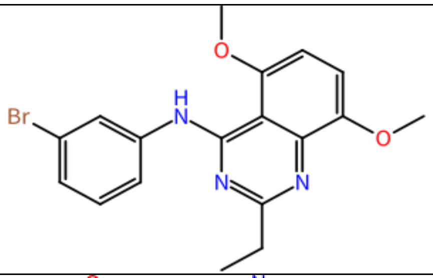	142	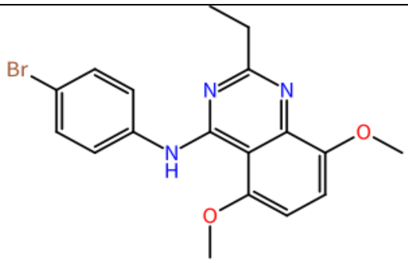
143	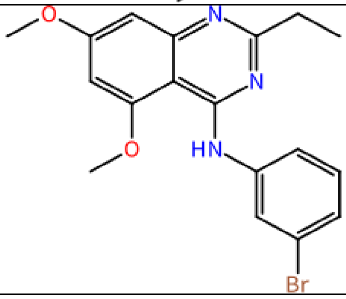	144	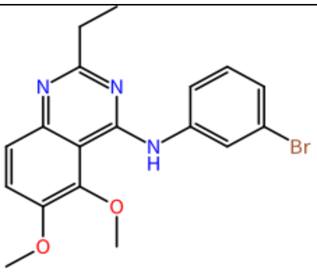
145	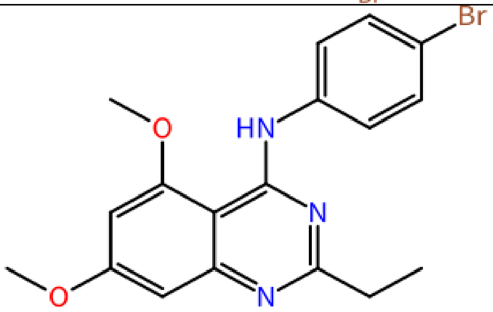	146	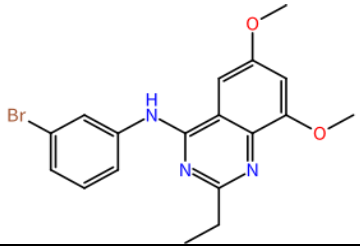
147	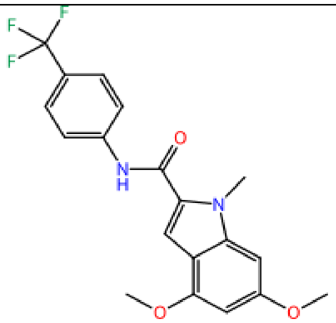	148	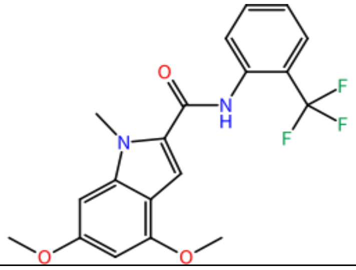
149	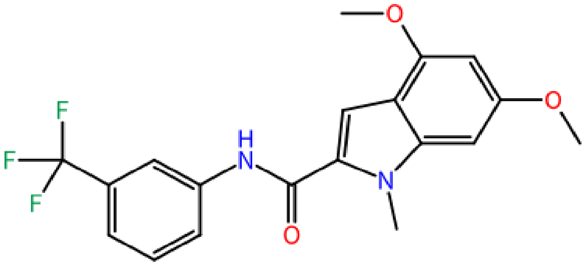	150	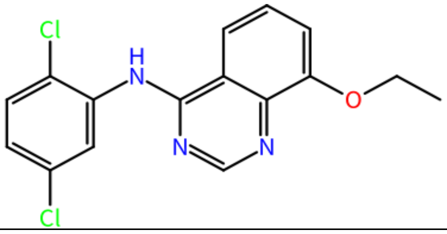
151	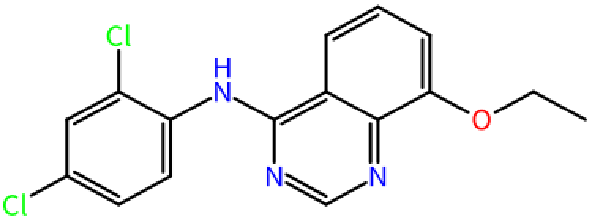	152	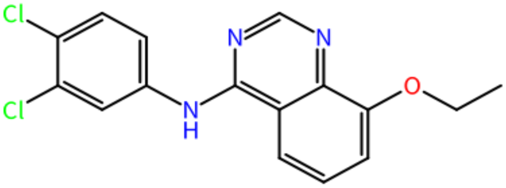

153	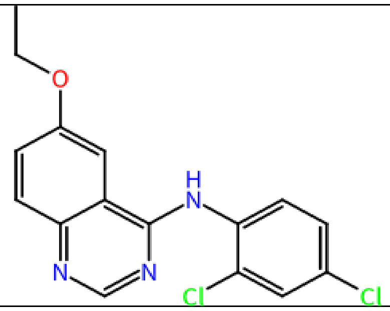	154	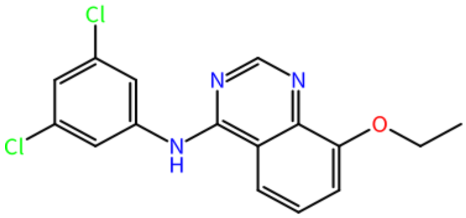
155	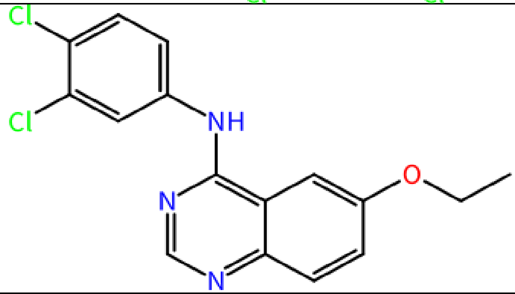	156	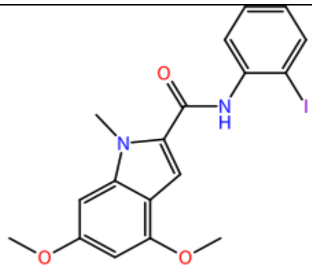
157	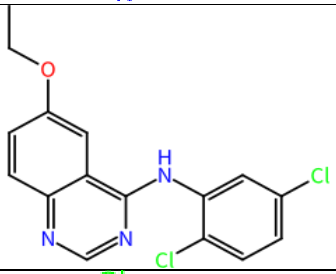	158	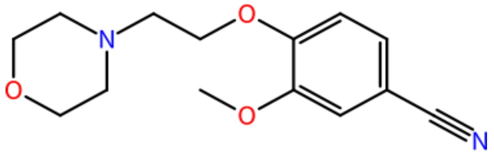
159	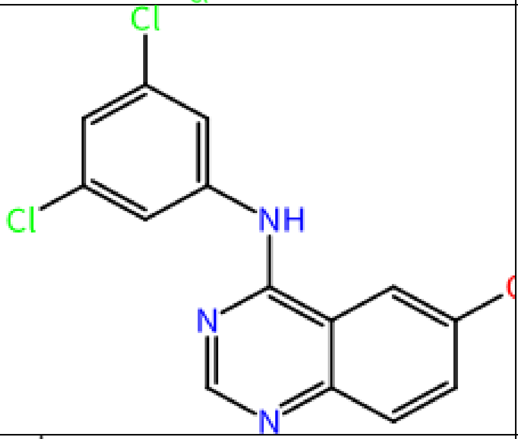	160	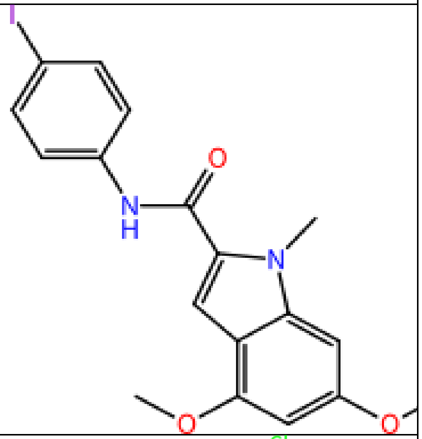
161	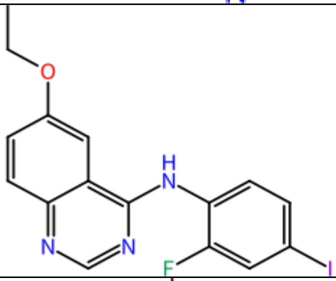	162	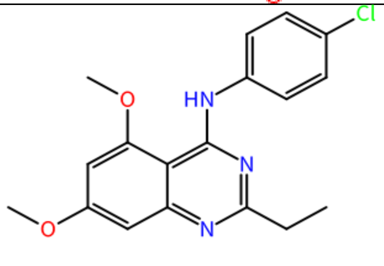
163	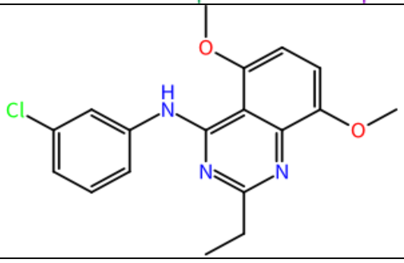	164	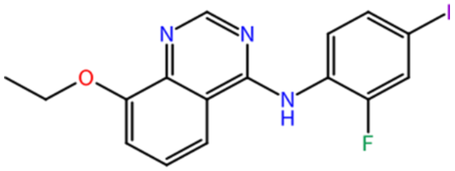

165	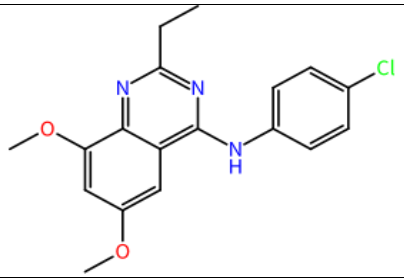	166	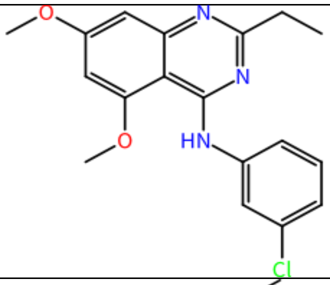
167	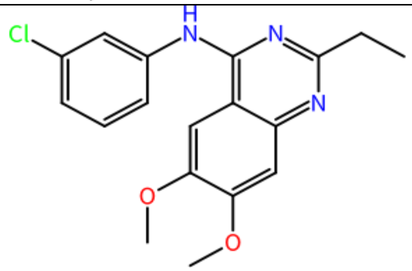	168	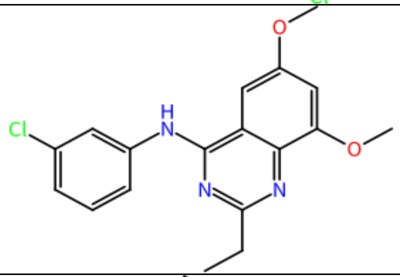
169	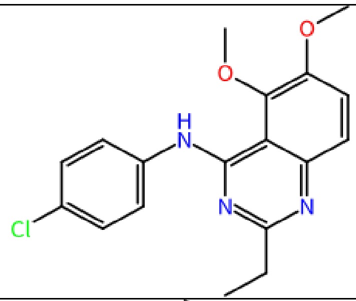	170	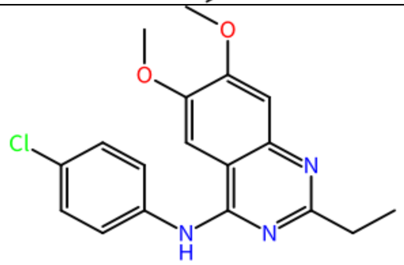
171	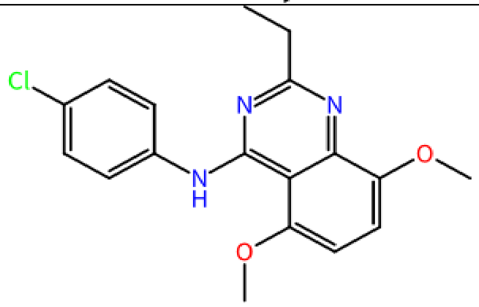	172	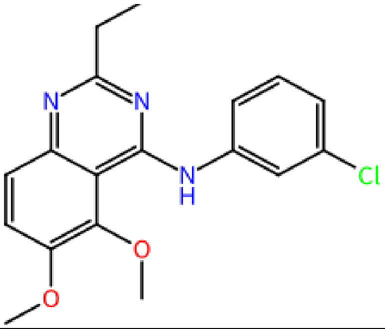
173	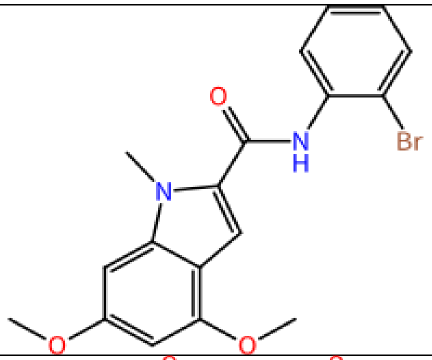	174	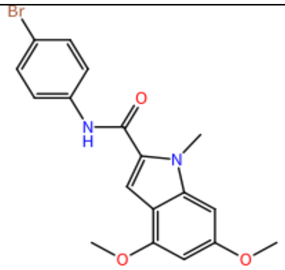
175	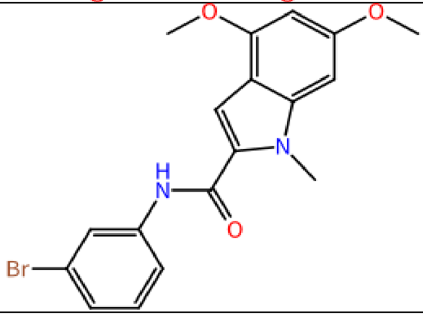	176	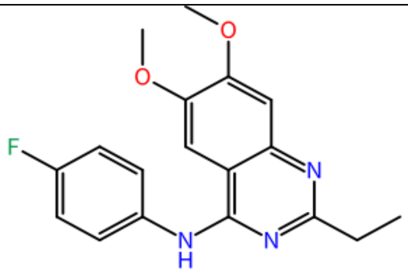

177	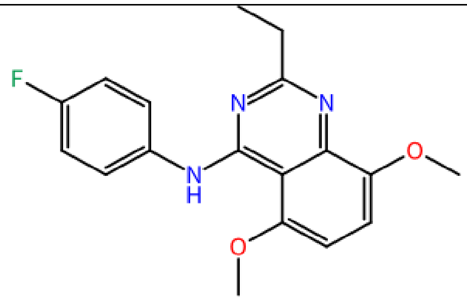	178	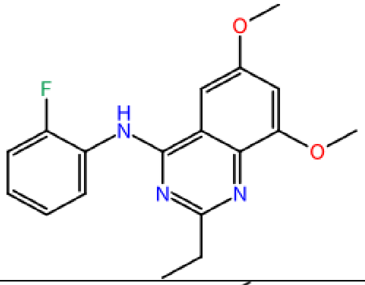
179	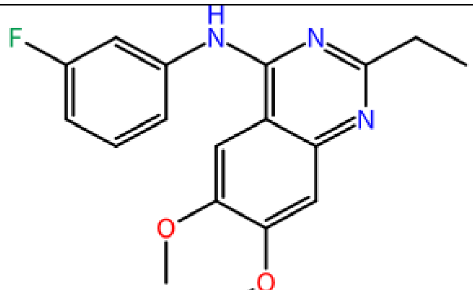	180	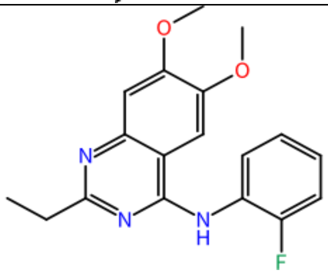
181	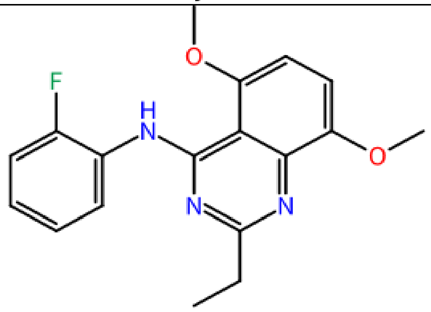	182	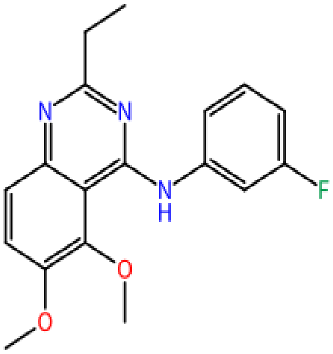
183	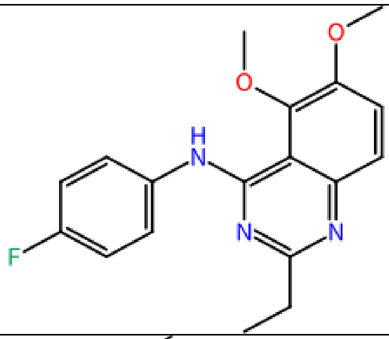	184	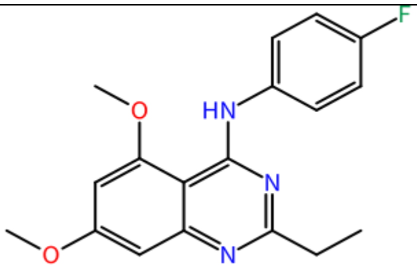
185	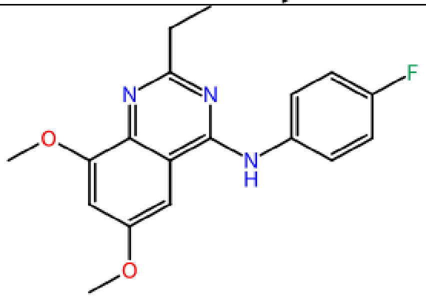	186	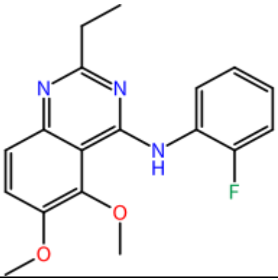

187	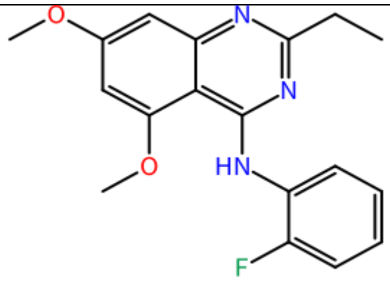	188	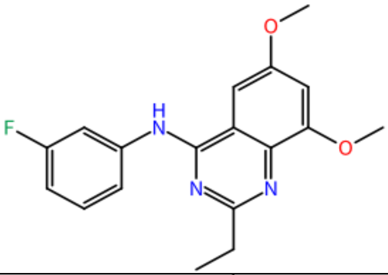
189	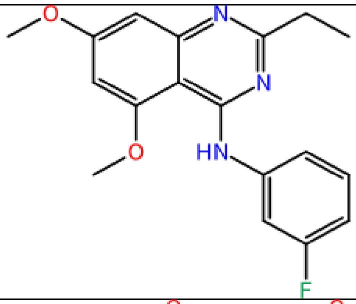	190	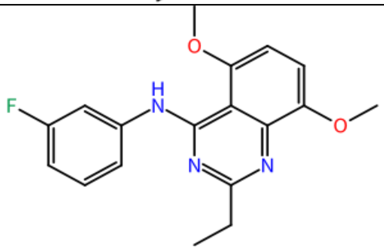
191	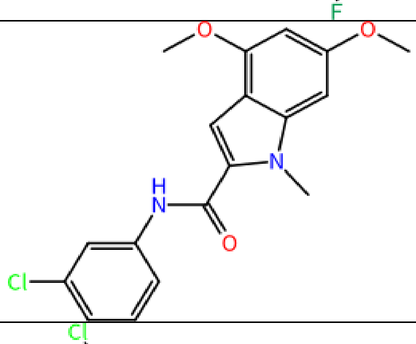	192	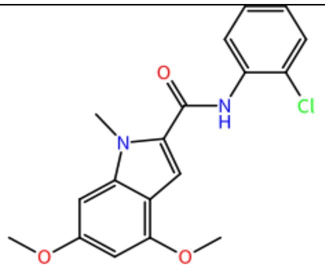
193	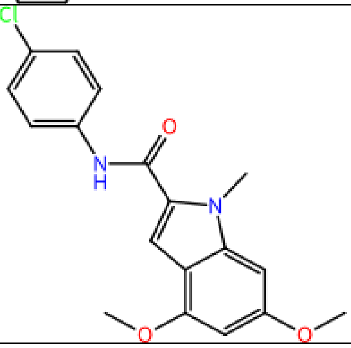	194	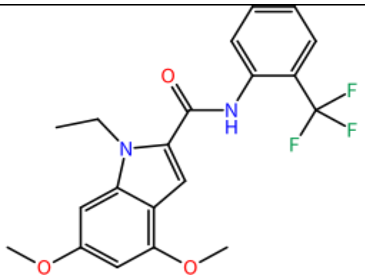
195	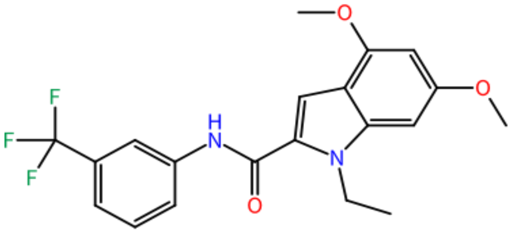	196	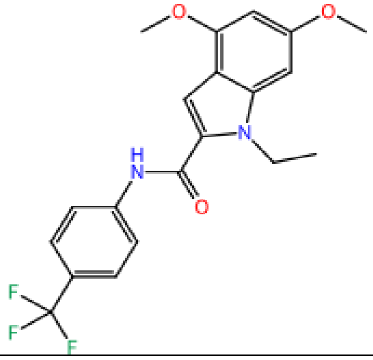

197	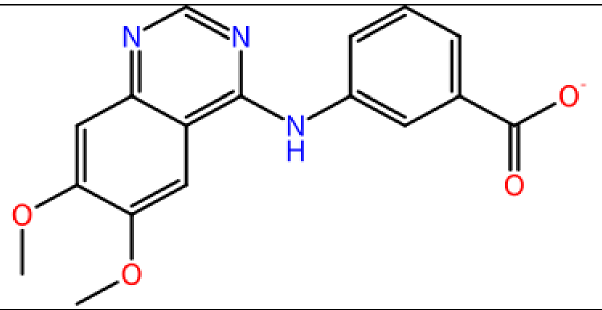	198	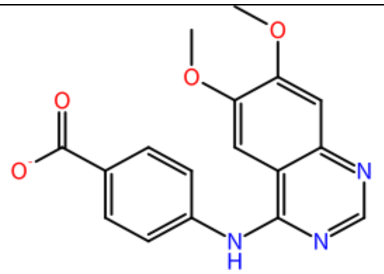
199	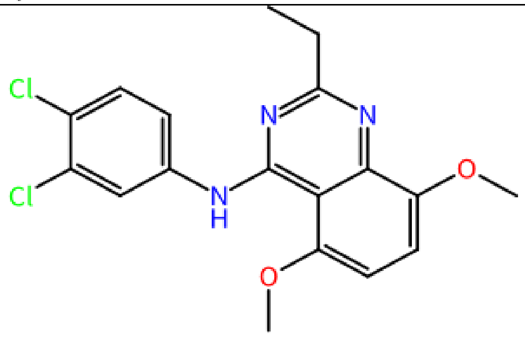	200	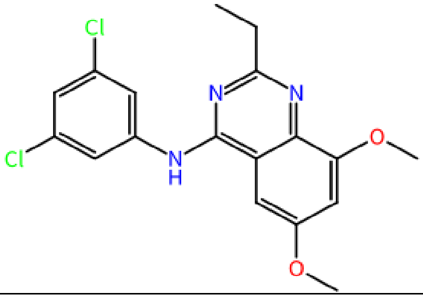
201	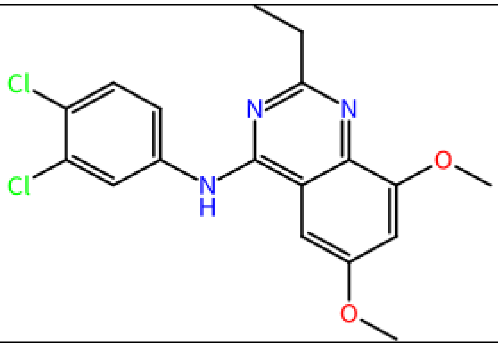	202	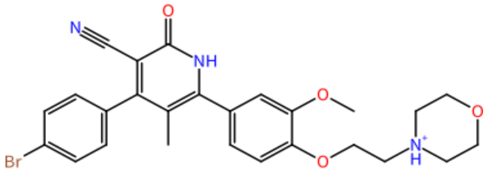
203	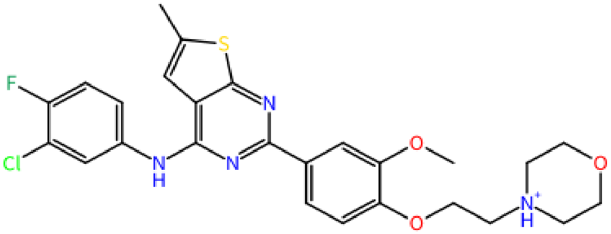	204	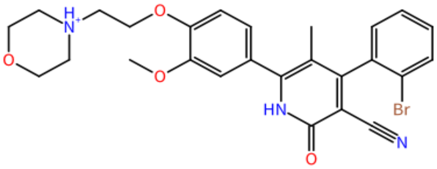
205	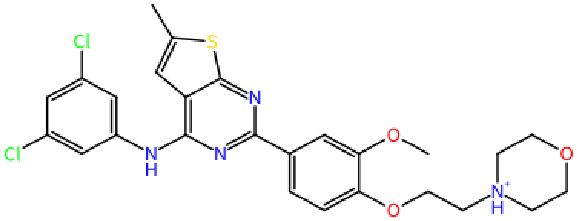	206	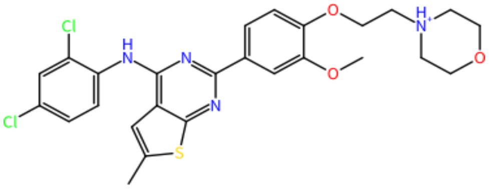
207	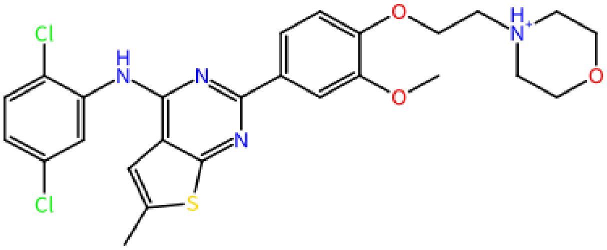	208	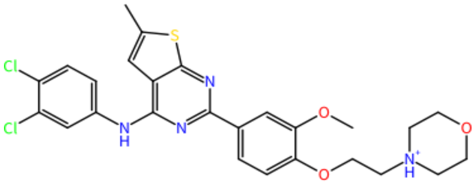

209	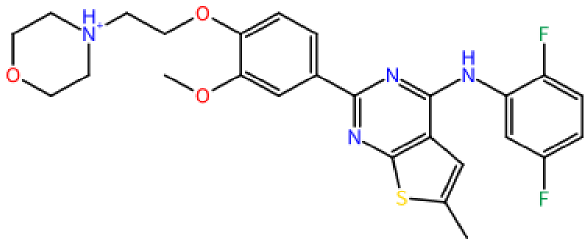	210	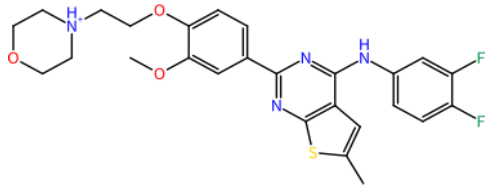
211	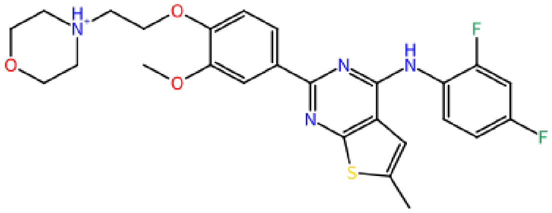	212	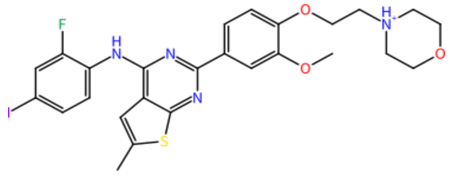
213	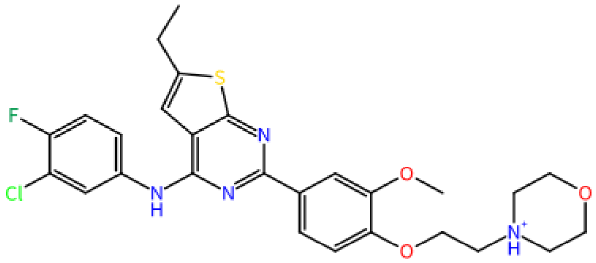	214	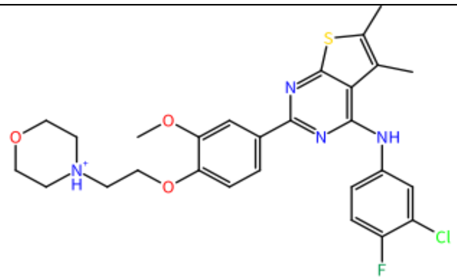
215	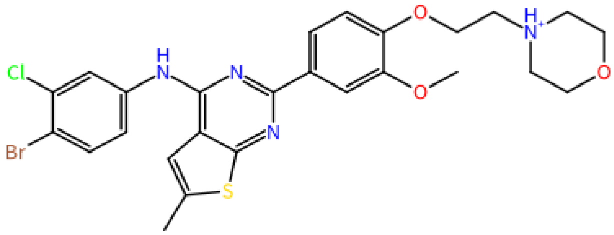	216	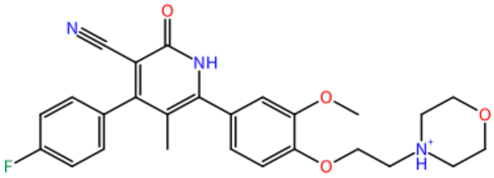
217	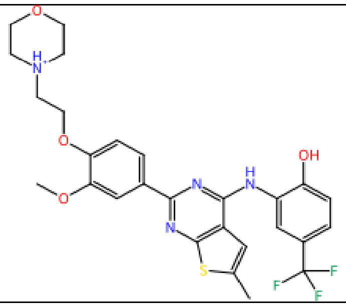	218	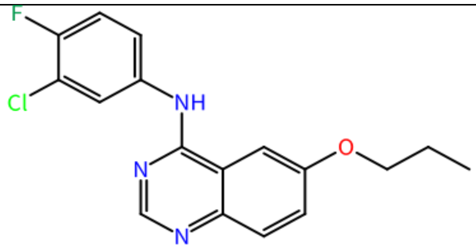

219	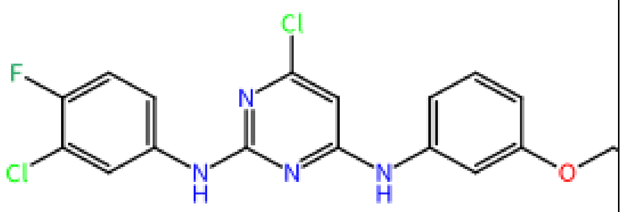	220	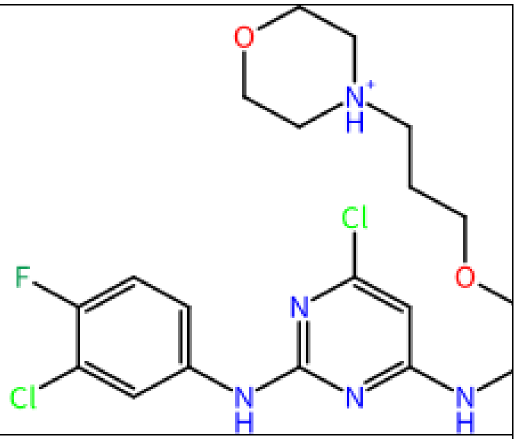
221	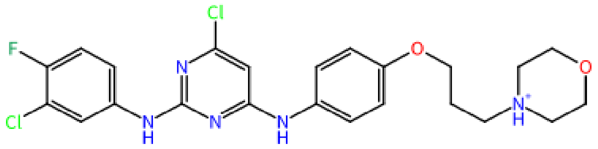	222	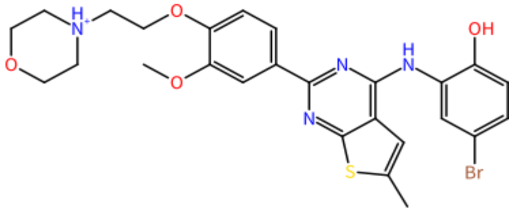
223	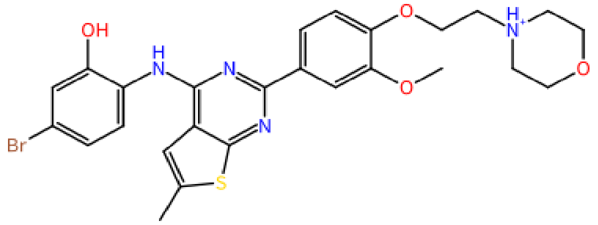	224	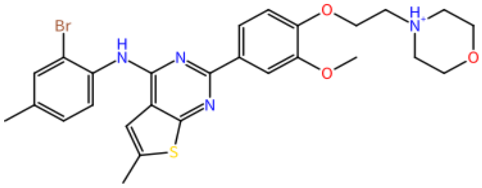
225	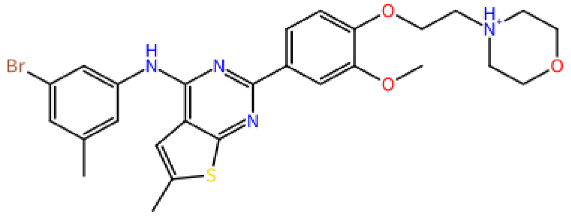	226	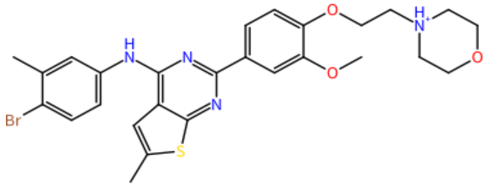
227	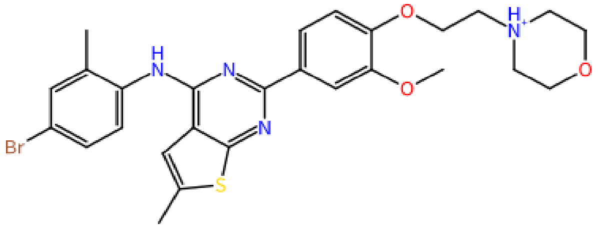	228	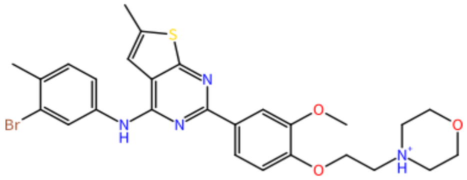
229	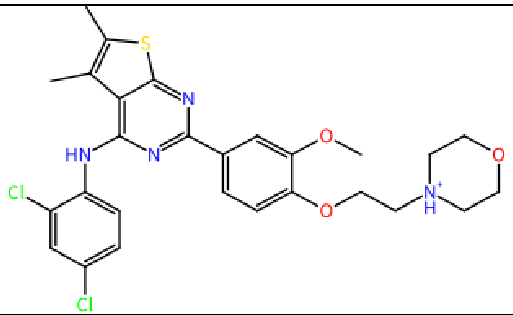	230	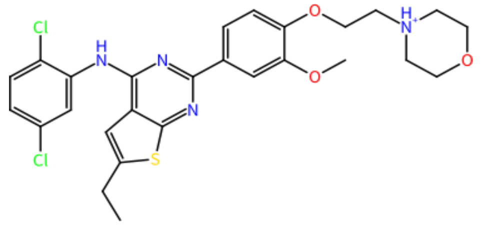

231	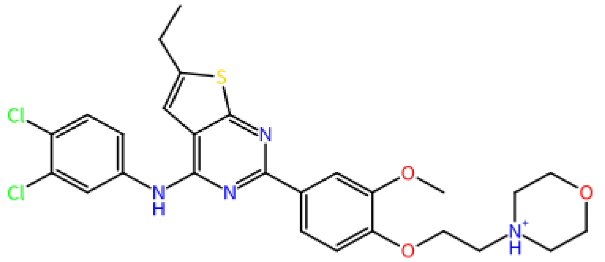	232	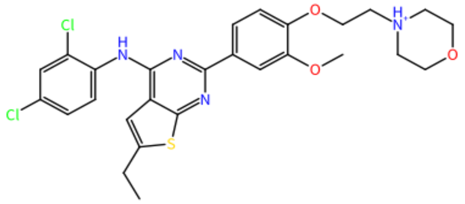
233	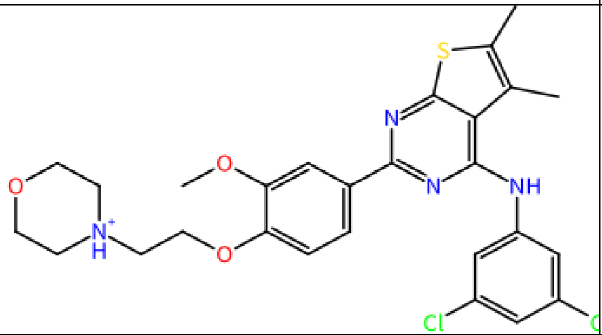	234	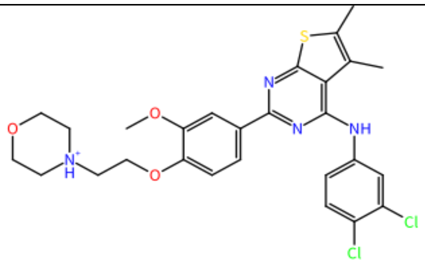
235	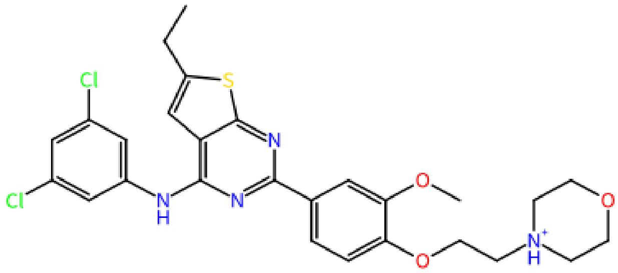	236	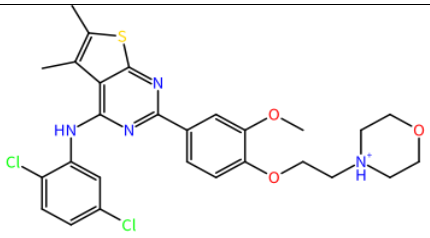
237	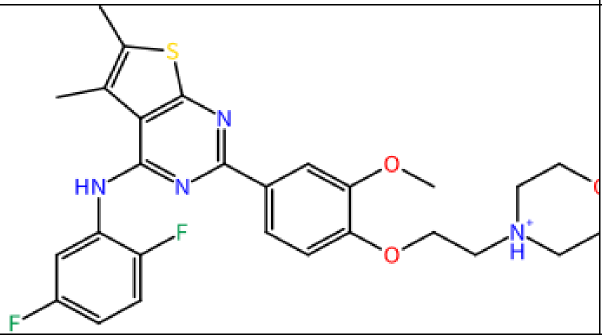	238	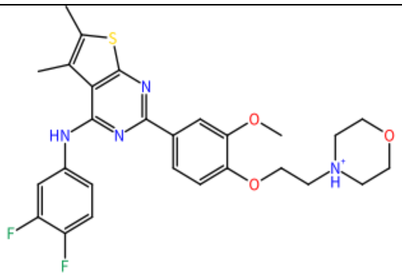
239	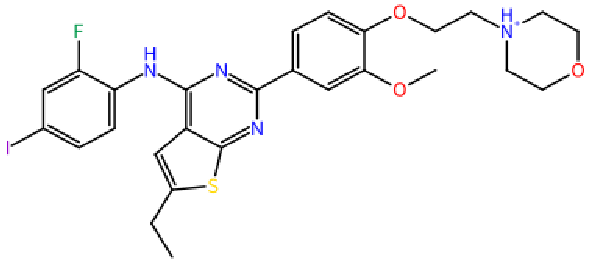	240	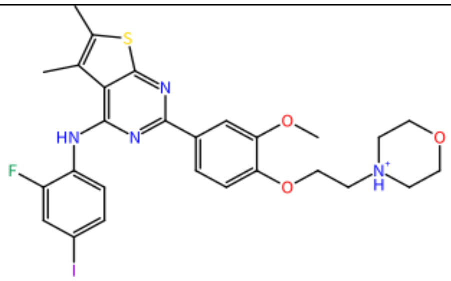
241	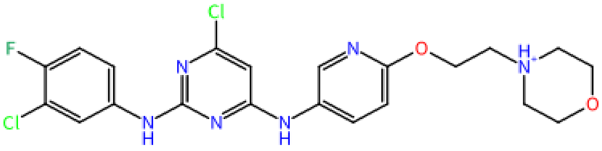	242	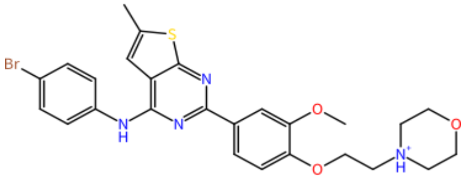

243	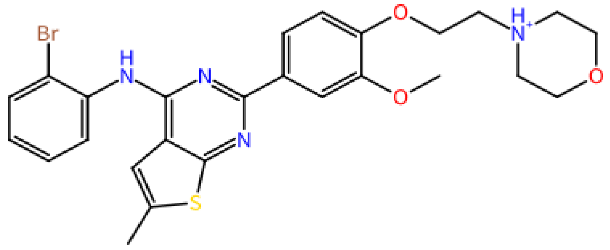	244	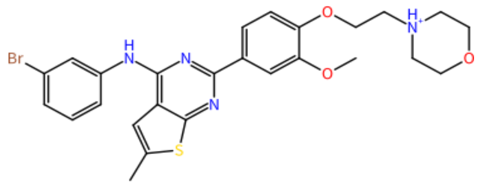
245	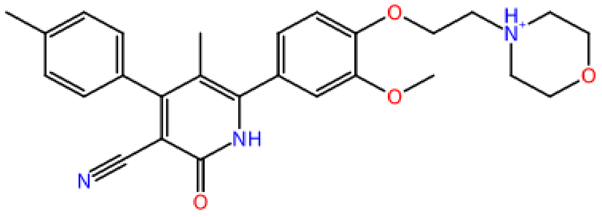	246	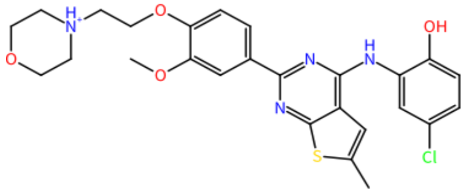
247	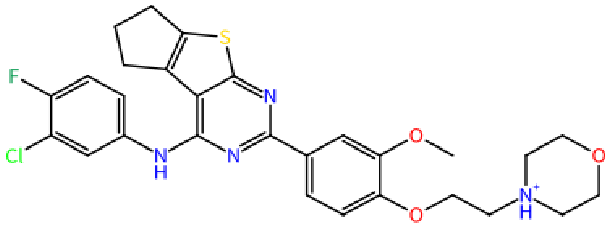	248	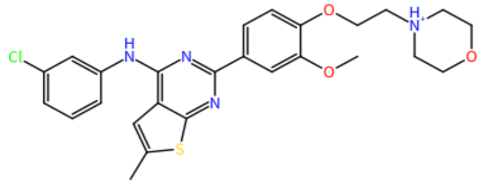
249	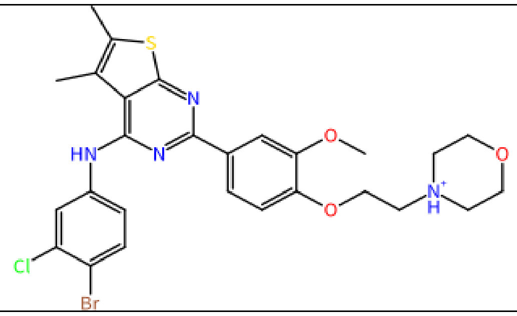	250	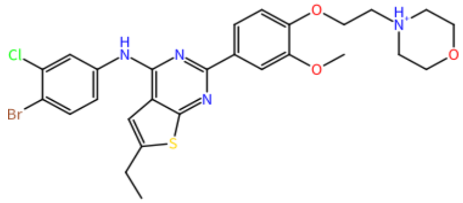
251	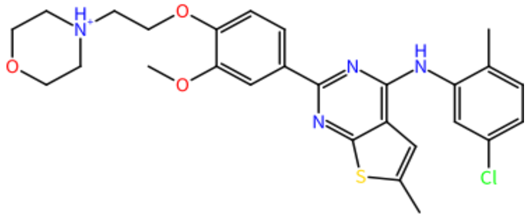	252	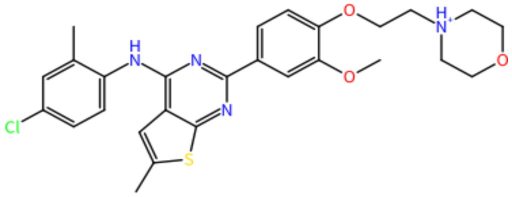
253	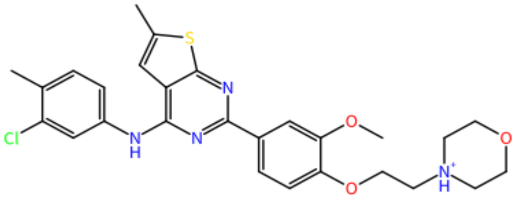	254	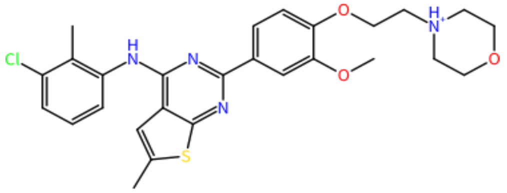

255	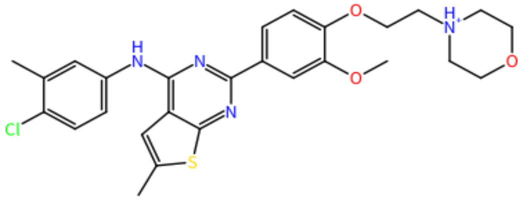	256	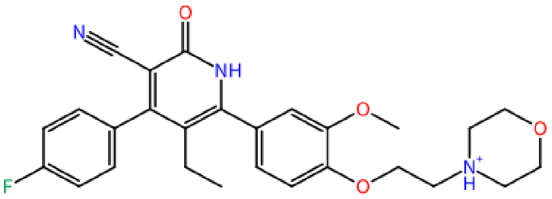
257	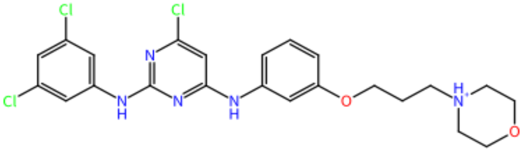	258	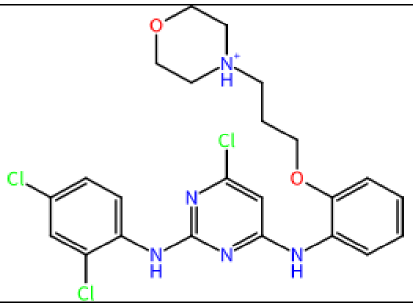
259	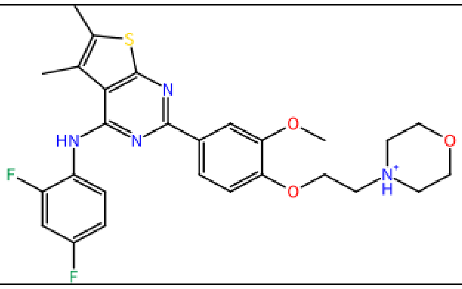	260	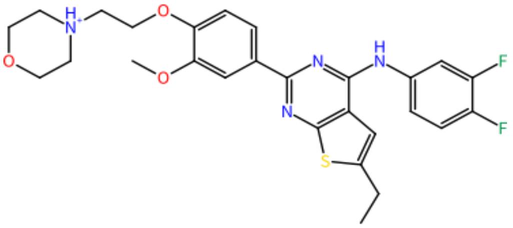
261	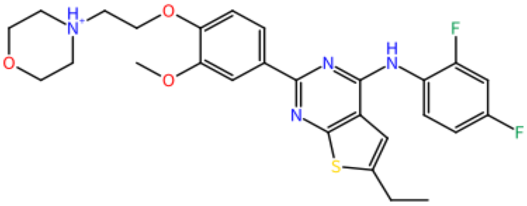	262	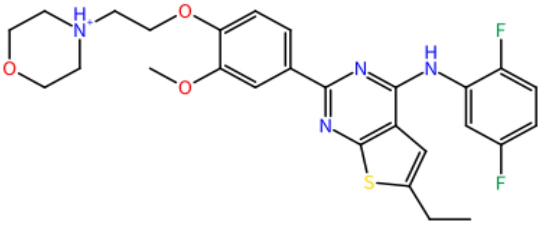
263	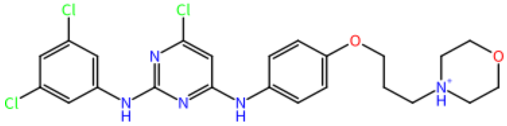	264	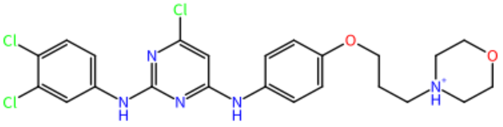
265	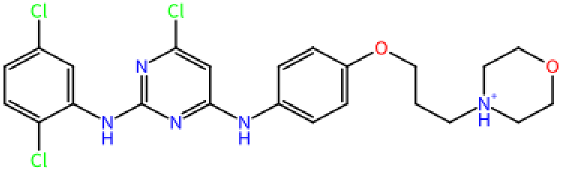	266	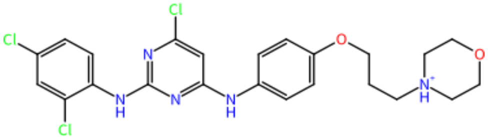

267	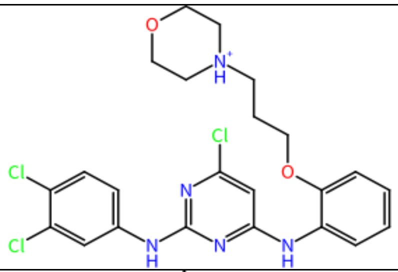	268	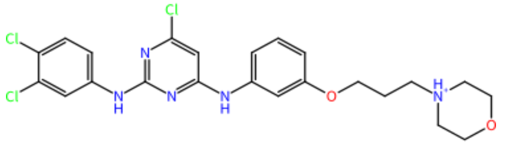
269	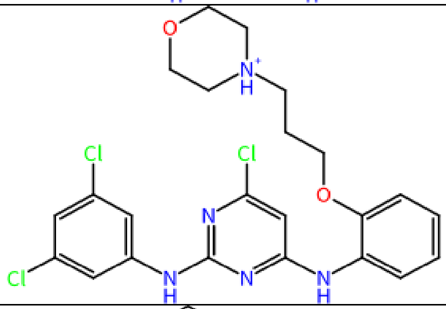	270	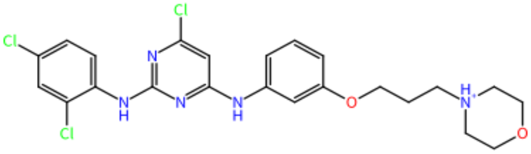
271	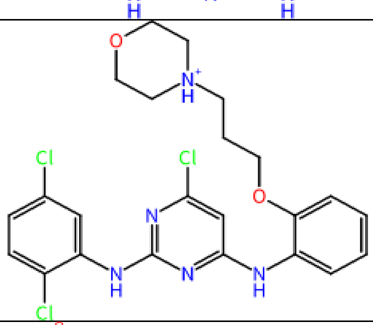	272	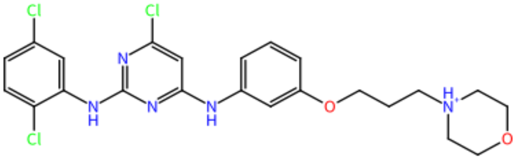
273	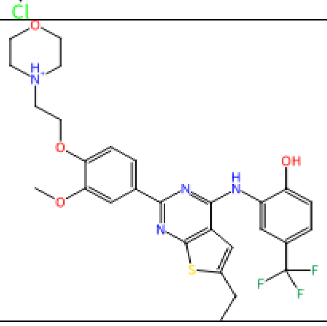	274	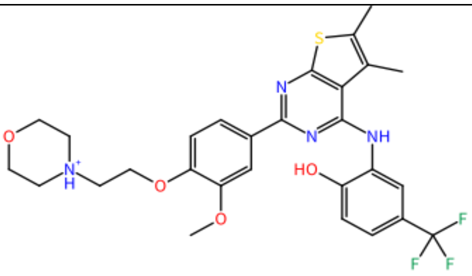
275	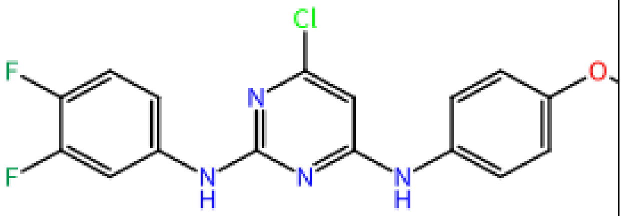	276	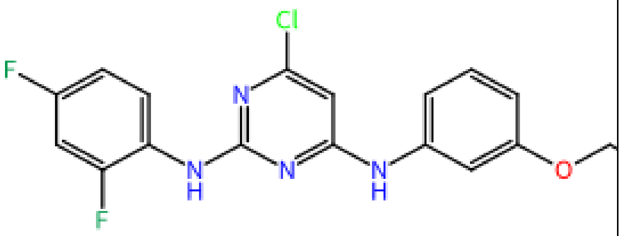

277	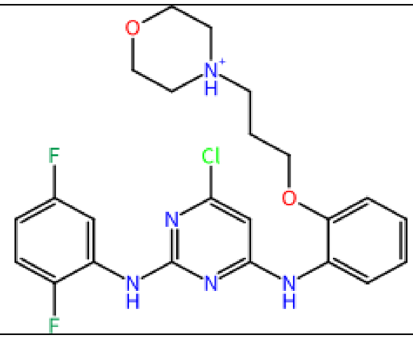	278	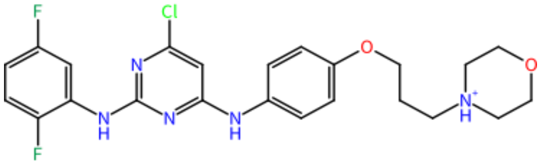
279	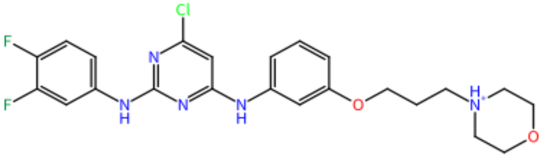	280	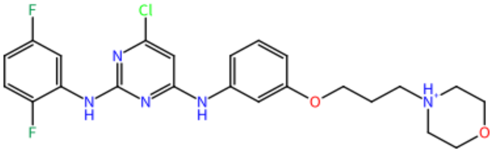
281	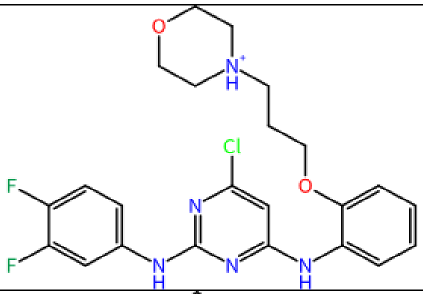	282	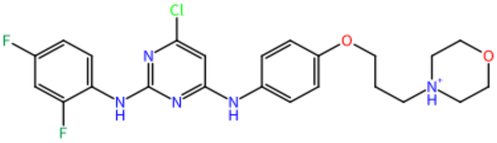
283	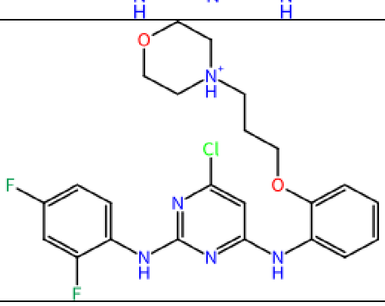	284	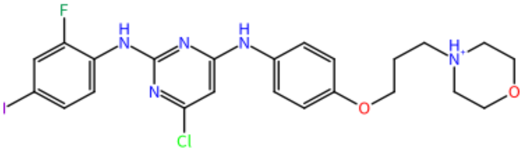
285	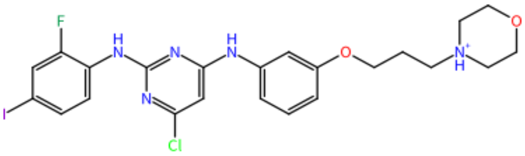	286	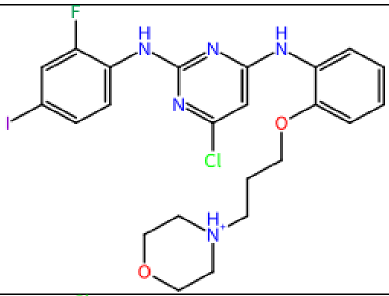
287	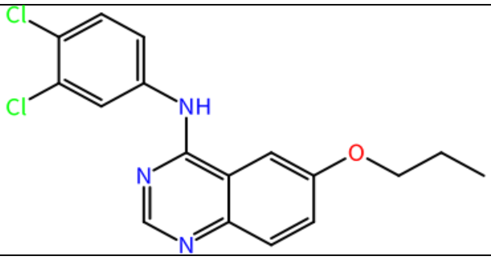	288	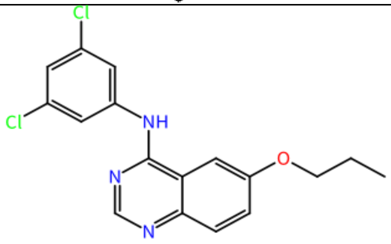

289	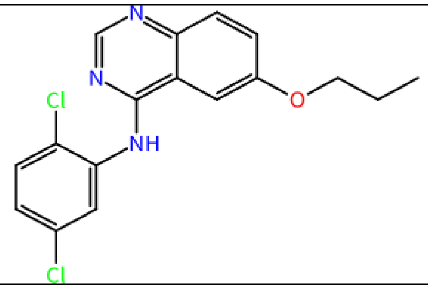	290	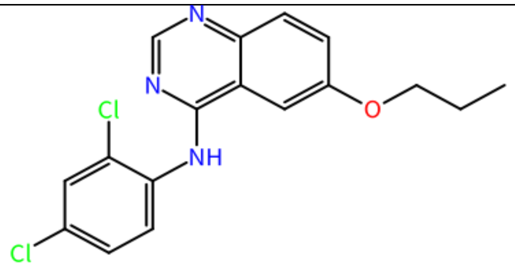
291	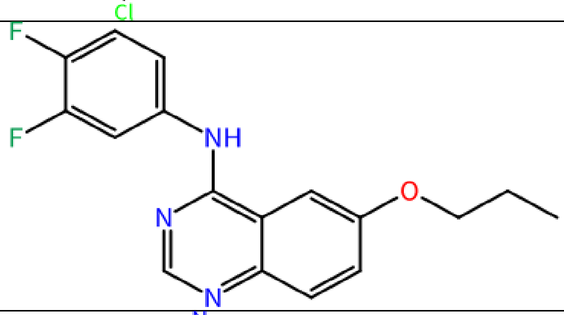	292	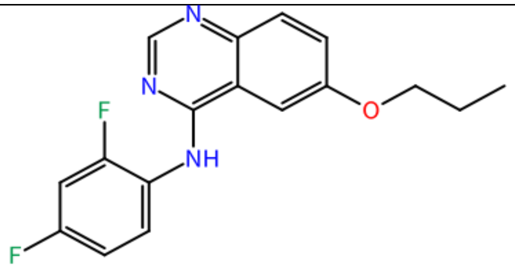
293	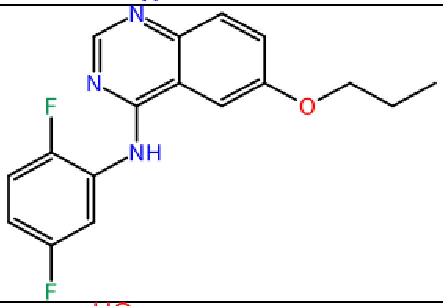	294	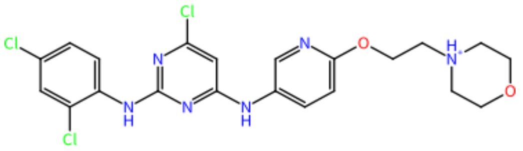
295	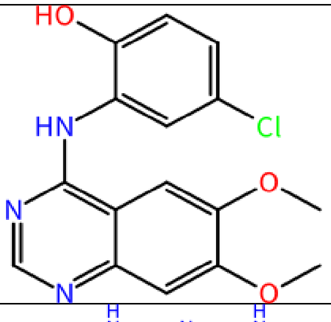	296	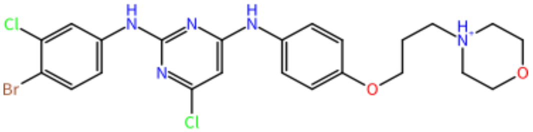
297	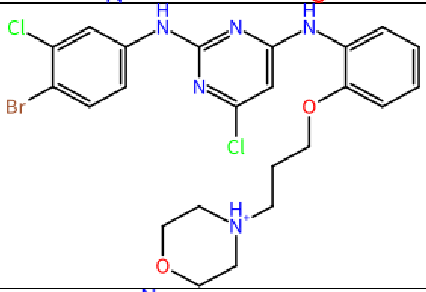	298	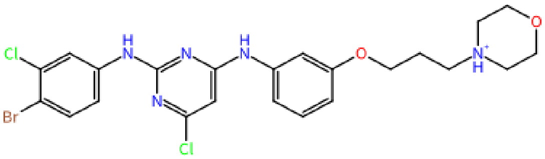
299	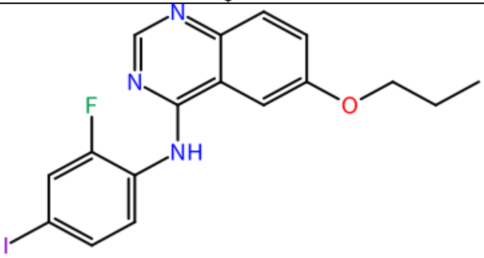	300	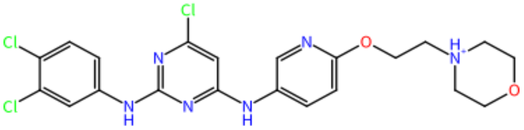

301	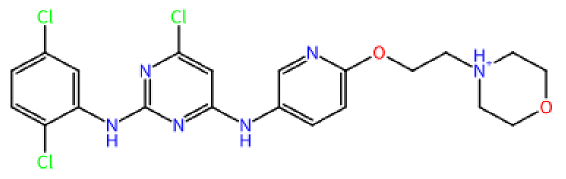

Table S4. HER3 inhibiting compounds generated from molecular editor and inspirator mode of SeeSAR via adding and replacing atoms and group of atoms. (The orange highlighted portion of compounds are representing fragments generated by ReCore).

No	Structure of compounds	No	Structure of compounds
6i	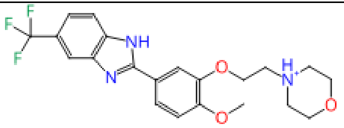	6j	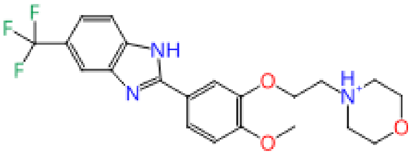
6k	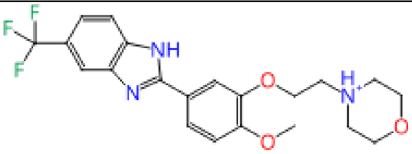	6l	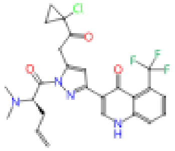
6m	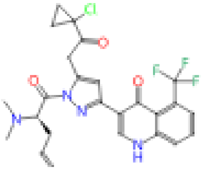	6n	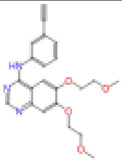
6o	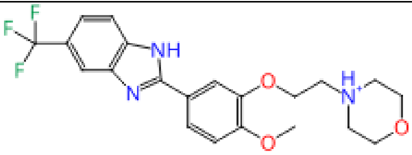	6p	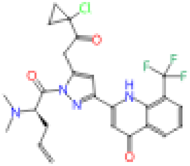
6q	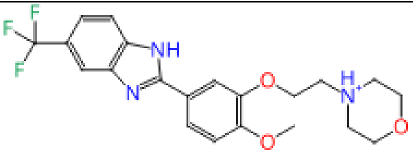	6r	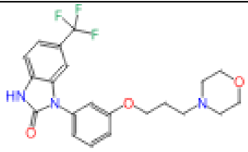
6s	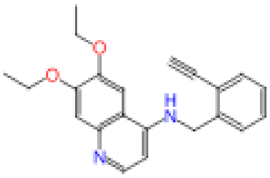	6t	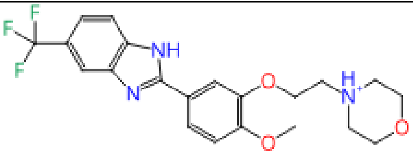
6u	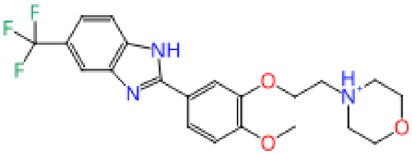	6v	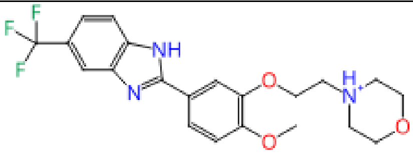
6w	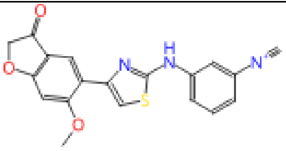	6x	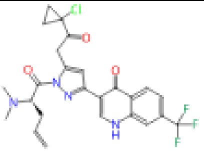

6y	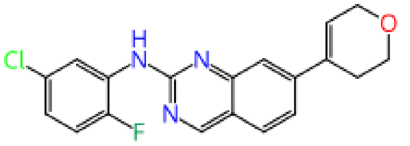	6z	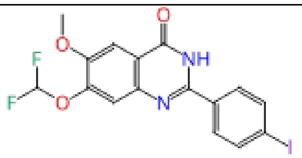
7a	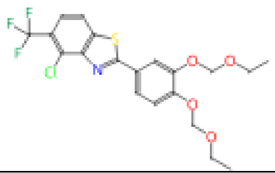	7b	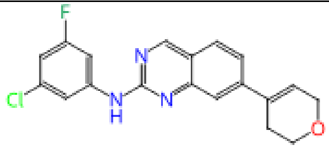
7c	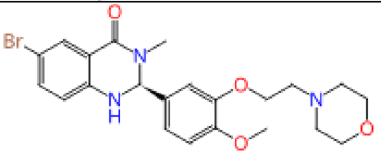	7d	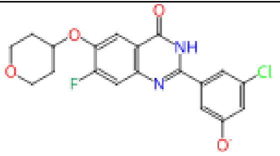
7e	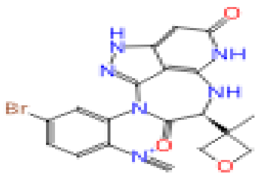	7f	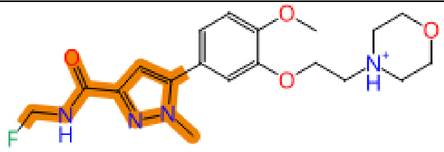
7g	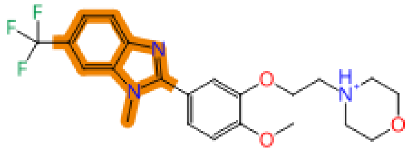	7h	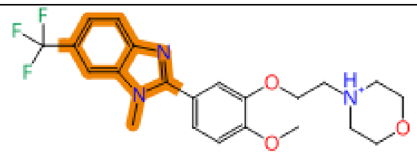
7i	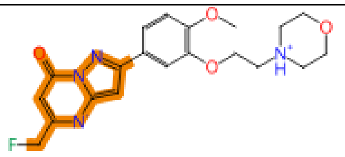	7j	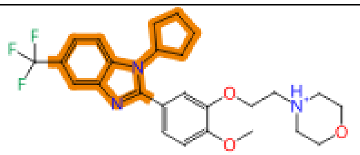
7k	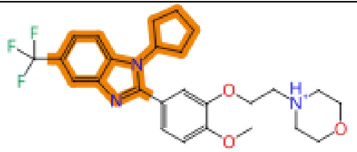	7l	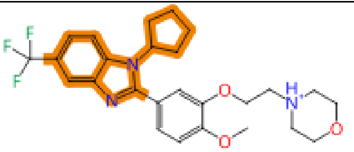
7m	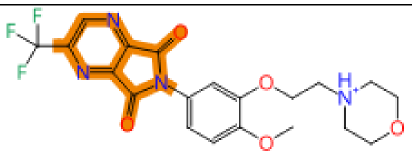	7n	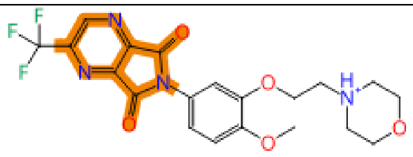
7o	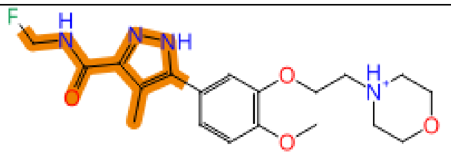	7p	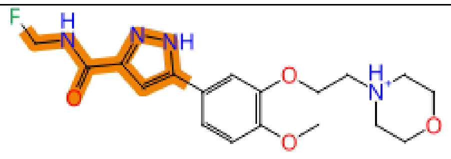
7q	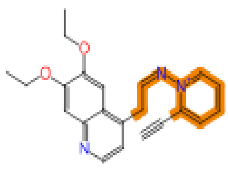	7r	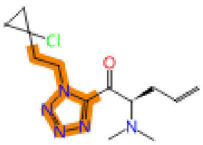

7s	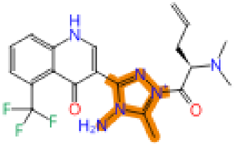	7t	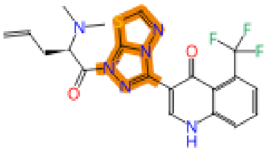
7u	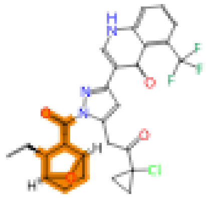	7v	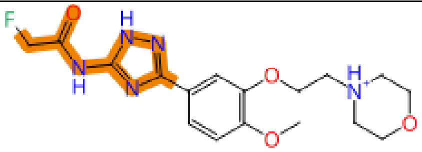
7w	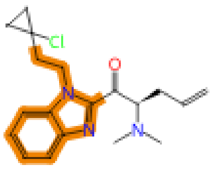	7x	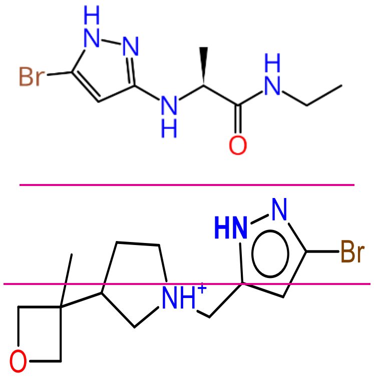
7y	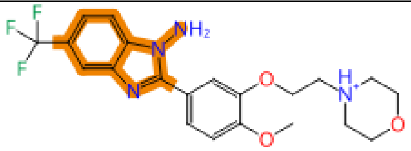	7z	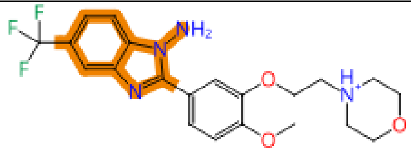
8a	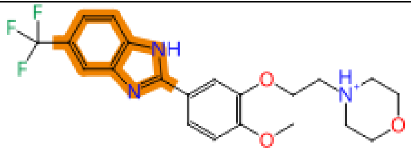	8b	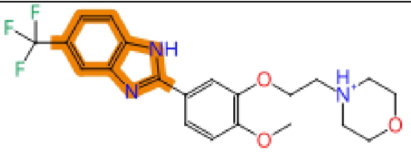
8c	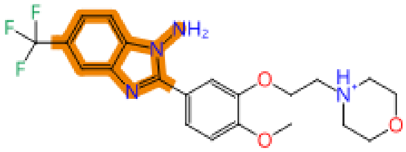	8d	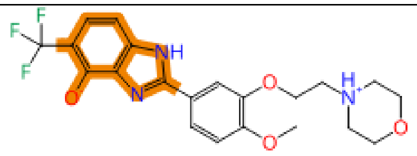
8e	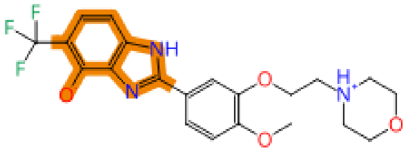	8f	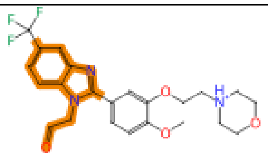
8g	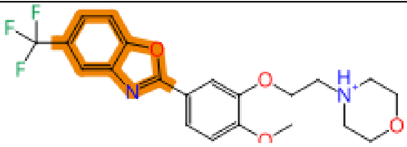	8h	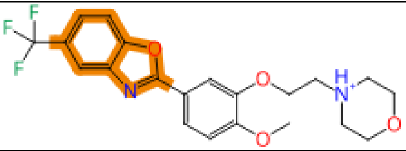
8i	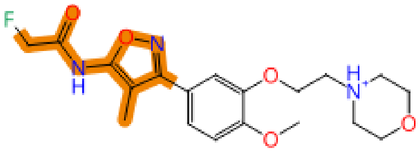	8j	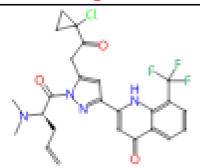
